# How to tame a palladium terminal oxo†
†Electronic supplementary information (ESI) available: Atomic coordinates, energies (Δ*E*, Δ*G*), detailed benchmark tests (basis set, functional, corrections for dispersion and solvent effects), molecular orbital diagrams for **5^PdO^** and **10^PdO^**, natural resonance theory results, details of CASSCF calculations, energies of isomers, other (non)linear correlations, calculation of TEP values and complete ref. 64. See DOI: 10.1039/c7sc05034h


**DOI:** 10.1039/c7sc05034h

**Published:** 2017-12-13

**Authors:** Dominik Munz

**Affiliations:** a Friedrich-Alexander Universität Erlangen-Nürnberg , Egerlandstr. 1 , 91058 Erlangen , Germany . Email: dominik.munz@fau.de

## Abstract

The isolation of terminal oxo complexes of the late transition metals promises new avenues in oxidation catalysis like the selective and catalytic hydroxylation of unreactive CH bonds, the activation of water, or the upgrading of olefins. While terminal oxo ligands are ubiquitous for early transition metals, well-characterized examples with group 10 metals remain hitherto elusive. In search for palladium terminal oxo complexes, the relative stability/reactivity of such compounds are evaluated computationally (CASSCF/NEVPT2; DFT). The calculations investigate only well-known ligand systems with established synthetic procedures and relevance for coordination chemistry and homogeneous catalysis. They delineate and quantify, which electronic properties of ancillary ligands are crucial for taming otherwise highly reactive terminal oxo intermediates. Notably, carbene ligands with both strong σ-donor and strong π-acceptor properties are best suited for the stabilization of palladium(ii) terminal oxo complexes, whereas ligands with a weaker ligand field lead to highly reactive complexes. Strongly donating ligands are an excellent choice for high-valent palladium(iv) terminal oxo compounds. Low coordinate palladium(ii) as well as high-valent palladium(iv) complexes are best suited for the activation of strong bonds.

## Abstract 2

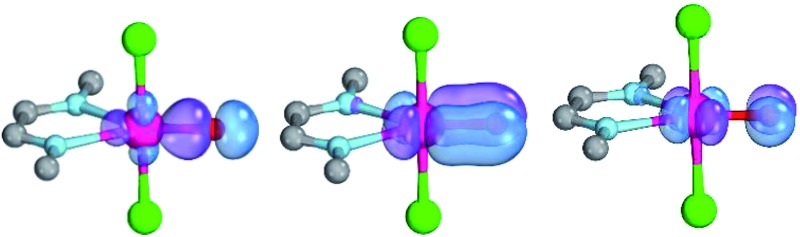
The isolation of terminal oxo complexes of the late transition metals promises new avenues in oxidation catalysis like the selective and catalytic hydroxylation of unreactive CH bonds, the activation of water, or the upgrading of olefins.

## Introduction

Oxo compounds of the late transition metals are alleged intermediates in fundamental chemical processes like the refinement of small molecules through redox catalysis. Prominent examples are the platinum catalyzed oxidation of ammonia to nitric acid by O_2_ (Ostwald process) or CO/NO_*x*_ emission control in the catalytic converters of automobiles by palladium (rhodium, platinum) catalysis.^1^,^2^ Group 10 terminal oxo compounds (*i.e.*, M(O) with M = Ni, Pd, Pt) hold therefore in analogy to the very rich CH oxidation chemistry of early transition metal oxo compounds great promise for selective CH activation or the splitting of water.^3^–^15^ The development of novel catalytic approaches in these areas is highly desirable in the context of green and sustainable oxidation catalysis.^16^–^25^ Collision experiments in the gas phase and computational studies established that naked oxides of the group 10 metals react even with methane to produce methanol.^26^–^32^ Importantly, these gas phase studies substantiate the intermediacy of terminal metal oxides both in heterogeneous as well as in homogeneous reaction media.
1

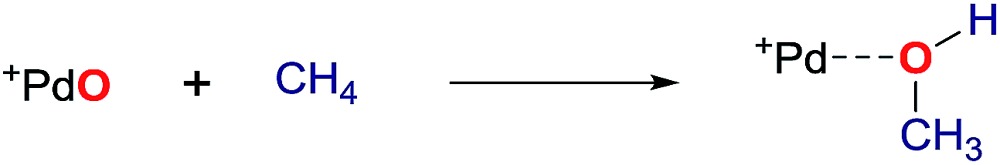




Accordingly, it was already proposed in the early 90s that palladium(iv) terminal oxo compounds constitute intermediates of the oxy-insertion reaction of alkanes with peroxides in the liquid phase.^33^–^37^ High-valent nickel(iii)^38^–^42^ and platinum(iv)^43^ (or surrogates, respectively)^44^–^46^ terminal oxo species were equally suggested as intermediates for hydrocarbon functionalization chemistry.^44^–^46^ It is well known that the isolation of such compounds is very challenging due to the population of anti-bonding molecular orbitals (“oxo wall” for *C*_4v_ symmetry).^47^–^51^ The synthesis, characterization and study of room temperature stable group 10 terminal oxo compounds remains consequently elusive.^2^,^38^,^52^–^55^ Nevertheless, Milstein and coworkers reported one surprising example, where a supposedly platinum(iv) terminal oxo complex was stabilized by an NCP pincer ligand. Whereas the high reactivity of the complex prevented characterization by X-ray crystallography, its oxygen transfer chemistry with carbon monoxide, phosphines, and dihydrogen could be evidenced.^56^

Even more surprisingly than the stability of the complex was the fact that the NCP pincer ligand did not feature strong π-backbonding capabilities.^57^ Up to this report, it was common believe that π-accepting ligands in a trigonal coordination environment should stabilize such compounds by releasing d-electron density from the transition metal center, thereby reducing the electron repulsion between the metal d-electrons and the p-electrons of the oxo ligand.^57^,^58^

2

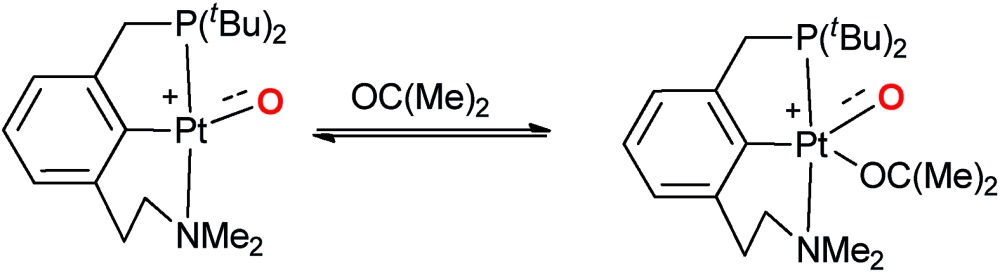




Later on, it was argued on the basis of computational investigations that both the excitation of d-electron density to s- and p-orbitals as well as the coordination of acetone in solution could stabilize this particular complex by supporting out of plane bending of the oxo ligand.^59^ We conclude therefore that the electronic factors leading to highly reactive or comparably stable group 10 terminal oxo compounds are only poorly understood.

Herein, the influence of ancillary ligands on the electronic properties and thermodynamic stability of palladium complexes with terminal oxido ligands is elucidated computationally.^57^ Provided that enough thermodynamic stability can be achieved, sterically encumbering ligands should be able to kinetically protect these reactive species, *i.e.* increase the barrier for O atom transfer.^60^–^62^ Eventually, the isolation and study of a stable terminal oxo compounds will then open up new avenues towards rational catalyst design for various applications.

## Results and discussion

First, high-level quantum chemical calculations on the NEVPT2/CASSCF(8,8) level of theory will be used to reliably model the electronic properties of palladium terminal oxo complexes. Based on these results, a computationally more efficient approach using density functional theory (DFT) is applied. A series of palladium(ii) and palladium(iv) complexes with different monodentate ancillary ligands (amines, pyridines, phosphines, isocyanides, series of carbenes) will be investigated with the latter method. We will show that the thermodynamic stability as well as electronic properties of such palladium(ii) terminal oxo complexes can be qualitatively rationalized through σ-donor/π-acceptor effects of the ancillary ligand. The calculation of the related singlet/triplet gap of the complexes (Pd^II^) allows for a quantification of the reactivity of the terminal oxo moiety. Contrarily, we will show that the π-acceptor effects are not important for the Pd^IV^ complexes, where the overall donor properties of the ancillary ligands determine the thermodynamic stability. Next, these results will be extended to chelating bidentate ligands and tricoordinate pincer-type ligands. Finally, overall trends in reactivity and electronic structure will be identified and implications for synthetic approaches will be discussed.^63^

### Computational methods

All singlet and triplet geometry optimizations were performed with Gaussian09, Rev. D01 (ref. 64) at the B3LYP-D3(BJ)/def2-TZVP^65^–^68^ (valence triple-ζ plus single polarization basis set)^69^ level of theory using the def2 quasirelativistic effective core potential (ECP, 28 core electrons) and the related def2-TZVP valence basis set for palladium as obtained from the emsl basis set exchange data base,^69^,^70^ and applying the D3 dispersion correction^71^ with Becke–Johnson damping.^72^ The restricted formalism was used for closed-shell singlet multiplicities. No symmetry or internal coordinate constraints were applied during optimizations. All reported optimized structures were verified as true minima by the absence of negative eigenvalues in the harmonic vibrational frequency analysis and all wave functions were checked for undesired internal instabilities. The energies of all structures were corrected by single-point calculations with ORCA (version 3.0.3) on the B2PLYP(COSMO, D3BJ)/def2-TZVPP level of theory, including corrections for solvent effects (except the structures shown in Table 1) with the conductor like screening model COSMO^73^ (solvent: hexane). The RIJCOSX approximation was used to speed up the calculations. Test calculations revealed negligible deviation from calculations without the RIJCOSX procedure. Tighter than default scf convergence criteria (tightscf), and finer than default grids (grid5; finalgrid6) were used as implemented in ORCA. Structures optimized with B3LYP and B2PLYP showed very similar structural parameters. The chosen computational method was benchmarked for the influence of functional (B3LYP, PBE0, M06, M06L, B2PLYP, B2GP-BLYP, PWP-B95), basis set [def2-SVP, def2-TZVP, def2-TZVPP, 6-31G(d), 6-311G(d,p), 6-311++G(d,p)] and dispersion and solvent corrections on experimental and computational results (structural parameters, multiplicities, energies) with Milstein's Pt^IV^ complex^56^,^59^ and Cundari's CASSCF(8,8) study^44^ on truncated Pd^IV^ bisimine complexes. For details, see the ESI.† Importantly, B3LYP, PBE0, M06 and M06L provided similar optimized structural parameters and consistent energy profiles, whereas it was found that triple-ζ basis sets are much better suited for the geometry optimizations than double-ζ basis sets. For energies of conformational and stereoisomers, see the ESI† as well. Structural optimizations using the complete active space SCF (CASSCF) method were performed with ORCA 3.0.3. Finer than default grid setting were chosen for the RIJCOSX approximation (IntAccX 4.34, 4.34, 4.67; GridX 2,2,2). The selection of the active space is delineated in more detail in the ESI.† The def2-ECP and related def2-TZVPP valence basis set was used as implemented in ORCA for Pd in conjunction with the def2-TZVPP all-electron basis set for the other elements. Effects of dynamic electron correlation were included by the second order multireference perturbation method NEVPT2.^74^ The optimized CASSCF structures were verified as true minima through numerical calculation of the hessian. The DLPNO-CCSD(T) calculations (“NORMAL” settings) were performed accordingly on the DLPNO-CCSD(T)/def2-TZVPP//B3LYP/def2-TZVP level of theory using ORCA 4.0.1. All energies reported – except in Table 1, which shows electronic energies – are Gibbs free energies under standard conditions (*T* = 298 K, *p* = 1 atm) using unscaled frequencies. Approximate Gibbs free energies were obtained through thermochemical analysis of the B3LYP-D3(BJ)/def2-TZVP optimized structures, using the thermal corrections to the Gibbs free energy as reported by Gaussian09 (including zero-point effects, thermal enthalpy corrections, and entropy) and the electronic energy including solvent effects and dispersion corrections from the single point calculations with ORCA. For a comparison to Δ*E* values, which reflect the same trends, see the ESI.† The biradical open-shell singlet electronic states were obtained through the broken-symmetry formalism *via* the flip-spin procedure of ORCA with the optimized triplet structures. Spin-decontamination through the Yamaguchi formula^75^–^77^ did not have a considerable influence on the obtained energy differences. The nature of the open-shell biradical wave functions was verified using corresponding orbitals and spin populations.^78^ Natural Bond Orbitals (NBOs) were calculated with NBO 6.0.^79^ The energy decomposition analysis (EDA) and analysis of fragment molecular orbitals was performed with AOMix v6.88 based on wave functions computed with Gaussian09 on the B2PLYP(D3BJ)/def2-TZVPP//B3LYP-D3/def2-TZVP level of theory.^80^,^81^ The EDA decomposes the overall bonding interaction energy (*E*^int^) in orbital (*E*^orb^: charge transfer and polarization effects) and steric contributions (*E*^steric^: electrostatic and Pauli exchange energy). The Tolman electronic parameter of the CAAC, MIC, and DAC ligands were calculated according to Gusev's procedure with Gaussian09, rev. D01.^82^,^83^ Calculated structures, molecular orbitals and spin densities were visualized with IboView,^84^ Molden 5.6,^85^ and Avogadro 1.2.0.^86^

**Table 1:** Electronic structures of Pd^IV^ terminal oxo complex with bisimine ligand (t: triplet; o.s.s.: open-shell singlet; s: closed-shell singlet) as predicted by CASSCF(8,8) and DFT. Electronic energies are given in [kcal mol^–1^]

	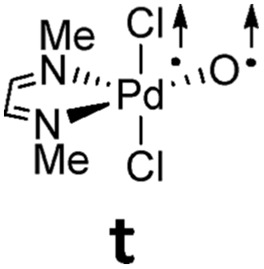	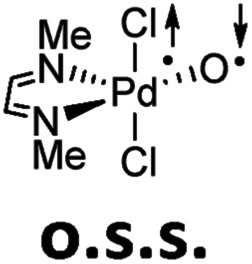	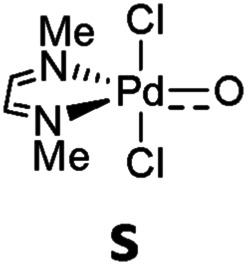
Δ*E* CASSCF(8,8)/def2-SVP	+2.0	0	+6.3
Δ*E* CASSCF(8,8)/def2-TZVPP	+3.1	+2.3	0
Δ*E* CASSCF(8,8)/NEVPT2^*a*^	+1.6	+4.9	0
Δ*E* B2PLYP/def2-TZVPP	+1.6	+6.9	0
Δ*E* B2PLYP//B3LYP^*b*^	+1.8	+9.6	0
Δ*E* B3LYP/def2-TZVPP	–13.8	n.d.	0
Δ*E* DLPNO-CCSD(T)//B3LYP^*c*^	0	n.d.	+4.7

^*a*^CASSCF(8,8)/NEVPT2/def2-TZVPP.

^*b*^B2PLYP/def2-TZVPP//B3LYP-D3/def2-TZVP.

^*c*^DLPNO-CCSD(T)/def2-TZVPP//B3LYP/def2-TZVP.

### Multireference character and perturbation theory results

Computational modeling of late transition metal terminal oxo compounds is a challenge for single-determinantal DFT.^87^ In order to investigate the multireference character of a model Pd^IV^ oxo complex with a truncated bisimine ligand (ethane-1,2-dimine), a CASSCF(8,8) study using the 6-31+G(d,p) basis set was reported.^44^ The calculations, which included the π-system of the imine ligand as well as the σ- and one π-bond of the oxo ligand with the metal, suggested surprisingly that the molecule should have an open-shell singlet ground state. Indeed, we obtained even for the larger ligand with *N*-methyl substituents (*N*^1^,*N*^2^-dimethyl ethane-1,2-dimine) as well an open-shell singlet ground state using the def2-SVP basis set (Table 1; Δ*E*_s/t_ = +2.0 kcal mol^–1^). However, when using a larger basis set (def2-TZVPP), the closed-shell singlet state becomes the ground state, whereas the triplet (CASSCF(8,8): Δ*E*_s/t_ = +3.1 kcal mol^–1^) and open-shell singlet states (CASSCF(8,8): Δ*E*_o.s.s._ = +2.3 kcal mol^–1^) get less favorable.^88^

**Fig. 1:**
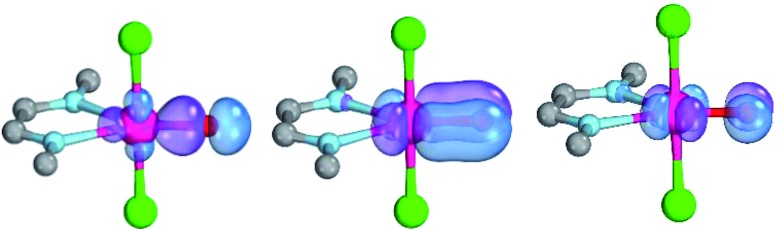
CASSCF(8,8) calculated coordination geometry and molecular orbitals for Pd^IV^ complex with bisimine ligand in the singlet state (left to right: Pd–O σ-orbital, ON: 1.90; Pd–O π-orbital, ON: 1.78; Pd–O π*-orbital; ON: 0.22).

The calculations are likewise in favor of a closed-shell singlet ground state, if dynamic correlation effects are considered *via* the n-electron valence state perturbation theory NEVPT2 correction^74^ on top of the converged CASSCF(8,8) wave function (CASSCF(8,8)/NEVPT2: Δ*E*_s/t_ = +1.6 kcal mol^–1^; Δ*E*_o.s.s._ = +4.9 kcal mol^–1^). We consequently believe that an open-shell singlet ground state is quite unlikely, which is also indicated by state averaged CASSCF calculations (ESI†). The closed-shell singlet state shows a slightly distorted trigonal planar coordination geometry (Fig. 1). The occupation numbers (ON) of the Pd=O bonding σ-orbital (1.9) and Pd=O bonding π-orbital (ON: 1.8) indicate moderate population of the respective σ*- (ON: 0.1) and π*- (ON: 0.2) anti-bonding orbitals and consequently strong double bond character with an overall PdO bond order between one and two.

It was reported for Milstein's platinum(iv) complex that the double-hybrid functional B2GP-BLYP predicted the experimentally observed singlet multiplicity much better than the hybrid functional PBE0, which is known to be biased towards the high-spin state.^59^,^89^ Indeed, although the optimized structural parameters are very similar to the CASSCF(8,8) results, also B3LYP predicts a fairly large singlet/triplet gap of Δ*E*_s/t_ = –13.8 kcal mol^–1^ in favor of the triplet state (Table 1). Additionally, it appears interesting to note that single-reference coupled cluster calculations on the DLPNO-CCSD(T) level of theory predict a triplet ground state as well (Δ*E*_s/t_ = –4.7 kcal mol^–1^).^90^ Contrarily, the CASSCF(8,8)/NEVPT2 results could be perfectly reproduced on the computationally demanding B2PLYP/def2-TZVPP level of theory (Δ*E*_s/t_ = +1.6 kcal mol^–1^). Geometry optimization with the open-shell singlet biradical wavefunction^91^,^92^ predicted in agreement with the NEVPT2 results (Δ*E*_o.s.s._ = +4.9 kcal mol^–1^) the open-shell singlet state (Δ*E*_o.s.s._ = +6.9 kcal mol^–1^) to be higher in energy than the high-spin triplet state (Δ*E*_o.s.s._ = +1.8 kcal mol^–1^). Fortunately, the singlet–triplet gap could as well be described well by single-point calculations on the B2PLYP/def2-TZVPP//B3LYP/def2-TZVP level of theory (Δ*E*_s/t_ = +1.8 kcal mol^–1^). For the determination of the open-shell singlet energy, the very similar geometrical parameters of the triplet state and the flip-spin procedure as implemented in ORCA was used. Here, the stability of the open-shell singlet state was a bit underestimated supposedly due to the unrelaxed open-shell singlet structure (Δ*E*_o.s.s._ = +9.6 kcal mol^–1^).

Altogether, both DFT and CASSCF give coherent results and confirm that closed-shell singlet and triplet state energies are close in energy, with a closed-shell singlet ground state. Notably, also single-point calculations on the B2PLYP//B3LYP level of theory give reliable singlet/triplet gap energies. Therefore, all the following results refer to the latter level of theory. For further benchmarking studies relating to the choice of functional, basis set, and influence of dispersion and solvation effects, see the ESI (Fig. S1, Tables S1 and S2†).

#### Monodentate ligands

**Chart 1:**
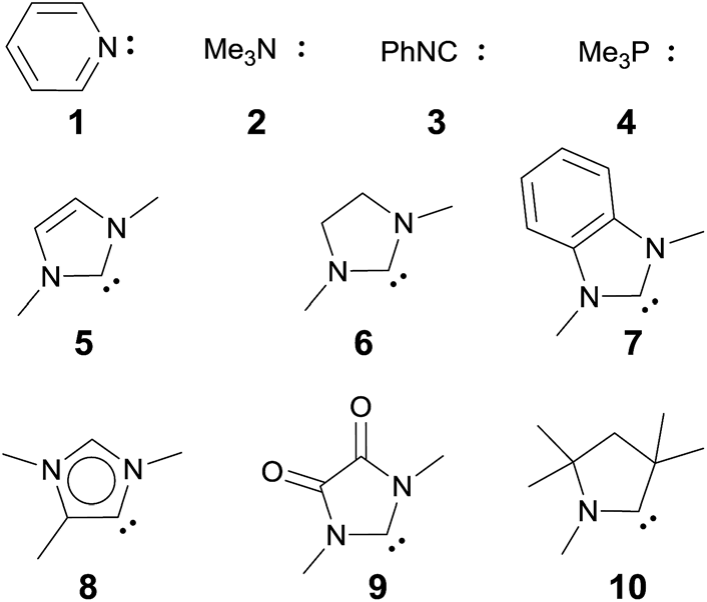
Evaluated monodentate ligands.

A series of electronically distinct ligands was chosen in order to evaluate the electronic influence of two-electron donor (“L-type”) ligands on the properties of palladium terminal oxo complexes (Chart 1). Only ligands with well-developed synthetic procedures were investigated. Note that Hillhouse and Mindiola used chelating bis-NHC and bis-phosphine ligands for the seminal synthesis of the isolobal nickel-imido complexes.^93^–^96^ Likewise, N-donor ligands (Py, **1**; NMe_3_, **2**), which are commonly applied in oxidation catalysis as well as isonitriles (PhNC, **3**) and phosphines (PMe_3_, **4**) were included.

We anticipated that carbene ligands^97^–^99^ qualify as ideal candidates for two reasons. First, they are (unlike phosphines) typically stable under oxidative conditions. Second, there is a large variety of electronically distinct free carbenes beyond the very well-known imidazole-2-ylidenes (NHC, **5**) available for the convenient synthesis of transition metal carbene complexes. Examples range from dihydroimidazole-2-ylidenes (saNHC, **6**) and benzimidazolylidenes (benzNHC, **7**) to mesoionic carbenes (MICs, **8**),^100^,^101^ diamidocarbenes (DACs, **9**)^102^ and cyclic (alkyl)(amino) carbenes (CAACs, **10**).^103^,^104^ A popular method for the description of the donor properties of ligands is the Tolman electronic parameter (TEP). The TEP, which is obtained through determination of the CO stretching frequencies of (L)Ni(CO)_3_ complexes, is related to the overall donor properties of a ligand and combines both σ- and π-effects as well as steric contributions.^105^,^106^ According to the TEP, mesoionic carbene ligands (**8**), which do not show considerable π-accepting properties, are exceptionally strong donor ligands. The electron deficient diamidocarbene **9** is expected by the TEP to be a very weak donor ligand. In extension, the ^13^C and ^15^N NMR shifts of the free carbenes,^107^^31^P and ^77^Se of phosphinidene and selenium adducts,^108^–^110^ and DFT calculations^111^–^117^ allowed for deconvolution of both σ-donor and π-acceptor effects.

**Fig. 2:**
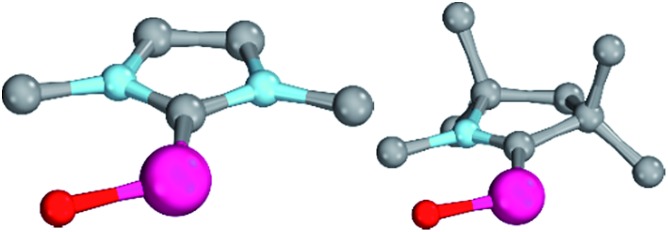
Structural parameters of (NHC)PdO (**5^PdO^**, left side) in comparison to (CAAC)PdO (**10^PdO^**, right side) in the singlet states. **5^PdO^**: C–Pd–O 107.7°, Pd–O 1.80 Å, Pd–C 1.97 Å, H–O 2.00 Å. **10^PdO^**: C–Pd–O: 110.9°, Pd–O 1.79 Å, Pd–C 1.93 Å, H–O 2.01 Å.

Magnetic circular dichroism studies allowed to evaluate the influence of NHC ligands on the ligand field of iron(ii) complexes.^118^,^119^ It has been reported that an ordering of both carbene ligands and N-donor as well as P-donor ligands according to their ligand field is a challenging task due to side effects with the ancillary ligands.^119^ Nevertheless, it can be argued that CAAC (**10**) ligands with their very strong σ-donor and π-acceptor capabilities should be extraordinary strong field ligands. Consequently, benzNHCs (**7**, fairly strong σ-donor, fairly weak π-acceptor), NHCs (**5**, strong σ-donor, weak π-acceptor), and especially N-donor (**1**, **2**) and P-donor ligands (**4**) should lead to smaller ligand field splittings. Optimizing the structures of the LPd^II^O complexes with these monodentate ligands L (B2PLYP(COSMO,D3)/def2-TZVPP//B3LYP/def2-TZVP) reveals that the singlet states are lower in energy than the triplet states for all the investigated carbene ligands (*vide infra*). Strongly bent coordination geometries with short distances between the oxo moiety and the *N*-methyl substituents of the carbene ligands (Fig. 2; **5^PdO^**: CH_3_–O 2.00 Å; **10^PdO^**: CH_3_–O 2.01 Å) are predicted for all singlet state structures, whereas pseudo-linear structures were optimized for the open-shell states (ESI†). The linear coordination geometry represents however a transition state for the closed-shell molecules. The complex with an NHC ligand (**5^PdO^**) shows a C–Pd–O angle of 107.7° and the CAAC complex (**10^PdO^**) of 110.9° (Fig. 2). The short distances between the oxo atom and the *N*-methyl substituents indicate weak hydrogen type bonding, which further stabilizes the terminal oxo group through delocalization of electron density.^120^,^121^ Note that it has been shown that hydrogen bonding interactions can be crucial for the stabilization of terminal oxo groups.^122^ Most importantly, the computed Pd–O bond length is predicted to be significantly shortened with 1.79 Å (**5^PdO^**: 1.80 Å) in comparison with crystallographically characterized Pd–O single bonds, which are typically around 2.06 Å.^44^

**Fig. 3:**
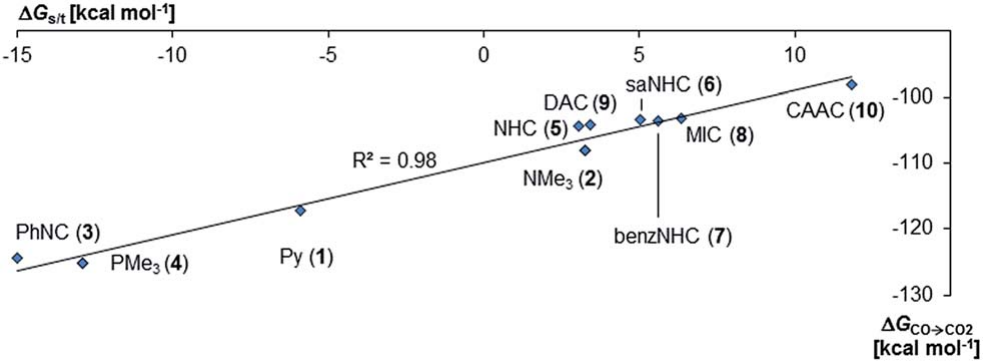
Correlation of singlet/triplet gap (Δ*G*_s/t_ = *G*_triplet_ – *G*_singlet_) of palladium(ii) complexes with monodentate ancillary ligands with Gibbs free energy for the oxidation of CO to CO_2_ (Δ*G*_CO→CO_2__) by the complexes in the singlet state.

The shorter bond between the CAAC ligand and the palladium atom (1.93 Å) for **10^PdO^** in comparison to the NHC complex **5^PdO^** (1.97 Å) is indicative of stronger interaction between the metal atom and the carbene ligand (Fig. 3). The Mayer bond indices for the Pd–O bond indicate considerable multiple bond character (**5^PdO^**: 1.47; **10^PdO^**: 1.53) and are in good agreement with the natural atomic orbital Wiberg bond index for oxygen (**5^PdO^**: 1.40; **10^PdO^**: 1.46). Natural resonance theory^123^ predicts as well a considerable double bond character (ESI, Fig. S4†), although the resonance weights for a double bond description are comparably small with 24% for a Pd=O double bond for **5^PdO^** and 23% for **10^PdO^**, respectively.

Complementing the partial double bond character of the Pd–O bond, considerable negative partial charge is localized on the terminal oxo atom (**5^PdO^**: Löwdin: –0.48 a.u.; Hirshfeld: –0.60 a.u., NPA: –0.94 a.u.; **10^PdO^**: Löwdin: –0.50 a.u.; Hirshfeld: –0.62 a.u.; NPA: –0.95 a.u.). Also note the higher accumulation of positive charge on the palladium atom for L = CAAC *vs.* L = NHC, which suggests enhanced relieve of d-electron density for **10^PdO^**.

(CAAC: Löwdin +0.14 a.u.; Hirshfeld: +0.35 a.u.; NPA: +0.64 a.u.; **5^PdO^**: Löwdin: +0.12 a.u.; Hirshfeld: +0.33 a.u.; NPA: +0.62 a.u.). The computational results suggest hence a fairly strong electrostatic interaction between the palladium and oxygen atoms. The bond properties of the oxygen – palladium bonds were furthermore investigated by Morokuma's energy decomposition analysis (EDA), which decomposes the overall bonding interaction (*E*^int^) in orbital (*E*^orb^) and steric contributions (*E*^steric^).^124^–^126^ Indeed, the Pd–O bond (Table 2) of **10^PdO^** was found to be stronger (*E*^int^ = –100.0 kcal mol^–1^) than the one of the NHC complex **5^PdO^** (*E*^int^ = –97.2 kcal mol^–1^), which *vice versa* suggests higher reactivity (*i.e.* lability of the Pd–O bond) for the latter. Notably and in agreement with the analysis of the double bond character and geometrical parameters, this is mainly due to more favorable orbital interactions (*E*^orb^ = –230.0 kcal mol^–1^) for the CAAC complex **10^PdO^** in comparison to the NHC analogue **5^PdO^** (*E*^orb^ = –222.3 kcal mol^–1^).

**Table 2:** Energy decomposition analysis (*E*^int^ = *E*^orb^ + *E*^steric^) of the palladium – oxygen bonds of (CAAC–Pd)–O (**10^PdO^**) and (NHC–Pd)–O (**5^PdO^**) in the closed-shell singlet states. Values are given in [kcal mol^–1^].^127^

Fragments	Complex	*E* ^int^	*E* ^orb^	*E* ^steric^
**(CAAC)Pd–O**	**10^PdO^**	–100.0	–230.0	+130.0
**(NHC)Pd–O**	**5^PdO^**	–97.2	–222.3	+125.1

The bonding interaction between the carbene ligands and the palladium monoxide molecular fragment in the closed-shell singlet state ([^1^Σ^+^(π*^4^)])^32^ was as well investigated by energy decomposition (ESI, Table S3†). This approach corresponds to the picture of an “ancillary ligand stabilized palladium monoxide”. The decomposition analysis suggests also for this covalent bond a stronger interaction between the CAAC and PdO (*E*^int^ = –65.8 kcal mol^–1^) than for the NHC complex (*E*^int^ = –59.6 kcal mol^–1^).

The analysis of the Löwdin orbital population and the molecular orbital interaction diagram for **10^PdO^** (ESI, Fig. S2†; fragments: PdO and the CAAC ligand) favor an overall Pd–O bond order between one and two, with the occupation of two π* antibonding orbitals of the Pd–O bond. Moderate (≈1 a.u.) excitation of 4d-electron density to the 5s and 5p orbitals is predicted. The bent coordination geometry of the oxo ligand optimizes the bonding and antibonding interactions between the oxo p- and metal d-orbitals through mixing of the d-orbitals.^39^

**Scheme 1:**
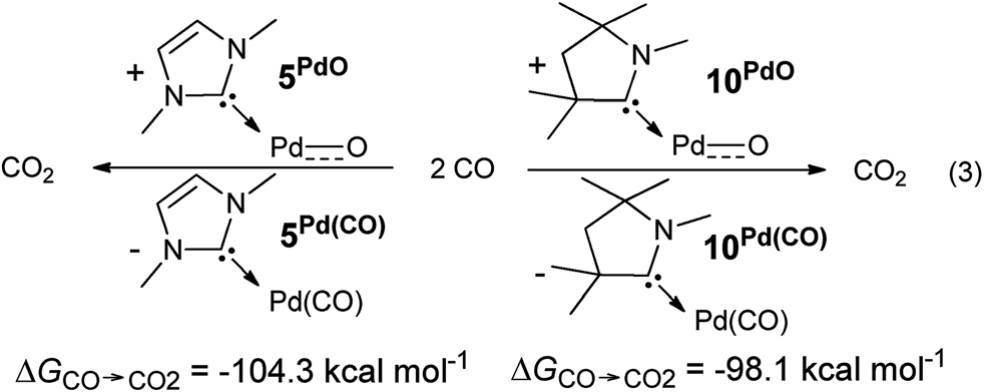
Oxidation of CO to CO_2_ by palladium(ii) terminal oxo complexes **5^PdO^** and **10^PdO^**.

The oxygen atom transfer to carbon monoxide (*i.e.* the oxidation of carbon monoxide to carbon dioxide) by the CAAC complex **10^PdO^** proceeds highly exergonic (**10^PdO^**: Δ*G*_CO→CO_2__ = –98.1 kcal mol^–1^, Scheme 1) and therefore confirms that the complex is indeed a strong oxidant with strong O-atom transfer capability.

Interestingly, the oxygen atom transfer from the NHC complex **5^PdO^** proceeds even more exergonic (**5^PdO^**: Δ*G*_CO→CO_2__ = –104.3 kcal mol^–1^). This is in perfect agreement with the analysis of the molecular orbital interactions and indicates that **5^PdO^** is more reactive than **10^PdO^**. Markedly, the reduced exergonicity for the oxidation of CO by **10^PdO^** relative to the oxidation by **5^PdO^** is also in line with the shorter Pd–O bond length (*vide supra*, **5^PdO^**, 1.80 Å; **10^PdO^**, 1.79 Å), which should be related with the palladium–oxo bond strength (Badger's rule) and the oxophilicity^38^ of the (carbene)Pd fragment.

Next, the Gibbs free energy for the oxidation of CO to CO_2_ (Δ*G*_CO→CO_2__) was studied as well for all the other ligands shown in Chart 1. Remarkably, the very strong field CAAC ligand led to the least exergonic oxidation reaction (Δ*G*_CO→CO_2__ = –98.1 kcal mol^–1^), *i.e.* the CAAC stabilizes the terminal oxo ligand the most (Fig. 3). The Δ*G* values for ligands with a supposedly weaker ligand field like pyridine **1** (Δ*G*_CO→CO_2__ = –117.3 kcal mol^–1^), the isonitrile ligand **3** (Δ*G*_CO→CO_2__ = –123.3 kcal mol^–1^) or the phosphine PMe_3_**4** (Δ*G*_CO→CO_2__ = –123.3 kcal mol^–1^) indicate thermodynamically much less stable terminal oxo complexes. The dihydroimidazolylidene (saNHC, **6**) and benzimidazolylidene (benz-NHC, **7**) ligands with their moderate π-accepting properties were predicted – in perfect agreement with the expectations – to be between the imidazolylidene (NHC, **5**) and the CAAC ligand (**10**).

Next, a general method, which quantifies the ancillary ligand effects and therefore allows for the prediction of the thermodynamic stability of terminal oxo complexes, was sought. We anticipated that the singlet/triplet gap of terminal oxo compounds should be related to the stability of the complexes, because the excitation of the singlet into the triplet state is associated with transition from partial Pd=O multiple bond character to a single bond (*vide supra*).

Looking into the literature corroborates as well that thermodynamic considerations or respectively the singlet/triplet gap of a transition metal oxo complex could be a good indicator for its reactivity. It has been experimentally shown that the rate as well as the thermodynamics of CH activation by Fe^IV^ terminal oxo complexes with bispidine ligands are mainly dependent on the energy required for the spin-state crossover from *S* = 1 to *S* = 2.^128^ It was also proposed for iron(iv) model complexes that weak field ligands should lead to higher reaction rates for the activation of CH bonds.^129^,^130^ Equally, it was shown computationally for late transition metal amido and alkoxo compounds that ground state effects (*i.e.* the stability of the metal starting material) can be more important for CH activation reactivity than transition state effects.^131^,^132^ Schwarz concluded that the spin state and more precisely spin density on terminal oxyl moieties is important for an efficient hydrogen atom abstraction from hydrocarbons in the gas phase.^133^ Calculations by Ziegler suggested that thermodynamics as well as the HOMO–LUMO gap are important for the CH and OH activation reactivity of a chosen set of group 5–8 terminal oxo complexes.^134^

Indeed, the TEP, HOMO/LUMO energy levels of the ligands or partial charges of the coordinating carbene ligands (Pd, O atoms) are not very good indicators (ESI; Fig. S5–S7†) for the thermodynamic stability of the terminal oxo ligand. Contrarily, the Δ*G* value for the oxidation of carbon monoxide to carbon dioxide (Δ*G*_CO→CO_2__) is related with the singlet/triplet gap (Δ*G*_s/t_) of the LPdO complexes (Fig. 3).^135^ Supposedly weaker field ligands stabilize the triplet over the singlet state (*e.g.*, **3^PdO^**: Δ*G*_s/t_ = –15.0 kcal mol^–1^) and lead therefore to more reactive terminal oxo species. Strong field ligands like *N*-heterocyclic carbenes (**5^PdO^**: Δ*G*_s/t_ = +3.0 kcal mol^–1^) and especially the CAAC ligand (**10^PdO^** Δ*G*_s/t_ = +11.8 kcal mol^–1^) stabilize the singlet state and accordingly the terminal oxo ligand. The open-shell singlet states (o.s.s.) are predicted to not be energetically competitive with the closed-shell singlet state for most strong field ligands (Δ*G*_s/o.s.s._ > 10 kcal mol^–1^), but could become important for some of the weaker field ligands with a triplet ground state (Δ*G*_s/o.s.s._ < 10 kcal mol^–1^ for **1**, **3**, **4**; ESI, Table S5†).

**Fig. 4:**
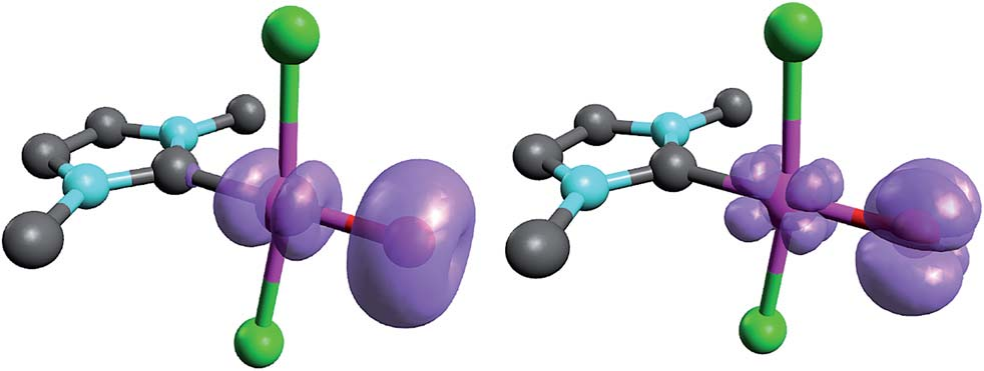
Structural parameters (C–Pd–O 180.0°, Pd–O 1.84 Å, Pd–C 2.03 Å) and spin density of NHC complex **5^PdCl_2_O^** in the triplet state (left side) and open-shell singlet state (right side).

When looking at the corresponding Pd^IV^ complexes with two chlorido ligands, the triplet state becomes more stable than the closed-shell singlet state for all investigated ligands. The geometry optimizations reveal that the chlorido ligands are positioned perpendicular to the carbene p_z_ orbital, thereby minimizing interaction between the potentially π-acidic carbene ligand and the transition metal (Fig. 4).

In addition, the elongation of the carbene palladium bond to 2.03 Å suggests negligible π-backbonding from the transition metal to the carbene ligand. Accordingly, the NHC complex **5^PdCl_2_O^** features a pseudo square planar coordination geometry with a still short Pd–O bond (1.84 Å), but a Mayer bond index of only 0.9 (Fig. 4, left side). The two unpaired electrons are localized on the oxygen and palladium atom, respectively, with only very few delocalization onto the carbene atom. This picture is complemented by the calculated partial charges on the palladium and oxygen atoms, respectively, which indicate less zwitterionic character as was predicted for the corresponding palladium(ii) complexes (Löwdin partial charges for **5^PdCl_2_O^**: O, +0.12 a.u.; Pd, –0.32 a.u.). The open-shell singlet state, which was calculated using the broken-symmetry formalism, is considerably higher in energy (Δ*G*_t/o.s.s._ = 9.8 kcal mol^–1^) and shows a similar spin density map like the triplet ground state (Fig. 4, right side). The reactivity of the palladium(iv) oxo complex **5^PdCl_2_O^** is of course higher (Δ*G*_CO→CO_2__ = –113.7 kcal mol^–1^) than the palladium(ii) oxo complex **5^PdO^** (Δ*G*_CO→CO_2__ = –104.3 kcal mol^–1^).
4

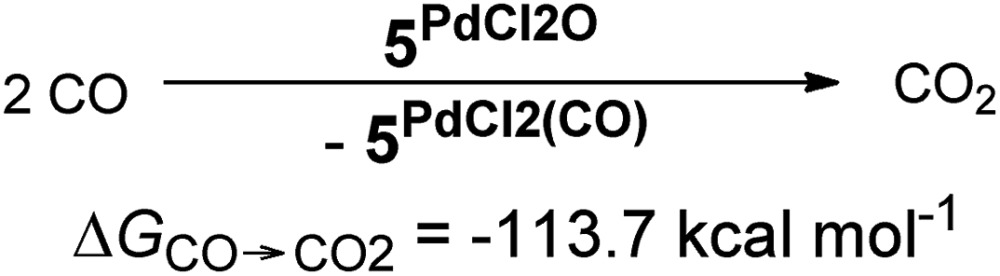




The overall oxidation strength of the palladium(iv) complexes with the carbene ligands seems to be correlated with the overall donor strength of the carbene ligand as obtained by the Tolman electronic parameter (ESI, Fig. S8†). However, the influence is small with ΔΔ*G*_CO→CO_2__ < 4 kcal mol^–1^ (**3^PdCl_2_O^**: –115.4 kcal mol^–1^; **8^PdCl_2_O^**: –112.1 kcal mol^–1^) for all carbene ligands. Note the analogy with chromium(v) nitrido complexes with weak ancillary ligands, where it has been shown that the molecular orbital energies are dominated by the {CrN}^2+^ unit.^136^,^137^

#### Bidentate ligands

**Chart 2:**
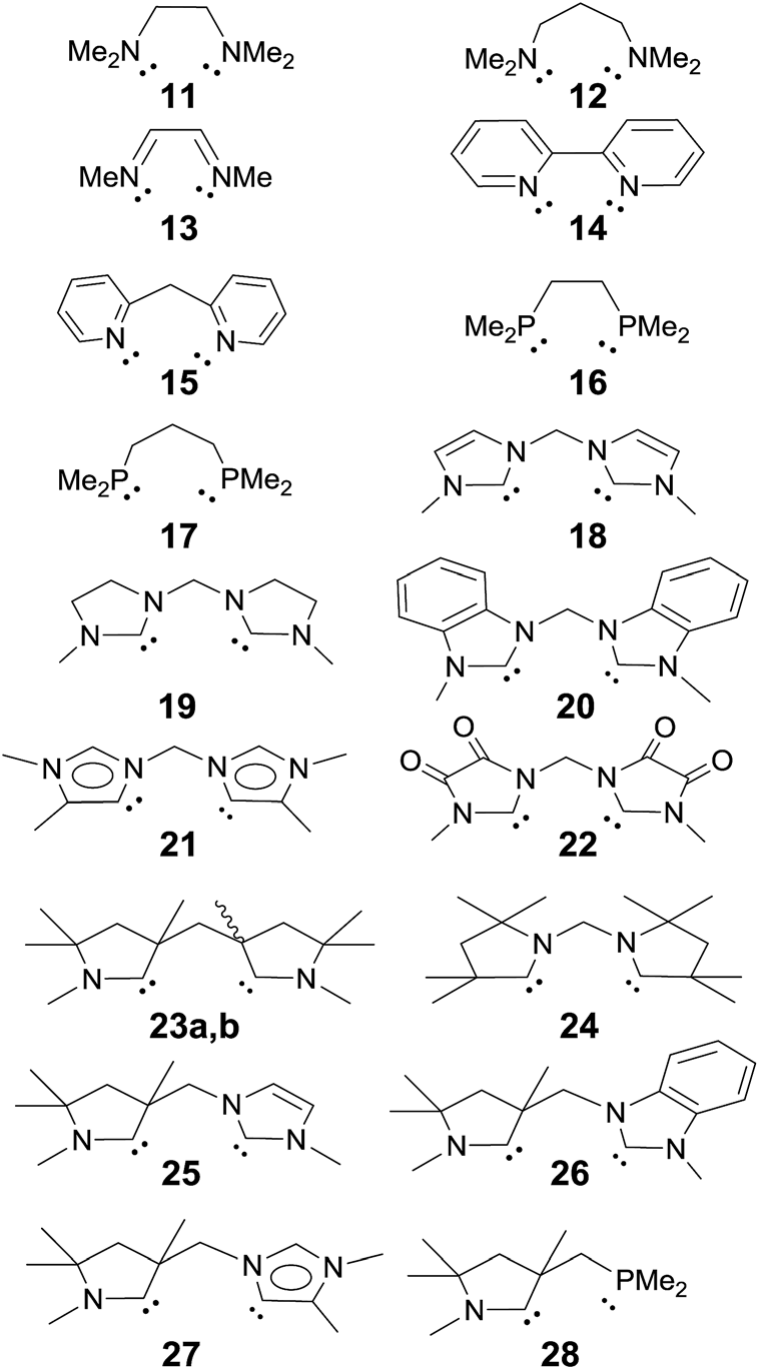
Evaluated bidentate ligands.

A series of chelating ligands was investigated in order to verify whether the correlations found for the monodentate ligands apply also for the estimation of the stability of palladium terminal oxo complexes with bidentate ligands (Chart 2).^138^,^139^

**Fig. 5:**
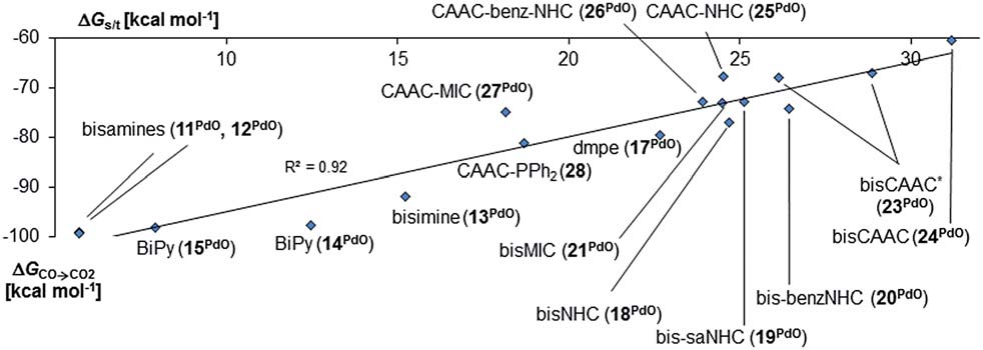
Correlation of singlet/triplet gap (Δ*G*_s/t_ = *G*_triplet_ – *G*_singlet_) of complexes with chelating ancillary ligands with Gibbs free energy for the oxidation of CO to CO_2_ (Δ*G*_CO→CO_2__) by the complexes in the singlet state.^140^

**Fig. 6:**
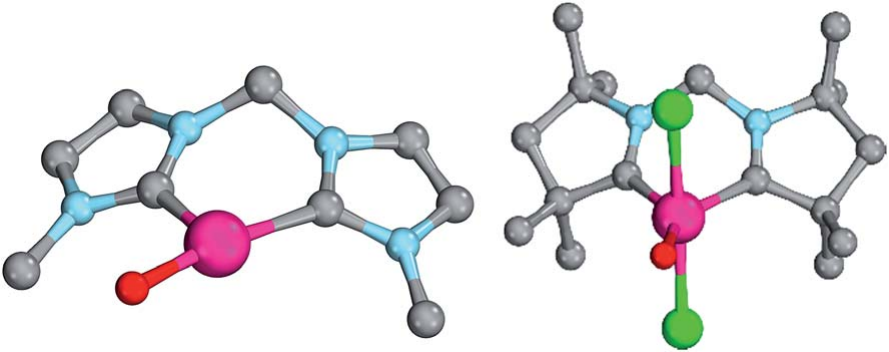
Structural parameters of **18^PdO^** (left side) and of **24^PdO^** (right side) in the singlet states. **18^PdO^**: C1–Pd–O 102.0°, Pd–O 1.83 Å, Pd–C1 1.96 Å, Pd–C2 2.04 Å, O–C 1.94 Å. **24^PdO^**: C1–Pd–O 138.0°, Pd–O 1.80 Å, Pd–C1 1.94 Å, Pd–C2 1.94 Å.

Indeed, we observed the very same trends and behavior for the bidentate ligands. Again, the molecules are expected to have a singlet ground state *e.g.*, a Δ*G*_s/t_ of more 32 kcal mol^–1^ was predicted for the CAAC complex **24** (Fig. 5). The singlet diradical electronic state was confirmed to not be important for all carbene ligands (ESI, Table S7†). The calculations predict as well a correlation between Δ*G*_s/t_ and Δ*G*_CO→CO_2__. Importantly, the CAAC complexes (**23^PdO^**, **24^PdO^**) are the most stable species, whereas the other carbene ligands and especially the N-donor ligands lead to much higher reactivity. Looking at the molecular structures of the complexes reveals in most cases similar structural parameters as were found for the monodentate ligands (Fig. 6, left side). The calculations predict T-shaped coordination geometries with a weak interaction between the *N*-methyl substituents and the terminal oxo ligand (1.94 Å). **24^PdO^** and **23a^PdO^**, which were optimized to a trigonal planar coordination geometry, are the most stable structures.

**Fig. 7:**
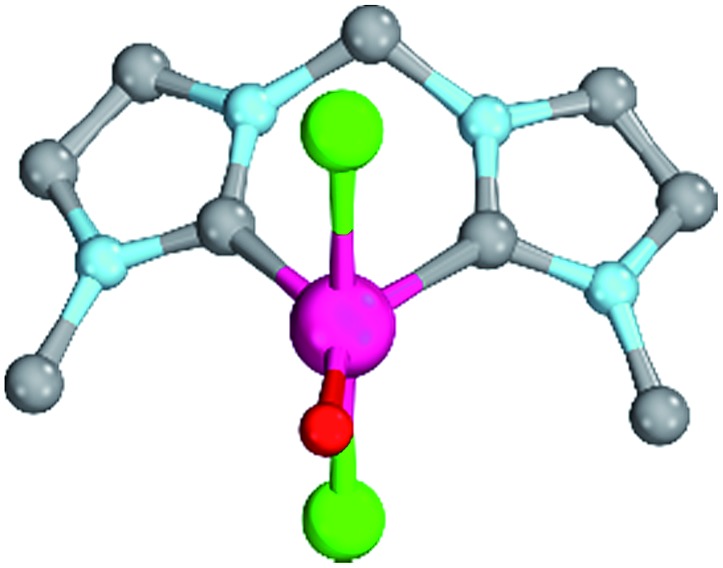
Structural parameters of **18^PdCl2O^** in the singlet state. C–Pd–O 140.0°, Pd–O 1.79 Å, Pd–C 2.05 Å.

All attempts to optimize trigonal planar coordination geometries for the other carbene ligands led to transition state structures, whereas optimizations of a T-shaped geometry for **24^PdO^** evolved to be unstable. Notably, of the two diastereomers **23a^PdO^** and **23b^PdO^**, one was optimized to a T-shaped geometry, whereas the other one features a trigonal planar coordination geometry. Looking at the bond lengths of the Pd–O bond suggests slightly reduced double bond character for the bisNHC complex **18^PdO^** (Pd–O: 1.83 Å) in relation to the bisCAAC complex **24^PdO^** (Pd–O: 1.80 Å) or the complexes with monodentate ligands (**5^PdO^**: 1.80 Å; **10^PdO^**: 1.79 Å; *vide supra*). The Mayer bond index predicts however the double bond character of the palladium oxygen bond to be slightly higher for the bidentate ligands (**18^PdO^**: 1.50; **24^PdO^**: 1.63). The electrostatic interactions between the palladium atoms and oxo ligands (Löwdin partial charges for **18^PdO^**: Pd, +/–0.00 a.u.; O, –0.64 a.u.; **24^PdO^**: Pd, +0.06 a.u.; O, –0.63 a.u.) are larger for the bidentate than the monodentate ligands (**5^PdO^**: Pd, +0.12 a.u.; O: –0.48 a.u., *vide supra*). Looking at the Pd–O bond by energy decomposition analysis (Table 3) confirms a stronger bonding interaction for the trigonal planar bisCAAC complex **24^PdO^** (*E*^int^ = –154.3 kcal mol^–1^) than for the T-shaped bisNHC complex (*E*^int^ = –140.2 kcal mol^–1^). Note that this time the steric repulsion energy (**24^PdO^**, *E*^steric^ = +61.8 kcal mol^–1^; **18^PdO^**, *E*^steric^ = +114.3 kcal mol^–1^) is – contrarily to the monodentate complexes – much smaller for the bisCAAC complex **24^PdO^**. Apparently the trigonal planar coordination geometry leads to combined reduced repulsive electrostatic and Pauli interactions, albeit at the cost of likewise reduced orbital stabilization (*E*^orb^ = –216.1 kcal mol^–1^). Looking at the isomeric bisCAAC complexes **23a^PdO^** and **23b^PdO^** supports this picture, with the trigonal planar coordination geometry showing much smaller steric repulsion energy (**23a^PdO^**, trigonal planar, *E*^steric^ = +78.5 kcal mol^–1^) than the T-shaped isomer (**23b^PdO^**, T-shaped, *E*^steric^ = +101.6 kcal mol^–1^). It was investigated, if the bidentate carbene ligands stabilize the palladium oxo species in the +IV oxidation state as well. The calculations predict the closed-shell singlet states to be more stable than the triplet states by at least Δ*G*_s/t_ >8.5 kcal mol^–1^ for all carbene ligands; note that for amine, imine, and phosphine ligands smaller singlet/triplet gaps were obtained (*vide supra*; ESI, Table S8†). The open-shell singlet states for the complexes with carbene ligands are predicted to be in most cases considerably higher in energy than the closed-shell singlet states (Δ*G* > 12 kcal mol^–1^). All carbene complexes show trigonal-bipyramidal coordination geometries with the chlorido ligands in the apical positions (Fig. 7).

**Table 3:** Energy decomposition analysis (*E*^int^ = *E*^orb^ + *E*^steric^) of the palladium – oxygen bonds of the bisCAAC complexes **24^PdO^**, **23a^PdO^**, **23b^PdO^** and the bisNHC complex **17^PdO^** in the singlet states. Values are given in kcal mol^–1^

	*E* ^int^	*E* ^orb^	*E* ^steric^
**24^PdO^** (L = bisCAAC; trigonal planar)	–154.3	–216.1	+61.8
**23a^PdO^** (L = bisCAAC, trigonal planar)	–146.7	–225.2	+78.5
**23b^PdO^** (L = bisCAAC, T-shaped)	–147.5	–249.1	+101.6
**18^PdO^** (L = bisNHC)	–140.2	–254.5	+114.3

As indicated by the Mayer bond indices and in agreement with the CASSCF(8,8) results for the bisimine ligand, the Pd–O bond shows still fairly strong double bond character (**18^PdCl_2_O^**: 1.40). Nevertheless, the zwitterionic character of the Pd–O bond is reduced through negative charge transfer from the chlorido ligands to the metal center (Löwdin partial charges for **18^PdCl_2_O^**: Pd, –0.23; O, –0.41). In fact, the Pd–O bond is modeled to be of similar length like the singlet ground state monodentate palladium(ii) complex **5^PdCl_2_O^** (1.80 Å).

**Fig. 8:**
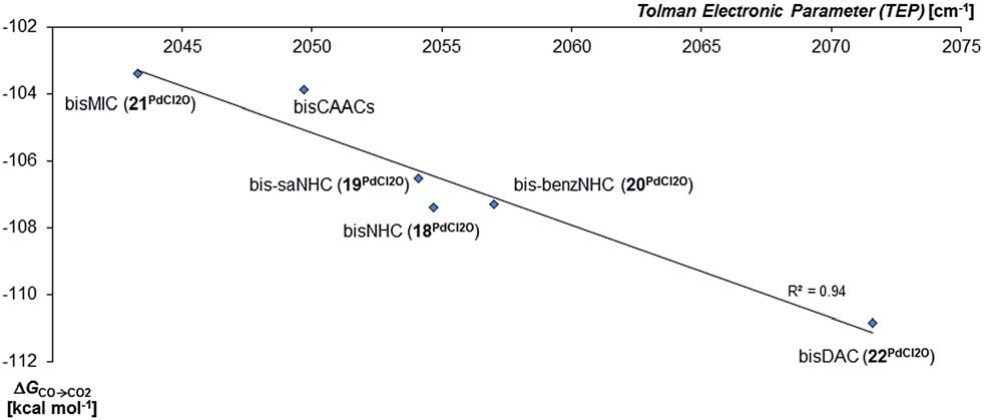
Correlation of the Gibbs free energy for the oxidation of CO to CO_2_ (Δ*G*_CO→CO_2__) by the palladium(iv) complexes with bidentate carbene ligands in the singlet state with the Tolman Electronic Parameter TEP (donicity).^141^ The values of the bisCAAC complexes **23^PdCl2O^** and **24^PdCl2O^** are averaged.

In agreement with the calculations on the triplet state of the mono coordinated palladium(iv) complexes, no correlation between Δ*G*_s/t_ and of Δ*G*_CO→CO_2__ could be found. However, the Tolman electronic parameter TEP seems to be fairly well correlated to the oxidation strength of the terminal oxo Pd^IV^ complex for the carbene ligands (Fig. 8). We conclude accordingly, that the stability of the singlet palladium(iv) terminal oxo complexes is rather dominated by the stabilization of the high-valent metal center, instead of orbital interactions with the oxo ligand. Notably, the Gibbs free enthalpy for the oxidation of the Pd^II^Cl_2_ complexes to the respective Pd^IV^OCl_2_ compounds by dimethyl dioxirane (DMDO) ranges from –2 to +9 kcal mol^–1^ (ESI, Fig. S12†). This suggests that the experimental isolation of such a complex should be possible.

#### Tridentate ligands

**Chart 3:**
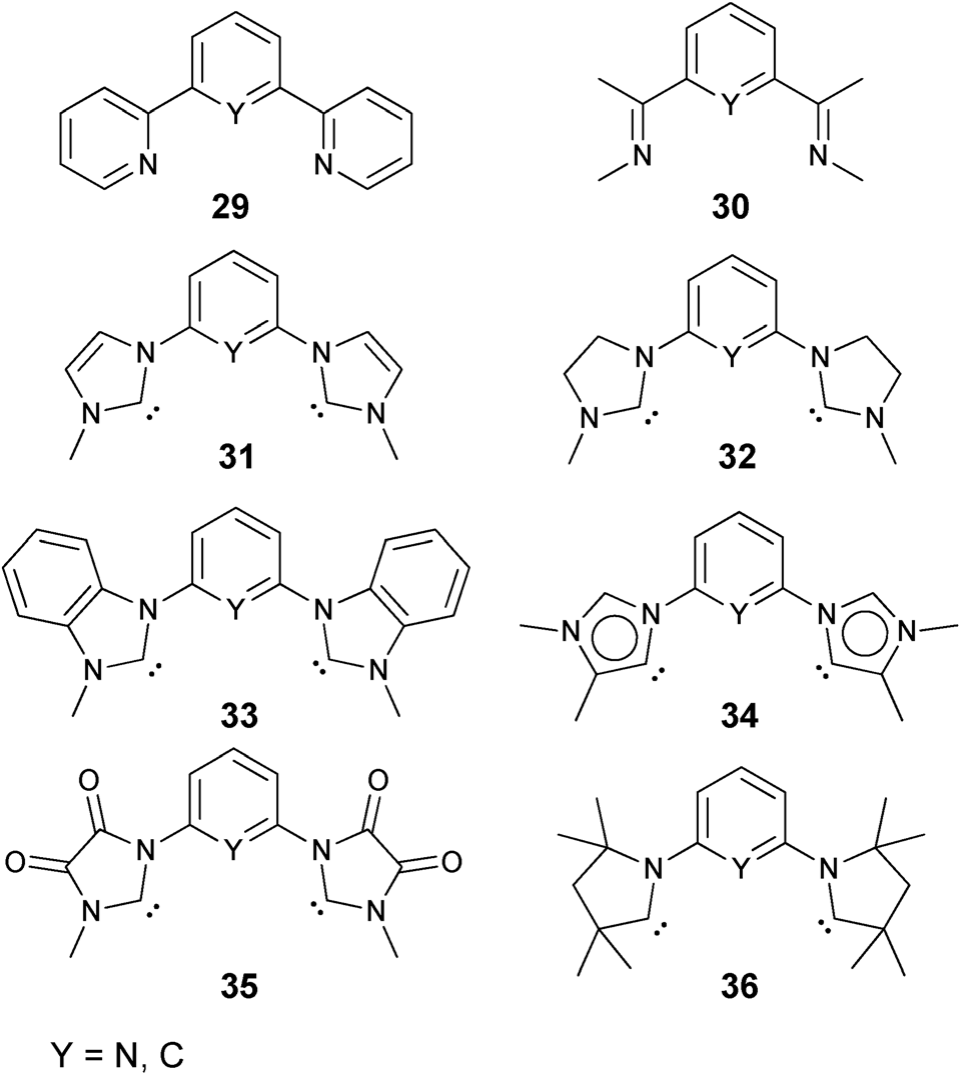
Evaluated tridentate ligands.

Finally also tridentate ligand systems were evaluated with a focus on carbene donor ligands.^142^,^143^ Pyridine bridged biscarbenes (-pyridines, -imines) were investigated for the oxidation state +II and following Milstein's experimental work^56^ phenylene bridged pincer ligands for the oxidation state +IV (Chart 3).

**Fig. 9:**
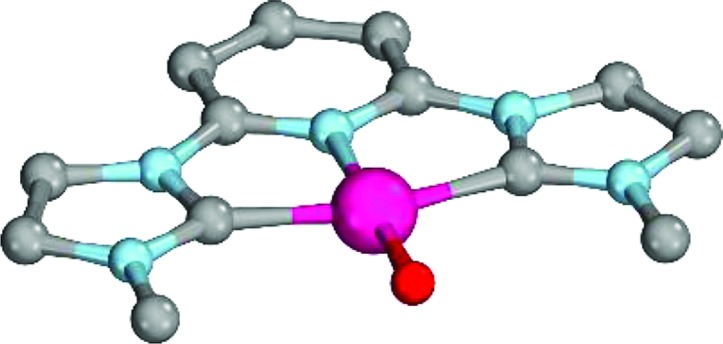
Structural parameters of **31^PdO^** in the singlet ground state. C–Pd–O 101.6°, Pd–O 1.86 Å, Pd–C 2.01 Å.

Enhanced electron donation from three L donor ligands in comparison to the bidentate coordination geometries leads to an elongation of the Pd–O bonds for the singlet-state palladium(ii) complexes (Fig. 9; **31^PdO^**: 1.86 Å) and a reduction of the Mayer bond order (1.21) in comparison to the bidentate ligand (*vide supra*). The charge separation within the Pd–O bond was found to be slightly larger (Löwdin partial charges for **31^PdO^**: O, –0.68 a.u.; Pd, –0.02 a.u.). Again, weak hydrogen bonding type interactions were found between the *N*-methyl substituents and the terminal oxo ligand.

**Fig. 10:**
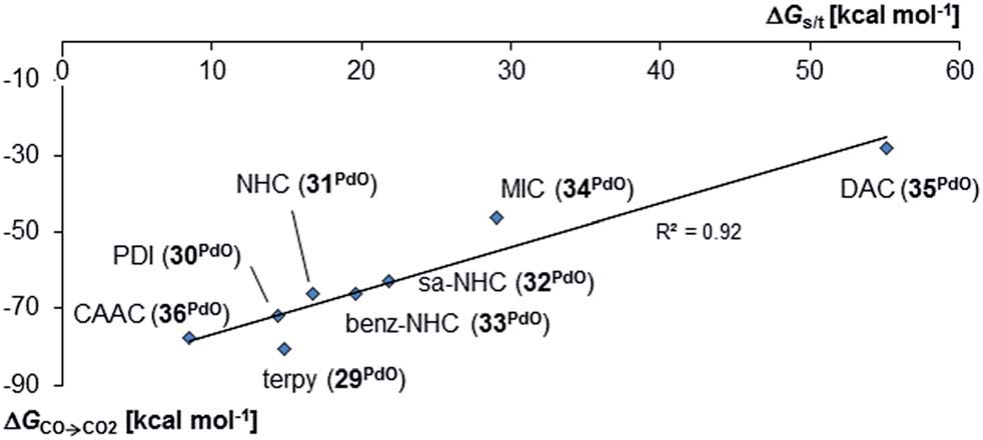
Correlation of singlet/triplet gap (Δ*G*_s/t_ = *G*_triplet_ – *G*_singlet_) of palladium(ii) complexes with tridentate ligands with Gibbs free energy for the oxidation of CO to CO_2_ (Δ*G*_CO→CO_2__) by the complexes in the singlet state.

Surprisingly, the Gibbs free energy of the oxidation of CO to CO_2_ (Δ*G*_CO→CO_2__) do not follow the same order as was obtained for the monodentate and bidentate cases. Whereas once more the stability of the palladium oxo compounds seems to be quite well correlated with the singlet/triplet gap of the complexes, the π-acidic CAAC ligand leads this time to a much more reactive compound than both the very electron rich mesoionic carbene (**34^PdO^**) or the very electron poor diamidocarbene (**35^PdO^**) ligand (Fig. 10).

**Fig. 11:**
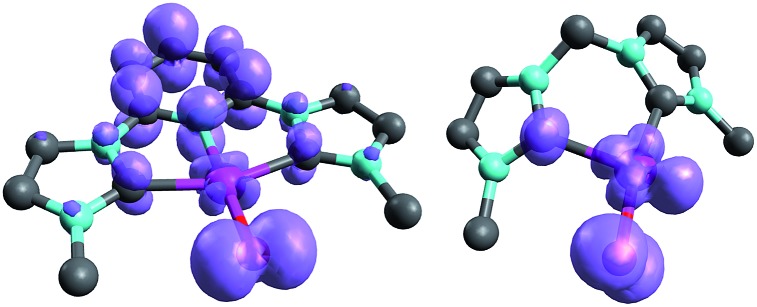
Spin densities of NHC complex **31^PdO^** (left side) and **18^PdO^** (right side) in the triplet states.

Examination of the triplet state wave functions reveals that the electronic structures of all these compounds show strong delocalization of spin density from the metal onto the pincer ligand (Fig. 11, left side).^144^,^145^ Whereas both the monodentate NHC complex **5^PdO^** (Fig. 4) and the bidentate complex **18^PdO^** (Fig. 6, left side) show localization of most spin density on the metal center with some delocalization onto the carbene atom, the complex **31^PdO^** involves a mainly pyridine centered radical with only few spin density on the metal atom (Fig. 11, left side). Therefore, simple donor–acceptor considerations are not an appropriate tool to explain reactivity trends. However, the relation shown in Fig. 10 strongly suggests that redox active ligands can be harnessed for the stabilization of terminal oxo complexes.

**Fig. 12:**
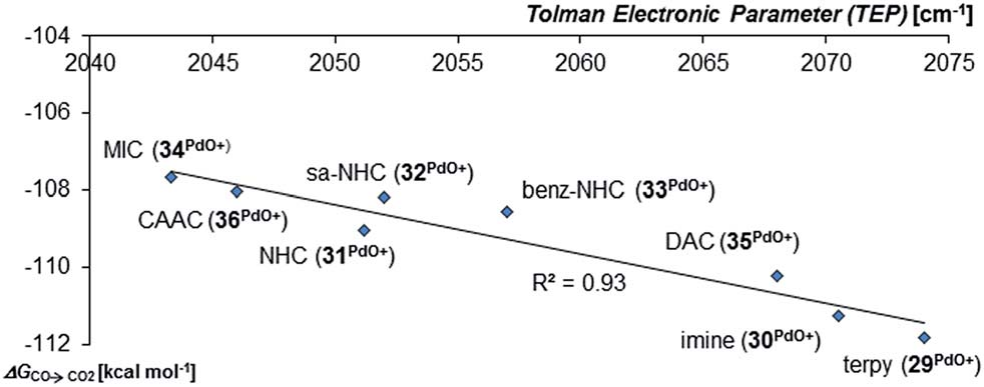
Correlation of the Tolman Electronic Parameter TEP (donicity) with Gibbs free energy for the oxidation of CO to CO_2_ (Δ*G*_CO→CO_2__) by the cationic Pd^IV^ pincer complexes in the triplet state.^48^

Looking at the pincer ligands with palladium in the oxidation state of +IV reveals that the triplet multiplicity should be more stable than the singlet state, albeit by less than 10 kcal mol^–1^ (ESI, Table S11†). The open-shell singlet biradicals are expected to be only slightly higher in energy than the triplet biradicals and in the same order of magnitude like the closed-shell singlet states (roughly 10 kcal mol^–1^ higher than the triplet states; ESI, Table S11†). The influence of the specific pincer ligand on the oxidation of CO to CO_2_ (Δ*G*_CO→CO_2__) seems to be less important than for the neutral palladium(iv) complexes with bidentate ligands (Fig. 12; *e.g.***30^PdO+^**: Δ*G*_CO→CO_2__ = –111.8 kcal mol^–1^; **34^PdO+^**: Δ*G*_CO→CO_2__ = –107.6 kcal mol^–1^).
5

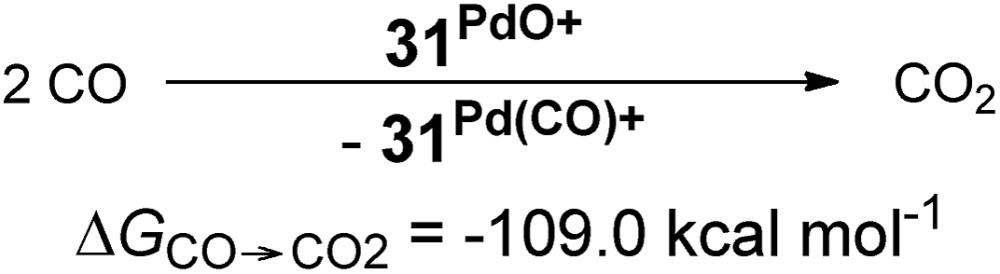




Nevertheless, there seems to be again a tentative connection between the overall donicity of the ligands, *i.e.* the Tolman electronic parameter, and the thermodynamic stability of the palladium terminal oxo complexes (Fig. 12). The correlation is in fact surprisingly good, considering that the TEP includes steric contributions and that it has been noted that values from different families of ligands (C-donor, N-donor, P-donor) can usually not be compared.

Table 4 shows a summary of the most important trends for the different evaluated ligand systems and oxidation states. As can be seen, the stability of the palladium(ii) complexes as well as the Pd–O bond length increases with the number of ancillary L type ligands. A closed shell singlet ground state was found for almost all complexes in the oxidation state of +II and the oxidation strength is correlated with the singlet/triplet gap of the terminal oxo complexes. For the palladium(iv) complexes, various electronic ground states (closed-shell singlet, open-shell singlet, triplet) are possible in dependence of the ancillary ligands. Notably, the oxidation strength of these complexes seems not to be dependent on the singlet/triplet gap of the particular complex, but rather the overall donor capabilities of ancillary ligands. Furthermore, an overall cationic charge as modeled with the L_2_X pincer ligands seems to enhance the reactivity of these complexes. We predict therefore that (transient) low-coordinate palladium(ii) and high-valent palladium(iv) terminal oxo complexes are best suited for the activation of strong bonds like *e.g.* C–H bonds. However, whereas the ancillary ligands have a huge influence on the O-atom transfer capabilities of the palladium(ii) complexes, they allow only for a fine tuning for the palladium(iv) compounds.

**Table 4:** Comparison of different ligand systems containing the imidazolylidene group

Oxidation state	Ligand	Spin state	Δ*G*_CO→CO_2__ [kcal mol^–1^]	Pd–O bond length [Å]	Descriptor
+II	L	s	–104.3	1.80	Δ*G*_s/t_
+II	L_2_	s	–77.1	1.83	Δ*G*_s/t_
+II	L_3_	s	–66.2	1.86	(Δ*G*_s/t_)^*a*^
+IV	L	t (o.s.s.)	–113.7	1.84	(TEP)^*b*^
+IV	L_2_	s (t, o.s.s.)	–106.5	1.79	TEP
+IV	L_2_X^+^	t (s, o.s.s)	–109.0	1.82	(TEP)^*c*^

^*a*^Redox non-innocence of ligands in triplet state.

^*b*^Influence small (ΔΔ*G*_CO→CO_2__ < 4 kcal mol^–1^).

^*c*^Influence small (ΔΔ*G*_CO→CO_2__ < 5 kcal mol^–1^).

**Fig. 13:**
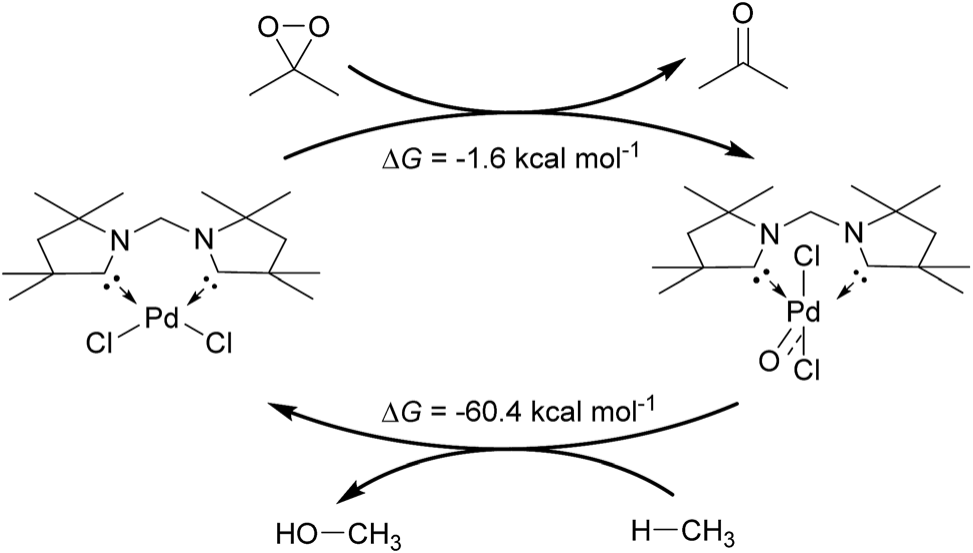
Catalytic cycle for the oxidation of methane by DMDO and **24^PdCl2^**.

Inspired by the development of new catalytic processes for the activation of strong O–H and C–H bonds, we investigated briefly the thermodynamics for the catalytic oxidation of methane by the model complex **24^PdCl_2_O^** (Fig. 13). The formation of the terminal oxo complex **24^PdCl_2_O^** is predicted to be thermodynamically feasible through oxidation of the dichloride palladium(ii) complex **24^PdCl2^** by dimethyldioxirane (DMDO; Δ*G* = –1.6 kcal mol^–1^). The oxyinsertion reaction with methane is very exergonic with Δ*G* = –60.4 kcal mol^–1^. We conclude therefore that O-atom transfer reactivity should be comparably facile for all complexes investigated herein. The oxidation step to generate the palladium terminal oxo compound appears to be potentially more challenging.

## Conclusions

Palladium terminal oxo complexes in the oxidation state of +II are predicted to show significant multiple bond character and closed-shell singlet ground states. Both the σ-donating and π-accepting properties as perfectly exemplified by ancillary cyclic (alkyl)(amino)carbene (CAAC) ligands in comparison to conventional NHC ligands are important for their thermodynamic stability. The singlet/triplet gap of the complexes, which can be controlled by the electronic properties of the ancillary ligand, is proposed to determine the reactivity of the terminal oxo group. Accordingly, potential redox activity of ancillary ligands has a remarkable influence on the electronic structure and stability.

Hydrogen bonds, which delocalize electron density from the terminal oxo ligand, enhance the stability of the basic terminal oxo complexes. Increasing the number of coordinating L-type ancillary ligands from one to three reduces the bond order, increases the dipolar character and reduces the reactivity of the Pd–O moiety. Overall, low-coordinate palladium(ii) terminal oxo intermediates are therefore especially promising targets for the design of catalytic cycles for the activation of strong bonds.

The electronic structures of the complexes in the oxidation state of +IV are more diverse. Whereas for some cases the singlet state seems to be most stable (bidentate ligands), other ligand frameworks seem to rather favor the triplet configuration (monodentate ligands). Pd–O bonds with strong single-bond character are predicted. Most salient, the stability of the palladium(iv) complexes is shown to be related to the overall donor properties of the ancillary ligands, although the influence is especially for the molecules with a triplet ground state small. The stability of these complexes can be quantified by the Tolman Electronic Parameter of the ancillary ligand. On the contrary to the palladium(ii) complexes, the π-accepting properties of ancillary ligands are not very important. Consequently, strongly donating ligands are best suited for synthetic endeavors towards the isolation of high valent palladium(iv) terminal oxo compounds. Accordingly, it also appears very plausible why Milstein's platinum(iv) terminal oxo complex did not feature a ligand with considerable π-acceptor capabilities. Of particular interest, palladium(iv) terminal oxos appear to be generally quite reactive and suitable candidates for the activation of very strong bonds.

In sight of those predictions, we believe that the synthetic isolation of palladium terminal oxo compounds is feasible with ancillary ligand systems providing balanced electronic stabilization and kinetic protection through a sterically encumbering ligand system. Consequently, we hope that such exciting compounds will become available for the scientific community in the future and will allow for the development of novel catalytic oxidation protocols.

## Conflicts of interest

There are no conflicts to declare.

## Supplementary Material

Supplementary information
