# Supplementary material for: How to tame a palladium terminal oxo
Source: Chem Sci. 2017 Dec 13;9(5):1155–67. doi: 10.1039/c7sc05034h (PMC5883948; doi:10.1039/c7sc05034h)
Supplement: Supplementary file 1 [file SC-009-C7SC05034H-s001.pdf]

Electronic Supplementary Material (ESI) for Chemical Science.
This journal is © The Royal Society of Chemistry 2017

How to Tame a Palladium Terminal Oxo

*Dominik Munz**

Friedrich-Alexander Universität Erlangen-Nürnberg, Egerlandstr. 1, 91058 Erlangen, Germany

E-mail address and telephone number:

dominik.munz@fau.de, +49 9131 85 27464

Content:

1.	Benchmarking of Functional, Basis Set and Solvent Effects	S2
2.	Molecular Orbital Diagrams	S4
3.	Natural Resonance Theory Analysis	S6
4.	Correlations TEP/Singlet-triplet Gaps/Partial Charges	S7
5.	Selection of Active Space	S10
6.	Energies	S12
7.	XYZ Coordinates of CASSCF(8,8) Optimized Structures	S20
8.	XYZ Coordinates of B2PLYP-D3 Optimized Structures	S22
9.	XYZ Coordinates and TEP of Optimized Carbene Structures	S23
10.	XYZ Coordinates of B3LYP Optimized Structures	S24
11.	References	S84
12.	Full Gaussian Citation	S84

1. Benchmarking of Functional, Basis Set and Solvent Effects

I. Palladium(IV) Bisimine Complex with Hydrogen Substituents

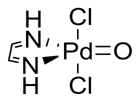

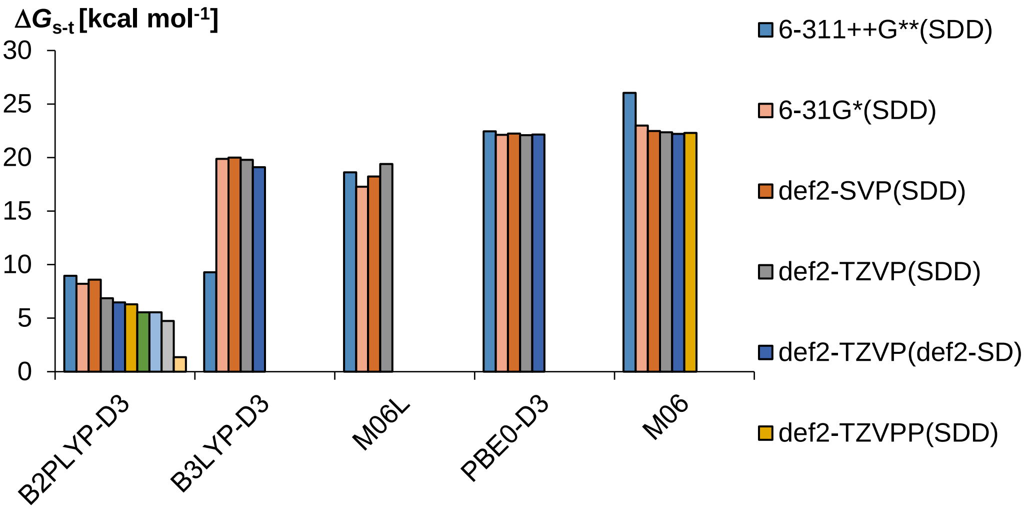

Figure S1. Dependence of singlet-triplet gap ($\Delta G_{s/t}$) of palladium(IV) oxo complex with bisimine ligand (truncated with H substituents) from basis set and computational method.

II. Palladium(IV)/Platinum(IV) Complex with Milstein's[1] Ligand

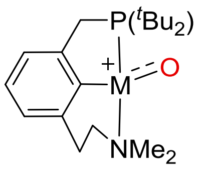

M = Pd, Pt

**Method**	**$\Delta G_{s/t}$ (M = Pd)**	**$\Delta G_{s/t}$ (M = Pt)**
B3LYP-D3/def2-TZVP	-16.6 kcal mol^{-1}	-12.5 kcal mol^{-1}
PWPB95D3/def2-TZVPP	-7.4 kcal mol^{-1}	n.d.
PWPB95(COSMO)/def2-TZVP	-7.0 kcal mol^{-1}	n.d.
PWPB95D3(COSMO)/def2-TZVP	-7.4 kcal mol^{-1}	n.d.
PWPB95D3(COSMO)/def2-TZVPP	-7.1 kcal mol^{-1}	n.d.
B2PLYP-D3/def2-TZVP	-6.2 kcal mol^{-1}	n.d.
B2PLYP-D3(COSMO)/def2-TZVP	-5.7 kcal mol^{-1}	n.d.

B2PLYP-D3(COSMO)/def2-TZVPP	-5.8 kcal mol^{-1}	-4.1 kcal mol^{-1}
B2GP-BLYP-D3(COSMO)/def2-TZVPP	-3.3 kcal mol^{-1}	-2.0 kcal mol^{-1}
B2GP-BLYP/spd/vdz according to [1b]	n.d.	-2.8 kcal mol^{-1}

Table S1. Gibbs free energy for oxidation of palladium(II) and platinum(II) complex with Milstein's ligand[1] by dimethyldioxirane with different computational methods.

III. Oxidation of bis-NHC Complex

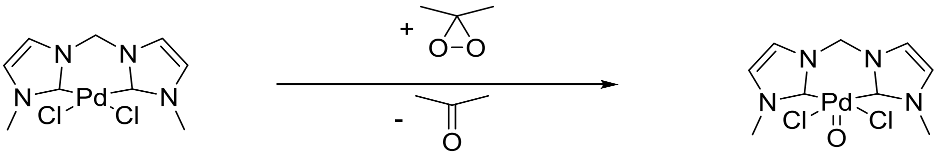

Method	Δ*G*
B2PLYP-D3/def2-TZVPP (SDD)	+22.9 kcal mol^{-1}
B3LYP/def2-TZVPP (LANL ECP)	+32.6 kcal mol^{-1}
B3LYP/def2-TZVPP (SDD)	+25.5 kcal mol^{-1}
B3LYP-D3/def2-TZVPP (SDD)	+20.1 kcal mol^{-1}
PBE0-D3/def2-TZVPP (SDD)	+33.2 kcal mol^{-1}
M06/def2-TZVPP (SDD)	+30.6 kcal mol^{-1}
M06-L/def2-TZVPP (SDD)	+23.7 kcal mol^{-1}

Table S2. Gibbs free energy for oxidation of bis-NHC palladium(II) complex by dimethyldioxirane with different computational methods.

2. <u>Molecular Orbital Diagrams</u>

Palladium oxide (PdO) is predicted to have a triplet ground state [$^3\Sigma(\pi^{*3}\ \sigma^{*1})$ or $^3\Sigma^-(\pi^{*2}\ \sigma^{*2})$, which have very similar energies].[2] The closed-shell singlet state [$^1\Sigma^+(\pi^{*4})$] is predicted to be higher in energy. The closed-shell singlet–triplet gap on the B2PLYP-D3/def2-TZVPP level of theory is $\Delta E_{s/t}$ = -27.6 kcal mol^{-1}. However, because the (CAAC)PdO and (NHC)PdO molecules were predicted to have a closed-shell ground state, also the closed-shell electronic configuration of PdO, i.e. [$^1\Sigma^+(\pi^{*4})$], was used for the construction of the following fragment orbital interaction diagram.

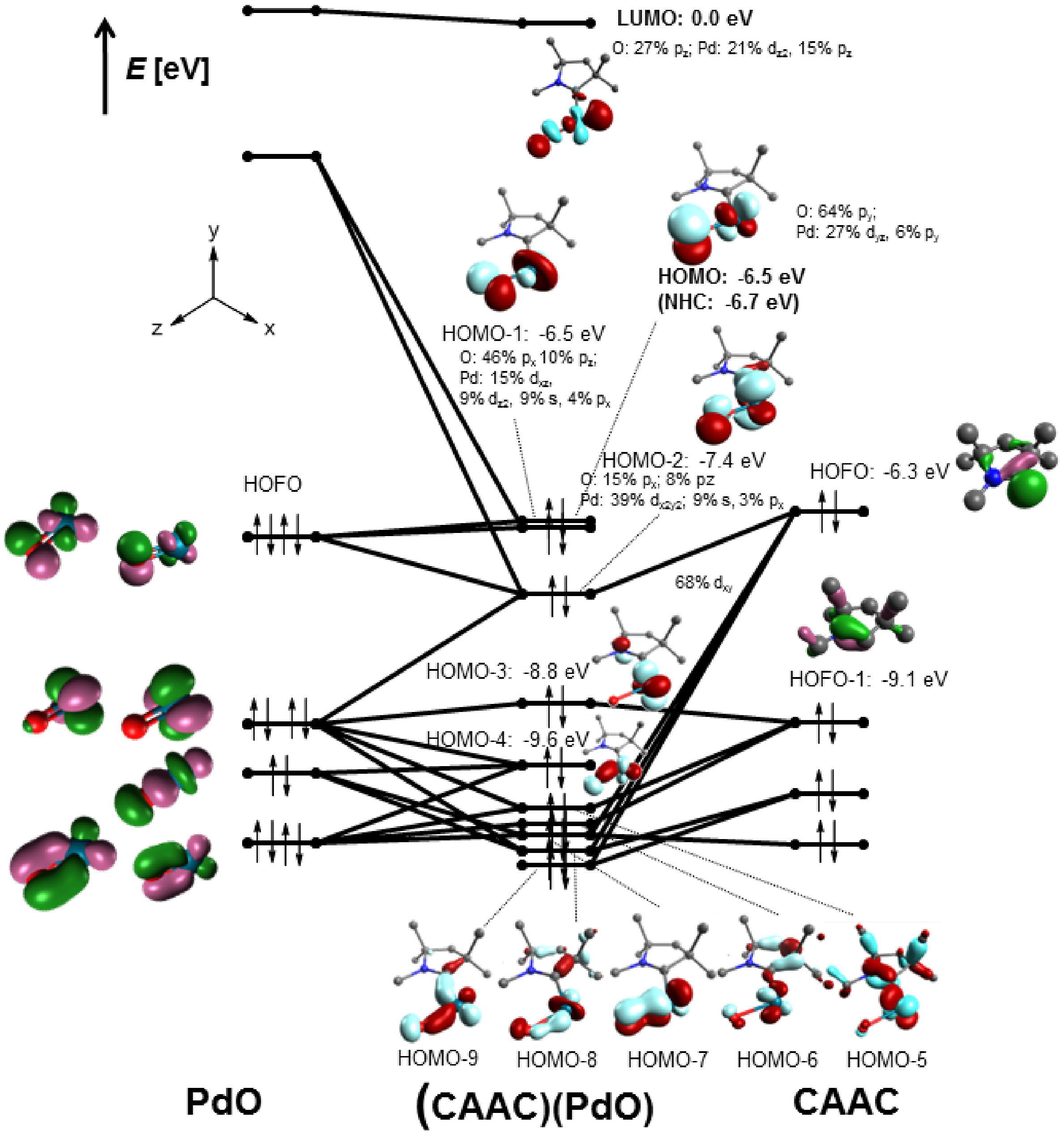

Figure S2. Fragment orbital diagram for **10^{PdO}**. Fragments are PdO in the closed-shell singlet state [$^1\Sigma^+(\pi^{*4})$][2] and the free CAAC ligand. Hydrogen atoms are omitted for clarity. Fragment orbital interactions are shown with a threshold of 8% for each corresponding fragment and main atomic orbital contributions in % are derived from

the reduced Löwdin orbital population analysis. Energies of molecular orbitals of the PdO fragment are shifted by +1.0 eV.

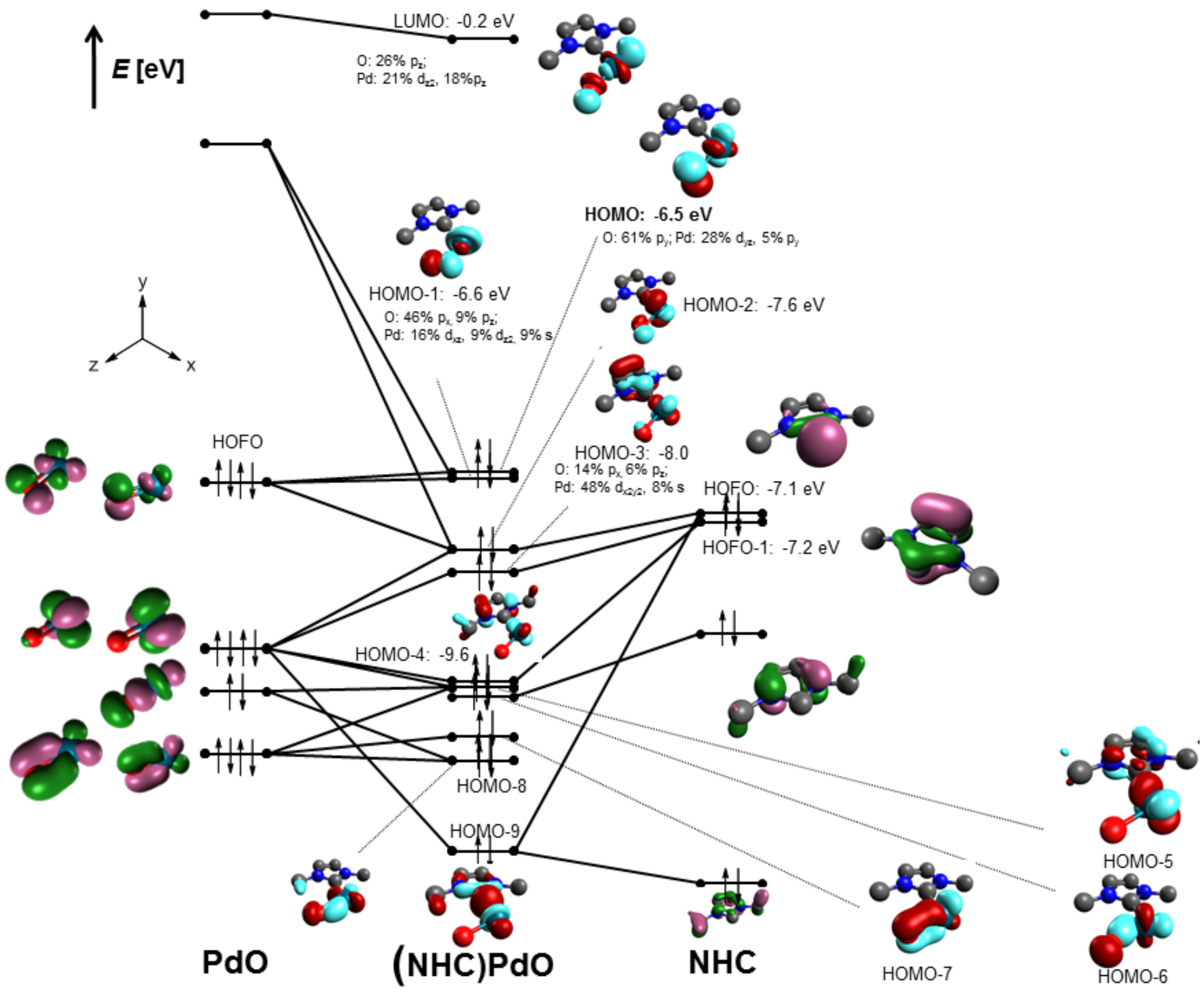

Figure S3. Fragment orbital diagram for $\mathbf{5^{PdO}}$. Fragments are PdO in the closed-shell singlet state $[^1\Sigma^+(\pi^{*4})]^2$ and the free CAAC ligand. Hydrogen atoms are omitted for clarity. Fragment orbital interactions are shown with a threshold of 8% for each corresponding fragment. Energies of molecular orbitals of the PdO fragment are shifted by +1.0 eV.

Table S3. Energy decomposition analysis ($E^{int} = E^{orb} + E^{steric}$) of (CAAC)–(PdO) ($\mathbf{10^{PdO}}$) and (NHC)–(PdO) ($\mathbf{5^{PdO}}$). Fragments are PdO in the closed-shell singlet state $[^1\Sigma^+(\pi^{*4})]2$ and the free carbene ligands. Values are given in kcal mol^{-1}.

Fragments	Complex	E^{int}	E^{orb}	E^{steric}
**(OPd)–(CAAC)**	$\mathbf{10^{PdO}}$ (L = CAAC)	-65.8	-335.6	-269.8
**(OPd)–(NHC)**	$\mathbf{5^{PdO}}$ (L = NHC)	-59.6	-100.6	+41.0

3. Natural Resonance Theory Analysis

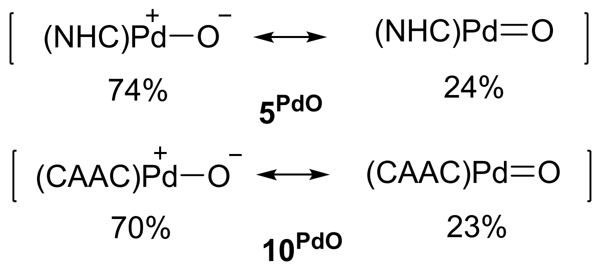

Figure S4. Double bond character of Pd=O bond as predicted by NRT analysis using NBO 6.0.

4. Correlations TEP/Singlet-triplet Gaps/Partial Charges/

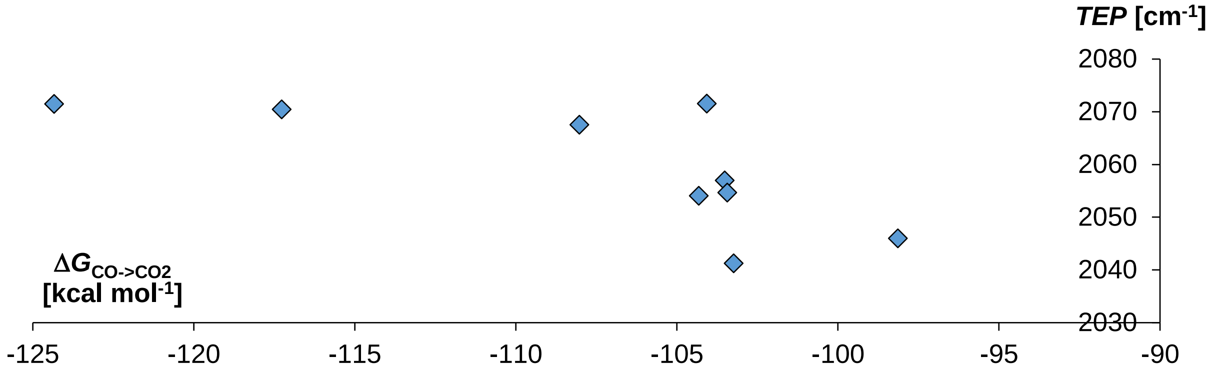

Figure S5. The oxo transfer from palladium(II) terminal oxo complexes with monodentate ligands to carbon monoxide ($\Delta G_{CO\text{->}CO2}$) appears to not be correlated with the Tolman Electronic Parameter *TEP*.

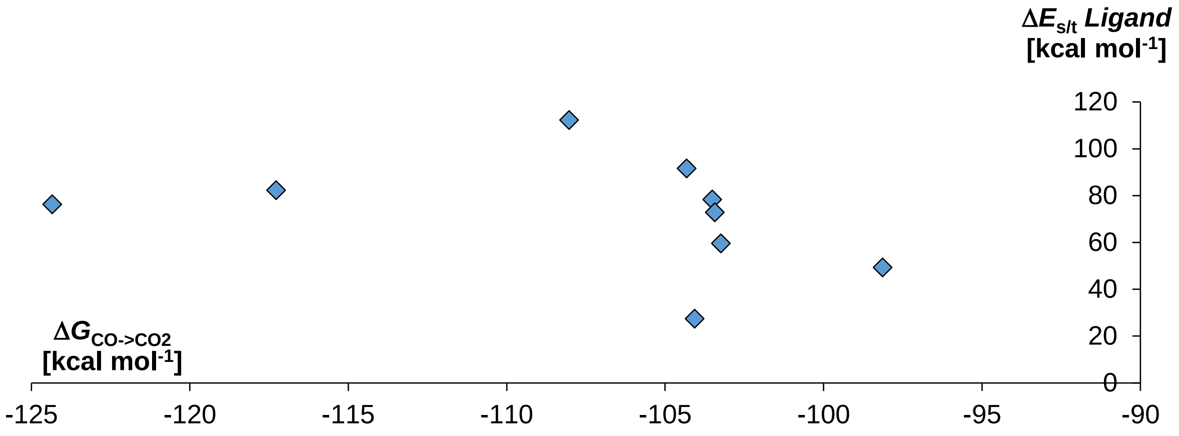

Figure S6. The oxo transfer from palladium(II) terminal oxo complexes with monodentate ligands to carbon monoxide ($\Delta G_{CO\text{->}CO2}$) appears to not be correlated with the singlet-triplet gap of the ligands ($\Delta E_{s/t}$) as obtained from ref. [3].

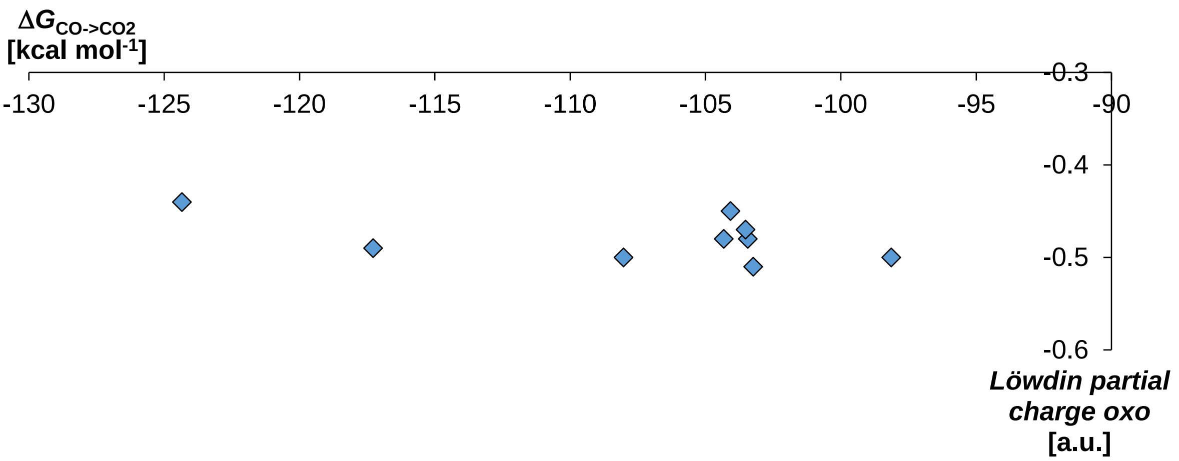

Figure S7. The oxo transfer from palladium(II) terminal oxo complexes with monodentate ligands to carbon monoxide ($\Delta G_{CO\text{->}CO2}$) appears to not be correlated with the Löwdin partial charges of the therminal oxo group.

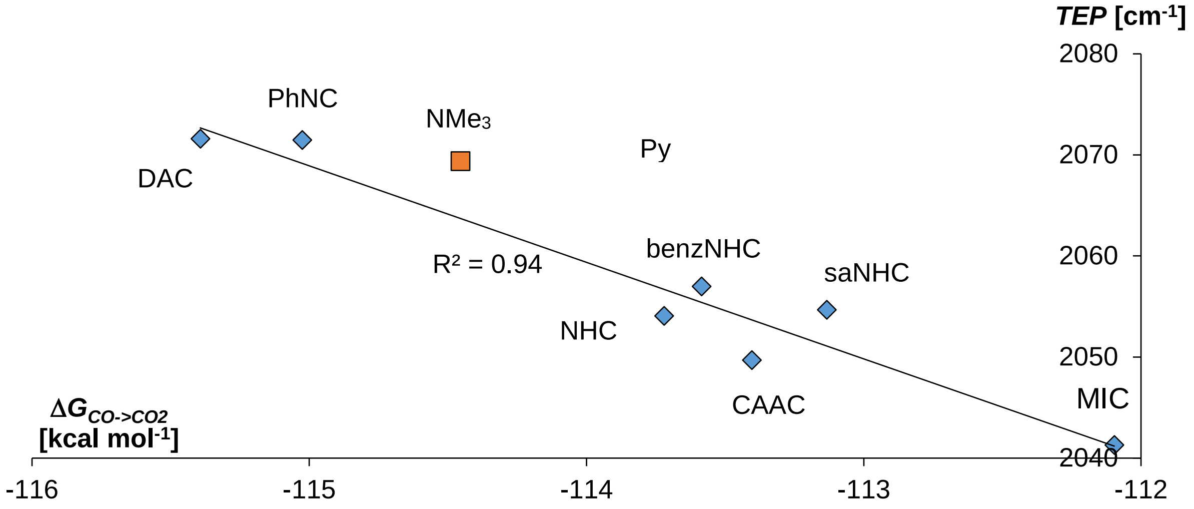

Figure S8. Relation between oxo transfer from palladium(IV) terminal oxo complexes with monodentate ligands to carbon monoxide ($\Delta G_{CO\text{->}CO2}$) and Tolman Electronic Parameter *TEP*. *N*-donor ligands are omitted from regression; palladium(IV) complexes with phosphine ligands are not stable regarding the formation of phosphine oxides.

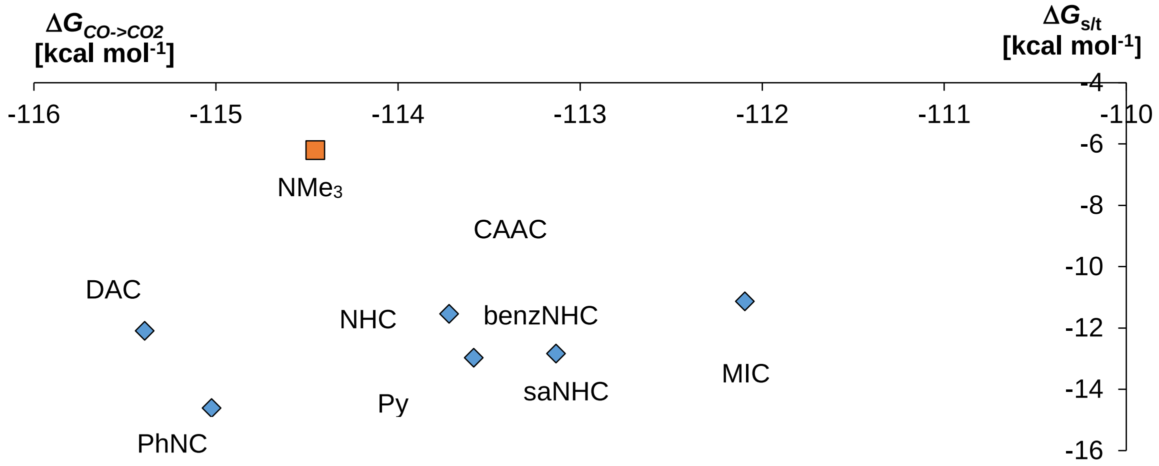

Figure S9. The oxo transfer from from palladium(IV) terminal oxo complexes with monodentate ligands to carbon monoxide ($\Delta G_{CO\text{->}CO2}$) appears to not be strongly correlated with the singlet-triplet gap ($\Delta G_{s/t}$) of the complexes.

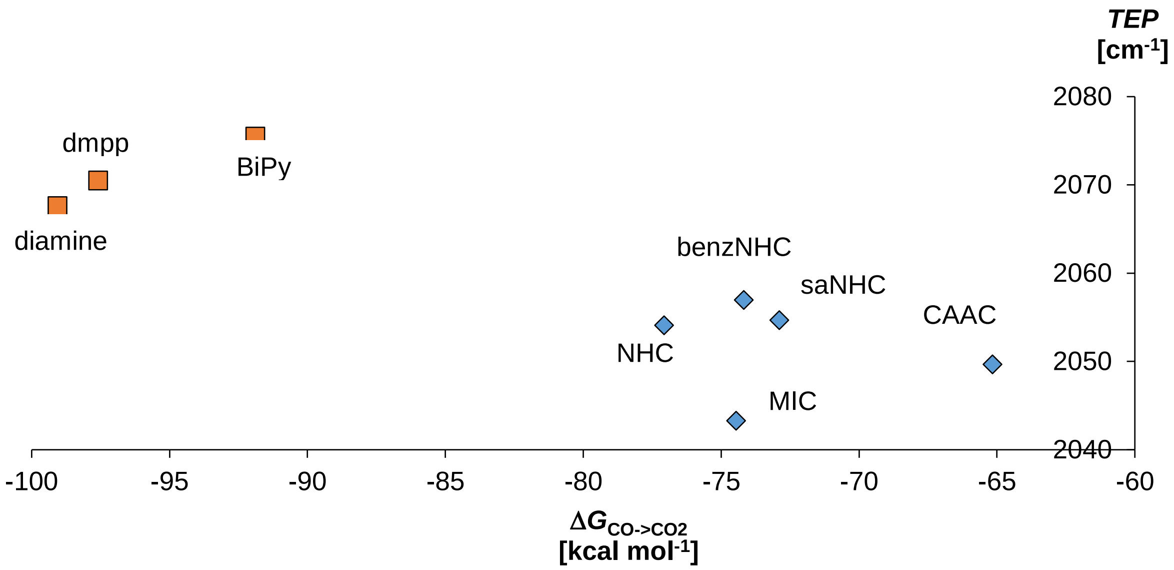

Figure S10. The oxo transfer from palladium(II) terminal oxo complexes with bidentate ligands to carbon monoxide ($\Delta G_{CO\text{->}CO2}$) appears to not be strongly correlated with the Tolman Electronic Parameter *TEP*. The DAC is omitted due to instability of the CO complex.

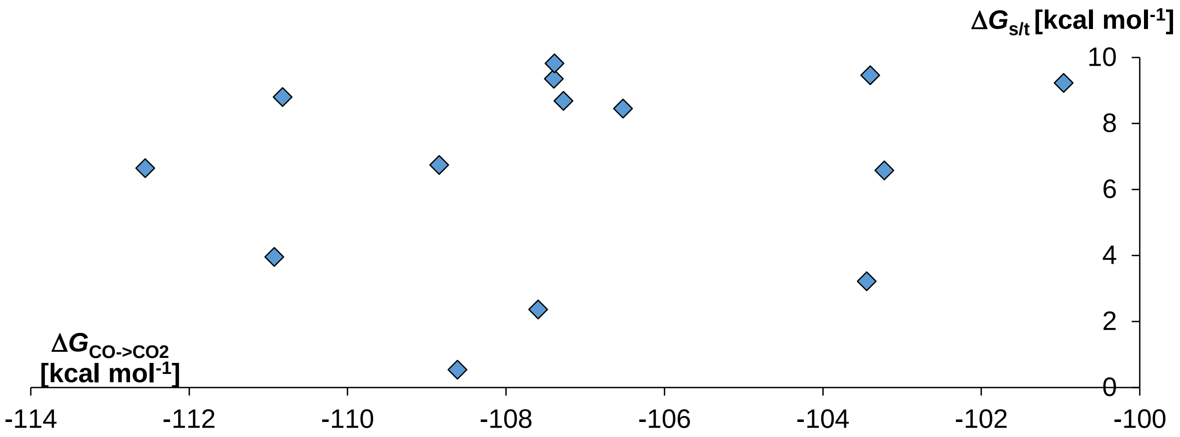

Figure S11. The oxo transfer from palladium(IV) terminal oxo complexes with bidentate ligands to carbon monoxide ($\Delta G_{CO\text{->}CO2}$) is not correlated with the singlet-triplet gap of the complexes ($\Delta G_{s/t}$).

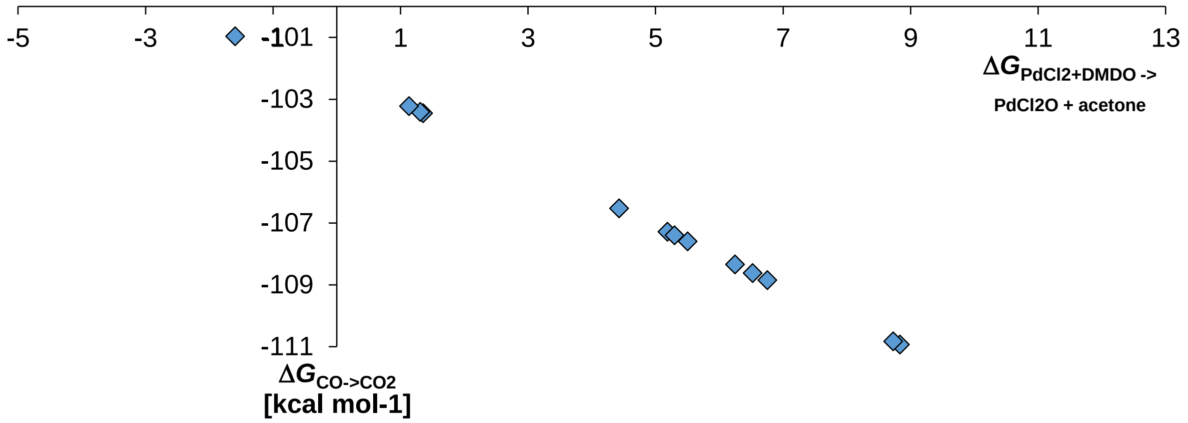

Figure S12. Comparison of the Gibbs free energy of oxidation of palladium(II)Cl_2 complexes with bidentate ligand by dimethyldioxirane (DMDO) to palladium(IV) terminal oxo complexes and acetone ($\Delta G_{PdCl2+DMDO\text{->}PdCl2O+acetone}$) with the oxygen transfer from the respective palladium(IV) terminal oxo complexes to carbon monoxide ($\Delta G_{CO\text{->}CO2}$).

5. Selection of Active Space

A selection of a larger active space, which included all palladium d-orbitals, did not lead to an improvement of the results, but instead to very sluggish SCF convergence with molecular orbital occupation numbers very close to 2 and 0. The calculation and optimization of the state averaged (multiplicities: 3,1; Roots: 1,2) CASSCF(8,8) wavefunction led as well to a closed shell singlet ground state with the excited open-shell singlet state higher in energy than the triplet excited state.

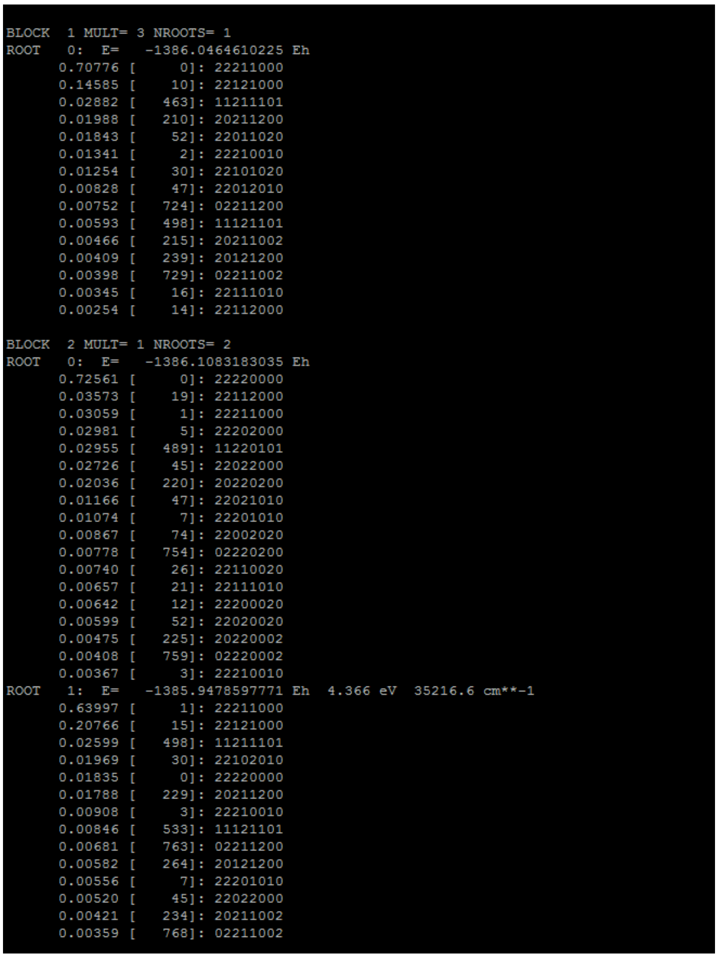

```
BLOCK  1 MULT= 3 NROOTS= 1
ROOT   0:  E=   -1386.0464610225 Eh
      0.70776 [     0]: 22211000
      0.14585 [    10]: 22121000
      0.02882 [   463]: 11211101
      0.01988 [   210]: 20211200
      0.01843 [    52]: 22011020
      0.01341 [     2]: 22210010
      0.01254 [    30]: 22101020
      0.00828 [    47]: 22012010
      0.00752 [   724]: 02211200
      0.00593 [   498]: 11121101
      0.00466 [   215]: 20211002
      0.00409 [   239]: 20121200
      0.00398 [   729]: 02211002
      0.00345 [    16]: 22111010
      0.00254 [    14]: 22112000

BLOCK  2 MULT= 1 NROOTS= 2
ROOT   0:  E=   -1386.1083183035 Eh
      0.72561 [     0]: 22220000
      0.03573 [    19]: 22112000
      0.03059 [     1]: 22211000
      0.02981 [     5]: 22202000
      0.02955 [   489]: 11220101
      0.02726 [    45]: 22022000
      0.02036 [   220]: 20220200
      0.01166 [    47]: 22021010
      0.01074 [     7]: 22201010
      0.00867 [    74]: 22002020
      0.00778 [   754]: 02220200
      0.00740 [    26]: 22110020
      0.00657 [    21]: 22111010
      0.00642 [    12]: 22200020
      0.00599 [    52]: 22020020
      0.00475 [   225]: 20220002
      0.00408 [   759]: 02220002
      0.00367 [     3]: 22210010
ROOT   1:  E=   -1385.9478597771 Eh  4.366 eV  35216.6 cm**-1
      0.63997 [     1]: 22211000
      0.20766 [    15]: 22121000
      0.02599 [   498]: 11211101
      0.01969 [    30]: 22102010
      0.01835 [     0]: 22220000
      0.01788 [   229]: 20211200
      0.00908 [     3]: 22210010
      0.00846 [   533]: 11121101
      0.00681 [   763]: 02211200
      0.00582 [   264]: 20121200
      0.00556 [     7]: 22201010
      0.00520 [    45]: 22022000
      0.00421 [   234]: 20211002
      0.00359 [   768]: 02211002
```

Figure S13. Results of state-averaged CAS(8,8) calculation.

Active Space Triplet Multiplicity:

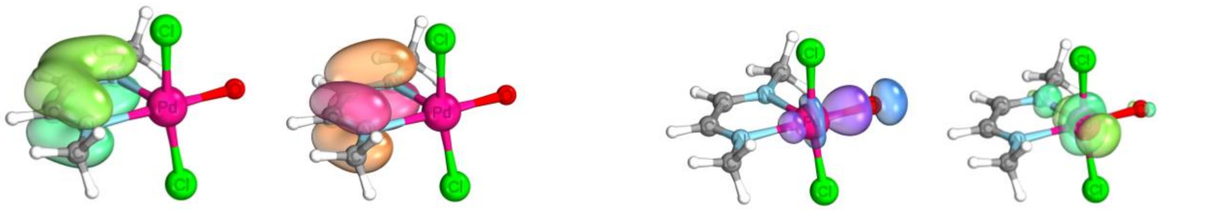

MO 49 (NO 1.93)

MO 50 (NO: 1.90)

MO 51 (NO: 1.87)

MO 52 (NO: 1.00)

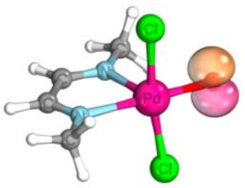

MO 53

(NO: 1.00)

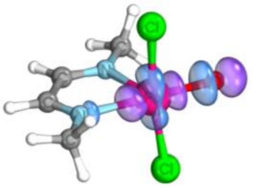

MO 54

(NO: 0.13)

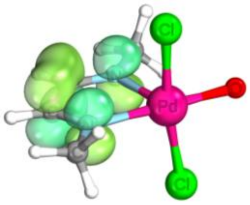

MO 55

(NO: 0.13)

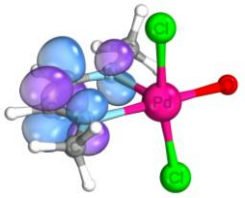

MO 56

(NO: 0.06)

Active Space Open-Shell Singlet Multiplicity:

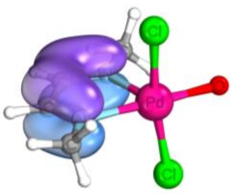

MO 49

(NO: 1.93)

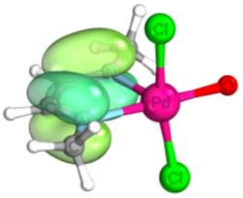

MO 50

(NO: 1.90)

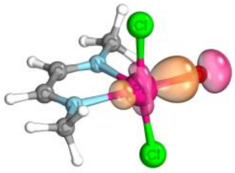

MO 51

(NO: 1.84)

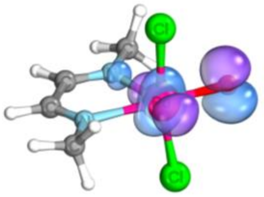

MO 52

(NO: 1.02)

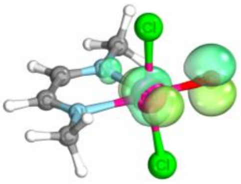

MO 53

(NO: 0.98)

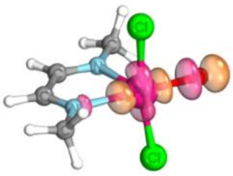

MO 54

(NO: 0.16)

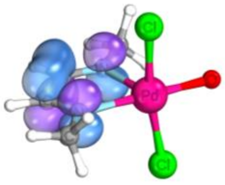

MO 55

(NO: 0.11)

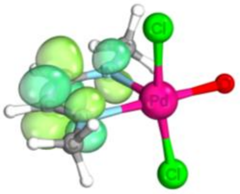

MO 56

(NO: 0.06)

Active Space Singlet Multiplicity:

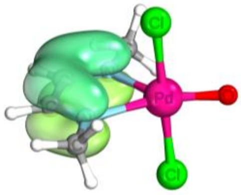

MO 49

(NO: 1.93)

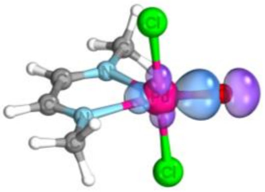

MO 50

(NO: 1.90)

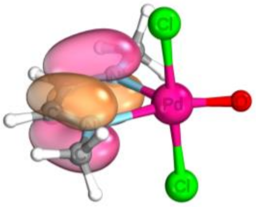

MO 51

(NO: 1.90)

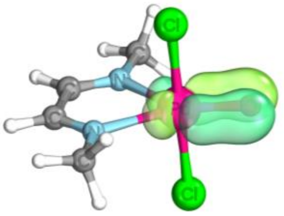

MO 52

(NO: 1.78)

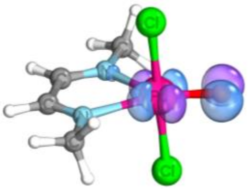

MO 53

(NO: 0.22)

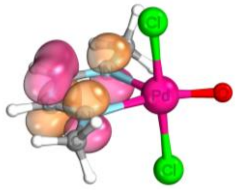

MO 54

(NO: 0.11)

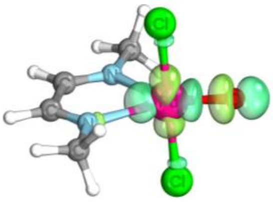

MO 55

(NO: 0.10)

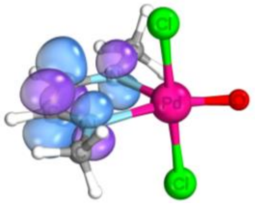

MO 56

(NO: 0.06)

6. <u>Energies</u>

	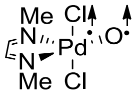<br>**t**	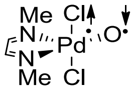<br>**o.s.s.**	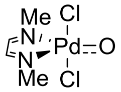<br>**s**
E [CASSCF(8,8)/def2-SVP]	-1385.38073	-1385.38392	-1385.37388
E [CASSCF(8,8)/def2-TZVPP]	-1386.119159	-1386.120440	-1386.124042
G [CASSCF(8,8)/def2-TZVPP]	-1386.017440	-1386.019952	-1386.020527
E [CASSCF(8,8)/NEVPT2]	-1388.791795	-1388.786484	-1388.794312
E [B2PLYP-D3/def2-TZVPP]	-1389.930273	-1389.921738	-1389.932810
G [B2PLYP-D3/def2-TZVPP]	-1389.845205	-1389.835225	-1389.844433
E [B2PLYP-D3//B3LYP]	-1389.929125	-1389.916723	-1389.932063
E [B3LYP-D3/def2-TZVP]	-1390.5093295	n.a.	-1390.4873797
E [DLPNO-CCSD(T)//B3LYP]	-1388.4969299	n.a.	-1388.4893816
G [B3LYP-D3/def2-TZVP]	-1390.427262	n.a.	-1390.401592

Table S4. Electronic energies (*E*) and Gibbs free energies (*G*) of CAS(8,8) and B2PLYP-D3 optimized palladium(IV) complexes with bisimine ligand (**13**^{PdCl2O}) and comparison with B3LYP results.

Name	Denominator	*E* (B3LYP)	*E* (B2PLYP)	*G* (B3LYP)
PhI	-	-529.559027	-529.148005	-529.500865
PhCl2	-	-1449.961250	-1449.353156	-1449.910720
Cl2	-	-920.424047	-920.228137	-920.444633
DMDO	-	-268.392923	-268.207912	-268.333424
acetone	-	-193.248157	-193.101432	-193.193234
CO	-	-113.363254	-113.299809	-113.377326
CO2	-	-188.671580	-188.568849	-188.681218
CH4	-	-40.5394383	-40.4936185	-40.514494
CH3OH	-	-115.77878	-115.697601	-115.750507

Table S5. Electronic energies (*E*) and Gibbs free energies (*G*) of small molecules.

Name	Denominator	*E* (B3LYP)	*E* (B2PLYP)	*E* (B2PLYP) o.s.s./UKS	*G* (B3LYP)
Pyridine_CO	1PdCO	-489.8022733	-489.4276804		-489.740608
pyridine_oxo_s	1PdO_s	-451.5326627	-451.1871626		-451.47534
pyridine_oxo_t	1PdO_t	-451.5580643	-451.1964078	-451.190486	-451.500909
NMe3_CO	2PdCO	-415.9632453	-415.625303		-415.868813
NMe3_oxo_s	2PdO_s	-377.6948439	-377.4002452		-377.604031
NMe3_oxo_t	2PdO_t	-377.7257734	-377.3936577	-377.3872504	-377.636334

Isonitrile_CO	3PdCO	-566.0223879	-565.5911217		-565.956363
Isonitrile_oxo_s	3PdO_s	-527.7446903	-527.3431278		-527.679228
Isonitrile_oxo_t	3PdO_t	-527.7826585	-527.3644993	-527.340677	-527.71984
PMe3_CO	4PdCO	-702.6183368	-702.2170572		-702.536301
PMe3_s	4PdO_s	-664.3371291	-663.9669457		-664.256281
PMe3_t	4PdO_t	-664.3780314	-663.9830759	-663.9767196	-664.301588
NHC_CO	5PdCO	-546.3728498	-545.9535927		-546.276714
NHC_oxo_s	5PdO_s	-508.123042	-507.7346085		-508.030357
NHC_oxo_t	5PdO_t	-508.1310926	-507.7275105	-507.7193146	-508.040674
NHCS_CO	6PdCO	-547.5780721	-547.1478368		-547.459939
NHCS_oxo_s	6PdO_s	-509.3306847	-508.9319624		-509.214311
NHCS_oxo_t	6PdO_t	-509.3410437	-508.9192657	-508.9143532	-509.229372
Benzimi_CO	7PdCO	-700.0960023	-699.5572013		-699.955721
benzimi_oxo_s	7PdO_s	-661.8465986	-661.3387996		-661.710474
benzimi_oxo_t	7PdO_t	-661.8587439	-661.328012	-661.3238196	-661.724477
MIC_CO	8PdO	-585.689849	-585.238395		-585.569741
MIC_oxo_s	8PdO_s	-547.445141	-547.023370-		-547.326394
MIC_oxo_t	8PdO_t	-547.455996	-547.0078983	-547.0030051	-547.342621
DAC_CO	9PdCO	-695.688442	-695.1971318		-695.612449
DAC_oxo_s	9PdO_s	-657.4415061	-656.9811522		-657.36636
DAC_oxo_t	9PdO_t	-657.4524218	-656.973585	-656.9687834	-657.37939
CAAC_CO	10PdCO	-649.5305978	-648.9949552		-649.321386
CAAC_oxo_s	10PdO_s	-611.291167	-610.7874144		-611.083817
CAAC_oxo_t	10PdO_t	-611.2947446	-610.7654978	-610.7592819	-611.090458

Table S6. Electronic energies (*E*) and Gibbs free energies (*G*) of Pd(II) terminal oxo complexes and related Pd(0) compounds with monodentate ligands.

**Name**	**Denominator**	***E* (B3LYP)**	***E* (B2PLYP)**	***E* (B2PLYP) o.s.s/UKS**	***G* (B3LYP)**
Pyridine_Cl2	1PdCl2	-1296.901180	-1296.391941		-1296.846421
Pyridine_Cl2_CO	1PdCl2_CO	-1410.331538	-1409.760164		-1410.270485
pyridine_oxo_s	1PdOCl2_s	-1372.036752	-1371.502445		-1371.981759
pyridine_oxo_t	1PdOCl2_t	-1372.077464	-1371.523291	-1371.485857	-1372.022402
NMe3_Cl2	2PdCl2	-1223.068584	-1222.596016		-1222.979704
NMe3_Cl2_CO	2PdCl2_CO	-1336.492894	-1335.95811		-1336.398438

NMe3_oxo_s	2PdOCl2_s	-1298.202694	-1297.711590		-1298.113204
NMe3_oxo_t	2PdOCl2_t	-1298.237211	-1297.719635	-1297.705231	-1298.149555
Isonitrile_Cl2	3PdCl2	-1373.128440	-1372.565392		-1373.070083
Isonitrile_Cl2_CO	3PdCl2CO	-1486.538681	-1485.91391		-1486.474367
Isonitrile_oxo_s	3PdOCl2_s	-1448.246495	-1447.652997		-1448.187225
Isonitrile_oxo_t	3PdOCl2_t	-1448.284006	-1447.673442	-1447.658189	-1448.227577
PMe3_Cl2	4PdCl2	-1509.757821	-1509.227705		-1509.679534
PMe3_Cl2_CO	4PdCl2CO	-1623.155731	-1622.5607		-1623.074019
PMe3_s	4PdOCl2_s	-1584.872743	-1584.310234		-1584.795272
PMe3_t	4PdOCl2_t	-1584.900430	-1584.320557	-1584.194466	-1584.824643
NHC_Cl2	5PdCl2	-1352.956460	-1352.956460		-1353.416862
NHC_Cl2_CO	5PdCl2CO	-1466.911315	-1466.297148		-1466.816762
NHC_oxo_s	5PdOCl2_s	-1428.042134	-1428.042134		-1428.534713
NHC_oxo_t	5PdOCl2_t	-1428.061057	-1428.061057	-1428.045349	-1428.570622
NHCS_Cl2	6PdCl2	-1354.715289	-1354.154755		-1354.602964
NHCS_Cl2_CO	6PdCl2CO	-1468.118634	-1467.4944208		-1468.001763
NHCS_oxo_s	6PdOCl2_s	-1429.831382	-1429.240255		-1429.718658
NHCS_oxo_t	6PdOCl2_t	-1429.867133	-1429.258563	-1429.242568	-1429.756553
Benzimi_Cl2	7PdCl2	-1507.228967	-1506.559163		-1507.096011
Benzimi_Cl2_CO	7PdCl2CO	-1620.632971	-1619.89990		-1620.495482
benzimi_oxo_s	7PdOCl2_s	-1582.344815	-1581.644790		-1582.211471
benzimi_oxo_t	7PdOCl2_t	-1582.380923	-1581.663442	-1581.647512	-1582.249609
MIC_Cl2	8PdCl2	-1392.832277	-1392.24853		-1392.131805
MIC_Cl2_CO	8PdCl2CO	-1506.234856	-1505.58753		-1506.114198
MIC_oxo_s	8PdCl2	-1467.950282	-1467.33774		-1467.22122
MIC_oxo_t	8PdCl2	-1467.984478	-1467.35361	-1467.3386623	-1467.238962
DAC_Cl2	9PdCl2	-1502.818831	-1502.196883		-1502.746038
DAC_Cl2_CO	9PdCl2CO	-1616.222612	-1615.53697		-1616.145919
DAC_oxo_s	9PdOCl2_s	-1577.934321	-1577.280942		-1577.861213
DAC_oxo_t	9PdOCl2_t	-1577.970105	-1577.298981	-1577.2660782	-1577.898235
CAAC_Cl2	10PdCl2	-1456.676434	-1456.010577		-1456.473479
CAAC_Cl2_CO	10PdCl2CO	-1570.076273	-1569.346052		-1569.867955
CAAC_oxo_s	10PdOCl2_s	-1531.795082	-1531.097200		-1531.592100
CAAC_oxo_t	10PdOCl2_t	-1531.825287	-1531.110601	-1531.070463	-1531.622422

Table S7. Electronic energies (*E*) and Gibbs free energies (*G*) of Pd(IV) terminal oxo complexes and related Pd(II) compounds with monodentate ligands.

**Name**	**Denominator**	***E* (B3LYP)**	***E* (B2PLYP)**	***E* (B2PLYP) o.s.s/UKS**	***G* (B3LYP)**
ethylenediamine_CO	11PdCO	-589.324853	-588.843775		-589.135186
ethylenediamine_oxo_s	11PdO_s	-551.085188	-550.636695		-550.895449
ethylenediamine_oxo_t	11PdO_t	-551.093638	-550.622695	-550.617962	-550.908792
propylenediamine_CO	12PdCO	-628.654996	-628.138539		-628.439074
propylenediamine_oxo_s	12PdO_s	-590.418114	-589.932134		-590.201214
propylenediamine_oxo_t	12PdO_t	-590.426615	-589.919647	-589.915876	-590.213116
Bisimine_CO	13PdCO	-508.226593	-507.834847		-508.143571
Bisimine_oxo_s	13PdO_s	-470.004054	-469.643541		-469.916620
Bisimine_oxo_t	13PdO_t	-469.997653	-469.614993	-469.615264	-469.914482
BiPy_CO	14PdCO	-737.010971	-736.459074		-736.888445
BiPy_oxo_s	14PdO_s	-698.780018	-698.255131		-698.656629
BiPy_oxo_t	14PdO_t	-698.779400	-698.230173	-698.231664	-698.661112
BiPy_Me_CO	15PdCO	-534.937105	-534.527881		-534.789432
BiPy_Me_oxo_s	15PdO_s	-738.110764	-737.551241		-737.960193
BiPy_Me_oxo_t	15PdO_t	-738.110196	-737.534557	-737.5343764	-737.963666
dmpe_CO	16PdCO_s	-1162.631237	-1162.027400		-1162.461254
dmpe_oxo_s	16PdO_s	not stable			
dmpe_oxo_t	16PdO_t	-1124.401178	-1123.810756	-1123.819824	-1124.235865
dmpp_CO	17PdCO	-1201.968962	-1201.328356		-1201.772291
dmpp_oxo_s	17PdO_s	-1163.755295	-1163.152050		-1163.556772
dmpp_oxo_t	17PdO_t	-1163.739209	-1163.113477	-1163.121269	-1163.546129
bisNHC_CO	18PdCO	-810.782217	-810.179450		-810.613431
bisNHC_oxo_s	18PdO_s	-772.582829	-772.014822		-772.413489
bisNHC_oxo_t	18PdO_t	-772.562916	-771.972226	-771.989278	-772.396817
bisNHCS_CO	19PdCO	-813.198290	-812.574746		-812.984623
bisNHCS_oxo_s	19PdO_s	-775.000546	-774.410006		-774.786170
bisNHCS_oxo_t	19PdO_t	-774.979478	-774.366701	-774.376681	-774.768349
bisBenzimi_CO	20PdCO	-876.782797	-876.120662		-876.528239
bisBenzimi_oxo_s	20PdO_s	-1080.032254	-1079.222990		-1079.775554
bisBenzimi_oxo_t	20PdO_t	-1080.012213	-1079.177588	-1079.188867	-1079.758776
bisMIC_CO	21PdCO	-921.449858	-920.779954		-921.254636

bisMIC_oxo_s	21PdO_s	-883.252303	-882.612671		-883.056414
bisMIC_oxo_t	21PdO_t	-883.232008	-882.571334	-882.579973	-883.039153
bisMIC_diazo_CO	21PdCO	-647.964641	-647.472663		-647.748479
bisMIC_diazo_oxo_s	21PdO_s	-851.206262	-850.571240		-850.986739
bisMIC_diazo_oxo_t	21PdO_t	-851.185189	-850.528228	-850.538287	-850.969627
bisDAC_CO	22PdCO	not stable			
bisDAC_oxo_s	22PdO_s	-1071.231469	-1070.518666		-1071.097291
bisDAC_oxo_t	22PdO_t	-1071.216033	-1070.480298	-1070.489236	-1071.084542
bisCAAC_CO	23aPdCO	-1017.103787	-1016.268218		-1016.705861
bisCAAC_oxo_s	23aPdO_s	-978.905544	-978.109078		-978.509070
bisCAAC_oxo_t	23aPdO_t	-978.885899	-978.064073		-978.492783
bisCAAC_diastereo_CO	23bPdCO	-1017.102357	-1016.266568		-1016.704931
bisCAAC_diastereo_oxo_s	23bPdO_s	-978.912325	-978.110272		-978.515307
bisCAAC_diastereo_oxo_t	23bPdO_t	-978.884337	-978.061118	-978.073797	-978.490486
bisCAAC_N_CO	24PdCO	-1017.102236	-1016.265860		-1016.704747
bisCAAC_oxo_s_N	24PdO_s	-978.913582	-978.117358		-978.518769
bisCAAC_oxo_t_N	24PdO_t	-978.886981	-978.063736	-978.096332	-978.496092
CAAC_NHC_CO	25PdCO	-913.943153	-913.224408		-913.658540
CAAC_NHC_oxo_s	25PdO_s	-875.746983	-875.064387		-875.465269
CAAC_NHC_oxo_t	25PdO_t	-875.730793	-875.023415	-875.028192	-875.450973
CAAC_benzimi_CO	26PdCO	-1067.674853	-1066.836629		-1067.347716
CAAC_benzimi_oxo_s	26PdO_s	-1029.472686	-1028.670275		-1029.146487
CAAC_benzimi_oxo_t	26PdO_t	-1029.455226	-1028.628803	-1028.634455	-1029.132362
CAAC_MIC_CO	27PdCO	-873.362289	-872.689623		-873.129607
CAAC_MIC_oxo_s	27PdO_s	-835.155058	-834.519313		-834.923912
CAAC_MIC_oxo_t	27PdO_t	-835.144313	-834.487111	-834.492490	-834.916440
CAAC_PMe2_CO	28PdCO	-1453.848770	-1452.856106		-1453.481517
CAAC_PMe2_oxo_s	28PdO_s	-1415.637870	-1414.680219		-1415.267882
CAAC_PMe2_oxo_t	28PdO_t	-1415.623284	-1414.643984	-1414.651042	-1415.259748

Table S8. Electronic energies (*E*) and Gibbs free energies (*G*) of Pd(II) terminal oxo complexes and related Pd(0) compounds with bidentate ligands.

**Name**	**Denominator**	***E* (B3LYP)**	***E* (B2PLYP)**	***E* (B2PLYP) o.s.s./UKS**	***G* (B3LYP)**
ethylenediamine_Cl2_oxo_s	11PdOCl2	-1396.477416	-1395.866588		-1396.288422
ethylenediamine_Cl2_oxo_s	11PdOCl2_s	-1471.592558	-1470.961099		-1471.400570
ethylenediamine_oxo_t	11PdOCl2_t	-1471.616720	-1470.955286	-1470.951588	-1471.429686
propylenediamine_Cl2	12PdOCl2	-1435.802570	-1435.152852		-1435.585361
propylenediamine_Cl2_oxo_s	12PdOCl2_s	-1510.929201	-1510.261480		-1510.709767
propylenediamine_Cl2_oxo_t	12PdOCl2_t	-1510.952199	-1510.254500	-1510.253191	-1510.735574
Bisimine_Cl2	13PdOCl2	-1315.374758	-1314.851887		-1315.289401
Bisimine_Cl2_oxo_s	13PdOCl2_s	-1390.487380	-1389.945460		-1390.401592
Bisimine_Cl2_oxo_t	13PdOCl2_t	-1390.509330	-1389.937971	-1389.927078	-1390.427262
BiPy_Cl2	14PdOCl2	-1544.163119	-1543.477405		-1544.041610
BiPy_Cl2_oxo_s	14PdOCl2_s	-1619.272089	-1618.568956		-1619.150982
BiPy_Cl2_oxo_t	14PdOCl2_t	-1619.293540	-1618.562451	-1618.551796	-1619.176220
BiPy_Me_Cl2	15PdOCl2	-1583.496508	-1582.775820		-1583.347184
BiPy_Me_Cl2_oxo_s	15PdOCl2_s	-1658.609872	-1657.869311		-1658.459863
BiPy_Me_Cl2_oxo_t	15PdOCl2_t	-1658.631244	-1657.861027	-1657.856813	-1658.484607
dmpe_Cl2_oxo_s	16PdOCl2_s	not stable			
dmpe_Cl2_oxo_t	16PdOCl2_t	not stable			
dmpp_Cl2	17PdOCl2	-2009.140700	-2008.377060		-2008.944525
dmpp_Cl2_oxo_s	17PdOCl2_s	-2084.256113	-2083.468791		-2084.058828
dmpp_Cl2_oxo_t	17PdOCl2_t	-2084.269221	-2083.452843	-2083.450878	-2084.075751
bisNHC_Cl2	18PdCl2	-1617.964855	-1617.233065		-1617.797353
bisNHC_Cl2_oxo_s	18PdOCl2_s	-1693.082244	-1692.329159		-1692.913496
bisNHC_Cl2_oxo_t	18PdOl2_t	-1693.095113	-1692.312844	-1692.310244	-1692.929224
bisNHCS_Cl2	19PdCl2	-1620.381372	-1619.632359		-1620.169173
bisNHCS_Cl2_oxo_s	19PdOCl2_s	-1695.500877	-1694.726736		-1695.287755
bisNHCS_Cl2_oxo_t	19PdOCl2_t	-1695.512041	-1694.708833	-1694.706269	-1695.301918
bisBenzimi_Cl2	20PdOCl2	-1925.411176	-1924.440733		-1925.156241
bisBenzimi_Cl2_oxo_s	20PdOCl2_s	-2000.526833	-1999.534897		-2000.271379
bisBenzimi_Cl2_oxo_t	20PdOCl2_t	-2000.539616	-1999.518455	-1999.515832	-2000.286770
bisMIC_Cl2	21PdCl2	-1728.640745	-1727.840246		-1728.445727
bisMIC_Cl2_oxo_s	21PdOCl2_s	-1803.759449	-1802.937220		-1803.563863
bisMIC_Cl2_oxo_t	21PdOCl2_t	-1803.771086	-1802.920132	-1802.917752	-1803.578446
bisMIC_Cl2_diazo	21PdCl2	-1696.598645	-1695.802881		-1696.380592

bisMIC_Cl2_diazo_oxo_s	21PdOCl2_s	-1771.721656	-1770.903232		-1771.503070
bisMIC_Cl3_diazo_oxo_t	21PdOCl2_t	-1771.732773	-1770.885866	-1770.883519	-1771.516478
bisDAC_Cl2	22PdCl2	-1916.592834	-1915.717649		-1916.460556
bisDAC_Cl2_oxo_s	22PdOCl2_s	-1991.703803	-1990.806581		-1991.570591
bisDAC_Cl2_oxo_t	22PdOCl2_t	-1991.717962	-1990.790666	-1990.787632	-1991.586649
bisCAAC_Cl2	23aPdOCl2	-1824.290349	-1823.327829		-1823.896134
bisCAAC_oxo_s	23aPdOCl2_s	-1899.409026	-1898.422771		-1899.014990
bisCAAC_oxo_t	23aPdOCl2_t	-1899.401540	-1898.384777	-1898.375324	-1899.003761
bisCAAC_diastereo_Cl2	23bPdOCl2	-1824.289908	-1823.326121		-1823.894010
bisCAAC_Cl2_diastereo_oxo_s	23bPdOCl2_s	-1899.412993	-1898.426764		-1899.016558
bisCAAC_Cl2_diastereo_oxo_t	23bPdOCl2_t	-1899.429093	-1898.414417	-1898.412019	-1899.034535
bisCAAC_N_Cl2	24PdOCl2	-1824.283508	-1823.319910		-1823.888727
bisCAAC_N_Cl2_oxo_s	24PdOCl2_s	-1899.412324	-1898.426232		-1899.016314
bisCAAC_N_Cl2_oxo_t	24PdOCl2_t	-1899.421356	-1898.406954	-1898.403076	-1899.029918
CAAC_NHC_Cl2	25PdOCl2	-1721.130384	-1720.283630		-1720.847751
CAAC_NHC_Cl2_oxo_s	25PdOCl2_s	-1796.245138	-1795.375485		-1795.961800
CAAC_NHC_Cl2_oxo_t	25PdOCl2_t	-1796.260083	-1795.361138	-1795.358897	-1795.980349
CAAC_benzimi_Cl2	26PdOCl2	-1874.857563	-1873.891448		-1874.532885
CAAC_benzimi_Cl2_oxo_s	26PdOCl2_s	-1949.967410	-1948.978837		-1949.640579
CAAC_benzimi_Cl2_oxo_t	26PdOCl2_t	-1949.982674	-1948.965086	-1948.962860	-1949.659015
CAAC_MIC_Cl2	27PdOCl2	-1680.541962	-1679.742553		-1680.311796
CAAC_MIC_Cl2_oxo_s	27PdOCl2_s	-1755.653783	-1754.837754		-1755.421880
CAAC_MIC_Cl2_oxo_t	27PdOCl2_t	-1755.669289	-1754.818272	-1770.815955	-1755.441218
CAAC_PMe2_Cl2	28PdOCl2	-2261.020470	-2259.901230		-2260.655836
CAAC_PMe2_oxo_s	28PdOCl2_s	-2336.130908	-2334.989843		-2335.765493
CAAC_PMe2_oxo_t	28PdOCl2_t	-2336.150879	-2334.982402	-2334.978323	-2335.786611

Table S9. Electronic energies (*E*) and Gibbs free energies (*G*) of Pd(IV) complexes with bidentate ligands.

**Name**	**Denominator**	**HF(B3LYP)**	**HF (B2PLYP)**	**HF(B2PLYP) o.s.s/UKS**	**G(B3LYP)**
terpy_Py_CO	29PdCO	-984.2154844	-983.4861845		-984.030101
terpy_py_s	29PdO_s	-946.0146234	-945.3112262		-945.82635
terpy_Py_t	29PdO_t	-946.0106964	-945.2838629	-945.2909744	-945.826188

Pincer_Py_Bismimine_CO	30PdCO	-834.110604	-833.4684372		-833.908666
Pincer_Py_Bismimine_oxo_s	30PdCO_s	-795.9130563	-795.3048811		-795.70895
Pincer_Py_Bismimine_oxo_t	30PdCO_t	-795.9136219	-795.2791518	-795.2787427	-795.713828
Pincer_Py_bisNHC_CO	31PdCO	-1018.652313	-1017.906298		-1018.447699
Pincer_Py_bisNHC_oxo_s	31PdO_s	-980.4720328	-979.7539807		-980.264987
Pincer_Py_bisNHC_oxo_t	31PdO_t	-980.4605066	-979.7213344	-979.7278908	-980.259353
Pincer_Py_bisNHCS_CO	32PdCO	-1021.079342	-1020.314377		-1020.82781
Pincer_Py_bisNHCS_oxo_s	32PdO_s	-982.9014606	-982.1630369		-982.651724
Pincer_Py_bisNHCS_oxo_t	32PdO_t	-982.8838299	-982.1252574	-982.1380795	-982.637042
Pincer_Py_benzimi_CO	33PdCO	-1326.103134	-1325.1192		-1325.808797
Pincer_Py_benzimi_oxo_s	33PdO_s	-1287.920671	-1286.963959		-1287.626938
Pincer_Py_benzimi_oxo_t	33PdO_t	-1287.907516	-1286.9304	-1286.939919	-1287.6161
Pincer_Py_bisMIC_CO	34PdCO	-1097.276412	-1096.461743		-1097.021426
Pincer_Py_bisMIC_oxo_s	34PdO_s	-1059.089092	-1058.306754		-1058.834054
Pincer_Py_bisMIC_oxo_t	34PdO_t	-1059.096419	-1058.287722	-1058.28742	-1058.846039
Pincer_Py_DAC_CO	35PdCO	-1317.323869	-1316.438126		-1317.147845
Pincer_Py_DAC_oxo	35PdO_s	-1279.120194	-1278.341501		-1278.94678
Pincer_Py_DAC_oxo_t	35PdO_t	-1279.122239	-1278.251669	-1278.248951	-1278.950833
Pincer_Py_bisCAAC_CO	36PdCO	-1224.99483	-1224.021297		-1224.558363
Pincer_Py_bisCAAC_oxo_s	36PdO_s	-1186.79159	-1185.844575		-1186.358958
Pincer_Py_bisCAAC_oxo_t	36PdO_t	-1186.797726	-1185.828524	-1185.828524	-1186.367667

Table S10. Electronic energies (*E*) and Gibbs free energies (*G*) of Pd(II) complexes with tridentate ligands.

**Name**	**Denominator**	***E* (B3LYP)**	***E* (B2PLYP)**	***E* (B2PLYP) o.s.s/UKS**	***G* (B3LYP)**
terpy_Ph_CO	29PdCO+	-967.372160	-966.662745		-967.179241
terpy_ph_oxo_s	29PdO_s+	-929.098486	-928.4203601		-928.908832
terpy_Ph_oxo_t	29PdO_t+	-929.124367	-928.429929	-928.414119	-928.937697
Pincer_Ph_Bismimine_CO	30PdCO+	-817.267057	-816.647939		-817.059431
Pincer_Ph_Bismimine_oxo_s	30PdCO_s+	-778.990408	-778.403547		-778.787314
Pincer_Ph_Bismimine_oxo_t	30PdCO_t+	-779.016824	-778.413157	-778.245249	-778.816501
Pincer_Ph_NHC_CO	31PdCO+				
Pincer_Ph_NHC_oxo_s	31PdO_s+	-963.562783	-962.867850		-963.355889

Pincer_Ph_NHC_oxo_t	31PdO_t+	-963.600023	-962.891155	-962.872281	-963.394499
Pincer_Ph_NHCS_CO	32PdCO+	-1004.26504	-1003.51777		-1004.01386
Pincer_Ph_NHCS_oxo_s	32PdO_s+	-965.991038	-965.277643		-965.739848
Pincer_Ph_NHCS_oxo_t	32PdO_t+	-966.026366	-965.295891	-965.284462	-965.776373
Pincer_Ph_benzimi_CO	33PdCO+	-1309.286407	-1308.321723		-1308.990407
Pincer_Ph_benzimi_oxo_s	33PdO_s+	-1271.011104	-1270.079349		-1270.717060
Pincer_Ph_benzimi_oxo_t	33PdO_t+	-1271.044225	-1270.095676	-1270.078812	-1270.751216
Pincer_Ph_bisMIC_CO	34PdCO+	-1080.510464	-1079.713056		-1080.248053
Pincer_Ph_bisMIC_oxo_s	34PdO_s+	-1042.238934	-1041.476334		-1041.978222
Pincer_Ph_bisMIC_oxo_t	34PdO_t+	-1042.265722	-1041.486674	-1041.475142	-1042.008854
Pincer_Ph_DAC_CO	35PdCO+	-1300.434451	-1299.572932		-1300.258253
Pincer_Ph_DAC_oxo_s	35PdO_s+	-1262.151703	-1261.321280		-1261.980050
Pincer_Ph_DAC_oxo_t	35PdO_t+	-1262.187882	-1261.341211	-1261.32851	-1262.018461
Pincer_Ph_bisCAAC_CO	36PdCO+	-1208.166909	-1207.207784		-1207.729517
Pincer_Ph_bisCAAC_oxo_s	36PdO_s+	-1169.902624	-1168.979027		-1169.469144
Pincer_Ph_bisCAAC_oxo_t	36PdO_t+	-1169.922185	-1168.980381	-1168.966208	-1169.490734

Table S11. Electronic energies (*E*) and Gibbs free energies (*G*) of Pd(IV) complexes with tridentate ligands.

7. XYZ Coordinates of CASSCF(8,8)/def2-TZVPP Optimized Structures

13^{PdOCl2}_dzeta_triplet

NImag = 0

C	-0.000718	1.611185	-2.549676
N	0.015055	1.343300	-1.130933
Pd	0.028574	-0.621953	-0.184792
O	0.040967	-2.230400	0.734614
N	0.006231	0.723347	1.495496
C	-0.011540	0.258349	2.857639
C	0.011553	1.937727	1.163213
C	0.015813	2.275163	-0.280927
Cl	-2.330782	-0.674199	-0.201726
Cl	2.388184	-0.661347	-0.204693
H	0.005415	2.751113	1.886770
H	0.010821	3.324207	-0.573378
H	-0.067316	1.081406	3.570582
H	-0.868782	-0.399042	2.978349
H	0.892241	-0.325603	3.021544
H	0.876084	1.151383	-2.999421
H	-0.886274	1.145554	-2.976408
H	-0.007107	2.681156	-2.765659

13^{PdOCl2}_dzeta_open_shell_singlet

NImag = 0

C	-0.000135	1.610468	-2.549474
N	0.016187	1.347037	-1.130242
Pd	0.029655	-0.614104	-0.188909
O	0.041844	-2.244224	0.735778
N	0.005953	0.724721	1.496316
C	-0.012934	0.259684	2.858303
C	0.011557	1.939452	1.164167
C	0.016243	2.278640	-0.279858
Cl	-2.334514	-0.677783	-0.205381
Cl	2.394138	-0.664566	-0.208081
H	0.005297	2.752380	1.888250
H	0.010783	3.327845	-0.571730
H	-0.069830	1.082360	3.571493
H	-0.870277	-0.397911	2.977800
H	0.890988	-0.323946	3.023169
H	0.875865	1.147982	-2.998210
H	-0.886602	1.143736	-2.973434
H	-0.005800	2.679576	-2.769365

13^{PdOCl2}_dzeta_singlet

NImag = 0

C	-0.002160	1.519069	-2.473362
N	0.024304	1.324079	-1.039934
Pd	0.030056	-0.705551	-0.081326
O	0.020201	-2.422896	-0.280391
N	0.006501	0.811459	1.535947
C	-0.016206	0.367028	2.906789
C	0.021203	2.020917	1.194162
C	0.027731	2.301242	-0.261556
Cl	-2.341637	-0.691849	-0.070770
Cl	2.398002	-0.699219	-0.079406
H	0.018033	2.847612	1.902426
H	0.022632	3.333264	-0.610562
H	-0.137155	1.193395	3.607318
H	-0.838235	-0.333467	3.022404
H	0.918486	-0.159500	3.096677
H	0.868601	1.042895	-2.918047
H	-0.893476	1.042379	-2.876670
H	-0.008460	2.580489	-2.733106

13^{PdOCl2}_tzeta_triplet

NImag = 0

C	-0.000250	1.606568	-2.497794
N	0.009304	1.365599	-1.076512
Pd	0.021887	-0.639102	-0.234343
O	-0.047336	-2.379494	0.373857
N	-0.009492	0.728756	1.498647
C	0.032939	0.346781	2.891937
C	-0.009942	1.943939	1.186425
C	-0.025087	2.303640	-0.248651
Cl	-2.308528	-0.812092	-0.457281
Cl	2.374144	-0.805644	-0.100677
H	0.016375	2.734513	1.921080
H	-0.062828	3.342609	-0.541560
H	0.265317	1.199970	3.518799
H	-0.926626	-0.077854	3.154475
H	0.789413	-0.406274	3.025310
H	0.962449	1.301292	-2.887640
H	-0.754832	0.977145	-2.937024
H	-0.208489	2.640995	-2.748454

13^{PdOCl2}_tzeta_open_shell_singlet

NImag = 0

C	-0.007385	1.611004	-2.497532
N	0.010403	1.369397	-1.076220
Pd	0.027765	-0.638067	-0.238289
O	-0.040067	-2.417906	0.355733
N	-0.007021	0.731977	1.501976
C	0.028442	0.354862	2.896607
C	-0.003612	1.946807	1.187426
C	-0.019458	2.306776	-0.247589
Cl	-2.302157	-0.833081	-0.469957
Cl	2.383158	-0.821340	-0.093673
H	0.024351	2.738289	1.921101
H	-0.055724	3.346043	-0.539843
H	0.264648	1.208407	3.521513
H	-0.935150	-0.062320	3.156915
H	0.779020	-0.403174	3.035187
H	0.951029	1.301349	-2.894166
H	-0.768549	0.985726	-2.931799
H	-0.211275	2.646599	-2.746797

13^{PdOCl2}_tzeta_singlet

NImag = 0

C	0.005581	1.540095	-2.463136
N	0.003693	1.327754	-1.035888
Pd	0.008514	-0.703724	-0.089345
O	0.008509	-2.425728	-0.167568
N	-0.012463	0.785545	1.505804
C	0.027958	0.374983	2.890459
C	-0.017097	1.995586	1.186768
C	-0.027141	2.305293	-0.255105
Cl	-2.364646	-0.708979	-0.281162
Cl	2.393705	-0.734083	0.017641
H	0.002649	2.793147	1.912817

H	-0.052514	3.331246	-0.591803
H	0.238368	1.214845	3.539759
H	-0.924268	-0.077040	3.134677
H	0.801832	-0.363839	3.007745
H	0.968703	1.225936	-2.843374
H	-0.752407	0.915674	-2.901253
H	-0.190552	2.574633	-2.726441

8. XYZ Coordinates of B2PLYP-D3/def2-TZVPP Optimized Structures

13^{PdOCl2}_B2PLYP_triplet (*NImag* = 0)

Pd	0.659227	0.035936	-0.147196
Cl	0.545994	2.328213	0.210704
Cl	0.726801	-2.281630	-0.297114
O	2.163418	0.206377	-1.108045
N	-1.632833	0.060135	-0. 939852
N	-0.625787	-0.202825	1.499790
C	0.006209	-0.349000	2.794375
C	-2.065957	0.225329	-2.302725
C	-1.888704	-0.219458	1.363573
C	-2.451573	-0.066589	0.016026
H	-3.152805	0.219235	-2.403502
H	-1.632086	-0.576424	-2.896598
H	-1.663443	1.165917	-2.673167
H	0.616232	0.533264	2.971191
H	0.657883	-1.218188	2.751678
H	-0.729701	-0.465843	3.588695
H	-3.532776	-0.065539	-0.114171
H	-2.546864	-0.342520	2.218431

13^{PdOCl2}_B2PLYP_singlet (*NImag* = 0)

Pd	0.000191	-0.561023	-0.010287
Cl	2.348331	-0.528590	-0.008340
Cl	-2.347969	-0.529224	-0.013519
O	0.000476	-2.346564	-0.035630
N	-0.005091	1.072067	1.279857
N	0.004735	1.121779	-1.238457
C	0.002709	2.269946	-0.683197
C	-0.003860	2.241267	0.770620
C	-0.012513	0.816069	2.701292
C	0.012246	0.924406	-2.669093
H	0.014840	1.874683	-3.200629
H	-0.869875	0.343820	-2.929754
H	0.897542	0.344376	-2.920136
H	0.869971	0.225744	2.937829
H	-0.897378	0.225456	2.928121
H	-0.015809	1.743607	3.271555
H	0.005828	3.194217	-1.250475
H	-0.007653	3.142042	1.374501

13^{PdOCl2}_B2PLYP_o.s.s. (*NImag* = 0)

Pd	0.479606	0.028192	-0.177746
Cl	0.553350	2.310709	0.227124
Cl	0.627857	-2.285325	-0.314904
O	1.433493	0.238118	-1.772149
N	-1.551636	0.067367	-0.876012
N	-0.625704	-0.209095	1.574558
C	-0.012326	-0.342482	2.876702
C	-1.861421	0.237823	-2.271945
C	-1.889039	-0.236159	1.414892
C	-2.404752	-0.084964	0.049579
H	-2.935410	0.203920	-2.452083
H	-1.349390	-0.545230	-2.825532
H	-1.445060	1.190547	-2.590556
H	0.579955	0.551372	3.058161
H	0.655649	-1.199979	2.844061
H	-0.756067	-0.470713	3.661941
H	-3.474138	-0.103305	-0.142630
H	-2.571732	-0.364410	2.248625

9. XYZ Coordinates of Optimized Carbene Structures for TEP[4]

CAAC (*NImag* = 0)

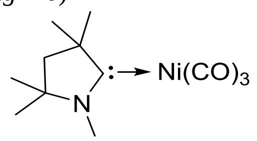

ν(CO, unscaled): 2148.3 cm^{-1}

ν(CO, scaled): 2049.7 cm^{-1} (*TEP*)

Ni	-3.20387	2.62266	-0.50557
C	-2.18575	1.92965	-1.81482
C	-3.84119	4.20585	-1.09931
C	-2.29380	2.90369	1.01903
O	-1.56214	1.49010	-2.66581
O	-1.73995	3.07977	2.00290
O	-4.16693	5.22914	-1.48962
C	-4.64345	1.33875	-0.11144
C	-6.14036	1.59149	-0.25406
C	-5.72003	-0.73463	0.44321
C	-6.56679	2.84715	0.50863
H	-6.26904	2.79327	1.55857
H	-6.12534	3.74568	0.07932
H	-7.65540	2.94882	0.46855
C	-6.47055	1.75989	-1.74364
H	-5.93678	2.61227	-2.16503
H	-6.19910	0.87634	-2.32530
H	-7.54396	1.92972	-1.86469
C	-3.19724	-0.52330	0.52317
H	-2.41939	0.17438	0.22902
H	-3.08412	-0.76291	1.58215
H	-3.10215	-1.44212	-0.05803
N	-4.48729	0.09708	0.28118
C	-5.72617	-1.44108	1.79594
H	-6.69636	-1.91919	1.94780
H	-4.96343	-2.22021	1.85901
H	-5.56908	-0.72985	2.60998
C	-5.80364	-1.76144	-0.68821
H	-4.98196	-2.47984	-0.64769
H	-6.73496	-2.32509	-0.60007
H	-5.78881	-1.27833	-1.66609
C	-6.81060	0.33764	0.34966
H	-7.66120	-0.00470	-0.24255
H	-7.18354	0.56524	1.35128

DAC (*NImag* = 0)

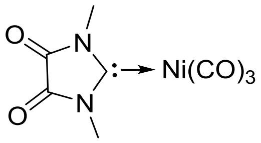

ν(CO, unscaled): 2171.3 cm^{-1}

ν(CO, scaled): 2071.6 cm^{-1} (*TEP*)

C	-4.48884	1.16405	-0.18494
C	-5.91656	-0.57871	-0.65293
C	-5.78262	-0.42297	0.86477
O	-6.57001	-1.37620	-1.25825
O	-6.30690	-1.07055	1.72223
C	-4.92062	0.60570	-2.58960
H	-5.32091	-0.27497	-3.08926
H	-5.46148	1.48815	-2.93199
H	-3.86221	0.71506	-2.81689
C	-4.48752	1.10940	2.32067
H	-4.97070	2.05594	2.56349
H	-4.78652	0.35806	3.04975
H	-3.40693	1.23587	2.33724
N	-4.90408	0.63983	1.01406
N	-5.09610	0.41632	-1.16319
Ni	-3.26811	2.61960	-0.44580
C	-1.58812	1.98181	-0.56011
C	-3.65104	3.54534	-1.96280
C	-3.34658	3.81626	0.92163
O	-0.52893	1.56983	-0.63035
O	-3.37405	4.60040	1.74672
O	-3.86795	4.16234	-2.89486

MIC (*NImag* = 0)

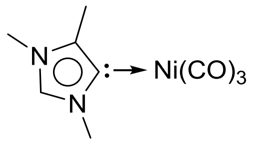

ν(CO, unscaled): 2139.5 cm^{-1}

ν(CO, scaled): 2041.3 cm^{-1} (*TEP*)

C	-4.42131	1.23094	-0.24292
C	-5.86120	-0.27106	0.64195
C	-4.61622	0.38884	-2.68687
H	-4.16704	-0.55358	-3.01646
H	-5.50834	0.56749	-3.29576
H	-3.90586	1.18733	-2.89337
C	-4.87517	1.30588	2.23665
H	-5.16760	2.35455	2.24222
H	-5.48869	0.74990	2.94552
H	-3.82716	1.23234	2.52222
N	-5.05658	0.75774	0.90775
Ni	-3.12671	2.74246	-0.39322
C	-1.62653	2.00445	-1.05585
C	-3.89951	3.90454	-1.52748
C	-2.77143	3.55362	1.16293
O	-0.67991	1.50989	-1.46547
O	-2.51056	4.10627	2.13245
O	-4.41686	4.63378	-2.24093
C	-4.91105	0.39867	-1.23172
H	-6.46381	-0.81593	1.34833
N	-5.79190	-0.51383	-0.66489
C	-6.51724	-1.55513	-1.36176
H	-7.12782	-2.10831	-0.65044
H	-7.16591	-1.11826	-2.12151
H	-5.82039	-2.24307	-1.84131

10. XYZ Files of B3LYP Optimized Structures

acetone (*NImag* = 0)

C	-3.67596	2.81800	1.26822
C	-3.62150	1.97090	0.01599
C	-3.71787	2.70999	-1.30048
O	-3.50718	0.76856	0.06468
H	-2.92180	3.60775	1.23008
H	-4.64882	3.31205	1.33815
H	-3.51988	2.19671	2.14686
H	-4.60744	3.34420	-1.32000
H	-2.85485	3.37107	-1.41718
H	-3.74755	2.00206	-2.12529

Cl2 (*NImag* = 0)

Cl	-0.31032	0.22321	0.00000
Cl	-2.32444	0.22321	0.00000

CO2 (*NImag* = 0)

C	-0.10973	0.21547	0.03333
O	-1.26911	0.22167	0.00663
O	1.04966	0.20927	0.06004

CO (*NImag* = 0)

C	-0.60214	0.22321	0.00000
O	-1.72703	0.22321	0.00000

DMDO (*NImag* = 0)

C	-3.68092	2.00063	-0.00000
O	-4.42914	0.81819	0.00000
O	-2.93210	0.81857	-0.00000
C	-3.68112	2.77789	1.28571
H	-2.79818	3.41745	1.33850
H	-4.56440	3.41699	1.33851
H	-3.68094	2.09084	2.12890
C	-3.68113	2.77788	-1.28571
H	-4.56441	3.41699	-1.33850
H	-2.79819	3.41744	-1.33851
H	-3.68097	2.09084	-2.12890

PhICl2 (*NImag* = 0)

C	-0.38482	0.66317	-0.09895
C	0.97153	0.81330	0.17025
C	1.55793	2.07376	0.22364
C	0.77239	3.19790	0.00150
C	-0.58477	3.05747	-0.27048
C	-1.16236	1.79357	-0.32185
H	-0.83477	-0.32081	-0.13052
H	2.61260	2.18154	0.44161
H	1.21975	4.18256	0.04168
H	-1.19355	3.93554	-0.44167
H	-2.21854	1.68640	-0.53228
I	2.20198	-0.94550	0.38323
Cl	1.81980	-0.71563	2.72892
Cl	3.60450	-3.02623	1.08622

PhI (*NImag* = 0)

C	1.29130	-0.13603	0.00004
C	2.68204	-0.14413	0.00055
C	3.36450	1.06561	0.00002
C	2.68214	2.27542	-0.00100
C	1.29140	2.26744	-0.00149
C	0.59424	1.06574	-0.00097
H	0.75540	-1.07638	0.00045
H	3.22262	-1.07962	0.00134
H	3.22282	3.21086	-0.00140
H	0.75559	3.20784	-0.00229
H	-0.48757	1.06579	-0.00136
I	5.47765	1.06553	0.00078

CH4 (*NImag* = 0)

C	-4.51141	-0.05725	-0.00000
H	-4.87441	-0.86222	0.63731
H	-4.87442	0.89717	0.37847
H	-4.87443	-0.20668	-1.01579
H	-3.42241	-0.05725	0.00001

CH3OH (*NImag* = 0)

O	-4.33205	0.04921	-0.01101
C	-5.75221	0.06581	-0.00352
H	-4.03454	-0.78471	-0.38753
H	-6.05306	1.01973	0.42718
H	-6.17186	-0.00429	-1.01285
H	-6.17186	-0.73764	0.61141

NMe3_oxo_s (*NImag* = 0)

C	-1.97569	-1.18340	0.06419
H	-1.74357	-1.73042	0.97620
H	-3.04498	-0.94043	0.05863
H	-1.75177	-1.80705	-0.79941
C	-1.47150	0.80352	-1.23290
H	-2.52423	1.11221	-1.23325
H	-0.81894	1.67123	-1.27560
H	-1.27824	0.17144	-2.09614
C	-1.46037	0.90769	1.18055
H	-1.25893	0.35226	2.09324
H	-0.80778	1.77559	1.14233
H	-2.51317	1.21569	1.16399
N	-1.17115	0.04909	0.00728
Pd	0.89020	-0.34407	0.01474
O	1.52591	1.33404	-0.06068

amine_oxo_t (*NImag* = 0)

C	-4.05635	0.16630	-0.02800
H	-4.42063	1.19119	-0.02266
H	-4.43953	-0.34890	-0.91858
H	-4.42127	-0.33927	0.86330
C	-2.08766	-1.22647	-0.02712
H	-2.44602	-1.76005	-0.91723
H	-0.99995	-1.22863	-0.02194
H	-2.44271	-1.73813	0.86468
C	-2.08660	0.86216	-1.23274
H	-2.44175	1.89029	-1.23044
H	-0.99886	0.86819	-1.23076
H	-2.44408	0.35759	-2.13994
N	-2.57997	0.16541	-0.02836
Pd	-1.85129	1.19503	1.75560
O	-1.24938	2.04559	3.22825

benzimi_oxo_t (*NImag* = 0)

N	-0.59810	0.90326	-0.69076
C	-1.15230	-0.30583	-0.41684
Pd	-0.14655	-2.06987	0.06620
O	0.74299	-3.58982	0.55561
N	-2.49444	-0.12605	-0.51901
C	-1.57333	1.85034	-0.96523
C	-2.80003	1.18466	-0.85430
C	-1.50578	3.19746	-1.29370
C	-4.00391	1.84242	-1.06805
C	-2.71009	3.85623	-1.50765
C	-3.93696	3.19059	-1.39684
H	-4.95281	1.33136	-0.98362
H	-2.70034	4.90643	-1.76596
H	-4.85290	3.73869	-1.57119
H	-0.55995	3.71377	-1.37994
C	-3.48291	-1.16584	-0.30323
H	-2.95753	-2.08076	-0.04115
H	-4.15274	-0.88410	0.51013
H	-4.06510	-1.32661	-1.21144
C	0.82734	1.17351	-0.69451
H	1.34468	0.25375	-0.43333
H	1.14552	1.50323	-1.68429
H	1.06521	1.94621	0.03758

benzimi_oxo_s (*NImag* = 0)

N	-0.49399	0.96785	-0.64903
C	-0.99504	-0.27348	-0.40008
Pd	0.05153	-1.86453	0.06244
O	-1.07476	-3.25034	0.25167
N	-2.33881	-0.18245	-0.52866
C	-1.51957	1.85943	-0.93339
C	-2.70414	1.11753	-0.85715
C	-1.52558	3.21126	-1.24306
C	-3.93927	1.70348	-1.09064
C	-2.76440	3.79906	-1.47684
C	-3.94852	3.05885	-1.40212
H	-4.85513	1.13262	-1.03286
H	-2.81240	4.85152	-1.72118
H	-4.89282	3.55139	-1.59036
H	-0.61211	3.78685	-1.30032
C	-3.28540	-1.27607	-0.35377
H	-3.84305	-1.42087	-1.27980
H	-2.72525	-2.18185	-0.10481
H	-3.97881	-1.02721	0.45045
C	0.91135	1.31060	-0.60975
H	1.11310	2.01735	0.19624
H	1.49162	0.40557	-0.43844
H	1.22140	1.74650	-1.56006

CAAC_oxo_s (*NImag* = 0)

C	2.83774	-2.72481	1.76157
N	1.51909	-3.08247	1.14205
C	1.20868	-4.34737	1.19175
C	2.29912	-5.13044	1.90495
C	3.46231	-4.11716	1.97082
C	0.66407	-2.04371	0.57356
Pd	-0.36837	-5.21728	0.50128
O	-1.56838	-4.00999	-0.07018
C	2.67166	-6.38976	1.11688
C	1.79207	-5.53142	3.30123
C	3.66624	-1.87155	0.80467
C	2.60336	-1.97300	3.07398
H	4.01004	-4.17547	2.91078
H	3.49097	-6.91485	1.61249
H	1.82635	-7.07986	1.05330
H	0.93246	-6.19691	3.22373
H	2.58627	-6.04933	3.84268
H	1.48490	-4.66607	3.88715
H	2.98095	-6.14382	0.10097
H	4.16802	-4.32016	1.16454
H	3.74989	-2.35101	-0.17140
H	3.23958	-0.87765	0.67072
H	4.67072	-1.75007	1.21247
H	2.10026	-1.02167	2.90113
H	1.99960	-2.55785	3.76579
H	3.56211	-1.76100	3.54870
H	1.17270	-1.57216	-0.26714
H	-0.27075	-2.50145	0.24018
H	0.45880	-1.28750	1.33166

CAAC_oxo_t (*NImag* = 0)

C	2.92817	-2.69897	1.73517
N	1.59262	-3.00889	1.12480
C	1.19053	-4.24780	1.19638
C	2.26120	-5.06937	1.88883
C	3.47596	-4.11581	1.97637
C	0.79717	-1.93636	0.54139
Pd	-0.62737	-4.99627	0.61318
O	-2.41067	-5.44978	0.64619
C	2.57777	-6.32525	1.07144
C	1.73731	-5.48192	3.27495
C	3.79168	-1.90315	0.75995
C	2.72894	-1.91421	3.03479
H	3.99277	-4.18754	2.93262
H	3.37758	-6.89291	1.55236
H	1.69833	-6.96526	0.99190
H	0.83769	-6.08826	3.17625
H	2.50051	-6.06370	3.79614
H	1.48753	-4.61624	3.88876
H	2.90123	-6.06768	0.06141
H	4.19692	-4.36386	1.19638
H	3.86414	-2.41347	-0.20147
H	3.40110	-0.89847	0.59429
H	4.79803	-1.80345	1.16851
H	2.26870	-0.94254	2.85267
H	2.10320	-2.46414	3.73618
H	3.69697	-1.73698	3.50489
H	0.73201	-1.09773	1.23413
H	1.24838	-1.59153	-0.38904
H	-0.19794	-2.32427	0.34096

DAC_oxo_s (*NImag* = 0)

N	-0.86019	0.07690	-0.33305
C	-0.07518	-0.71569	-1.10229
C	0.03279	-1.59608	1.00830
C	-0.88171	-0.35654	0.99612
Pd	0.28930	-0.55306	-2.98389
O	-0.57218	0.85185	-3.67398
N	0.45356	-1.70342	-0.31650
O	0.32376	-2.30790	1.92452
O	-1.47972	0.13419	1.90717
C	1.34737	-2.74129	-0.80236

H	0.85089	-3.33703	-1.56880
H	2.25621	-2.29570	-1.20772
H	1.60338	-3.37897	0.04022
C	-1.60904	1.24678	-0.78716
H	-1.27279	2.11985	-0.23021
H	-1.42868	1.37544	-1.85526
H	-2.66794	1.08706	-0.59029

DAC_oxo_t (*NImag* = 0)

N	-0.96080	0.07949	-0.18792
C	-0.12836	-0.60828	-1.02997
C	-0.04501	-1.66929	1.00695
C	-1.00157	-0.46576	1.09109
Pd	0.23696	-0.18326	-2.95389
O	0.57339	0.20827	-4.72612
N	0.40704	-1.64147	-0.30824
O	0.24564	-2.44969	1.86795
O	-1.63480	-0.08373	2.03338
C	1.34717	-2.60974	-0.84993
H	0.89346	-3.14784	-1.68096
H	2.24834	-2.10450	-1.19433
H	1.59917	-3.30764	-0.05532
C	-1.72367	1.25392	-0.57981
H	-1.05080	2.04666	-0.90357
H	-2.40577	1.00384	-1.39111
H	-2.29055	1.58542	0.28663

Isonitrile_oxo_s (*NImag* = 0)

C	-2.37162	0.41169	0.00001
C	-0.98540	0.39687	0.00072
C	-0.29263	1.60763	0.00028
C	-0.97522	2.82447	-0.00085
C	-2.36142	2.82065	-0.00155
C	-3.06182	1.61904	-0.00112
H	-2.91395	-0.52425	0.00034
H	-0.43394	-0.53277	0.00160
H	-0.41546	3.74908	-0.00117
H	-2.89600	3.76104	-0.00243
H	-4.14346	1.62358	-0.00167
N	1.08509	1.60319	0.00098
C	2.25203	1.62791	0.00153
Pd	4.24723	1.60945	0.00247
O	6.03318	1.63576	0.00318

Isonitrile_oxo_t (*NImag* = 0)

C	-2.36837	0.41114	0.00001
C	-0.98212	0.40175	0.00069
C	-0.29488	1.61558	0.00024
C	-0.98213	2.82939	-0.00087
C	-2.36838	2.81999	-0.00153
C	-3.06375	1.61556	-0.00110
H	-2.90673	-0.52709	0.00035
H	-0.42636	-0.52529	0.00155
H	-0.42639	3.75644	-0.00119
H	-2.90675	3.75822	-0.00240
H	-4.14540	1.61556	-0.00162
N	1.08312	1.61558	0.00091
C	2.24928	1.61558	0.00148
Pd	4.22397	1.61551	0.00243
O	6.08152	1.61540	0.00333

MIC_oxo_s (*NImag* = 0)

N	1.10514	1.05704	0.07930
C	0.43611	-0.16886	0.04683
C	2.42201	0.86717	0.10059
Pd	-1.52737	-0.31248	-0.03866
H	3.17524	1.63409	0.12216
N	2.65755	-0.44813	0.08353
C	1.43656	-1.11579	0.04190
O	-2.07601	1.12573	-0.98052
C	3.97078	-1.06637	0.06925
H	4.73126	-0.29214	0.13564
H	4.07583	-1.74259	0.91674
H	4.11420	-1.62808	-0.85331
C	0.45215	2.36676	0.10471
H	-0.50672	2.26550	-0.41661
H	0.27495	2.66355	1.13847
H	1.09754	3.09403	-0.38527
C	1.34404	-2.59662	-0.02411
H	1.69741	-3.07968	0.89129
H	0.30096	-2.87177	-0.17067
H	1.91574	-3.00710	-0.86076

MIC_oxo_t (*NImag* = 0)

N	-1.02266	1.35684	0.00353
C	-0.38619	0.11188	-0.00973
C	-2.34789	1.22715	0.01397
Pd	1.67669	-0.18050	-0.02578
H	-3.07240	2.02176	0.02519
N	-2.63006	-0.07922	0.00819
C	-1.43079	-0.78755	-0.00652
O	3.49702	-0.32978	-0.03237
C	-3.96450	-0.65128	0.01702
H	-4.69972	0.14963	0.02899
H	-4.11649	-1.26190	-0.87220
H	-4.09997	-1.27323	0.90104
C	-0.31542	2.62883	0.00257
H	-1.03530	3.44529	0.03500
H	0.34209	2.67272	0.86785
H	0.29333	2.70065	-0.89611
C	-1.39519	-2.27248	-0.01575
H	-1.88600	-2.69208	-0.89848
H	-0.35386	-2.58750	-0.02490
H	-1.87367	-2.70282	0.86856

NHC_Me_oxo_s (*NImag* = 0)

N	-0.60648	0.39634	-0.39047
C	0.27489	-0.30366	-1.13827
N	0.66732	-1.33731	-0.35106
C	0.02639	-1.29049	0.87442
C	-0.76937	-0.19834	0.84806
C	1.58947	-2.37177	-0.77969
Pd	0.85987	0.06289	-2.97985
O	0.19554	1.66325	-3.45689
C	-1.28984	1.61575	-0.81292
H	-1.42448	0.21047	1.59556
H	0.19758	-2.02153	1.64359
H	-1.04712	2.42143	-0.12024
H	-0.94476	1.87208	-1.81909
H	-2.36648	1.44488	-0.81134
H	2.15579	-2.73410	0.07632
H	1.05668	-3.20380	-1.24138

H	2.28591	-1.94373	-1.50052

NHC_Me_oxo_t (*NImag* = 0)

C	-2.88353	0.67596	0.00748
N	-1.55695	1.06951	-0.00060
C	-0.71798	-0.00073	0.00486
N	-1.55823	-1.06991	0.01650
C	-2.88434	-0.67470	0.01823
C	-1.11339	2.45121	-0.01332
Pd	1.34556	-0.00195	-0.00254
O	3.20584	-0.00303	-0.00915
C	-1.11632	-2.45217	0.02590
H	-3.69675	1.37911	0.00485
H	-3.69840	-1.37683	0.02685
H	-1.47875	2.95991	-0.90585
H	-1.47173	2.97406	0.87385
H	-0.02608	2.45801	-0.01767
H	-1.48202	-2.97458	-0.85851
H	-0.02902	-2.46033	0.02203
H	-1.47554	-2.96040	0.92119

NHCS_oxo_s (*NImag* = 0)

N	-0.81588	0.08204	-0.37389
C	0.02052	-0.64443	-1.10993
C	-0.15617	-1.73322	0.93780
C	-1.19593	-0.61203	0.86122
Pd	0.73950	-0.19366	-2.87534
O	-0.05760	1.29807	-3.47568
H	0.66243	-1.48884	1.62434
H	-1.16099	0.06564	1.71381
N	0.33473	-1.77211	-0.44106
H	-2.21595	-0.99896	0.76974
H	-0.57925	-2.69332	1.23332
C	1.40413	-2.65868	-0.83094
H	1.52618	-2.62020	-1.91465
H	2.35365	-2.38726	-0.35780
H	1.15468	-3.68516	-0.55807
C	-1.51554	1.27971	-0.79646
H	-1.10611	1.60850	-1.75532
H	-2.58268	1.06673	-0.91066
H	-1.39230	2.05856	-0.04091

NHCS_oxo_t (*NImag* = 0)

N	-0.89858	0.11021	-0.25030
C	-0.12114	-0.59782	-1.07513
C	0.00191	-1.62527	1.02308
C	-1.04396	-0.50590	1.07270
Pd	0.25948	-0.15273	-3.09570
O	0.59208	0.23499	-4.84909
H	0.87980	-1.40512	1.63844
H	-0.85611	0.22063	1.86434
N	0.37301	-1.63724	-0.39580
H	-2.06139	-0.88926	1.19804
H	-0.39313	-2.59416	1.33135
C	1.35207	-2.57605	-0.89041
H	2.30947	-2.44551	-0.37652
H	1.00893	-3.60136	-0.73254
H	1.49339	-2.40336	-1.95486
C	-1.71340	1.24107	-0.62681
H	-1.46216	1.52751	-1.64548
H	-2.77676	0.98675	-0.57854
H	-1.52609	2.08400	0.04276

PMe3_oxo_s (*NImag* = 0)

P	-0.40558	1.11451	0.00048
Pd	-2.76100	1.11545	-0.00080
C	0.40411	1.93822	-1.42332
H	1.49237	1.89355	-1.34800
H	0.08909	1.45405	-2.34779
H	0.09037	2.98139	-1.46401
C	0.40440	-0.53021	-0.00133
H	1.49267	-0.44253	-0.00076
H	0.08970	-1.08915	0.88005
H	0.09048	-1.08669	-0.88453
C	0.40431	1.93564	1.42558
H	1.49255	1.89128	1.34959
H	0.09037	2.97867	1.46784
H	0.08978	1.44995	2.34940
O	-4.54049	1.11686	-0.00241

PMe3_t (*NImag* = 0)

P	-0.39182	1.21329	0.00057
Pd	-2.71678	1.28028	0.00309
C	0.45928	1.97935	-1.42792
H	1.54144	1.85110	-1.35893
H	0.09967	1.52154	-2.34898
H	0.22536	3.04314	-1.46374
C	0.25535	-0.49537	-0.00611
H	1.34741	-0.50758	-0.00711
H	-0.11358	-1.02116	0.87385
H	-0.11510	-1.01476	-0.88922
C	0.46177	1.96923	1.43297
H	1.54380	1.84137	1.36130
H	0.22801	3.03277	1.47660
H	0.10363	1.50504	2.35140
O	-4.40529	0.52279	0.00223

pyridine_oxo_s (*NImag* = 0)

C	-5.16836	0.85708	-0.00953
C	-3.34675	2.29087	0.01776
C	-4.16853	3.40349	0.00655
C	-5.54474	3.22124	-0.01346
C	-6.04604	1.92708	-0.02152
H	-5.49097	-0.17547	-0.01480
H	-2.26970	2.39070	0.03347
H	-3.72972	4.39120	0.01350
H	-6.21239	4.07239	-0.02259
H	-7.10983	1.73627	-0.03696
N	-3.83542	1.04254	0.00991
Pd	-2.68189	-0.64252	0.02557
O	-3.88455	-1.97130	0.00843

pyridine_oxo_t (*NImag* = 0)

C	-5.47626	0.99224	-0.00361
C	-3.48226	2.14358	0.00345
C	-4.13822	3.36218	0.00214
C	-5.52629	3.38126	-0.00224
C	-6.20369	2.16952	-0.00515
H	-5.97062	0.03041	-0.00581
H	-2.40212	2.09091	0.00686
H	-3.56289	4.27727	0.00454
H	-6.06718	4.31797	-0.00335

H	-7.28385	2.12874	-0.00859
N	-4.13283	0.96804	0.00063
Pd	-3.07874	-0.86285	0.00278
O	-2.16393	-2.45569	0.00466

amine_CO (*NImag* = 0)

C	-4.03550	0.17986	0.00808
N	-2.56237	0.14599	-0.01474
Pd	-1.77394	1.08666	1.80781
C	-1.11274	1.86864	3.33207
C	-2.10323	-1.25418	-0.06947
C	-2.07162	0.87102	-1.19987
H	-4.37650	1.21188	0.05477
H	-4.44798	-0.29552	-0.89295
H	-4.39887	-0.34724	0.88763
H	-2.48401	-1.75187	-0.97247
H	-1.01573	-1.28160	-0.07924
H	-2.45801	-1.78876	0.80902
H	-2.40333	1.90627	-1.15677
H	-0.98391	0.85420	-1.21298
H	-2.45082	0.40865	-2.12199
O	-0.69860	2.35479	4.28077

benzimi_CO (*NImag* = 0)

C	-0.54563	4.67148	-0.44101
C	-1.10534	3.47942	-0.88648
C	-0.53103	2.30168	-0.42910
C	0.56441	2.31310	0.44306
C	1.12376	3.50266	0.88824
C	0.54907	4.68289	0.43055
N	-0.82086	0.96499	-0.65968
C	0.03031	0.14913	0.01809
N	0.87110	0.98261	0.68735
Pd	0.04369	-1.93182	0.02908
H	-1.95113	3.47360	-1.55989
H	0.95679	5.63072	0.75503
H	-0.96529	5.61069	-0.77523
H	1.96953	3.51445	1.56160
C	1.95019	0.53724	1.54651
H	1.79903	0.90233	2.56345
H	1.94881	-0.55022	1.54518
H	2.90738	0.90260	1.17150
C	-1.89412	0.49718	-1.51421
H	-1.74743	0.85363	-2.53487
H	-1.87901	-0.59011	-1.50162
H	-2.85589	0.85431	-1.14303
C	0.05574	-3.80851	0.03915
O	0.06304	-4.95013	0.04537

CAAC_CO (*NImag* = 0)

C	2.58540	-0.79776	-0.11127
N	1.58743	0.32310	-0.06573
C	0.32669	-0.00795	-0.07503
C	0.22780	-1.51902	-0.15842
C	1.67297	-1.98889	-0.44720
Pd	-1.30570	1.27181	0.06707
C	-0.73974	-1.92863	-1.27227
C	-0.30107	-2.03477	1.19032
C	3.63055	-0.54793	-1.19590
C	3.25966	-0.93707	1.25626
H	1.94616	-2.87522	0.12455
H	-0.78013	-3.01781	-1.35095
H	-1.74200	-1.55300	-1.06598
H	-1.28303	-1.61016	1.39830
H	-0.38621	-3.12309	1.15970
H	0.35931	-1.76603	2.01551
H	-0.42324	-1.52603	-2.23589
H	1.77558	-2.23893	-1.50388
H	3.15333	-0.34971	-2.15659
H	4.28455	0.28956	-0.95037
H	4.25631	-1.43482	-1.30384
H	3.83828	-0.04862	1.51172
H	2.52451	-1.10574	2.04197
H	3.94569	-1.78484	1.24034
C	2.05281	1.70059	0.03499
H	2.59911	1.98280	-0.86500
H	1.18033	2.33855	0.14795
H	2.70879	1.81727	0.89794
C	-2.78414	2.42068	0.23696
O	-3.67765	3.12294	0.34804

DAC_CO (*NImag* = 0)

N	-1.31879	1.06784	0.01735
C	-0.54786	-0.05684	-0.06390
N	-1.38985	-1.13054	-0.00356
C	-2.72275	-0.75981	0.14454
C	-2.67306	0.78195	0.16043
C	-0.95999	-2.51993	-0.05292
Pd	1.46719	-0.12044	-0.38009
C	3.35038	-0.17986	-0.67557
O	4.49097	-0.20916	-0.84964
O	-3.58169	1.55549	0.26063
O	-3.67897	-1.47583	0.22889
C	-0.79692	2.42592	-0.02479
H	-0.55449	2.77366	0.97920
H	0.10256	2.42867	-0.63503
H	-1.55746	3.07360	-0.45503
H	0.00727	-2.55640	-0.54765
H	-0.87131	-2.92761	0.95397
H	-1.69818	-3.09547	-0.60735

Isonitrile_CO (*NImag* = 0)

C	-2.34831	0.41085	0.00000
C	-0.96198	0.40187	0.00071
C	-0.27533	1.61558	0.00028
C	-0.96198	2.82930	-0.00085
C	-2.34832	2.82031	-0.00154
C	-3.04318	1.61558	-0.00112
H	-2.88706	-0.52717	0.00033
H	-0.40500	-0.52441	0.00158
H	-0.40501	3.75557	-0.00116
H	-2.88706	3.75833	-0.00241
H	-4.12492	1.61558	-0.00167
N	1.10346	1.61558	0.00098
C	2.26859	1.61558	0.00156
Pd	4.25496	1.61556	0.00256
C	6.16169	1.61554	0.00353
O	7.29862	1.61553	0.00410

MIC_CO (*NImag* = 0)

N	1.33563	1.34011	-0.02731
C	0.64826	0.12248	0.01693

C	2.65608	1.16070	-0.05433
Pd	-1.43127	-0.08816	0.06882
H	3.41103	1.92568	-0.08978
N	2.88633	-0.15530	-0.02989
C	1.65927	-0.81516	0.01432
C	4.19637	-0.77934	-0.04990
H	4.96288	-0.00879	-0.08389
H	4.33807	-1.38376	0.84543
H	4.29464	-1.41880	-0.92654
C	0.67804	2.63712	-0.03327
H	-0.01894	2.67792	-0.86723
H	0.11598	2.76074	0.89020
H	1.42421	3.42523	-0.12658
C	1.56622	-2.29733	0.04938
H	2.05189	-2.72323	0.93226
H	0.51235	-2.56663	0.07842
H	2.01337	-2.76266	-0.83394
C	-3.28660	-0.25146	0.11166
O	-4.42802	-0.34207	0.13748

NHC_CO (*NImag* = 0)

N	-0.61433	0.87821	-0.59193
C	-1.15965	-0.35024	-0.40044
N	-2.49343	-0.13891	-0.54050
C	-1.58297	1.83041	-0.84476
C	-2.77211	1.18672	-0.81222
C	-3.49740	-1.18153	-0.41862
H	-4.05478	-1.28027	-1.35053
H	-2.98106	-2.11400	-0.20293
H	-4.18637	-0.94952	0.39398
C	0.81128	1.15089	-0.53656
H	1.02756	1.87286	0.25135
H	1.31897	0.21369	-0.32077
H	1.15869	1.54205	-1.49321
H	-3.77279	1.55253	-0.95644
H	-1.34883	2.86450	-1.02305
Pd	-0.17735	-2.14438	0.01331
C	0.70355	-3.75377	0.38495
O	1.24103	-4.73609	0.61195

NHCS_CO (*NImag* = 0)

N	1.09210	1.57490	0.02033
C	0.09175	0.69477	-0.11144
N	-1.05630	1.38258	-0.15331
C	-0.87679	2.82170	0.05929
C	0.64566	2.97016	-0.02860
Pd	0.28776	-1.38780	-0.23129
H	0.96605	3.43565	-0.96650
H	-1.40773	3.39966	-0.69853
H	-1.26809	3.10933	1.04069
H	1.06791	3.54651	0.79588
C	2.49717	1.25159	-0.02912
H	2.60158	0.16895	-0.00650
H	2.95461	1.63650	-0.94643
H	3.01939	1.68519	0.82762
C	-2.37601	0.79983	-0.15434
H	-2.88554	0.98464	0.79705
H	-2.98041	1.22435	-0.96006
H	-2.27756	-0.27340	-0.30271
C	0.46296	-3.24989	-0.33864
O	0.56975	-4.38528	-0.40419

PMe3_CO (*NImag* = 0)

P	-0.41140	1.11649	0.00078
Pd	-2.74541	1.11737	0.00136
C	0.39289	1.93910	-1.42548
H	1.48174	1.89403	-1.35123
H	0.07398	1.45438	-2.34787
H	0.07717	2.98129	-1.46557
C	0.39134	-0.53082	0.00006
H	1.48026	-0.44468	-0.00080
H	0.07439	-1.08650	0.88224
H	0.07279	-1.08687	-0.88134
C	0.39547	1.93766	1.42633
H	1.48412	1.89386	1.34898
H	0.07877	2.97944	1.46961
H	0.07966	1.45066	2.34862
C	-4.64436	1.11207	-0.00154
O	-5.78339	1.10839	-0.00415

pyridine_CO (*NImag* = 0)

C	-5.46424	0.96972	-0.00039
C	-3.47009	2.12124	0.00006
C	-4.12605	3.34127	-0.00072
C	-5.51366	3.35776	-0.00136
C	-6.19302	2.14771	-0.00119
H	-5.95472	0.00672	-0.00024
H	-2.39086	2.06466	0.00057
H	-3.55225	4.25738	-0.00082
H	-6.05475	4.29479	-0.00197
H	-7.27328	2.10868	-0.00167
N	-4.12292	0.94933	0.00022
Pd	-3.05804	-0.89493	0.00139
C	-2.13692	-2.49038	0.00251
O	-1.56498	-3.48010	0.00321

benzimi_Cl2 (*NImag* = 0)

N	0.99660	0.92109	0.70010
C	0.66573	2.24216	0.41591
C	-0.50126	2.19959	-0.35373
N	-0.82761	0.85434	-0.49696
C	0.08887	0.09768	0.13937
C	1.27019	3.44348	0.75459
C	0.66331	4.60438	0.29107
C	-0.50328	4.56195	-0.48080
C	-1.10780	3.35710	-0.81817
C	-1.97858	0.36344	-1.23605
Pd	0.11202	-1.80932	0.21274
Cl	1.95507	-1.92946	-1.13761
C	2.15821	0.51712	1.47389
Cl	-1.72756	-1.90401	1.56912
H	-2.00668	3.32695	-1.41739
H	1.10332	5.56250	0.53163
H	-0.94405	5.48802	-0.82368
H	2.17182	3.47831	1.34955
H	2.10208	0.95308	2.47122
H	2.16900	-0.56508	1.54263
H	3.06637	0.84888	0.97221
H	-1.82721	0.51414	-2.30506
H	-2.10260	-0.69233	-1.02049
H	-2.86925	0.89806	-0.90918

benzimi_Cl2_CO (*NImag* = 0)

C	4.69152	0.55550	0.42403
C	3.50700	1.13307	0.86461
C	2.32514	0.55541	0.42447
C	2.32474	-0.55623	-0.42445
C	3.50618	-1.13479	-0.86453
C	4.69112	-0.55810	-0.42391
N	0.98974	0.86220	0.66061
C	0.19100	0.00038	-0.00002
N	0.98912	-0.86201	-0.66065
Pd	-1.81661	0.00108	-0.00002
Cl	-1.74331	-1.93077	1.29996
Cl	-1.74200	1.93278	-1.30013
H	3.51010	1.99357	1.51870
H	5.63459	-0.97928	-0.74338
H	5.63530	0.97597	0.74353
H	3.50865	-1.99528	-1.51864
C	0.53939	-1.96904	-1.48537
H	0.86090	-2.91303	-1.04641
H	-0.54418	-1.95322	-1.52747
H	0.94890	-1.86794	-2.49046
C	0.54081	1.96957	1.48532
H	0.86291	2.91333	1.04631
H	-0.54277	1.95447	1.52751
H	0.95033	1.86822	2.49038
C	-3.78630	0.00177	-0.00003
O	-4.91195	0.00206	0.00007

CAAC_Cl2 (*NImag* = 0)

C	2.34874	-0.83828	-0.71844
N	1.31465	0.24929	-0.64612
C	0.29282	-0.02968	0.09242
C	0.42977	-1.37225	0.77046
C	1.61873	-1.99389	-0.00477
Pd	-1.20959	1.11007	0.25006
Cl	-0.45592	2.18353	2.12766
C	-0.84211	-2.21297	0.63888
C	0.75552	-1.15165	2.25851
C	2.64558	-1.16790	-2.17826
C	3.61153	-0.38529	0.01243
H	2.28986	-2.54100	0.65524
H	-0.65668	-3.20823	1.04749
H	-1.66372	-1.75920	1.19171
H	-0.06069	-0.63880	2.76259
H	0.91308	-2.12393	2.72840
H	1.65207	-0.54869	2.39681
H	-1.15102	-2.30505	-0.40131
H	1.23714	-2.69406	-0.74756
H	1.72605	-1.39201	-2.71939
H	3.16127	-0.35236	-2.68601
H	3.29139	-2.04586	-2.22012
H	4.05419	0.49312	-0.45841
H	3.40225	-0.14696	1.05407
H	4.35202	-1.18527	-0.01688
C	1.46350	1.49015	-1.40369
H	0.89931	1.41635	-2.33241
H	1.07420	2.31370	-0.81196
H	2.51564	1.65705	-1.61754
Cl	-2.04485	0.26940	-1.72612

CAAC_Cl2_CO (*NImag* = 0)

C	-2.74084	-0.12349	0.39406
N	-1.29259	-0.09857	0.79318
C	-0.45305	0.05754	-0.17327
C	-1.17115	0.26438	-1.48571
C	-2.62328	-0.14898	-1.14126
Pd	1.52159	-0.03734	0.07013
Cl	1.54995	2.26839	0.43174
C	-0.58090	-0.59328	-2.60726
C	-1.06841	1.75211	-1.87125
C	-3.40098	-1.38748	0.93762
C	-3.43620	1.13094	0.92165
Cl	1.23740	-2.35579	-0.09085
H	-3.35576	0.50728	-1.60864
H	-1.18343	-0.47023	-3.50948
H	0.44061	-0.28714	-2.83188
H	-0.03171	2.04052	-2.02959
H	-1.62980	1.91480	-2.79318
H	-1.47087	2.40444	-1.09741
H	-0.55916	-1.64649	-2.33255
H	-2.80650	-1.16144	-1.50123
H	-2.84093	-2.27418	0.63921
H	-3.48064	-1.37307	2.02514
H	-4.41057	-1.46622	0.53249
H	-3.42998	1.16518	2.01169
H	-2.95785	2.03514	0.54825
H	-4.47713	1.13137	0.59671
C	-0.89570	-0.29057	2.18634
H	-0.64273	-1.33726	2.35100
H	-0.02773	0.32901	2.39293
H	-1.71283	0.00021	2.84099
C	3.49461	-0.17407	0.27431
O	4.61167	-0.24929	0.39432

DAC_Cl2 (*NImag* = 0)

N	-1.16603	-0.20900	-0.27772
C	-0.09911	-0.64361	-0.97761
N	0.79309	-1.24523	-0.16583
C	0.34064	-1.23629	1.16366
C	-1.02470	-0.51542	1.08555
C	2.06818	-1.81624	-0.59013
Pd	0.11465	-0.43900	-2.82894
O	-1.78850	-0.27030	1.96593
O	0.89250	-1.68385	2.11954
C	-2.32637	0.47184	-0.84510
Cl	1.51204	1.35152	-2.55838
Cl	-1.26272	-2.21021	-3.27326
H	-2.01159	1.40833	-1.30049
H	-2.78773	-0.16954	-1.59246
H	-3.01697	0.66519	-0.02875
H	1.88761	-2.62558	-1.29428
H	2.66902	-1.04073	-1.05953
H	2.56246	-2.19647	0.29974

DAC_Cl2_CO (*NImag* = 0)

N	1.57040	0.92315	0.60880
C	0.79895	-0.00001	0.00085
C	2.93219	-0.64581	-0.42413
C	2.93171	0.64854	0.42399
N	1.57111	-0.92229	-0.60753
O	3.86421	-1.26841	-0.82983
O	3.86324	1.27249	0.82873
C	1.06316	-2.07328	-1.34596

H	0.46439	-2.69147	-0.68043
H	0.45770	-1.72882	-2.18177
H	1.92142	-2.63132	-1.71048
C	1.06186	2.07452	1.34630
H	0.46010	2.68998	0.68092
H	0.45921	1.73058	2.18437
H	1.91970	2.63526	1.70764
Pd	-1.18114	-0.00120	0.00026
Cl	-1.01977	1.49357	-1.77771
Cl	-1.02305	-1.49759	1.77716
C	-3.16378	-0.00202	-0.00073
O	-4.28798	-0.00251	-0.00090

Isonitrile_Cl2 (*NImag* = 0)

C	-0.95857	2.76671	-0.43050
C	-2.34397	2.77459	-0.38711
C	-3.03756	1.66396	0.08249
C	-2.34667	0.53598	0.51297
C	-0.96120	0.50885	0.47793
C	-0.28166	1.62917	0.00447
N	1.09902	1.61249	-0.03531
C	2.25351	1.60507	-0.07181
Pd	4.10000	1.59838	-0.13289
Cl	4.27506	-0.44717	0.84363
Cl	4.20941	3.64416	-1.11960
H	-2.88725	-0.32676	0.87770
H	-0.40288	-0.35597	0.80751
H	-0.39795	3.61726	-0.79178
H	-2.88237	3.65096	-0.72133
H	-4.11876	1.67753	0.11296

Isonitrile_Cl2_CO (*NImag* = 0)

C	-4.93564	1.09870	0.50549
C	-3.54956	1.10416	0.51571
C	-2.87212	0.00272	-0.00275
C	-3.55129	-1.09499	-0.52683
C	-4.93736	-1.08193	-0.52805
C	-5.62881	0.01028	-0.01413
H	-5.47499	1.94690	0.90454
H	-2.98847	1.93772	0.91404
H	-2.99152	-1.93162	-0.92056
H	-5.47803	-1.92716	-0.93158
H	-6.71055	0.01324	-0.01858
N	-1.49069	-0.00112	0.00295
C	-0.33659	-0.00474	0.00747
Pd	1.63056	-0.01115	0.01478
Cl	1.61514	2.07119	1.04128
Cl	1.60827	-2.09337	-1.01176
C	3.57970	-0.01724	0.02191
O	4.70347	-0.02060	0.02605

MIC_Cl2 (*NImag* = 0)

C	0.51027	-0.28453	-1.31822
C	-0.03167	-0.46060	-0.07572
N	0.52930	0.53271	0.71676
C	1.36582	1.27479	-0.01500
N	1.36948	0.79787	-1.25965
C	0.27787	0.71900	2.14230
Pd	-1.28811	-1.77122	0.53585
Cl	-2.87955	-0.21270	1.12075
C	2.14768	1.32280	-2.37032
Cl	0.13293	-3.48570	0.02412
H	1.93848	2.11345	0.33672
H	0.37197	-0.85339	-2.21792
H	2.75394	2.15491	-2.02094
H	2.79911	0.54330	-2.76233
H	1.48098	1.66915	-3.15861
H	-0.79567	0.79657	2.29640
H	0.65941	-0.14223	2.68728
H	0.78338	1.62383	2.47276

MIC_Cl2_CO (*NImag* = 0)

C	1.72386	-0.56974	-0.50507
C	0.71058	0.12394	0.10534
N	1.33959	1.08884	0.88005
C	2.66332	0.99749	0.75207
N	2.92685	-0.00413	-0.09080
C	0.65176	2.05307	1.72940
Pd	-1.28176	-0.10221	-0.04772
C	4.25290	-0.43212	-0.49939
Cl	-1.06866	-2.18232	0.98536
Cl	-1.39712	1.99923	-1.07716
H	3.39439	1.61444	1.24199
C	1.66806	-1.71914	-1.44161
H	4.99604	0.20207	-0.02244
H	4.42073	-1.46623	-0.20155
H	4.35497	-0.35181	-1.58075
H	-0.04564	2.62037	1.11727
H	0.10248	1.52026	2.50344
H	1.38669	2.71302	2.18556
H	2.26986	-2.55955	-1.08790
H	0.63812	-2.05893	-1.51131
H	2.01520	-1.44854	-2.44261
C	-3.22947	-0.34223	-0.21272
O	-4.34440	-0.48020	-0.30403

NHC_Cl2 (*NImag* = 0)

C	-0.45330	2.14571	-0.45405
C	0.74050	2.20319	0.17946
N	-0.83262	0.81535	-0.47703
C	0.10652	0.06424	0.12410
N	1.07365	0.90631	0.52787
Pd	0.07328	-1.83157	0.35871
Cl	-1.62882	-1.70995	1.88607
C	2.27814	0.50548	1.24579
H	2.02414	0.21621	2.26425
H	2.73449	-0.33475	0.72825
H	2.96405	1.34874	1.26297
C	-2.05347	0.29352	-1.08042
H	-1.81425	-0.24379	-1.99668
H	-2.53610	-0.37902	-0.37555
H	-2.70999	1.13033	-1.30527
Cl	1.77215	-2.14433	-1.14493
H	-1.06060	2.91931	-0.88604
H	1.37613	3.03727	0.41220

NHC_Cl2_CO (*NImag* = 0)

N	-1.78891	0.62860	-0.87005
C	-0.97803	-0.00003	-0.00011
N	-1.78918	-0.62874	0.86954
C	-3.11346	0.39445	-0.55005
C	-3.11363	-0.39442	0.54925

C	-1.32363	-1.42121	2.00040
H	-0.54807	-2.10239	1.65848
H	-0.92553	-0.76765	2.77533
H	-2.16256	-1.98842	2.39617
C	-1.32295	1.42111	-2.00073
H	-0.54824	2.10301	-1.65832
H	-0.92363	0.76762	-2.77508
H	-2.16198	1.98749	-2.39745
H	-3.92395	-0.80314	1.12411
H	-3.92360	0.80319	-1.12516
Pd	1.02936	0.00006	0.00027
Cl	0.95764	2.22520	0.69416
Cl	0.95844	-2.22512	-0.69359
C	2.99803	0.00008	0.00070
O	4.12394	0.00006	0.00097

NHCS_Cl2 (*NImag* = 0)

N	0.78468	1.43334	0.83298
C	0.01042	0.61982	0.12338
N	-0.77595	1.30595	-0.69858
C	-0.44370	2.73454	-0.66719
C	0.42939	2.83575	0.58959
Pd	0.02580	-1.28318	0.26601
Cl	-1.21222	-1.21888	2.19238
H	1.32271	3.44148	0.44267
H	0.10393	3.00402	-1.57518
H	-1.34689	3.34083	-0.61102
H	-0.12365	3.22855	1.44797
C	1.61678	1.06006	1.95685
H	1.81133	-0.00702	1.91417
H	2.56055	1.60417	1.90325
H	1.11551	1.28763	2.90056
C	-1.60063	0.75603	-1.75325
H	-2.55315	1.28672	-1.78006
H	-1.10203	0.84858	-2.72096
H	-1.77790	-0.29581	-1.55184
Cl	1.26497	-1.48693	-1.64988

NHCS_Cl2_CO (*NImag* = 0)

N	1.69252	0.52227	-0.95652
C	0.93265	0.00028	-0.00002
N	1.69284	-0.52104	0.95660
C	3.11392	-0.48313	0.59392
C	3.11359	0.48551	-0.59369
Pd	-1.07300	-0.00058	-0.00014
Cl	-0.96023	-2.20577	-0.74652
Cl	-0.96199	2.20468	0.74632
H	3.44716	1.48861	-0.31058
H	3.72031	-0.13686	1.43026
H	3.44833	-1.48596	0.31085
H	3.72035	0.13972	-1.42996
C	1.22804	1.34817	-2.04875
H	0.16768	1.17198	-2.20227
H	1.37767	2.40757	-1.82562
H	1.77062	1.08751	-2.95864
C	1.22889	-1.34732	2.04877
H	1.37911	-2.40661	1.82553
H	1.77136	-1.08644	2.95865
H	0.16843	-1.17175	2.20237
C	-3.04634	-0.00150	-0.00031
O	-4.17230	-0.00205	-0.00041

pyridine_Cl2 (*NImag* = 0)

C	-5.57356	1.00350	-0.13137
C	-3.52524	2.05842	0.07392
C	-4.11876	3.30728	0.08599
C	-5.50092	3.39149	-0.01931
C	-6.23532	2.22011	-0.12985
H	-6.09682	0.06117	-0.19009
H	-2.45214	1.94475	0.15998
H	-3.50370	4.19058	0.18138
H	-5.99517	4.35375	-0.01170
H	-7.31280	2.23710	-0.21138
N	-4.23916	0.92892	-0.03728
Pd	-3.20542	-0.85058	-0.12627
Cl	-4.78989	-1.88785	1.09644
Cl	-1.84677	-2.59943	-0.41114

pyridine_Cl2_CO (*NImag* = 0)

C	1.73269	0.97042	-0.63138
C	1.73260	-0.97065	0.63143
C	3.11572	-0.99596	0.65891
C	3.82208	-0.00022	0.00002
C	3.11581	0.99557	-0.65889
H	1.13730	1.73986	-1.09854
H	1.13713	-1.74005	1.09856
H	3.62098	-1.79197	1.18691
H	4.90409	-0.00029	0.00000
H	3.62114	1.79151	-1.18693
N	1.05651	-0.00006	0.00006
Pd	-1.03192	0.00004	0.00021
Cl	-1.09518	2.31471	0.18097
Cl	-1.09524	-2.31460	-0.18069
C	-2.91743	-0.00007	0.00040
O	-4.04354	0.00036	0.00056

NMe3_Cl2 (*NImag* = 0)

C	-2.00901	1.21788	0.04197
H	-1.77216	1.83030	-0.82078
H	-3.04062	0.85709	-0.01544
H	-1.87812	1.79848	0.95119
C	-1.46062	-0.80663	1.24374
H	-2.49784	-1.13078	1.11661
H	-0.80084	-1.66579	1.28750
H	-1.35443	-0.23641	2.15968
C	-1.24398	-0.75752	-1.16336
H	-1.01039	-0.13747	-2.02155
H	-0.56092	-1.60178	-1.12390
H	-2.27360	-1.12348	-1.22357
N	-1.09772	0.04372	0.08050
Pd	0.83577	0.73329	0.17774
Cl	1.49032	-0.21532	2.14844
Cl	0.69741	1.88802	-1.80303

NMe3_Cl2_CO (*NImag* = 0)

C	-2.06948	-0.56268	-1.21487
H	-1.48536	-0.81827	-2.09663
H	-3.00171	-1.13649	-1.22317
H	-2.28804	0.50033	-1.22178
C	-2.07492	-0.55766	1.20938
H	-3.00718	-1.13145	1.21586
H	-1.49477	-0.80955	2.09480

H	-2.29351	0.50537	1.21089
C	-1.03034	-2.35717	0.00334
H	-0.45475	-2.63208	-0.87472
H	-0.45862	-2.62839	0.88507
H	-1.98811	-2.88653	0.00235
N	-1.28960	-0.90032	-0.00027
Pd	0.49955	0.31455	0.00087
Cl	-0.75716	2.28179	-0.00546
Cl	1.96729	-1.48667	0.00713
C	2.03963	1.40177	0.00162
O	2.95932	2.05148	0.00207

NMe3_pdIV_oxo_s (*NImag* = 0)

C	-1.77549	1.10102	0.39708
N	-1.15034	-0.10171	-0.18907
Pd	1.09106	-0.02779	-0.20204
Cl	0.86402	1.82764	-1.52984
C	-1.46130	-1.28902	0.63462
C	-1.63051	-0.31550	-1.56913
O	2.58638	-0.90877	-0.04094
Cl	1.11465	0.32237	2.09050
H	-1.54913	1.96127	-0.22503
H	-2.85923	0.95658	0.45166
H	-1.37252	1.25984	1.39304
H	-2.54220	-1.46191	0.63463
H	-0.96305	-2.16316	0.21859
H	-1.11176	-1.12641	1.64919
H	-1.38500	0.54932	-2.17687
H	-1.15058	-1.19856	-1.98836
H	-2.71400	-0.47041	-1.56267

NMe3_pdIV_oxo_t (*NImag* = 0)

C	-1.82127	1.21019	0.32575
H	-1.58648	1.95543	-0.43163
H	-2.90315	1.04792	0.34520
H	-1.47767	1.55738	1.29443
C	-1.43730	-1.08974	1.01481
H	-2.51595	-1.26865	1.04679
H	-0.92949	-2.01589	0.75279
H	-1.08669	-0.74493	1.98177
C	-1.68216	-0.54266	-1.31286
H	-1.48686	0.18910	-2.08922
H	-1.20567	-1.47632	-1.59285
H	-2.76024	-0.69179	-1.20206
N	-1.14013	-0.06460	-0.01789
Pd	1.00720	0.30154	0.00213
O	2.77329	0.60267	0.01859
Cl	0.86818	0.94492	2.24932
Cl	1.24158	-0.31578	-2.23450

PMe3_Cl2 (*NImag* = 0)

Pd	-2.12657	-1.79741	-1.14104
P	-4.03700	-1.38228	-2.10268
C	-5.26018	-2.66675	-1.73363
H	-6.20117	-2.43738	-2.23817
H	-4.87234	-3.62666	-2.06703
H	-5.42498	-2.70550	-0.65773
C	-3.87299	-1.31737	-3.90578
H	-4.84698	-1.12122	-4.35911
H	-3.18058	-0.52050	-4.17366
H	-3.47309	-2.26630	-4.25593
C	-4.81335	0.18275	-1.62610
H	-5.75805	0.29008	-2.16372
H	-4.98682	0.19141	-0.55245
H	-4.14671	1.00808	-1.86546
Cl	-2.00063	-3.77953	-2.29154
Cl	-2.02382	0.10612	0.10676

PMe3_Cl2_CO (*NImag* = 0)

P	-1.80337	-1.16109	-0.03758
C	-1.22700	-0.22938	1.41120
H	-1.64140	0.78016	1.40290
H	-1.53636	-0.75638	2.31146
H	-0.13898	-0.17351	1.39158
C	-3.61693	-1.07504	0.00688
H	-3.94786	-0.03574	0.04539
H	-4.01946	-1.54946	-0.88763
H	-3.97131	-1.61569	0.88213
C	-1.32006	-0.15274	-1.46592
H	-0.23485	-0.11389	-1.53394
H	-1.69292	-0.61145	-2.37935
H	-1.72625	0.85469	-1.35876
C	-0.44376	-5.23068	0.08314
O	-0.09725	-6.29974	0.12666
Pd	-1.05788	-3.33768	0.00010
Cl	-2.17443	-3.53883	2.03940
Cl	0.03371	-3.07336	-2.03346

PMe3_pdIV_oxo_s (*NImag* = 0)

C	-5.19787	-2.71063	0.90140
P	-4.68483	-1.18767	0.08704
C	-5.32606	0.21878	1.02208
Pd	-2.47588	-1.01816	-0.01954
Cl	-2.18105	-1.91950	2.07870
Cl	-2.55071	0.47459	-1.77123
O	-2.35573	-2.57739	-0.99985
C	-5.43287	-1.18671	-1.55193
H	-6.28641	-2.78790	0.85915
H	-4.73280	-3.54142	0.37337
H	-4.84904	-2.71062	1.93029
H	-6.50816	-1.34990	-1.45283
H	-5.22834	-0.24137	-2.04671
H	-4.97361	-1.98967	-2.12608
H	-6.41433	0.14986	1.08387
H	-4.90000	0.20637	2.02436
H	-5.04476	1.14212	0.51755

PMe3_pdIV_oxo_t (*NImag* = 0)

Pd	-2.38744	-1.21889	-0.02381
P	-4.74599	-1.25117	0.01536
C	-5.55298	-2.72563	0.69777
H	-6.63584	-2.59637	0.64990
H	-5.25557	-3.60414	0.12947
H	-5.23898	-2.87308	1.72871
C	-5.43586	-1.08342	-1.65606
H	-6.52596	-1.06258	-1.61138
H	-5.05762	-0.16867	-2.10599
H	-5.11685	-1.93307	-2.25932
C	-5.40843	0.12792	0.99363
H	-6.49925	0.11696	0.96641
H	-5.07341	0.02504	2.02551
H	-5.02887	1.06365	0.59039

Cl	-2.68835	0.88827	-0.98420
Cl	-2.39375	-3.32575	0.93902
O	-0.54272	-1.10267	-0.09538

benzimi_pdIV_oxo_s (*NImag* = 0)

C	-0.70047	4.66453	0.13420
C	-1.42417	3.47838	0.12882
C	-0.69659	2.30122	0.03961
C	0.70018	2.30543	-0.03947
C	1.42391	3.48863	-0.03587
C	0.69687	4.66929	0.05342
N	-1.08231	0.96357	0.00624
C	0.00647	0.17854	-0.08315
N	1.09043	0.97166	-0.11052
Pd	-0.07111	-1.85276	-0.12680
Cl	0.41806	-1.49875	2.14465
O	-0.71766	-3.38516	0.49335
Cl	0.43597	-1.59746	-2.33938
H	-2.50318	3.47759	0.19241
H	1.22305	5.61403	0.06086
H	-1.22906	5.60543	0.20273
H	2.50280	3.49548	-0.09954
C	2.46748	0.51773	-0.18482
H	3.03540	0.95121	0.63738
H	2.48318	-0.56276	-0.09741
H	2.90597	0.81147	-1.13810
C	-2.45658	0.50170	0.09207
H	-3.03324	0.89598	-0.74428
H	-2.46604	-0.58365	0.05731
H	-2.89804	0.83008	1.03263

benzimi_pdIV_oxo_t (*NImag* = 0)

C	-0.60939	4.68876	-0.31676
C	-1.24359	3.50554	-0.67453
C	-0.60420	2.32304	-0.33265
C	0.62399	2.32103	0.33745
C	1.25738	3.50138	0.69744
C	0.61716	4.68671	0.35782
N	-0.94216	0.98744	-0.52486
C	0.01530	0.19209	-0.01394
N	0.96875	0.98437	0.50914
Pd	0.02054	-1.84044	-0.02958
Cl	-0.68342	-1.62826	2.17824
O	0.02532	-3.67696	-0.04369
Cl	0.72322	-1.59068	-2.23386
H	-2.18964	3.51043	-1.19714
H	1.07702	5.62954	0.62066
H	-1.07404	5.63317	-0.56512
H	2.20341	3.50307	1.22009
C	2.16439	0.52547	1.19305
H	2.11071	0.78212	2.25073
H	2.23188	-0.55281	1.09389
H	3.04306	0.98548	0.74206
C	-2.13549	0.53303	-1.21575
H	-2.08315	0.80611	-2.26938
H	-2.19752	-0.54698	-1.13313
H	-3.01646	0.98162	-0.75781

CAAC_pdIV_oxo_s (*NImag* = 0)

C	2.60498	-0.81217	-0.10268
N	1.66374	0.36590	-0.04974
C	0.42281	0.04032	-0.06586
C	0.18489	-1.43984	-0.13706
C	1.62330	-1.96524	-0.40144
Pd	-1.03127	1.32513	0.03753
Cl	-0.74963	1.48711	2.33364
C	-0.78201	-1.81292	-1.26603
C	-0.38830	-1.91457	1.21154
C	3.61750	-0.61268	-1.22399
C	3.28787	-0.94678	1.25629
Cl	-0.82602	1.67128	-2.24778
O	-2.67047	0.56035	0.06836
H	1.84522	-2.83830	0.20918
H	-0.85580	-2.90073	-1.31589
H	-1.76941	-1.39419	-1.07477
H	-1.37214	-1.47912	1.37719
H	-0.47916	-3.00122	1.18220
H	0.24996	-1.63617	2.04929
H	-0.43247	-1.43830	-2.22751
H	1.71307	-2.25772	-1.44672
H	3.11466	-0.41823	-2.17159
H	4.30611	0.20692	-1.01665
H	4.20998	-1.52198	-1.33096
H	3.90056	-0.07484	1.48760
H	2.55555	-1.07370	2.05235
H	3.94196	-1.81901	1.24411
C	2.14265	1.74562	0.05063
H	1.95226	2.25942	-0.89058
H	1.61646	2.24425	0.86336
H	3.20690	1.74384	0.26216

CAAC_pdIV_oxo_t (*NImag* = 0)

C	2.47570	-0.86996	-0.69625
N	1.46586	0.24468	-0.68376
C	0.38709	0.00373	-0.02237
C	0.44865	-1.34718	0.65112
C	1.66635	-2.00568	-0.04437
Pd	-1.18472	1.27574	0.04878
Cl	-0.05527	2.24702	1.83831
C	-0.83272	-2.16000	0.45072
C	0.68642	-1.14008	2.15942
C	2.86279	-1.19757	-2.13521
C	3.69708	-0.45531	0.12311
Cl	-1.73276	0.22103	-1.96397
O	-2.72296	2.29254	0.17352
H	2.27322	-2.57815	0.65516
H	-0.70156	-3.14901	0.89391
H	-1.68040	-1.67755	0.93714
H	-0.15611	-0.62922	2.62020
H	0.80763	-2.11772	2.62911
H	1.57491	-0.54305	2.35712
H	-1.07363	-2.27051	-0.60475
H	1.31396	-2.68900	-0.81696
H	1.97653	-1.39436	-2.73908
H	3.43255	-0.39276	-2.60055
H	3.48725	-2.09171	-2.14133
H	4.19803	0.40998	-0.31241
H	3.42207	-0.21123	1.14797
H	4.41416	-1.27655	0.14410
C	1.71001	1.48814	-1.41164
H	1.19008	1.45584	-2.36837
H	1.34038	2.32250	-0.82157

H	2.77667	1.60925	-1.57632

DAC_pdIV_oxo_s (*NImag* = 0)

N	-1.15298	-0.18095	-0.30513
C	-0.09736	-0.62294	-1.01592
C	0.34152	-1.23978	1.12111
C	-1.01051	-0.49139	1.05477
N	0.77973	-1.25372	-0.21141
O	0.89128	-1.70307	2.07132
O	-1.76389	-0.23529	1.94187
C	2.04341	-1.83439	-0.64890
H	1.85329	-2.59612	-1.40217
H	2.67861	-1.05212	-1.06004
H	2.51503	-2.28099	0.22218
C	-2.28312	0.55309	-0.86111
H	-1.92501	1.47290	-1.31920
H	-2.78749	-0.06311	-1.60259
H	-2.95869	0.78004	-0.04099
Pd	0.13549	-0.41895	-3.03329
Cl	-1.10751	-2.31770	-3.01179
Cl	1.10214	1.49695	-2.02020
O	0.75152	0.27788	-4.53443

DAC_pdIV_oxo_t (*NImag* = 0)

N	-0.90825	0.17213	-0.19733
C	-0.19635	-0.62462	-1.01657
C	-0.28148	-1.74362	0.95316
C	-1.04247	-0.40195	1.07478
N	0.17971	-1.74465	-0.37091
O	-0.11878	-2.58497	1.78114
O	-1.61181	0.04802	2.01991
C	0.96329	-2.82289	-0.96311
H	0.41663	-3.24986	-1.80161
H	1.92092	-2.43067	-1.29938
H	1.11584	-3.57530	-0.19416
C	-1.46969	1.46426	-0.57478
H	-0.66550	2.13888	-0.86177
H	-2.16100	1.32922	-1.40414
H	-1.99572	1.85662	0.29126
Pd	0.24435	-0.20031	-2.93929
Cl	-1.77519	-1.29909	-3.27950
Cl	2.11198	0.75217	-1.93628
O	0.64533	0.18591	-4.68959

Isonitrile_pdIV_oxo_s (*NImag* = 0)

C	4.88050	-0.60284	1.03321
C	3.49513	-0.56150	1.02594
C	2.84871	-0.03349	-0.08931
C	3.55545	0.45085	-1.18725
C	4.94049	0.40159	-1.16057
C	5.60202	-0.12338	-0.05528
H	5.39708	-1.00974	1.89172
H	2.91227	-0.92482	1.86052
H	3.01933	0.85556	-2.03398
H	5.50354	0.77480	-2.00503
H	6.68307	-0.15835	-0.04179
N	1.46643	0.01304	-0.10211
C	0.31454	0.04742	-0.08102
Pd	-1.68393	0.08557	-0.01102
Cl	-1.40103	-0.18754	2.25807
O	-1.50387	1.16170	-1.41981
Cl	-3.84321	-0.57819	0.11376

Isonitrile_pdIV_oxo_t (*NImag* = 0)

C	-2.31442	0.53635	0.53824
C	-0.92870	0.52444	0.53614
C	-0.25196	1.61959	0.00246
C	-0.93004	2.71709	-0.52467
C	-2.31574	2.70996	-0.51339
C	-3.00667	1.62434	0.01576
H	-2.85453	-0.30578	0.94875
H	-0.36710	-0.30813	0.93580
H	-0.36944	3.54773	-0.92972
H	-2.85689	3.55396	-0.91867
H	-4.08840	1.62621	0.02099
N	1.12816	1.61723	-0.00418
C	2.28142	1.61523	-0.00966
Pd	4.28025	1.61165	-0.01909
O	6.09342	1.60850	-0.02785
Cl	4.06525	-0.46959	0.98886
Cl	4.06374	3.69379	-1.02483

MIC_pdIV_oxo_s (*NImag* = 0)

C	0.25100	0.24892	-1.47910
C	-0.13738	-0.17399	-0.23812
N	0.79916	0.31412	0.65438
C	1.72543	1.01004	-0.00472
N	1.41839	0.98791	-1.30421
C	0.77891	0.09136	2.09606
Pd	-1.70297	-1.35748	0.17442
O	-2.57876	-2.65923	-0.66160
C	2.18340	1.62530	-2.36271
Cl	-0.12604	-3.01144	0.62166
Cl	-2.61416	0.52098	1.20182
H	2.57393	1.50524	0.43136
C	-0.36153	0.01049	-2.80955
H	3.03833	2.13569	-1.92656
H	2.53582	0.87669	-3.07079
H	1.56228	2.34999	-2.88701
H	-0.13844	0.51101	2.50213
H	0.79996	-0.98086	2.27895
H	1.64799	0.57229	2.53862
H	-0.68800	0.94078	-3.28083
H	0.33123	-0.49104	-3.48940
H	-1.23096	-0.63310	-2.68931

MIC_pdIV_oxo_t (*NImag* = 0)

C	0.33574	0.20447	-1.49410
C	-0.04420	-0.28164	-0.26937
N	0.80273	0.32141	0.64850
C	1.65395	1.13611	0.02630
N	1.38881	1.08887	-1.28198
C	0.77502	0.08665	2.08704
Pd	-1.53578	-1.57112	0.21151
O	-2.86627	-2.75392	0.68613
C	2.09560	1.83250	-2.31068
Cl	-0.27820	-3.19609	-0.89104
Cl	-2.58678	0.25693	1.23128
H	2.42471	1.72462	0.48976
C	-0.19871	-0.08475	-2.84722
H	2.86736	2.44030	-1.84519
H	2.55864	1.14476	-3.01674

H	1.40294	2.48086	-2.84533
H	-0.21458	0.33868	2.46142
H	0.97991	-0.96441	2.28159
H	1.53331	0.70456	2.56283
H	0.59010	-0.40120	-3.53337
H	-0.91609	-0.89790	-2.77429
H	-0.70063	0.78402	-3.28157

NHC_pdIV_oxo_s (*NImag* = 0)

C	3.06516	0.16762	0.47690
N	1.77771	0.48403	0.87000
C	0.90497	0.12275	-0.08249
N	1.62754	-0.41611	-1.07680
C	2.97158	-0.40339	-0.74689
C	1.05342	-1.00249	-2.28047
Pd	-1.12208	0.26231	-0.06707
Cl	-0.75952	2.47626	-0.48900
C	1.39560	1.09541	2.13592
O	-2.74219	-0.43046	-0.28142
Cl	-0.79981	-1.73534	1.14694
H	0.90656	2.04900	1.94696
H	0.71090	0.43131	2.66004
H	2.29131	1.24870	2.73182
H	0.30524	-0.32753	-2.68976
H	1.84541	-1.14902	-3.01044
H	0.58635	-1.95706	-2.04220
H	3.92023	0.38105	1.09133
H	3.72737	-0.79012	-1.40515

NHC_pdIV_oxo_t (*NImag* = 0)

N	-0.75586	0.94545	-0.11602
C	-1.16606	-0.29143	-0.44074
O	0.54469	-3.62797	0.50175
N	-2.29028	-0.13552	-1.15866
C	-1.62478	1.88882	-0.63200
C	-2.59320	1.20714	-1.28806
C	-3.05753	-1.23989	-1.71992
H	-2.40902	-1.83821	-2.35670
H	-3.45480	-1.85760	-0.91631
H	-3.87737	-0.83244	-2.30564
C	0.43350	1.22652	0.67757
H	0.35835	0.70637	1.63037
H	1.32012	0.88891	0.14364
H	0.49526	2.29858	0.84522
H	-3.45682	1.55129	-1.82643
H	-1.47826	2.94334	-0.48839
Pd	-0.26808	-2.04291	0.05387
Cl	-1.27001	-1.71743	2.13146
Cl	0.54116	-1.99256	-2.12981

NHCS_pdIV_oxo_s (*NImag* = 0)

N	1.03561	1.50333	0.13737
C	-0.03246	0.71949	0.04470
N	-1.13493	1.43565	-0.09941
C	-0.85192	2.87263	0.00160
C	0.67749	2.90619	-0.11233
Pd	0.11726	-1.30681	0.11211
Cl	0.06367	-0.94692	-2.20754
O	0.99850	-2.77465	-0.36327
Cl	-0.81172	-1.08914	2.19049
H	1.01241	3.19665	-1.11254
H	-1.35286	3.41992	-0.79610
H	-1.20543	3.25298	0.96427
H	1.14763	3.56221	0.61896
C	2.41462	1.06816	0.06861
H	2.47743	0.01625	0.33433
H	2.81010	1.19705	-0.94231
H	3.01542	1.64839	0.76954
C	-2.48955	0.93025	-0.11958
H	-2.95806	1.02944	0.86224
H	-3.06672	1.48821	-0.85746
H	-2.47651	-0.11758	-0.40274

NHCS_pdIV_oxo_t (*NImag* = 0)

N	0.29558	1.48658	1.03542
C	-0.06201	0.73435	0.00292
N	-0.43812	1.49365	-1.01781
C	-0.48035	2.91136	-0.63881
C	0.30163	2.91075	0.67917
Pd	-0.03751	-1.30041	-0.01213
Cl	-2.27820	-1.06654	0.57022
O	-0.01570	-3.13931	-0.02586
Cl	2.19705	-1.00555	-0.58920
H	1.33302	3.25237	0.54876
H	-0.02499	3.53331	-1.40843
H	-1.52008	3.22449	-0.50314
H	-0.16920	3.50855	1.45859
C	0.95237	1.01628	2.23516
H	0.72658	-0.03638	2.37897
H	2.03599	1.13696	2.15921
H	0.58266	1.57748	3.09397
C	-1.08500	1.02537	-2.22384
H	-2.17147	1.11152	-2.14232
H	-0.73551	1.61308	-3.07328
H	-0.82769	-0.01714	-2.38753

pyridine_pdIV_oxo_s (*NImag* = 0)

C	-5.37988	1.00609	-0.36822
C	-3.48603	2.10490	0.38066
C	-4.15352	3.31617	0.42042
C	-5.48630	3.35793	0.03577
C	-6.10581	2.18431	-0.36953
H	-5.81836	0.06092	-0.64905
H	-2.44222	2.02346	0.64911
H	-3.62921	4.20460	0.74169
H	-6.03302	4.29135	0.05063
H	-7.14087	2.17069	-0.67940
N	-4.09591	0.97881	-0.00047
Pd	-2.95056	-0.81542	-0.00187
Cl	-4.71223	-1.65787	1.28182
Cl	-2.32780	-0.08395	-2.06255
O	-1.98088	-2.15028	0.56969

pyridine_pdIV_oxo_t (*NImag* = 0)

C	-5.46503	0.98421	-0.21660
C	-3.48329	2.13014	0.21599
C	-4.14995	3.35132	0.23208
C	-5.52526	3.37614	-0.00344
C	-6.19014	2.17154	-0.23684
H	-5.94115	0.01258	-0.34718
H	-2.40391	2.05787	0.34839
H	-3.58628	4.26620	0.42007

H	-6.07182	4.32198	-0.00511
H	-7.26396	2.13954	-0.42641
N	-4.13598	0.97191	0.00078
Pd	-3.08514	-0.84584	0.00369
Cl	-4.85900	-1.77538	1.22070
Cl	-1.39267	0.22434	-1.21392
O	-2.18903	-2.39485	0.00651

ethylenediamine_oxo_s (*NImag* = 0)

C	1.41130	0.43178	0.45249
C	0.39875	1.23080	-0.34919
H	1.24243	0.57326	1.51869
H	0.59309	1.10953	-1.41412
N	1.29358	-1.01402	0.17067
N	-0.97819	0.76415	-0.09429
Pd	-0.76169	-1.48331	-0.04558
H	0.50404	2.29960	-0.11832
H	2.42681	0.78391	0.23305
O	-0.65084	-3.28335	-0.01192
C	1.84954	-1.82519	1.27621
H	1.64494	-2.87158	1.06510
H	2.92849	-1.64742	1.37299
H	1.34983	-1.55845	2.20393
C	1.97353	-1.38730	-1.08731
H	3.05424	-1.21375	-0.99797
H	1.77497	-2.43742	-1.28308
H	1.58132	-0.80236	-1.91492
C	-1.47937	1.25116	1.19832
H	-1.52591	2.34917	1.21914
H	-2.47502	0.84713	1.36635
H	-0.83915	0.90534	2.00590
C	-1.87413	1.20768	-1.16986
H	-2.87666	0.83237	-0.97480
H	-1.91016	2.30416	-1.23974
H	-1.53214	0.79644	-2.11721

ethylenediamine_oxo_t (*NImag* = 0)

C	1.44265	0.40835	0.52246
C	0.43701	1.21090	-0.28991
H	1.22233	0.50942	1.58479
H	0.67070	1.13186	-1.35118
N	1.40440	-1.03197	0.20005
N	-0.92704	0.72070	-0.09189
Pd	-0.75555	-1.66448	0.08880
H	0.52734	2.27440	-0.02380
H	2.45186	0.81216	0.36167
O	-2.44370	-2.43188	0.02885
C	2.10798	-1.79541	1.24175
H	2.06427	-2.85488	1.00078
H	3.16033	-1.48632	1.31944
H	1.61721	-1.64023	2.20031
C	2.02821	-1.30041	-1.10506
H	3.08017	-0.98096	-1.11114
H	1.97605	-2.36673	-1.31104
H	1.49469	-0.77952	-1.89610
C	-1.51526	1.18524	1.16454
H	-1.64862	2.27766	1.16359
H	-2.48142	0.70488	1.30265
H	-0.88262	0.90938	2.00576
C	-1.80590	1.03566	-1.21749
H	-2.76930	0.55565	-1.05783
H	-1.95341	2.12030	-1.32871
H	-1.37876	0.63856	-2.13675

propylenediamine_oxo_s (*NImag* = 0)

C	-4.76272	1.45687	1.02684
C	-6.26692	1.35837	0.79835
C	-6.85367	-0.02627	1.03795
H	-4.48254	2.50841	1.18455
H	-6.74436	2.03326	1.51336
H	-6.54209	1.73502	-0.18762
H	-6.43768	-0.42835	1.96257
H	-7.94039	0.05324	1.17903
N	-3.92936	0.89251	-0.05100
N	-6.60371	-1.02499	-0.02042
Pd	-4.60269	-1.13424	-0.75652
O	-4.85646	-2.75060	-1.54029
C	-2.54579	0.75593	0.43175
H	-1.92524	0.35071	-0.36515
H	-2.13512	1.72590	0.74631
H	-2.52283	0.06600	1.27259
C	-7.44561	-0.76204	-1.20827
H	-7.22757	-1.52315	-1.95229
H	-8.50761	-0.79265	-0.92891
H	-7.21446	0.21415	-1.62674
C	-6.94405	-2.37403	0.50127
H	-8.00567	-2.40544	0.78029
H	-6.70587	-3.10360	-0.26827
H	-6.33241	-2.58136	1.37572
C	-3.93954	1.77574	-1.22800
H	-4.94887	1.87368	-1.61827
H	-3.55428	2.77396	-0.97633
H	-3.31947	1.33902	-2.00779
H	-4.51264	0.91922	1.94316

propylenediamine_oxo_t (*NImag* = 0)

C	-4.74544	1.38533	1.03643
C	-6.25393	1.34746	0.80826
C	-6.92192	-0.00340	1.04563
H	-4.43909	2.42315	1.23551
H	-6.69323	2.04701	1.52428
H	-6.51184	1.74295	-0.17477
H	-6.55170	-0.41221	1.98790
H	-8.00644	0.14778	1.16894
N	-3.89857	0.85783	-0.05586
N	-6.68187	-1.00985	0.00973
Pd	-4.42155	-1.20455	-0.71504
O	-4.61809	-2.92174	-1.43621
C	-2.50192	0.82728	0.41428
H	-1.86098	0.45905	-0.38315
H	-2.16610	1.83011	0.71606
H	-2.41941	0.15255	1.26368
C	-7.42772	-0.72399	-1.21761
H	-7.19330	-1.48764	-1.95567
H	-8.51232	-0.71864	-1.02967
H	-7.14079	0.24349	-1.62496
C	-7.03316	-2.35013	0.49688
H	-8.10626	-2.41616	0.73030
H	-6.76311	-3.07912	-0.26364
H	-6.46003	-2.56917	1.39616
C	-3.97963	1.72574	-1.24245
H	-4.99003	1.73107	-1.64203

H	-3.68799	2.75617	-0.99356
H	-3.31446	1.34017	-2.01136
H	-4.50877	0.80473	1.92979

BiPy_oxo_t (*NImag* = 0)

C	-0.06346	1.20401	0.04200
C	0.53277	2.43878	-0.20729
C	-0.24853	3.58318	-0.16518
C	-1.60344	3.46848	0.11744
C	-2.12849	2.20304	0.33543
H	1.58362	2.50722	-0.44766
H	0.19410	4.55121	-0.35893
H	-2.24511	4.33735	0.16145
H	-3.18004	2.04549	0.53846
C	0.67526	-0.07704	0.03207
C	2.06553	-0.14929	0.07430
C	2.68393	-1.38893	0.07787
H	2.65740	0.75259	0.12302
C	0.52020	-2.38528	0.00100
C	1.89746	-2.53200	0.04324
H	3.76280	-1.46040	0.11407
H	-0.13815	-3.24196	-0.03655
H	2.33558	-3.51997	0.04536
N	-1.37580	1.10578	0.29891
N	-0.08046	-1.19227	-0.00566
Pd	-2.25144	-0.98261	0.06480
O	-4.10158	-1.13329	-0.00399

BiPy_oxo_s (*NImag* = 0)

C	-0.04661	1.17521	0.02570
C	0.52569	2.44583	-0.03131
C	-0.28862	3.56232	-0.02293
C	-1.67073	3.39622	0.04254
C	-2.18864	2.11781	0.09733
H	1.59880	2.55477	-0.08165
H	0.14504	4.55220	-0.06680
H	-2.34045	4.24413	0.05118
H	-3.24793	1.90969	0.14952
C	0.69751	-0.08081	0.02391
C	2.08759	-0.16821	-0.03567
C	2.69510	-1.40965	-0.03175
H	2.68631	0.72942	-0.08461
C	0.52239	-2.39562	0.08931
C	1.89288	-2.54804	0.03222
H	3.77223	-1.49310	-0.07753
H	-0.13958	-3.24929	0.13997
H	2.32317	-3.53948	0.03779
N	-1.39203	1.03720	0.08885
N	-0.07733	-1.19513	0.08606
Pd	-2.14363	-0.84036	0.16886
O	-3.91902	-0.67102	0.24319

bis_benzimi_oxo_t (*NImag* = 0)

N	-2.19434	-0.21395	-0.87040
C	-1.03733	-0.08482	-0.16726
C	-2.56778	1.43522	0.58914
C	-0.24907	1.39596	1.62856
H	-0.64014	2.22422	2.21166
H	0.03416	0.58559	2.29735
C	1.68423	0.90978	0.22205
N	0.92062	1.82138	0.90590
Pd	0.80732	-0.89731	-0.46736
C	-2.39229	-1.16988	-1.94090
H	-3.25798	-1.79911	-1.72853
H	-2.54582	-0.65421	-2.89005
H	-1.49840	-1.78582	-2.00568
C	3.64930	1.08012	-1.27940
H	3.59394	1.51364	-2.27929
H	4.62904	1.29611	-0.84988
H	3.48592	0.00137	-1.33020
N	2.60529	1.64892	-0.44229
C	2.41825	3.00938	-0.22342
C	1.31459	3.12775	0.63446
C	0.85089	4.36623	1.04872
C	3.09917	4.12826	-0.67940
C	1.53071	5.48970	0.58504
H	-0.00619	4.46685	1.70070
C	2.63620	5.37249	-0.26072
H	3.95390	4.03850	-1.33520
H	1.19474	6.47258	0.88675
H	3.14219	6.26690	-0.59835
O	2.40539	-1.83825	-1.00486
C	-3.15621	0.69493	-0.44587
C	-4.45675	0.93916	-0.86097
C	-5.15605	1.94827	-0.20602
H	-4.91051	0.37122	-1.66119
C	-3.26530	2.43920	1.24319
C	-4.57027	2.68501	0.82642
H	-6.17298	2.16656	-0.50250
H	-2.82388	3.01331	2.04626
H	-5.14223	3.46304	1.31360
N	-1.28439	0.92229	0.72659

bis_benzimi_oxo_s (*NImag* = 0)

N	-2.29120	-0.31388	-0.83726
C	-1.10604	0.05714	-0.27426
C	-2.77077	1.46206	0.43330
C	-0.42330	1.78700	1.32023
H	-0.83437	2.70807	1.72262
H	-0.14990	1.12388	2.14406
C	1.42054	1.17173	-0.19381
N	0.76201	2.10520	0.55565
Pd	0.77064	-0.62699	-0.59408
C	-2.43967	-1.41549	-1.76551
H	-3.14399	-2.15034	-1.37282
H	-2.79733	-1.05341	-2.73062
H	-1.46618	-1.88132	-1.89763
C	3.56124	1.13846	-1.47473
H	3.57803	1.59869	-2.46407
H	4.53093	1.27363	-0.99515
H	3.32134	0.06860	-1.53503
N	2.53855	1.77717	-0.65483
C	2.59548	3.09452	-0.21460
C	1.45555	3.31047	0.57142
C	1.21263	4.53453	1.17548
C	3.52565	4.10131	-0.42699
C	2.14559	5.54416	0.96097
H	0.33866	4.70884	1.78803
C	3.28079	5.33138	0.17379
H	4.40373	3.93610	-1.03494
H	1.98794	6.51344	1.41412
H	3.98426	6.14018	0.02990

O	2.29895	-1.52297	-1.01747
C	-3.33156	0.51280	-0.43356
C	-4.68404	0.52397	-0.73802
C	-5.46424	1.51316	-0.14464
H	-5.11794	-0.20480	-1.40836
C	-3.54718	2.44437	1.02681
C	-4.90648	2.45491	0.72167
H	-6.52360	1.55142	-0.35914
H	-3.12664	3.17707	1.70202
H	-5.54148	3.20861	1.16712
N	-1.41864	1.14691	0.49700

bisCAAC_diastereo_oxo_s (*NImag* = 0)

C	3.45982	-0.80049	-0.28293
N	2.52503	0.35005	-0.46345
C	1.38536	0.30184	0.20061
C	1.40805	-0.93573	1.07425
C	2.57072	-1.78049	0.50458
C	2.86688	1.43116	-1.37226
Pd	-0.12182	1.60582	0.09827
O	-1.11442	3.14129	0.05933
C	0.05085	-1.67633	1.07771
C	-0.92256	-1.37891	-0.07722
C	-1.34380	0.08190	-0.02437
N	-2.65150	0.16302	-0.05389
C	-3.37355	-1.14594	-0.15988
C	-2.24524	-2.14642	0.13631
C	1.70364	-0.47833	2.51633
C	-3.42371	1.40264	-0.03552
C	-3.95102	-1.30150	-1.56969
C	-4.49075	-1.23889	0.87703
C	-0.31225	-1.70168	-1.44935
C	4.70549	-0.36170	0.49276
C	3.87040	-1.37375	-1.63940
H	0.23563	-2.75442	1.10475
H	-0.47635	-1.43429	2.00220
H	0.93435	0.21702	2.84978
H	1.71606	-1.34341	3.18409
H	2.66589	0.02605	2.59421
H	3.88653	1.77198	-1.19069
H	2.78310	1.10733	-2.41104
H	2.17576	2.25408	-1.20737
H	-2.31799	-2.47431	1.17412
H	-2.31788	-3.03420	-0.49117
H	-4.70098	-0.53823	-1.77602
H	-3.17337	-1.22781	-2.32832
H	-4.43218	-2.27621	-1.66279
H	-5.31215	-0.55798	0.65575
H	-4.89012	-2.25434	0.88042
H	-4.11309	-1.01725	1.87600
H	-2.72979	2.25483	-0.00393
H	-4.04666	1.45270	-0.92954
H	-4.06756	1.41230	0.84405
H	3.13038	-2.29608	1.28517
H	2.18262	-2.54147	-0.17277
H	5.35836	-1.21969	0.65937
H	5.27617	0.38310	-0.06329
H	4.44472	0.06294	1.46040
H	4.43895	-2.29245	-1.48752
H	2.99388	-1.61188	-2.24314
H	4.50155	-0.68241	-2.19914
H	-0.00509	-2.74940	-1.48133
H	-1.03477	-1.53679	-2.24772
H	0.54858	-1.07344	-1.66176

bisCAAC_diastereo_oxo_t (*NImag* = 0)

N	2.55763	0.23359	-0.35508
C	1.47448	0.29848	0.38038
C	1.29864	-1.02842	1.09020
C	-0.14977	-1.56235	1.13248
H	-0.09581	-2.64715	1.27368
H	-0.63295	-1.16420	2.02730
C	1.74107	-0.79473	2.54941
H	1.12818	-0.01925	3.00765
H	1.63118	-1.71898	3.12224
H	2.78150	-0.47749	2.60845
C	-1.45988	0.20727	-0.05450
Pd	-0.09663	1.66173	0.08613
C	3.10455	1.37299	-1.08283
H	4.12390	1.57321	-0.75037
H	3.12024	1.16140	-2.15314
H	2.47490	2.24177	-0.88629
N	-2.77075	0.31016	-0.11756
C	-2.45688	-1.98757	0.19969
H	-2.54258	-2.27195	1.24942
H	-2.55595	-2.89672	-0.39282
C	-3.55240	-0.96311	-0.14063
C	-4.16066	-1.18267	-1.52937
H	-4.88215	-0.40285	-1.77618
H	-3.39267	-1.19287	-2.30120
H	-4.68631	-2.13833	-1.55509
C	-4.65615	-0.93958	0.91568
H	-5.43701	-0.21747	0.67396
H	-5.12294	-1.92401	0.97519
H	-4.24820	-0.69598	1.89755
C	-1.10639	-1.27081	-0.03919
C	-3.46623	1.58604	-0.17175
H	-2.72816	2.36597	-0.34500
H	-4.19581	1.58878	-0.98256
H	-3.98183	1.78605	0.76849
C	2.27827	-1.98648	0.36698
C	3.26772	-1.08819	-0.39655
H	2.79399	-2.64420	1.06707
H	1.74152	-2.62091	-0.33510
C	4.62639	-0.97870	0.30163
H	5.10475	-1.95891	0.32669
H	5.29321	-0.29885	-0.22900
H	4.52083	-0.62379	1.32573
C	3.46458	-1.55116	-1.83979
H	3.85921	-2.56853	-1.84336
H	2.51924	-1.55028	-2.38269
H	4.17321	-0.91878	-2.37500
O	0.98874	3.27946	0.11962
C	-0.51628	-1.63994	-1.40792
H	-0.24943	-2.69849	-1.43748
H	-1.23775	-1.45341	-2.20311
H	0.36390	-1.04177	-1.62923

bisCAAC_oxo_t (*NImag* = 0)

N	2.08606	-0.42088	-0.54519
C	1.44491	0.35789	0.31825
C	1.12397	-0.48379	1.54261

C	-0.19858	-0.09084	2.22994
H	-0.39906	-0.79816	3.04079
H	-0.01097	0.87287	2.70520
C	2.24345	-0.22448	2.57178
H	2.25796	0.82783	2.85429
H	2.07692	-0.82620	3.46891
H	3.22496	-0.47132	2.17190
C	-1.14884	0.52991	-0.01345
Pd	0.26494	1.89793	-0.32408
C	2.63657	0.08305	-1.79072
H	3.63039	-0.33262	-1.96431
H	1.99752	-0.17510	-2.63902
H	2.70149	1.16552	-1.72147
N	-1.80644	-0.21897	-0.87247
C	-2.29456	-1.23906	1.18149
H	-3.15043	-1.29893	1.85351
H	-1.67543	-2.11208	1.37844
C	-2.73903	-1.23597	-0.29277
C	-2.55099	-2.59848	-0.95558
H	-2.84317	-2.58302	-2.00639
H	-1.51566	-2.93112	-0.88821
H	-3.17639	-3.33553	-0.44981
C	-4.18682	-0.76725	-0.47236
H	-4.45931	-0.71137	-1.52700
H	-4.86596	-1.47369	0.00673
H	-4.34230	0.21481	-0.02886
C	-1.48805	0.06732	1.38655
C	-2.36063	1.14218	2.06238
H	-2.58192	0.84475	3.09029
H	-1.84051	2.09881	2.07524
H	-3.30610	1.28086	1.53807
C	-1.71578	-0.03820	-2.31143
H	-1.19050	0.89505	-2.49935
H	-1.16310	-0.85889	-2.77202
H	-2.71063	0.00473	-2.75627
C	1.19572	-1.93405	1.00608
C	2.09776	-1.88142	-0.24443
H	1.57081	-2.63721	1.75048
H	0.20637	-2.27298	0.70869
C	3.52241	-2.38003	0.01399
H	3.50520	-3.44431	0.25424
H	4.14916	-2.25167	-0.86939
H	3.98954	-1.85040	0.84165
C	1.48785	-2.66323	-1.40954
H	1.34942	-3.70751	-1.12395
H	0.51654	-2.24949	-1.68050
H	2.12958	-2.64173	-2.29109
O	1.17063	3.50889	-0.82829

bisCAAC_oxo_s (*NImag* = 0)

N	1.87910	-0.38363	-0.77071
C	1.16614	0.37083	0.04990
C	1.32433	-0.15854	1.46044
C	0.00002	-0.07240	2.25319
H	0.00002	-0.82982	3.04212
H	0.00001	0.89212	2.75916
C	2.35662	0.71935	2.19663
H	2.01657	1.75364	2.21834
H	2.47852	0.36312	3.22234
H	3.33076	0.69878	1.71181
C	-1.16606	0.37082	0.04988
Pd	0.00005	1.87415	-0.40130
C	2.00656	-0.11538	-2.19176
H	3.04086	-0.24890	-2.50992
H	1.37197	-0.78426	-2.77701
H	1.69661	0.91128	-2.37207
N	-1.87911	-0.38356	-0.77072
C	-1.87111	-1.58753	1.23890
H	-2.57549	-1.88643	2.01498
H	-1.05468	-2.30925	1.24516
C	-2.52642	-1.57977	-0.15800
C	-2.17514	-2.83908	-0.95025
H	-2.62924	-2.83581	-1.94176
H	-1.09553	-2.93848	-1.06418
H	-2.54309	-3.71809	-0.41902
C	-4.04824	-1.41176	-0.11603
H	-4.46422	-1.34479	-1.12213
H	-4.50181	-2.27605	0.37125
H	-4.34040	-0.51785	0.43053
C	-1.32427	-0.15857	1.46040
C	-2.35663	0.71926	2.19656
H	-2.47851	0.36306	3.22228
H	-2.01668	1.75358	2.21825
H	-3.33077	0.69859	1.71172
C	-2.00663	-0.11526	-2.19175
H	-1.69666	0.91139	-2.37205
H	-1.37207	-0.78414	-2.77705
H	-3.04094	-0.24876	-2.50987
C	1.87122	-1.58751	1.23898
C	2.52633	-1.57989	-0.15801
H	2.57569	-1.88633	2.01501
H	1.05480	-2.30925	1.24539
C	4.04819	-1.41210	-0.11629
H	4.50170	-2.27643	0.37098
H	4.46402	-1.34528	-1.12247
H	4.34059	-0.51821	0.43014
C	2.17477	-2.83919	-0.95014
H	2.54267	-3.71822	-0.41891
H	1.09514	-2.93844	-1.06393
H	2.62874	-2.83603	-1.94172
O	0.00012	3.62811	-0.80541

bisDAC_oxo_t (*NImag* = 0)

C	-2.22472	2.86874	-0.19763
N	-1.24953	1.93226	-0.55105
C	-1.46026	0.69573	0.00409
N	-2.59308	0.79267	0.75051
C	-3.17636	2.06519	0.68834
C	-0.05065	2.23397	-1.28778
N	1.11786	1.91284	-0.48298
C	2.26554	2.71397	-0.42134
C	3.19727	1.92491	0.49596
N	2.46752	0.76632	0.81955
C	1.23144	0.78101	0.27309
C	2.98516	-0.26027	1.71806
C	-3.16409	-0.30017	1.52074
H	-3.22002	-1.19515	0.90344
H	-2.55667	-0.50289	2.40237
H	-4.16235	-0.00179	1.83108
H	2.63518	-1.22903	1.36880
H	4.07016	-0.20657	1.68709
H	2.64721	-0.07210	2.73710

H	-0.02721	1.65543	-2.21179
H	-0.03047	3.29764	-1.51380
Pd	-0.02731	-0.72539	0.05173
O	2.43292	3.77376	-0.95787
O	4.28906	2.22005	0.89470
O	-2.28109	4.02097	-0.52971
O	-4.19671	2.41917	1.21375
O	1.14602	-2.22202	0.00478

bisDAC_oxo_s (*NImag* = 0)

C	-2.32929	2.83173	-0.33775
N	-1.22079	1.99475	-0.42243
C	-1.39656	0.78781	0.21267
N	-2.65488	0.80851	0.74056
C	-3.33105	2.00280	0.48617
C	-0.02665	2.29211	-1.17583
N	1.15126	1.95835	-0.40231
C	2.26818	2.79000	-0.28168
C	3.24921	1.94257	0.54814
N	2.56299	0.74328	0.78137
C	1.32495	0.75825	0.23330
C	3.16282	-0.36961	1.51774
C	-3.22555	-0.27755	1.52412
H	-3.24048	-1.19321	0.93536
H	-2.63731	-0.43770	2.42624
H	-4.24036	0.00623	1.79061
H	2.58940	-1.26985	1.29306
H	4.19450	-0.47248	1.18953
H	3.14752	-0.15337	2.58546
H	-0.02262	1.71848	-2.10392
H	-0.00333	3.35651	-1.39829
Pd	-0.01246	-0.63049	0.36581
O	2.40484	3.88980	-0.72755
O	4.34819	2.23026	0.91769
O	-2.45798	3.92579	-0.80721
O	-4.43383	2.30916	0.83872
O	0.89406	-2.18289	0.48163

bisimine_oxo_s (*NImag* = 0)

C	0.23308	-0.87470	-2.53256
N	0.83396	-0.65993	-1.23259
Pd	-0.05536	0.31778	0.33983
O	-1.02596	1.19367	1.53981
N	1.72743	-0.18960	1.10734
C	2.10777	0.14502	2.46692
C	2.52778	-0.83849	0.31521
C	2.03548	-1.10492	-0.99261
H	3.51246	-1.15295	0.64679
H	2.61947	-1.63948	-1.73513
H	3.11355	-0.21788	2.69317
H	2.06421	1.22589	2.59436
H	1.38903	-0.29422	3.15750
H	-0.01975	0.09069	-2.97426
H	0.89661	-1.42014	-3.20871
H	-0.69666	-1.43263	-2.40939

bisimine_oxo_t (*NImag* = 0)

Pd	-0.13416	-0.84770	-0.00302
O	-1.61802	-1.94985	0.01887
N	1.54372	0.37411	-0.00011
N	-0.96894	1.31328	-0.00916
C	-2.34308	1.74742	-0.00824
C	2.90547	-0.13040	-0.00499
C	-0.00522	2.15349	0.01099
C	1.35226	1.65209	0.01058
H	3.64541	0.67492	-0.00481
H	3.05134	-0.75494	-0.88739
H	3.05635	-0.76028	0.87271
H	-2.84907	1.31482	0.85646
H	-2.83924	1.35097	-0.89574
H	-2.44299	2.83826	0.01357
H	2.186685	2.34986	0.01976
H	-0.17305	3.23221	0.02855

bisMIC_diazo_oxo_s (*NImag* = 0)

N	-3.18257	-1.88057	0.16749
C	-2.71679	-0.60695	0.47891
C	-1.44903	-0.43950	-0.05933
N	-1.22544	-1.67577	-0.69201
C	-2.25685	-2.52508	-0.54552
Pd	0.03117	0.96143	0.17849
O	1.15042	2.36399	0.56392
C	-3.51073	0.34837	1.29221
C	-0.02725	-1.92836	-1.45415
N	1.15061	-1.68012	-0.65094
C	1.36243	-0.46289	-0.00423
C	2.63688	-0.56996	0.51814
N	3.12756	-1.82803	0.18217
C	2.21440	-2.49240	-0.53141
C	3.40500	0.44273	1.27999
C	4.43410	-2.34289	0.55040
C	-4.46599	-2.43477	0.55846
H	-0.01940	-2.95852	-1.80683
H	-0.00843	-1.23963	-2.30077
H	-4.45830	0.62046	0.81722
H	-3.73643	-0.03970	2.29007
H	-2.92018	1.25483	1.41355
H	4.36613	0.66164	0.80556
H	2.79090	1.35766	1.27663
H	3.59912	0.12426	2.30947
H	4.55237	-3.34795	0.15206
H	5.21284	-1.69873	0.14497
H	4.53016	-2.36987	1.63487
H	-5.27452	-1.83327	0.14472
H	-4.54968	-3.45244	0.18442
H	-4.55343	-2.44371	1.64423
H	2.32931	-3.47728	-0.94605
H	-2.34389	-3.52391	-0.93335

bisMIC_diazo_oxo_t (*NImag* = 0)

N	-3.12634	-1.89805	0.15724
C	-2.69804	-0.61916	0.51252
C	-1.46910	-0.37034	-0.07240
N	-1.21860	-1.56269	-0.77358
C	-2.20485	-2.45620	-0.62918
Pd	-0.03318	1.09690	0.18464
O	1.08778	2.57149	0.74194
C	-3.50474	0.26442	1.39164
C	-0.02938	-1.76959	-1.58822
N	1.16981	-1.55870	-0.82115
C	1.49274	-0.29262	-0.32554
C	2.68019	-0.51111	0.34901

N	3.00807	-1.86209	0.24848
C	2.06874	-2.49495	-0.46183
C	3.49965	0.46931	1.10424
C	4.19449	-2.48854	0.80425
C	-4.36746	-2.53008	0.56739
H	-0.04484	-2.78062	-1.99279
H	-0.04223	-1.04161	-2.39706
H	-4.50155	0.46158	0.98508
H	-3.62896	-0.14777	2.39755
H	-2.97458	1.21137	1.47980
H	4.48083	0.63091	0.64628
H	2.94329	1.41312	1.10369
H	3.66212	0.15675	2.14061
H	4.18456	-3.55218	0.57780
H	5.08897	-2.03762	0.37603
H	4.21530	-2.35036	1.88438
H	-5.21789	-1.94282	0.22315
H	-4.42533	-3.52759	0.13773
H	-4.40719	-2.60495	1.65342
H	2.05514	-3.54534	-0.69216
H	-2.26689	-3.43536	-1.07012

bisNHC_oxo_s (*NImag* = 0)

C	-2.38518	2.77772	-0.34460
N	-1.23786	2.04324	-0.41076
C	-1.35445	0.84732	0.28103
C	-2.60326	0.83394	0.79645
N	-3.21841	2.01390	0.40922
C	-0.10641	2.49737	-1.18518
N	0.18293	3.87694	-0.87015
C	1.42634	4.43624	-0.61816
C	1.21189	5.75784	-0.43715
N	-0.14901	5.97530	-0.58314
C	-0.80504	4.81709	-0.85545
Pd	-2.74304	4.54892	-1.15002
C	-0.80407	7.26721	-0.45667
C	-4.57486	2.40058	0.76226
O	-4.14703	5.45462	-1.86926
H	1.90196	6.55294	-0.22008
H	2.33385	3.86030	-0.59919
H	-0.56102	0.12415	0.33697
H	-3.10157	0.09529	1.39778
H	-1.83341	7.15416	-0.79263
H	-0.79650	7.59748	0.58257
H	-0.29182	8.00169	-1.07775
H	-4.83418	3.28129	0.17719
H	-5.26167	1.58729	0.52894
H	-4.63803	2.63983	1.82431
H	-0.34158	2.41299	-2.24931
H	0.76062	1.88429	-0.95081

bisNHC_oxo_t (*NImag* = 0)

C	-2.36723	2.81034	-0.42521
N	-1.26113	2.01386	-0.48924
C	-1.38332	0.89448	0.32176
C	-2.60847	0.98003	0.88674
N	-3.19014	2.14917	0.41950
C	-0.10990	2.40825	-1.26278
N	0.50914	3.61114	-0.73206
C	1.83221	3.74408	-0.34676
C	1.97247	5.01794	0.08108
N	0.73334	5.62306	-0.05601
C	-0.19470	4.76879	-0.55921
Pd	-2.23571	4.91986	-0.72911
C	0.44683	6.99921	0.30675
C	-4.52332	2.62101	0.77303
O	-4.11695	5.37959	-0.73128
H	2.83241	5.53582	0.46599
H	2.54581	2.94269	-0.41627
H	-0.61632	0.14599	0.41200
H	-3.11145	0.31500	1.56590
H	-0.56301	7.22365	-0.03004
H	0.50220	7.12966	1.38795
H	1.15744	7.66949	-0.17771
H	-4.65506	3.60976	0.32407
H	-5.27480	1.92999	0.38866
H	-4.61362	2.68494	1.85799
H	-0.42901	2.60075	-2.28558
H	0.62359	1.60563	-1.25456

bisNHCS_oxo_t (*NImag* = 0)

C	-2.11166	2.88280	0.02437
N	-1.34939	1.82520	-0.65322
C	-1.56004	0.60396	-0.06823
N	-2.63150	0.73820	0.72517
C	-3.23301	2.07181	0.66939
C	-0.06913	2.11203	-1.23601
N	1.02622	1.99608	-0.27424
C	2.27347	2.75199	-0.46563
C	3.18222	2.09207	0.57388
N	2.50048	0.80849	0.79186
C	1.25859	0.80335	0.32945
C	3.09114	-0.24063	1.59846
C	-3.29602	-0.34644	1.40456
H	-2.72400	-1.25662	1.23938
H	-3.35702	-0.15228	2.47827
H	-4.31144	-0.48219	1.01696
H	2.50608	-1.15016	1.44870
H	4.11704	-0.41507	1.26800
H	3.10841	0.04420	2.65543
H	0.09372	1.42441	-2.07050
H	-0.07610	3.13271	-1.61762
Pd	-0.08983	-0.80451	0.06893
H	-4.13912	2.05611	0.05246
H	-1.49075	3.37788	0.78024
H	2.12716	3.81826	-0.29527
H	4.20231	1.93916	0.22167
H	-2.47348	3.63026	-0.68106
H	-3.50162	2.42742	1.66488
H	2.65793	2.60962	-1.48255
H	3.22125	2.66169	1.50877
O	1.10894	-2.31949	0.25632

bisNHCS_oxo_s (*NImag* = 0)

C	-2.47629	2.80794	-0.38007
N	-1.22439	2.10698	-0.09033
C	-1.43279	0.84556	0.37504
N	-2.74435	0.74970	0.65476
C	-3.45969	2.01395	0.47962
C	-0.02340	2.43176	-0.81447
N	1.15515	2.06807	-0.06645
C	2.42067	2.75929	-0.32399

C	3.38841	1.93180	0.52085
N	2.65432	0.67523	0.69003
C	1.36522	0.80328	0.38753
C	3.28792	-0.46815	1.32026
C	-3.34620	-0.33740	1.38841
H	-2.65626	-1.17878	1.39809
H	-3.55887	-0.04554	2.42234
H	-4.27980	-0.64423	0.91205
H	2.58181	-1.31020	1.29756
H	4.19267	-0.72337	0.76440
H	3.56540	-0.21923	2.34959
H	-0.01940	1.94383	-1.80267
H	0.00059	3.51155	-0.96798
Pd	-0.01492	-0.57402	0.58497
H	-4.42528	1.86121	-0.00320
H	-2.41972	3.86175	-0.10708
H	2.37049	3.80507	-0.02148
H	4.34659	1.75859	0.03194
H	-2.72596	2.73495	-1.44598
H	-3.63127	2.49196	1.45149
H	2.67424	2.71395	-1.39006
H	3.57682	2.38833	1.49847
O	0.91913	-2.12352	0.81919

CAAC_benzimi_oxo_t (*NImag* = 0)

N	-1.85936	-0.45491	-0.53174
C	-0.93994	-0.51941	0.41490
C	-2.17432	1.53749	0.67237
C	-2.76333	0.73524	-0.50668
H	-2.95474	1.96813	1.30031
C	-1.27047	0.55212	1.44164
C	-0.01724	1.21874	2.01893
H	-0.30018	2.04876	2.66532
H	0.53117	0.49280	2.61935
C	-2.00360	-0.12857	2.60931
H	-1.36619	-0.88262	3.07121
H	-2.28307	0.60952	3.36584
H	-2.90953	-0.62612	2.26879
C	1.44870	0.88007	0.08426
N	0.87450	1.72198	0.98981
Pd	0.99236	-1.08133	-0.06044
C	-1.94375	-1.43356	-1.60548
H	-2.94718	-1.86056	-1.65009
H	-1.71721	-0.97117	-2.56819
H	-1.21753	-2.21946	-1.40328
C	2.95137	1.20964	-1.86778
H	2.54411	1.58587	-2.80779
H	3.98777	1.53646	-1.76971
H	2.90868	0.12302	-1.86538
N	2.16806	1.68597	-0.74681
C	2.04890	3.02026	-0.38194
C	-2.64872	1.51362	-1.81816
H	-3.06930	0.95994	-2.65810
H	-3.19370	2.45540	-1.73458
H	-1.60434	1.74027	-2.03811
C	-4.21433	0.30932	-0.27238
H	-4.85628	1.18999	-0.22023
H	-4.57800	-0.31754	-1.08716
H	-4.31987	-0.24689	0.65805
H	-1.57248	2.35925	0.28192
C	1.21079	3.04519	0.74324
C	0.87658	4.23966	1.36429
C	2.57666	4.18570	-0.91781
C	1.40523	5.40914	0.82614
H	0.23042	4.26851	2.23055
C	2.24082	5.38345	-0.29372
H	3.22132	4.16754	-1.78566
H	1.16399	6.35814	1.28573
H	2.63412	6.31252	-0.68360
O	0.83457	-2.94712	-0.50673

CAAC_MIC_diazo_oxo_s (*NImag* = 0)

N	-3.08551	-0.93541	0.80562
C	-1.81010	-1.49105	0.90360
C	-0.95336	-0.75197	0.11429
N	-1.77514	0.23985	-0.43211
C	-3.03626	0.11949	-0.01305
Pd	1.06584	-0.83473	-0.27337
O	2.59128	-1.67257	-0.75487
C	-1.52112	-2.67991	1.74431
C	-1.25257	1.22021	-1.36685
C	-0.01341	1.95501	-0.81329
C	0.83566	1.01736	0.04030
N	1.14804	1.70907	1.15274
C	0.45180	2.93303	1.21852
C	-0.28964	3.11014	0.12428
C	1.93420	1.18133	2.25225
C	0.81809	2.44607	-2.01908
H	-3.87116	0.73872	-0.28732
H	-0.86867	3.98657	-0.12101
H	0.58715	3.57530	2.07519
H	-2.03836	1.93159	-1.61932
H	-0.96132	0.68036	-2.26741
H	1.14093	1.59148	-2.61353
H	0.22494	3.12186	-2.64011
H	1.70176	2.98124	-1.67559
H	2.56941	1.96765	2.66186
H	1.28693	0.79242	3.04194
H	2.54515	0.36767	1.86759
H	-2.09772	-3.55737	1.43758
H	-0.46412	-2.91846	1.63914
H	-1.72464	-2.50131	2.80432
C	-4.27800	-1.41849	1.47749
H	-4.50629	-2.43373	1.15474
H	-4.12675	-1.41671	2.55629
H	-5.11599	-0.76971	1.23395

CAAC_MIC_diazo_oxo_t (*NImag* = 0)

N	1.14337	1.81005	1.12721
C	0.98405	1.12267	-0.07651
C	-0.08096	1.67640	-0.75306
N	-0.52329	2.69338	0.09279
C	0.21001	2.76406	1.20165
Pd	-0.94208	1.32996	-2.56297
O	-1.62953	0.46671	-4.12452
C	1.86976	-0.00323	-0.46743
C	-1.63100	3.55926	-0.27670
C	-1.35061	4.28733	-1.59816
C	-0.86828	3.32579	-2.69872
N	0.25487	3.91212	-3.20938
C	0.64336	5.04108	-2.48293
C	-0.22828	5.30089	-1.50272

C	1.06463	3.32331	-4.25515
C	-2.66461	4.95502	-2.05329
H	0.08340	3.45303	2.01709
H	-0.20268	6.14352	-0.82931
H	1.52598	5.58936	-2.77821
H	-1.80189	4.27647	0.52698
H	-2.51631	2.93268	-0.38598
H	-3.43221	4.19873	-2.22091
H	-3.01885	5.66772	-1.30329
H	-2.50477	5.49177	-2.98685
H	1.49698	4.10787	-4.87798
H	1.87029	2.71493	-3.83275
H	0.43086	2.68155	-4.86198
H	1.83510	-0.82821	0.25007
H	1.53268	-0.37991	-1.43128
H	2.91432	0.30504	-0.56971
C	2.15273	1.54365	2.13532
H	3.14877	1.66201	1.70986
H	2.03173	2.24363	2.95852
H	2.04848	0.52711	2.51322

CAAC_MIC_para_oxo_t (*NImag* = 0)

N	-3.04588	-1.01407	0.76677
C	-1.82393	-1.52511	0.91952
C	-0.89226	-0.82220	0.11572
N	-1.64844	0.09789	-0.49833
N	-2.93733	-0.01514	-0.08331
Pd	1.06978	-0.98570	-0.24281
O	1.52609	-2.84808	-0.43597
C	-1.53277	-2.67698	1.81258
C	-1.15277	1.07247	-1.46131
C	0.01349	1.89976	-0.88072
C	0.98151	1.01193	-0.07856
N	1.11394	1.65091	1.12165
C	0.26567	2.75569	1.24440
C	-0.43502	2.94164	0.12208
C	1.85595	1.10560	2.23800
C	0.74717	2.55407	-2.06786
C	-4.06304	0.71989	-0.61050
H	-1.10535	3.76092	-0.08687
H	0.27506	3.34016	2.15241
H	-1.96784	1.72497	-1.76667
H	-0.80951	0.51190	-2.33048
H	1.15864	1.78649	-2.72440
H	0.07073	3.19349	-2.64210
H	1.56852	3.16792	-1.70218
H	-3.93126	1.79019	-0.45375
H	-4.20399	0.51404	-1.67304
H	-4.93162	0.37726	-0.05698
H	2.20814	1.91282	2.88112
H	1.23907	0.42149	2.82741
H	2.70868	0.54986	1.85152
H	-2.43805	-3.24780	2.01378
H	-0.78526	-3.31635	1.34210
H	-1.12081	-2.33683	2.76560

CAAC_MIC_oxo_s (*NImag* = 0)

N	1.10079	1.64922	1.18876
C	0.81507	1.00551	0.04956
C	-0.31622	3.08694	0.18362
C	0.40038	2.87376	1.28644
H	-0.89650	3.96736	-0.04274
H	0.51643	3.48156	2.17013
C	-0.02358	1.96382	-0.78488
C	-1.25763	1.24257	-1.39043
H	-2.06229	1.95275	-1.56121
H	-0.96781	0.80665	-2.34609
C	0.82686	2.49572	-1.96050
H	1.16467	1.66171	-2.57537
H	0.23932	3.18610	-2.57003
H	1.70111	3.02576	-1.58633
C	-0.92650	-0.77254	-0.02314
C	-1.82196	-1.49861	0.83403
N	-1.73198	0.15586	-0.55810
N	-2.99418	0.09475	0.01048
N	-3.03396	-0.98346	0.80119
C	-4.17897	0.43630	-0.75789
H	-4.13921	1.47963	-1.06183
H	-4.28099	-0.20532	-1.63743
H	-5.02934	0.28907	-0.09885
Pd	1.07124	-0.84042	-0.34715
C	1.86390	1.08319	2.28730
H	2.49165	1.85438	2.73412
H	1.19546	0.67532	3.04871
H	2.48096	0.28019	1.89126
C	-1.46818	-2.66043	1.68922
H	-2.35856	-3.07846	2.15534
H	-0.97180	-3.42845	1.09427
H	-0.76431	-2.36171	2.46872
O	2.63351	-1.58956	-0.83124

CAAC_NHC_oxo_s (*NImag* = 0)

C	0.70242	3.16658	-0.19981
N	0.83070	1.80491	-0.41262
C	-0.20360	1.13112	0.15121
N	-0.97896	2.07922	0.72064
C	-0.43142	3.33552	0.51955
Pd	-0.42259	-0.81724	0.18219
O	-2.24610	-0.97875	0.22125
C	1.78468	1.13897	-1.28570
C	2.50366	-0.04763	-0.62391
C	1.57961	-0.82440	0.30304
N	2.30486	-1.19632	1.34271
C	3.68189	-0.62896	1.44773
C	3.66818	0.38351	0.28682
C	1.76808	-2.00607	2.42333
C	3.86239	0.07182	2.79496
C	4.73937	-1.72425	1.28616
C	2.95800	-0.98434	-1.75637
C	-2.23371	1.81042	1.41796
H	4.62395	0.42131	-0.23633
H	2.51347	1.87810	-1.61860
H	1.23788	0.78320	-2.15902
H	2.09194	-1.36923	-2.29417
H	3.59940	-0.44644	-2.45965
H	3.51771	-1.83472	-1.37285
H	2.50879	-2.73582	2.75178
H	1.48117	-1.38546	3.27379
H	0.88366	-2.52228	2.06010
H	-2.10275	1.96543	2.49003
H	-2.99825	2.48931	1.04202
H	-2.51559	0.77247	1.19995

H	-0.89626	4.22866	0.89545
H	1.40856	3.88088	-0.58257
H	3.46747	1.37964	0.68695
H	3.87541	-0.63625	3.62421
H	4.81277	0.60781	2.80143
H	3.06139	0.79250	2.96508
H	5.73768	-1.28680	1.33526
H	4.66463	-2.46352	2.08460
H	4.63994	-2.24298	0.33460

CAAC_NHC_oxo_t (*NImag* = 0)

N	2.26121	-1.35332	1.18705
C	1.57937	-1.04272	0.09394
C	3.45825	0.45905	0.29324
C	3.48678	-0.54615	1.46307
H	4.45101	0.61896	-0.12887
C	2.46224	-0.11944	-0.73331
C	1.67325	0.96308	-1.47543
H	2.35926	1.66004	-1.95785
H	1.06315	0.49408	-2.24743
C	3.16092	-0.97660	-1.80276
H	2.42481	-1.43758	-2.46147
H	3.83710	-0.36261	-2.40393
H	3.74214	-1.77653	-1.34839
C	-0.18359	1.11939	0.11725
N	0.79549	1.72471	-0.60300
Pd	-0.47039	-0.88973	0.20703
C	1.79752	-2.35109	2.13911
H	2.56862	-3.10671	2.30084
H	1.55386	-1.88969	3.09842
H	0.90246	-2.81870	1.73116
C	-1.86861	1.97193	1.72600
H	-1.55755	2.20657	2.74466
H	-2.69747	2.62073	1.44154
H	-2.18427	0.93218	1.67745
N	-0.76109	2.14016	0.80330
C	-0.15522	3.35149	0.51248
C	0.82819	3.08822	-0.37861
H	-0.47379	4.27927	0.95217
H	1.52737	3.74167	-0.86833
H	3.09327	1.42161	0.65564
C	3.35836	0.16764	2.81010
H	3.38657	-0.53334	3.64475
H	4.18658	0.86736	2.93531
H	2.42352	0.72792	2.85767
C	4.73584	-1.43117	1.45880
H	5.62421	-0.82102	1.62953
H	4.69069	-2.17839	2.25173
H	4.85400	-1.94986	0.50850
O	-1.09281	-2.67808	0.54462

.

CAAC_NHC_para_oxo_t (*NImag* = 0)

C	0.77516	3.13433	-0.27687
N	0.84100	1.76454	-0.46480
C	-0.15674	1.13132	0.19838
N	-0.84608	2.11785	0.80775
C	-0.28717	3.35625	0.53338
Pd	-0.43654	-0.90763	0.13566
O	-2.37033	-1.07765	0.27020
C	1.72633	1.04211	-1.36832
C	2.48295	-0.11195	-0.69306
C	1.57385	-0.97040	0.18077
N	2.27231	-1.27214	1.27166
C	3.58323	-0.58789	1.45459
C	3.56888	0.40029	0.27454
C	1.74682	-2.12541	2.32066
C	3.62033	0.14551	2.79643
C	4.73553	-1.59390	1.37571
C	3.06280	-0.98691	-1.81587
C	-2.03544	1.88313	1.61745
H	4.54772	0.48622	-0.19819
H	2.43790	1.75486	-1.78662
H	1.12116	0.64709	-2.18465
H	2.25955	-1.41187	-2.41723
H	3.71622	-0.39668	-2.46449
H	3.64533	-1.81258	-1.41154
H	2.54108	-2.74287	2.74302
H	1.29479	-1.53897	3.12322
H	0.97884	-2.76307	1.88778
H	-1.79018	1.94296	2.67886
H	-2.78647	2.63502	1.37782
H	-2.41369	0.88989	1.36763
H	-0.69163	4.27273	0.92381
H	1.46576	3.81951	-0.73458
H	3.29029	1.38979	0.64174
H	3.62800	-0.54810	3.63826
H	4.52498	0.75284	2.85875
H	2.75700	0.80469	2.89763
H	5.69041	-1.07712	1.48388
H	4.66954	-2.33387	2.17430
H	4.73693	-2.12202	0.42351

CAAC_NHC_oxo_s (*NImag* = 0)

N	2.26821	-1.17983	1.33953
C	1.57406	-0.77499	0.29964
C	3.63919	0.41432	0.29785
C	3.64091	-0.60209	1.46037
H	4.60344	0.45835	-0.20839
C	2.49630	-0.02320	-0.63813
C	1.78900	1.14855	-1.34065
H	2.52513	1.88060	-1.67331
H	1.26533	0.76787	-2.21816
C	2.96788	-0.99181	-1.73653
H	2.10944	-1.39035	-2.27577
H	3.62224	-0.47021	-2.43981
H	3.51741	-1.83324	-1.32170
C	-0.19842	1.11845	0.08284
N	0.81688	1.81879	-0.49813
Pd	-0.32508	-0.92782	0.10816
C	1.72926	-2.06468	2.36547
H	2.46245	-2.83514	2.60394
H	1.49482	-1.50554	3.27295
H	0.82305	-2.52295	1.97033
C	-2.13539	1.80317	1.47202
H	-2.01589	2.15488	2.49739
H	-3.00059	2.29029	1.02078
H	-2.28855	0.72653	1.47778
N	-0.93840	2.07823	0.70025
C	-0.40157	3.34094	0.49877
C	0.70577	3.17685	-0.25988

H	-0.84726	4.23089	0.90500
H	1.40414	3.89575	-0.64888
H	3.42160	1.40844	0.69368
C	3.79078	0.09728	2.81127
H	3.79093	-0.61470	3.63686
H	4.73810	0.63826	2.83715
H	2.98216	0.81229	2.96803
C	4.70879	-1.68885	1.31656
H	5.70122	-1.23844	1.36491
H	4.63718	-2.41764	2.12420
H	4.62007	-2.22111	0.37182
O	-0.85340	-2.66480	0.13777

CAAC_PPh2_oxo_t (*NImag* = 0)

C	-3.18231	0.58997	0.94850
N	-2.70497	-0.49127	0.02548
C	-1.57802	-1.08230	0.34759
C	-1.14735	-0.57586	1.72406
C	-1.99209	0.70384	1.92043
C	-3.45255	-0.81769	-1.18253
Pd	-0.20121	-1.65989	-1.08228
O	-1.10717	-2.83018	-2.35077
C	0.37253	-0.31192	1.77143
P	1.03876	-0.11497	0.05501
C	0.63908	1.61164	-0.39009
C	0.82461	2.68278	0.48740
C	0.45578	3.96889	0.11857
C	-0.09905	4.20154	-1.13642
C	-0.28237	3.14427	-2.01860
C	0.08191	1.85543	-1.64561
C	-1.50490	-1.66112	2.75377
C	-3.39688	1.89150	0.17672
C	-4.48149	0.16413	1.63591
C	2.84364	-0.14467	0.30739
C	3.41386	-1.36064	0.70092
C	4.78334	-1.47194	0.88368
C	5.60948	-0.37514	0.65531
C	5.05476	0.82908	0.24285
C	3.67978	0.94671	0.07108
H	-2.32471	0.82408	2.95166
H	0.61721	0.52722	2.42379
H	0.89012	-1.18549	2.16945
H	-0.98172	-2.58857	2.52042
H	-1.21537	-1.33718	3.75611
H	-2.57361	-1.87410	2.75894
H	-2.95257	-1.64670	-1.68214
H	-4.47481	-1.09835	-0.92517
H	-3.48377	0.04301	-1.85201
H	2.78118	-2.22909	0.84148
H	3.26241	1.88904	-0.25441
H	5.20924	-2.41867	1.19012
H	5.69224	1.68306	0.05217
H	6.67982	-0.46422	0.78814
H	1.26001	2.51398	1.46390
H	-0.07629	1.02188	-2.31882
H	0.60078	4.79034	0.80850
H	-0.71493	3.31939	-2.99511
H	-0.38781	5.20451	-1.42307
H	-4.24083	1.82185	-0.51039
H	-3.60841	2.69759	0.88110
H	-2.50426	2.15869	-0.38832
H	-4.79898	0.93886	2.33527
H	-5.28469	0.02328	0.91192
H	-4.35445	-0.76507	2.18956
H	-1.39998	1.57977	1.66014

CAAC_PPh2_oxo_s (*NImag* = 0)

N	-2.76215	-0.27921	-0.04346
C	-1.58354	-0.77983	0.24623
C	-2.09051	0.84050	1.92420
H	-2.41108	0.92871	2.97238
C	-1.19538	-0.39527	1.67554
C	0.32631	-0.11595	1.79492
H	0.52565	0.84330	2.29510
H	0.81704	-0.90614	2.38137
C	-1.56515	-1.58741	2.58383
H	-0.98693	-2.47607	2.29243
H	-1.33928	-1.34021	3.63341
H	-2.63010	-1.84621	2.50872
Pd	-0.38181	-1.66771	-0.98750
C	-3.49531	-0.59077	-1.26788
H	-2.95229	-1.40470	-1.78649
H	-4.50928	-0.92984	-1.00964
H	-3.57168	0.30441	-1.90440
P	1.06926	-0.19045	0.08718
C	2.87354	-0.34591	0.35416
C	3.64470	-0.81759	-0.72049
C	3.51067	-0.02143	1.56253
C	5.02888	-0.94512	-0.59535
H	3.14996	-1.09319	-1.65577
C	4.89600	-0.15693	1.68820
H	2.93120	0.33737	2.41573
C	5.65706	-0.61476	0.60952
H	5.61845	-1.31288	-1.43839
H	5.38210	0.09591	2.63366
H	6.73983	-0.72077	0.70988
C	0.86749	1.55429	-0.48532
C	1.53389	2.62424	0.13302
C	-0.02423	1.81868	-1.53520
C	1.29646	3.93682	-0.27827
H	2.24958	2.43079	0.93576
C	-0.26099	3.13384	-1.94735
H	-0.52922	0.98186	-2.02525
C	0.39465	4.19433	-1.31712
H	1.82015	4.76291	0.20912
H	-0.95548	3.32814	-2.76843
H	0.21136	5.22207	-1.63987
C	-3.29549	0.70281	0.96025
C	-3.60121	2.03286	0.26404
H	-4.46126	1.95163	-0.41699
H	-3.84513	2.79238	1.02252
H	-2.72783	2.38293	-0.30629
C	-4.55805	0.15455	1.63616
H	-4.91244	0.87010	2.39335
H	-5.36977	0.01132	0.90776
H	-4.37085	-0.80690	2.13304
H	-1.52752	1.75020	1.66867
O	-1.45560	-2.79540	-1.93166

dmpp_oxo_s (*NImag* = 0)

C	-4.68610	1.39613	1.08857
C	-6.13265	0.92806	0.86664

C	-6.42516	-0.55327	1.21021
H	-4.66885	2.48386	1.21656
H	-6.78306	1.54082	1.49560
H	-6.42772	1.14660	-0.16044
H	-5.60822	-0.98596	1.79074
H	-7.31690	-0.59871	1.84408
P	-3.51604	0.92410	-0.27761
P	-6.73574	-1.76431	-0.13774
Pd	-4.04261	-1.08788	-0.82818
O	-5.60287	-2.66319	-0.57359
C	-1.91783	1.44162	0.47507
H	-1.13040	1.37702	-0.27606
H	-1.96031	2.46370	0.86109
H	-1.66593	0.75895	1.28628
C	-7.52357	-0.87875	-1.50695
H	-7.89880	-1.61599	-2.21715
H	-8.35153	-0.26103	-1.15558
H	-6.78124	-0.25788	-2.00502
C	-8.05823	-2.81205	0.53990
H	-8.95472	-2.23557	0.77059
H	-8.30072	-3.58598	-0.18807
H	-7.69877	-3.29417	1.44965
C	-3.78828	2.31892	-1.45142
H	-4.79590	2.25765	-1.86246
H	-3.65490	3.29034	-0.96759
H	-3.08686	2.23120	-2.28109
H	-4.29572	0.96104	2.01182

dmpp_oxo_t (*NImag* = 0)

C	-4.74736	1.43390	1.21304
C	-6.26591	1.37954	0.99835
C	-6.91490	0.00443	1.20671
H	-4.43256	2.47665	1.31744
H	-6.72588	2.07573	1.70414
H	-6.51688	1.76381	0.00577
H	-6.55880	-0.43111	2.14423
H	-7.99942	0.11874	1.30007
P	-3.71450	0.65868	-0.10990
P	-6.56034	-1.22453	-0.11945
Pd	-4.29636	-1.52294	-0.57184
O	-4.23590	-3.43032	-0.97796
C	-2.03362	1.10314	0.48128
H	-1.30542	0.82251	-0.27950
H	-1.94260	2.17148	0.69344
H	-1.80951	0.53576	1.38450
C	-7.62639	-0.64288	-1.49438
H	-7.57933	-1.37153	-2.30345
H	-8.66580	-0.52115	-1.17930
H	-7.25513	0.30661	-1.87850
C	-7.44975	-2.71082	0.46281
H	-8.51066	-2.51739	0.63824
H	-7.33036	-3.49338	-0.28564
H	-6.98534	-3.06344	1.38342
C	-3.93153	1.84451	-1.49564
H	-4.94205	1.77152	-1.89559
H	-3.74346	2.87292	-1.17845
H	-3.23863	1.58426	-2.29545
H	-4.49121	0.93058	2.14949

ethylenediamine (*NImag* = 0)

C	1.45364	0.44880	0.48814
C	0.43997	1.23254	-0.33175
H	1.25039	0.58153	1.55077
H	0.66184	1.12366	-1.39339
N	1.40258	-0.98806	0.20108
N	-0.92897	0.75949	-0.11153
Pd	-0.89641	-1.66405	0.00556
H	0.53485	2.30372	-0.09616
H	2.46007	0.85502	0.30427
C	2.04248	-1.75489	1.27213
H	1.96428	-2.81688	1.05046
H	3.10564	-1.49005	1.38250
H	1.53027	-1.56514	2.21381
C	2.03047	-1.30530	-1.08389
H	3.09513	-1.02370	-1.09071
H	1.94275	-2.37327	-1.26998
H	1.52543	-0.78455	-1.89442
C	-1.46080	1.21609	1.17391
H	-1.50529	2.31549	1.22328
H	-2.46159	0.81247	1.31016
H	-0.84296	0.85386	1.99275
C	-1.81157	1.18350	-1.19916
H	-2.81022	0.78810	-1.02676
H	-1.86992	2.28072	-1.27320
H	-1.44614	0.78379	-2.14364
C	-2.00140	-3.11880	-0.03533
O	-2.69854	-4.03437	-0.06183

propylenediamine_CO (*NImag* = 0)

C	-4.79141	1.45303	1.05637
C	-6.29921	1.39442	0.84038
C	-6.94878	0.03412	1.06650
H	-4.55459	0.96053	2.00200
H	-4.48946	2.50850	1.16234
H	-6.74405	2.07619	1.57020
H	-6.57207	1.80235	-0.13370
H	-6.58468	-0.37387	2.01195
H	-8.03696	0.17513	1.17836
N	-3.96309	0.81876	0.02180
N	-6.69952	-0.98173	0.03515
Pd	-4.39462	-1.50121	-0.43324
C	-3.43124	-2.97771	-0.92282
O	-2.82431	-3.90838	-1.22632
C	-7.24144	-2.26654	0.49724
H	-8.32344	-2.19479	0.69332
H	-7.06340	-3.02653	-0.25923
H	-6.73516	-2.57007	1.41144
C	-7.33787	-0.62521	-1.23403
H	-7.14730	-1.41126	-1.96157
H	-8.42607	-0.50238	-1.11554
H	-6.92671	0.30114	-1.62650
C	-2.56540	0.81408	0.47470
H	-1.94167	0.34820	-0.28383
H	-2.20509	1.83817	0.66368
H	-2.47931	0.23348	1.39102
C	-4.04799	1.54110	-1.24982
H	-5.06377	1.52543	-1.63594
H	-3.73259	2.59040	-1.13705
H	-3.40450	1.05423	-1.97945

Bipy_CO (*NImag* = 0)

N	0.69964	-1.38128	-0.08363

C	1.89058	-0.77009	-0.00922
C	3.07112	-1.49514	0.14600
C	3.00825	-2.87652	0.24026
C	1.77048	-3.49989	0.17492
C	0.64217	-2.70887	0.00869
C	1.86576	0.70916	-0.11743
C	2.94766	1.51007	0.24522
C	2.84420	2.88587	0.11289
C	1.66410	3.42816	-0.37520
C	0.62854	2.56461	-0.70427
N	0.72456	1.24214	-0.57681
Pd	-1.14343	-0.13140	-0.75446
H	3.85034	1.07045	0.64304
H	3.67162	3.52416	0.39347
H	1.54077	4.49528	-0.49635
H	-0.31517	2.93347	-1.08300
H	4.02698	-0.99322	0.17356
H	3.91417	-3.45668	0.35642
H	-0.34675	-3.14255	-0.05495
H	1.67496	-4.57434	0.24598
C	-2.92764	-0.19071	-1.16254
O	-4.04901	-0.22777	-1.41905

bisbenzimi_CO (*NImag* = 0)

N	2.54132	-0.11357	0.69000
C	1.45704	-0.41839	-0.06795
C	2.07184	1.75689	-0.43753
C	-0.00093	0.83467	-1.57838
H	-0.00181	1.78611	-2.10161
H	-0.00112	0.02289	-2.30189
C	-1.45528	-0.41924	-0.06502
N	-1.19537	0.72945	-0.76663
Pd	0.00158	-1.97686	0.04289
C	3.18094	-1.05342	1.58733
H	4.23764	-1.15664	1.33475
H	3.09116	-0.71738	2.62168
H	2.67512	-2.00858	1.46808
C	-3.17728	-1.05621	1.59147
H	-3.08809	-0.71887	2.62545
H	-4.23377	-1.16200	1.33914
H	-2.66942	-2.01039	1.47311
N	-2.53984	-0.11589	0.69305
C	-2.95446	1.19811	0.50333
C	-2.07457	1.75433	-0.43659
C	-2.20147	3.07168	-0.84980
C	-3.98819	1.94104	1.05372
C	-3.23676	3.81847	-0.29488
H	-1.52666	3.51161	-1.57137
C	-4.11606	3.26307	0.63804
H	-4.66668	1.51343	1.77884
H	-3.36203	4.85073	-0.59281
H	-4.91037	3.87303	1.04658
C	2.95317	1.20149	0.50152
C	3.98561	1.94592	1.05229
C	4.11067	3.26864	0.63792
H	4.66520	1.51892	1.77675
C	2.19593	3.07491	-0.84941
C	3.22991	3.82323	-0.29408
H	4.90392	3.87977	1.04680
H	1.51995	3.51423	-1.57026
H	3.35296	4.85606	-0.59097
N	1.19470	0.73052	-0.76850
C	0.00044	-3.86935	0.13476
O	-0.00074	-5.01628	0.23806

bisCAAC_CO (*NImag* = 0)

C	2.22811	-2.11425	-0.33240
N	1.73242	-0.79397	-0.83962
C	1.27463	0.05422	0.05308
C	1.32087	-0.62335	1.40905
C	1.67281	-2.10168	1.10313
C	1.83760	-0.46093	-2.25000
Pd	0.22362	1.82231	-0.14757
C	0.00006	-0.41453	2.19362
C	-1.36347	-0.36018	1.46783
C	-1.34577	0.53031	0.23339
N	-2.12453	-0.04088	-0.66870
C	-2.66496	-1.39592	-0.35079
C	-1.90003	-1.71583	0.94756
C	2.44213	0.03872	2.23395
C	-2.39914	0.56794	-1.95772
C	-2.31844	-2.37701	-1.47141
C	-4.18452	-1.35092	-0.16315
C	-2.35635	0.27154	2.46538
C	3.76009	-2.13947	-0.37583
C	1.67354	-3.27123	-1.15920
C	0.66807	3.64604	-0.44262
O	0.94734	4.74174	-0.66088
H	-0.05465	-1.17542	2.97954
H	0.09725	0.54375	2.70529
H	2.26753	1.11066	2.31306
H	2.46844	-0.39340	3.23736
H	3.41974	-0.10831	1.77585
H	2.82480	-0.71914	-2.63536
H	1.08531	-0.99678	-2.83220
H	1.67241	0.60893	-2.34756
H	-2.53177	-2.22334	1.67738
H	-1.07583	-2.38570	0.71392
H	-2.88508	-2.17057	-2.38032
H	-1.25625	-2.32591	-1.71046
H	-2.55138	-3.39623	-1.15854
H	-4.68673	-1.04500	-1.08192
H	-4.55518	-2.34324	0.09897
H	-4.47059	-0.65947	0.62667
H	-2.40273	-0.32728	3.37857
H	-2.03814	1.28130	2.72200
H	-3.36071	0.33732	2.05052
H	-2.15109	1.62386	-1.88506
H	-1.79226	0.11812	-2.74680
H	-3.45165	0.45017	-2.22035
H	2.39465	-2.50485	1.81347
H	0.78731	-2.73161	1.15916
H	4.12726	-3.07188	0.05531
H	4.12870	-2.08225	-1.40082
H	4.18575	-1.30955	0.18533
H	1.97828	-4.21800	-0.71054
H	0.58501	-3.24814	-1.18914
H	2.04987	-3.25506	-2.18314

bisDAC_CO (*NImag* = 0)
not stable

bisimine_CO (*NImag* = 0)

C	1.48347	2.79084	0.16174
N	1.52030	1.34873	0.14694
Pd	-0.28561	-0.00557	0.77748
N	1.51899	-1.34806	0.15147
C	1.47948	-2.79039	0.16318
C	2.56695	-0.73738	-0.21552
C	2.56882	0.73625	-0.21477
H	3.47282	-1.26029	-0.53370
H	3.47795	1.25699	-0.52744
H	1.23864	3.12044	1.17260
H	2.42521	3.24897	-0.16146
H	0.66893	3.12304	-0.48371
H	2.41837	-3.24920	-0.16719
H	1.24051	-3.12202	1.17474
H	0.66030	-3.11938	-0.47795
C	-2.02343	-0.00038	1.38258
O	-3.10888	0.00318	1.76065

bisMIC_CO_diazo (*NImag* = 0)

N	-3.02528	-1.95665	0.19218
C	-2.67459	-0.65764	0.56711
C	-1.50274	-0.29602	-0.07031
N	-1.20558	-1.44168	-0.82355
C	-2.10747	-2.42014	-0.65883
Pd	-0.00643	1.23054	0.27709
C	-0.00114	3.02764	0.81164
O	0.00339	4.12651	1.17685
C	-3.49387	0.13907	1.51491
C	-0.01349	-1.52739	-1.64212
N	1.18156	-1.44516	-0.82752
C	1.48449	-0.30034	-0.07531
C	2.65730	-0.66539	0.55839
N	3.00306	-1.96534	0.18224
C	2.08119	-2.42622	-0.66578
C	3.48189	0.12891	1.50361
C	4.17927	-2.69799	0.61566
C	-4.20212	-2.68593	0.62950
H	-0.01580	-2.46341	-2.19910
H	-0.01338	-0.68115	-2.32491
H	-4.53021	0.25283	1.18171
H	-3.51188	-0.29424	2.51967
H	-3.03791	1.12567	1.58096
H	4.51739	0.23999	1.16694
H	3.02882	1.11672	1.57143
H	3.50215	-0.30467	2.50822
H	4.17000	-3.69383	0.17834
H	5.08260	-2.17702	0.29989
H	4.18477	-2.78377	1.70161
H	-5.10499	-2.16211	0.31715
H	-4.19742	-3.68162	0.19177
H	-4.20404	-2.77214	1.71543
H	2.09115	-3.39416	-1.13558
H	-2.12174	-3.38805	-1.12858

bisNHC_CO (*NImag* = 0)

C	-1.46436	-0.56340	0.05647
N	-1.20057	-1.75077	-0.56437
C	-2.14377	-2.72110	-0.26583
C	-3.03709	-2.12935	0.55789
N	-2.60576	-0.82356	0.73991
C	-0.00243	-1.92922	-1.35935
N	1.19651	-1.75299	-0.56505
C	2.13837	-2.72492	-0.26747
C	3.03330	-2.13481	0.55568
N	2.60423	-0.82837	0.73835
C	1.46274	-0.56620	0.05584
Pd	0.00071	1.00720	0.19181
C	3.27711	0.15467	1.56744
C	-3.27638	0.16075	1.56931
H	2.77036	1.10470	1.41272
H	3.21224	-0.12348	2.62010
H	4.32549	0.23610	1.27815
H	-2.76840	1.10999	1.41376
H	-4.32493	0.24359	1.28103
H	-3.21087	-0.11709	2.62201
H	-0.00198	-1.19124	-2.15856
H	-0.00347	-2.92984	-1.78511
H	2.10279	-3.72273	-0.66746
H	-2.11014	-3.71906	-0.66561
H	3.92362	-2.52609	1.01469
H	-3.92769	-2.51907	1.01768
C	0.00283	2.88340	0.37136
O	0.00413	4.02798	0.51650

bisNHCS_CO (*NImag* = 0)

C	0.18183	-1.41323	-0.09444
N	1.26055	-1.18249	-0.88642
C	2.49931	-1.74299	-0.32713
C	1.94004	-2.76357	0.66628
N	0.56663	-2.27757	0.84849
C	1.24459	-0.03976	-1.78182
N	1.06165	1.23592	-1.11616
C	2.19114	2.04645	-0.63718
C	1.46065	3.13236	0.15393
N	0.16592	2.48909	0.40773
C	-0.06301	1.43141	-0.37543
Pd	-1.52142	-0.12604	-0.23267
C	-3.40015	-0.31743	-0.30001
O	-4.54699	-0.43327	-0.28688
C	-0.83504	3.13132	1.22426
C	-0.32645	-2.91143	1.78743
H	-1.74062	2.53111	1.17536
H	-0.50006	3.20543	2.26212
H	-1.04740	4.14035	0.85503
H	-1.30786	-2.45720	1.67260
H	-0.39874	-3.98619	1.58930
H	0.02563	-2.77106	2.81274
H	0.41562	-0.17214	-2.47451
H	2.17723	-0.01743	-2.34425
H	1.31967	4.04687	-0.43344
H	2.84912	1.45755	0.01202
H	3.08584	-0.97001	0.18205
H	1.92968	-3.77855	0.25309
H	2.78289	2.44198	-1.46245
H	1.96333	3.39777	1.08474
H	3.12185	-2.19521	-1.09902
H	2.48372	-2.78530	1.61154

CAAC_benzimi_CO (*NImag* = 0)

N	-1.87340	1.33461	-0.76826
C	-1.65392	0.54260	0.26448

C	-0.50574	2.66496	0.59275
C	-1.32896	2.72510	-0.70660
H	-0.63072	3.56633	1.19320
C	-0.97269	1.39242	1.33286
C	0.20621	0.63008	1.96454
H	0.78901	1.29978	2.59664
H	-0.17661	-0.17841	2.58657
C	-1.98673	1.69442	2.44450
H	-2.36886	0.76713	2.87058
H	-1.52120	2.28522	3.23804
H	-2.83594	2.25680	2.05821
C	0.64529	-0.94119	0.13591
N	1.08926	0.04715	0.96680
Pd	-1.38501	-1.51771	0.17937
C	-2.53310	0.86640	-1.97453
H	-3.19986	1.63377	-2.36997
H	-1.80331	0.60613	-2.74392
H	-3.10077	-0.02499	-1.71879
C	1.64084	-2.17173	-1.76689
H	1.68960	-1.69996	-2.75012
H	2.47905	-2.86229	-1.66047
H	0.70340	-2.71335	-1.66674
N	1.67683	-1.17416	-0.71885
C	2.76100	-0.34932	-0.44569
C	-0.44140	3.00371	-1.91978
H	-1.01682	3.03362	-2.84592
H	0.04959	3.97114	-1.80197
H	0.33186	2.23962	-2.01396
C	-2.47043	3.74256	-0.63761
H	-2.06542	4.75258	-0.55843
H	-3.09203	3.70597	-1.53325
H	-3.10774	3.55907	0.22654
H	0.55211	2.58304	0.34138
C	2.38078	0.44403	0.64836
C	3.24350	1.39116	1.18141
C	4.00915	-0.21863	-1.03638
C	4.49505	1.52342	0.58760
H	2.95982	2.00658	2.02387
C	4.87246	0.73272	-0.50141
H	4.30138	-0.82926	-1.87964
H	5.19082	2.25360	0.97868
H	5.85439	0.86215	-0.93624
C	-2.17947	-3.24398	0.19159
O	-2.63685	-4.29975	0.16120

CAAC_MIC_CO (*NImag* = 0)

N	-0.47197	1.61197	1.89382
C	-0.03066	1.45741	0.62272
C	-2.39436	1.92137	0.76605
C	-1.85552	1.85374	1.98285
H	-3.42082	2.15847	0.53178
H	-2.31764	1.98551	2.95006
C	-1.27590	1.75004	-0.23899
C	-1.52795	0.59970	-1.23205
H	-2.50484	0.70236	-1.70094
H	-0.76481	0.61690	-2.00825
C	-1.05486	3.03780	-1.05280
H	-0.18399	2.92544	-1.69898
H	-1.93161	3.26934	-1.66358
H	-0.87488	3.87644	-0.38194
C	-0.32925	-1.17432	0.01477
C	-0.79103	-2.39602	0.57563
N	-1.43206	-0.70197	-0.58336
N	-2.48820	-1.54004	-0.37183
N	-2.08393	-2.58770	0.31294
C	-3.78917	-1.43716	-0.98897
H	-4.27527	-0.50402	-0.70538
H	-3.71662	-1.50229	-2.07643
H	-4.36957	-2.27482	-0.61504
Pd	1.36886	0.07881	0.08544
C	0.34597	1.35291	3.06057
H	0.07520	2.03272	3.87001
H	0.22196	0.32193	3.40238
H	1.38708	1.50089	2.78095
C	0.00466	-3.36998	1.36937
H	-0.56369	-4.28312	1.53956
H	0.93133	-3.61132	0.84726
H	0.28429	-2.94590	2.33597
C	3.10933	-0.57361	-0.36099
O	4.12908	-1.03659	-0.61570

CAAC_NHC_CO (*NImag* = 0)

N	1.33035	-1.43637	1.01467
C	0.62239	-0.94667	0.01115
C	2.80325	0.16892	0.13712
C	2.74605	-1.05787	1.05663
H	3.36618	-1.88584	0.69605
C	1.61451	-0.16412	-0.84529
C	0.98037	1.04199	-1.56158
H	1.76572	1.66962	-1.98510
H	0.38059	0.66359	-2.38953
C	2.05796	-1.12746	-1.96601
H	1.17917	-1.50366	-2.48787
H	2.70718	-0.62592	-2.68659
H	2.59549	-1.98593	-1.56505
C	-0.90797	1.42147	-0.00489
N	0.12361	1.89941	-0.74987
Pd	-1.41985	-0.63660	-0.03633
C	0.80620	-2.33491	2.02129
H	1.45986	-3.20590	2.12261
H	0.74274	-1.83443	2.99114
H	-0.18900	-2.64543	1.71015
C	-2.59290	2.51033	1.43985
H	-2.29823	2.77429	2.45677
H	-3.35353	3.20952	1.09162
H	-2.99185	1.49893	1.42539
N	-1.44732	2.53519	0.55080
C	-0.78156	3.68169	0.14774
C	0.20686	3.27870	-0.68235
H	-1.06596	4.66652	0.47243
H	0.94741	3.84648	-1.21654
H	3.05621	-0.83344	2.07980
C	2.52174	1.41560	0.98704
H	3.30189	1.52302	1.74349
H	2.52582	2.32325	0.38546
H	1.55950	1.35367	1.49414
C	4.16350	0.33210	-0.53729
H	4.14766	1.15741	-1.25291
H	4.93063	0.56466	0.20471
H	4.47609	-0.56698	-1.06666
C	-3.17090	-1.34247	-0.18942
O	-4.25246	-1.73792	-0.23833

CAAC_PMe2_CO (*NImag* = 0)

N	-2.69841	-0.14493	0.00269
C	-1.66643	-0.78844	0.49151
C	-2.07863	1.21362	1.80844
H	-2.47088	1.46119	2.79503
C	-1.28095	-0.11171	1.80847
C	0.25173	0.11713	1.86541
H	0.49357	1.07506	2.32993
H	0.70873	-0.66256	2.47652
C	-1.69927	-1.03415	2.96485
H	-1.19500	-1.99629	2.87865
H	-1.43437	-0.57994	3.92241
H	-2.77352	-1.21991	2.96133
Pd	-0.27006	-1.98261	-0.49346
C	-3.33390	-0.51533	-1.25031
H	-2.99213	-1.51337	-1.51317
H	-4.41930	-0.50497	-1.14576
H	-3.05156	0.18015	-2.04216
P	1.01485	-0.08384	0.18512
C	2.78641	0.25211	0.48402
C	3.60354	0.46932	-0.63171
C	3.37779	0.22557	1.74823
C	4.96582	0.67212	-0.48527
H	3.16323	0.47817	-1.62143
C	4.74781	0.42061	1.89370
H	2.77751	0.05473	2.63060
C	5.54545	0.64742	0.78099
H	5.58032	0.84387	-1.35984
H	5.18882	0.39721	2.88231
H	6.61076	0.79954	0.89553
C	0.52226	1.44618	-0.70227
C	0.77733	2.72461	-0.20226
C	-0.11535	1.31935	-1.93609
C	0.39030	3.85104	-0.91451
H	1.29276	2.83981	0.74320
C	-0.49494	2.44652	-2.65695
H	-0.30808	0.32660	-2.32471
C	-0.24637	3.71381	-2.14518
H	0.59203	4.83735	-0.51622
H	-0.98387	2.33400	-3.61626
H	-0.54210	4.59302	-2.70297
C	-3.20609	1.03437	0.77799
C	-3.34791	2.25846	-0.12354
H	-4.14291	2.13111	-0.85984
H	-3.59984	3.12729	0.48672
H	-2.41680	2.46911	-0.64680
C	-4.55319	0.69058	1.41963
H	-4.90362	1.53451	2.01548
H	-5.31277	0.48279	0.66479
H	-4.47274	-0.17913	2.07002
H	-1.43391	2.03337	1.49480
C	0.25343	-3.61633	-1.33295
O	0.61769	-4.56147	-1.87097

dmpe_CO (*NImag* = 0)

C	2.70511	1.56411	1.56359
P	1.85573	1.73882	-0.05787
C	3.25914	1.54217	-1.22630
C	1.02883	0.07655	-0.21284
C	-0.29918	0.04398	0.54785
P	-1.35776	1.51144	0.11537
C	-2.08042	0.96883	-1.48581
Pd	0.12209	3.34091	-0.07691
C	-0.01237	5.25818	-0.17715
C	-2.77765	1.26631	1.25555
H	-0.83849	-0.88841	0.35931
H	-0.11615	0.09699	1.62447
H	0.85299	-0.08624	-1.27962
H	1.69806	-0.71889	0.12619
H	3.31482	0.65840	1.60856
H	3.33992	2.43441	1.72866
H	1.96393	1.53890	2.36208
H	3.92848	2.39630	-1.12273
H	3.82127	0.62223	-1.04658
H	2.87900	1.53631	-2.24792
H	-3.55553	1.99048	1.01347
H	-3.19219	0.25733	1.18684
H	-2.45313	1.45202	2.27953
H	-2.53680	-0.02126	-1.41234
H	-2.83440	1.69089	-1.79853
H	-1.30294	0.95106	-2.24917
O	-0.09188	6.39938	-0.23295

dmpp_CO (*NImag* = 0)

C	-4.78521	1.46106	1.18570
C	-6.29422	1.38233	0.91246
C	-6.95799	0.02658	1.19418
H	-4.56241	0.99248	2.14843
H	-4.48379	2.51029	1.26776
H	-6.78447	2.12860	1.54296
H	-6.50203	1.69101	-0.11608
H	-6.60930	-0.35894	2.15639
H	-8.04080	0.16197	1.28174
P	-3.70966	0.63991	-0.07767
P	-6.63253	-1.28959	-0.06639
Pd	-4.35048	-1.57063	-0.50772
C	-3.31363	-3.14344	-0.90700
O	-2.69634	-4.07997	-1.14140
C	-7.67913	-2.64478	0.60293
H	-8.69867	-2.31427	0.81859
H	-7.70895	-3.45774	-0.12250
H	-7.22464	-3.02899	1.51618
C	-7.67811	-0.70411	-1.46231
H	-7.69339	-1.46824	-2.23928
H	-8.70211	-0.49751	-1.14139
H	-7.24981	0.19975	-1.89451
C	-2.04867	1.07116	0.58175
H	-1.29317	0.77531	-0.14607
H	-1.94993	2.13943	0.79224
H	-1.87006	0.50614	1.49665
C	-3.84354	1.82671	-1.47699
H	-4.84459	1.78595	-1.90525
H	-3.63051	2.85071	-1.16028
H	-3.13760	1.53840	-2.25567

Bipy_Cl2 (*NImag* = 0)

N	-0.07871	-1.20687	0.08627
C	0.67525	-0.08549	0.02500
C	2.06197	-0.16685	-0.03450
C	2.67274	-1.41078	-0.03074
C	1.88260	-2.54877	0.03254

C	0.50545	-2.40743	0.09013
C	-0.07284	1.18015	0.02680
C	0.52445	2.43438	-0.03082
C	-0.27083	3.56923	-0.02369
C	-1.64870	3.42558	0.04101
C	-2.18883	2.15082	0.09660
N	-1.41871	1.06020	0.08948
Pd	-2.10890	-0.87747	0.16816
Cl	-4.31497	-0.27356	0.25110
H	1.59884	2.52480	-0.08073
H	0.18196	4.55059	-0.06812
H	-2.30606	4.28320	0.04890
H	-3.25280	1.95879	0.14856
H	2.65916	0.73090	-0.08327
H	3.75075	-1.48719	-0.07668
H	-0.17574	-3.24707	0.14118
H	2.31699	-3.53821	0.03781
Cl	-2.64331	-3.10157	0.24702

bisCAAC_Cl2

C	-2.50339	-0.23869	1.07920
C	-3.46979	0.83154	0.51922
C	-2.63095	1.77950	-0.35996
N	-1.37728	0.97019	-0.50725
C	-1.33858	-0.18115	0.11097
C	-0.22045	1.42259	-1.27160
N	0.98743	1.32053	-0.45298
C	1.34956	0.17628	0.07882
C	2.65835	0.37075	0.81638
C	2.77856	1.91072	0.91047
C	1.89973	2.48906	-0.21078
Pd	0.17619	-1.39884	-0.18439
Cl	1.95713	-2.92034	-0.47555
C	1.13473	3.71717	0.27002
C	2.65704	2.81671	-1.49857
C	-2.36445	3.12402	0.31183
C	-3.25069	1.98577	-1.74088
C	3.82716	-0.21981	0.00286
C	2.60272	-0.30276	2.18857
C	-3.19738	-1.58388	1.25603
C	-1.89601	0.18388	2.43773
H	-2.50492	-2.34048	1.61728
H	-4.00631	-1.46541	1.98198
H	-3.60441	-1.94814	0.31698
H	3.72349	-1.29708	-0.08288
H	3.87418	0.18997	-1.00553
H	4.75911	0.02750	0.51586
H	-0.10760	0.80083	-2.15876
H	-0.36651	2.45556	-1.56756
H	-4.22582	0.33929	-0.09184
H	3.81045	2.24810	0.82770
H	-3.98550	1.37248	1.31175
H	2.39400	2.25071	1.87368
H	-1.17456	-0.56051	2.77248
H	-1.38766	1.14725	2.38541
H	-2.69389	0.25404	3.17870
H	1.76377	0.07112	2.77896
H	2.49050	-1.37916	2.06754
H	3.52497	-0.10194	2.73798
H	1.85473	4.47215	0.58801
H	0.52472	4.16567	-0.51481
H	0.49885	3.47783	1.12117
H	3.34865	3.64109	-1.32188
H	3.22431	1.96142	-1.85745
H	1.96826	3.12593	-2.28776
H	-1.89303	3.00050	1.28519
H	-1.73591	3.76915	-0.30102
H	-3.31340	3.64050	0.45863
H	-3.39614	1.02934	-2.24292
H	-4.22105	2.47319	-1.63804
H	-2.62820	2.62129	-2.37449
Cl	-1.37523	-3.01030	-0.96991

bisCAAC_diastereo_Cl2 (*NImag* = 0)

C	-2.37732	-2.12907	0.90817
C	-1.01118	-1.59991	0.44022
C	-1.36416	-0.23931	-0.15449
N	-2.61595	-0.27081	-0.51681
C	-3.42260	-1.41782	0.02657
C	-0.00288	-1.49452	1.59220
C	1.40440	-0.93811	1.24509
C	1.36197	-0.06842	0.01174
N	2.36079	-0.34146	-0.76933
C	3.28223	-1.42152	-0.28027
C	2.45643	-2.01274	0.88117
C	4.60363	-0.79927	0.17487
C	3.53251	-2.44584	-1.38419
C	2.62883	0.35442	-2.02137
Pd	-0.05939	1.26442	-0.10941
Cl	1.70058	2.84642	0.14564
C	1.87930	-0.07303	2.43064
C	-0.46643	-2.47685	-0.70632
C	-3.28551	0.72253	-1.34817
C	-3.98271	-2.29823	-1.09230
C	-4.57036	-0.85720	0.86804
H	0.11795	-2.48005	2.05092
H	-0.44650	-0.84896	2.35114
H	1.20315	0.76518	2.58631
H	1.90483	-0.68907	3.33220
H	2.87124	0.33926	2.26010
H	3.69534	0.55113	-2.11033
H	2.30492	-0.25656	-2.86526
H	2.09852	1.30064	-2.00512
H	-2.53722	-1.85507	1.95179
H	-2.45360	-3.21347	0.83457
H	-4.70893	-1.75712	-1.69844
H	-3.20244	-2.67579	-1.75023
H	-4.49756	-3.15250	-0.65132
H	-5.32791	-0.37404	0.25112
H	-5.05077	-1.67444	1.40828
H	-4.19816	-0.13207	1.59203
H	-2.54334	1.38562	-1.77209
H	-3.82923	0.20052	-2.13625
H	-3.96977	1.32344	-0.75577
H	3.08893	-2.26823	1.73029
H	1.96842	-2.92787	0.54957
H	5.23936	-1.57150	0.60971
H	5.14386	-0.35884	-0.66345
H	4.44226	-0.02041	0.91690
H	4.11025	-3.27669	-0.97728
H	2.59530	-2.84468	-1.77408
H	4.10121	-2.02035	-2.21149

H	-0.20772	-3.46449	-0.31991
H	-1.20554	-2.60552	-1.49326
H	0.41635	-2.04419	-1.16770
Cl	-1.73599	2.91462	0.23162

bisCAAC_Cl2 (*NImag* = 0)

N	1.98014	-0.58323	-0.75400
C	1.25828	-0.00684	0.16020
C	1.34263	-0.78774	1.44658
C	0.00001	-0.82478	2.21811
H	0.00002	-1.69651	2.87842
H	-0.00000	0.04634	2.87172
C	2.35984	-0.04841	2.34432
H	2.04491	0.98222	2.49988
H	2.42509	-0.55466	3.30960
H	3.35181	-0.02390	1.89888
C	-1.25828	-0.00686	0.16021
Cl	-1.79637	3.01426	-0.30012
Cl	1.79633	3.01429	-0.30011
Pd	-0.00001	1.47559	-0.05894
C	2.17182	-0.03625	-2.09401
H	3.18123	-0.25695	-2.43515
H	1.45647	-0.48059	-2.78754
H	2.03142	1.04059	-2.04575
N	-1.98014	-0.58324	-0.75399
C	-1.89158	-2.15765	0.98809
H	-2.57393	-2.59388	1.71676
H	-1.07330	-2.86314	0.84390
C	-2.58692	-1.90015	-0.36655
C	-2.21763	-2.95983	-1.40238
H	-2.69650	-2.77377	-2.36404
H	-1.13823	-2.99672	-1.55359
H	-2.54538	-3.93892	-1.05076
C	-4.10799	-1.77965	-0.26166
H	-4.55245	-1.53969	-1.22791
H	-4.52736	-2.73111	0.06745
H	-4.40369	-1.00947	0.44689
C	-1.34261	-0.78777	1.44658
C	-2.35985	-0.04849	2.34431
H	-2.42509	-0.55474	3.30959
H	-2.04497	0.98215	2.49988
H	-3.35182	-0.02402	1.89886
C	-2.17183	-0.03624	-2.09399
H	-2.03165	1.04063	-2.04567
H	-1.45631	-0.48039	-2.78748
H	-3.18115	-0.25714	-2.43523
C	1.89164	-2.15761	0.98811
C	2.58694	-1.90013	-0.36654
H	2.57401	-2.59380	1.71678
H	1.07337	-2.86312	0.84395
C	4.10802	-1.77961	-0.26170
H	4.52740	-2.73106	0.06743
H	4.55244	-1.53969	-1.22797
H	4.40374	-1.00940	0.44681
C	2.21764	-2.95984	-1.40235
H	2.54541	-3.93891	-1.05069
H	1.13824	-2.99673	-1.55354
H	2.69650	-2.77380	-2.36402

bisDAC_Cl2 (*NImag* = 0)

C	-2.21110	2.73420	-0.46567
N	-1.18487	1.80980	-0.67235
C	-1.36682	0.65099	0.03838
N	-2.51350	0.75885	0.73669
C	-3.14435	1.99595	0.51876
C	0.00015	2.04531	-1.47590
N	1.18502	1.80976	-0.67214
C	2.21125	2.73412	-0.46528
C	3.14431	1.99582	0.51929
N	2.51338	0.75876	0.73711
C	1.36681	0.65093	0.03862
C	3.04205	-0.21755	1.68893
C	-3.04234	-0.21743	1.68843
H	-2.66157	-1.19637	1.41667
H	-2.74138	0.05919	2.69848
H	-4.12689	-0.20241	1.61928
H	2.66108	-1.19644	1.41726
H	4.12660	-0.20277	1.61975
H	2.74117	0.05922	2.69896
H	0.00022	1.37928	-2.33704
H	0.00020	3.08401	-1.79868
Pd	-0.00002	-0.75812	-0.12620
Cl	-1.68900	-2.31244	-0.56786
Cl	1.68897	-2.31251	-0.56752
O	-4.15872	2.38505	1.00849
O	-2.31741	3.82570	-0.93403
O	2.31771	3.82560	-0.93366
O	4.15864	2.38487	1.00916

Bisimine_Cl2 (*NImag* = 0)

C	2.98228	0.48614	-0.00411
N	1.60684	0.94336	-0.00254
Pd	-0.05509	-0.26001	-0.00092
Cl	-2.04280	-1.37434	0.00128
N	-0.92513	1.59866	0.00012
C	-2.34959	1.86599	0.00177
C	-0.07513	2.55911	-0.00074
C	1.32954	2.19558	-0.00199
Cl	1.14303	-2.19836	-0.00219
H	-0.38465	3.60122	-0.00027
H	2.10574	2.95672	-0.00292
H	3.13630	-0.14679	-0.87643
H	3.67795	1.32752	-0.00517
H	3.13870	-0.14653	0.86816
H	-2.54977	2.93933	0.00246
H	-2.79307	1.38856	-0.87038
H	-2.79131	1.38773	0.87436

bisMIC_diazo_Cl2 (*NImag* = 0)

N	-3.06123	-1.85571	0.21158
C	-2.58652	-0.58609	0.54144
C	-1.38469	-0.39819	-0.10984
N	-1.19126	-1.59446	-0.80440
C	-2.19747	-2.45705	-0.60629
Pd	-0.00753	1.04071	-0.20613
Cl	1.72184	2.65387	-0.38665
C	-3.32880	0.30743	1.46347
C	-0.01419	-1.81948	-1.61635
N	1.16683	-1.59758	-0.80924
C	1.36625	-0.40180	-0.11553
C	2.57022	-0.59291	0.53086
N	3.04026	-1.86376	0.19908

C	2.17154	-2.46281	-0.61526
C	3.31860	0.29865	1.44985
C	4.29374	-2.44110	0.65286
C	-4.31438	-2.42975	0.67049
Cl	-1.73343	2.65835	-0.37957
H	-0.01632	-2.83747	-2.00095
H	-0.01490	-1.10266	-2.43623
H	-4.35020	0.48716	1.11887
H	-3.37758	-0.10568	2.47588
H	-2.81894	1.26813	1.48167
H	4.33905	0.47570	1.10110
H	2.81133	1.26067	1.47015
H	3.37043	-0.11462	2.46205
H	4.38691	-3.45158	0.26225
H	5.12932	-1.83896	0.29926
H	4.31549	-2.47134	1.74097
H	-5.14978	-1.82540	0.32027
H	-4.41179	-3.43999	0.28030
H	-4.33176	-2.45988	1.75868
H	-2.31083	-3.43409	-1.03944
H	2.28045	-3.44014	-1.04888

bisNHC_Cl2 (*NImag* = 0)

C	-2.14550	2.85001	-0.10904
N	-1.28767	1.84950	-0.44471
C	-1.66889	0.64326	0.11921
C	-2.79372	0.90377	0.82041
N	-3.06654	2.25502	0.67395
C	-0.10615	2.10913	-1.24306
N	0.66634	3.17723	-0.64035
C	1.99377	3.13198	-0.24775
C	2.25249	4.33253	0.31464
N	1.08276	5.07434	0.25827
C	0.09606	4.37312	-0.33338
Pd	-1.82458	4.71161	-0.74442
Cl	-4.09365	4.92117	-1.34332
C	0.93935	6.40413	0.84125
C	-4.16368	2.93685	1.35228
Cl	-1.28499	6.82950	-1.62459
H	0.07227	6.87908	0.39026
H	0.82210	6.32098	1.92215
H	1.82860	6.98854	0.61215
H	-4.35260	3.87251	0.83298
H	-5.05356	2.31208	1.30171
H	-3.89767	3.11462	2.39470
H	-0.40642	2.40303	-2.24787
H	0.50108	1.20905	-1.28690
H	2.62502	2.27901	-0.41791
H	-1.13112	-0.27319	-0.04176
H	3.15673	4.72604	0.74178
H	-3.42388	0.25484	1.40071

bisNHCS_Cl2 (*NImag* = 0)

C	-2.09603	2.84812	-0.19736
N	-1.20737	1.78171	-0.67061
C	-1.34778	0.64698	0.04996
N	-2.38892	0.78346	0.85855
C	-3.11007	2.04060	0.62029
C	-0.00010	2.00267	-1.44343
N	1.20731	1.78175	-0.67081
C	2.09581	2.84825	-0.19747
C	3.11006	2.04076	0.61994
N	2.38899	0.78360	0.85832
C	1.34781	0.64705	0.04979
C	2.94283	-0.22261	1.73973
C	-2.94260	-0.22276	1.74004
H	-2.30398	-1.09912	1.72007
H	-3.01978	0.18088	2.75247
H	-3.93472	-0.52028	1.39424
H	2.30423	-1.09899	1.71985
H	3.93491	-0.52010	1.39380
H	3.02013	0.18103	2.75215
H	-0.00016	1.32086	-2.29346
H	-0.00015	3.02620	-1.81302
Cl	-1.72796	-2.36022	-0.52003
Cl	1.72809	-2.36011	-0.52025
Pd	0.00004	-0.79747	-0.15888
H	-4.02635	1.83950	0.05699
H	-1.54653	3.56306	0.42509
H	1.54623	3.56295	0.42518
H	4.02621	1.83970	0.05642
H	-2.55366	3.38611	-1.02626
H	-3.37983	2.51731	1.56220
H	2.55326	3.38648	-1.02631
H	3.38003	2.51746	1.56180

CAAC_benzimi_Cl2 (*NImag* = 0)

N	-1.86251	-0.20373	-0.68287
C	-0.95817	-0.23369	0.24593
C	-2.49367	1.51518	0.78630
C	-3.00714	0.73761	-0.44345
H	-3.29399	1.73792	1.49079
C	-1.38938	0.63487	1.40492
C	-0.19087	1.43256	1.95536
H	-0.54396	2.33182	2.45688
H	0.34225	0.82484	2.68543
C	-1.90344	-0.27530	2.53408
H	-1.14157	-1.00339	2.80908
H	-2.15624	0.32921	3.40814
H	-2.79007	-0.82734	2.23197
C	1.38453	0.89749	0.14101
N	0.75658	1.82333	0.92068
Cl	3.07060	-1.78417	0.25000
Cl	-0.15906	-3.16003	0.32118
Pd	0.82991	-1.01489	0.20591
C	-1.78211	-0.98490	-1.91382
H	-2.77795	-1.32183	-2.19491
H	-1.36996	-0.37491	-2.71808
H	-1.14768	-1.84702	-1.72757
C	3.09797	1.00311	-1.66978
H	2.74841	1.30483	-2.65850
H	4.11964	1.35157	-1.52282
H	3.07948	-0.07717	-1.55786
N	2.24157	1.58210	-0.64608
C	2.17484	2.94454	-0.37163
C	-3.18380	1.65227	-1.65327
H	-3.52751	1.10397	-2.53051
H	-3.93074	2.41306	-1.42278
H	-2.24845	2.15628	-1.90036
C	-4.29193	-0.04709	-0.17666
H	-5.10388	0.64571	0.04736
H	-4.58592	-0.63045	-1.04944

H	-4.17862	-0.73009	0.66251
H	-2.06583	2.46320	0.45433
C	1.22219	3.10214	0.64410
C	0.91756	4.35350	1.16037
C	2.84560	4.03240	-0.91114
C	1.59041	5.44367	0.62043
H	0.19121	4.48368	1.95054
C	2.53655	5.28663	-0.39775
H	3.57943	3.91130	-1.69535
H	1.37900	6.43469	0.99855
H	3.04068	6.15876	-0.79124

CAAC_MIC_Cl2 (*NImag* = 0)

N	-3.22933	-1.06253	0.52355
C	-1.94789	-1.60166	0.64131
C	-1.07835	-0.76670	-0.02340
N	-1.88774	0.26443	-0.50858
C	-3.16727	0.06875	-0.18158
Pd	0.91985	-0.67798	-0.22973
Cl	3.26359	-0.27865	-0.34516
C	-1.69179	-2.85557	1.39147
C	-1.37820	1.33031	-1.36239
C	-0.19454	2.09447	-0.73795
C	0.63705	1.19227	0.15057
N	0.87932	1.86739	1.25468
C	0.17178	3.10547	1.29009
C	-0.52509	3.25780	0.16803
C	1.68434	1.38334	2.37030
C	0.72759	2.58216	-1.88647
H	-4.00206	0.69049	-0.44988
Cl	1.03687	-2.99281	-0.70339
H	-1.11279	4.11868	-0.10904
H	0.27666	3.75050	2.14788
H	-2.19333	2.01426	-1.59509
H	-1.04198	0.86268	-2.28659
H	1.10742	1.72488	-2.43969
H	0.16824	3.23647	-2.55762
H	1.57611	3.13306	-1.48569
H	2.25236	2.21438	2.78644
H	1.03724	0.96013	3.14014
H	2.36467	0.62680	1.98672
H	-2.29712	-3.68358	1.01429
H	-0.64670	-3.12447	1.25250
H	-1.90368	-2.74051	2.45903
C	-4.44281	-1.64197	1.07292
H	-4.35294	-1.74127	2.15365
H	-5.28600	-0.99619	0.84018
H	-4.61360	-2.62687	0.64075

CAAC_NHC_Cl2 (*NImag* = 0)

N	2.16371	-0.13406	1.24142
C	1.32396	-0.03055	0.25483
C	2.96410	1.72484	0.03309
C	3.37487	0.68843	1.09298
H	4.20730	0.05812	0.77018
C	1.93135	0.87830	-0.79385
C	0.86174	1.67538	-1.56554
H	1.29275	2.59160	-1.96681
H	0.51909	1.07407	-2.40644
C	2.61948	-0.05737	-1.81156
H	1.90149	-0.79048	-2.17585
H	3.00475	0.51538	-2.65635
H	3.44448	-0.60706	-1.36206
C	-1.02928	1.12090	-0.07675
N	-0.30923	2.03650	-0.77393
Cl	-2.65124	-1.56423	-0.41610
Cl	0.52539	-2.94123	0.02678
Pd	-0.45363	-0.78737	0.01432
C	2.00860	-0.98209	2.41286
H	2.89095	-1.61419	2.51700
H	1.90920	-0.35601	3.30205
H	1.13762	-1.61388	2.27523
C	-3.05778	1.27621	1.36475
H	-2.80682	1.52195	2.39732
H	-4.02555	1.70562	1.11175
H	-3.09588	0.19947	1.21983
N	-2.04750	1.82069	0.46522
C	-1.97851	3.15327	0.09453
C	-0.88757	3.28962	-0.69209
H	-2.70906	3.87376	0.41419
H	-0.48668	4.14840	-1.19847
H	3.63818	1.13528	2.05257
C	2.28372	2.90313	0.74374
H	2.99844	3.39668	1.40431
H	1.93154	3.64993	0.03281
H	1.43071	2.58414	1.34257
C	4.15309	2.24159	-0.77004
H	3.82256	2.91088	-1.56723
H	4.82565	2.81268	-0.12682
H	4.72886	1.43622	-1.22311

CAAC_PPh2_Cl2 (*NImag* = 0)

N	-2.63581	0.13984	-0.01707
C	-1.56831	-0.37677	0.50375
C	-2.18687	1.52289	1.84077
H	-2.63545	1.70913	2.81569
C	-1.27145	0.27972	1.84373
C	0.24172	0.61605	1.92950
H	0.40078	1.65557	2.21363
H	0.72838	-0.01027	2.67714
C	-1.63850	-0.72716	2.94733
H	-1.05312	-1.63935	2.83621
H	-1.43509	-0.28873	3.92605
H	-2.69120	-1.00357	2.90208
Cl	1.51471	-2.81769	-1.29066
Cl	-1.84331	-3.28564	-0.57210
Pd	-0.22477	-1.58567	-0.28799
C	-3.19621	-0.24875	-1.30781
H	-2.82142	-1.23565	-1.56309
H	-4.28152	-0.28265	-1.23688
H	-2.90935	0.48238	-2.06389
P	1.02866	0.19384	0.31568
C	2.81656	0.25177	0.57876
C	3.64886	-0.33375	-0.37835
C	3.37701	0.90036	1.68183
C	5.02692	-0.25797	-0.23130
H	3.21093	-0.88110	-1.20202
C	4.75673	0.96996	1.82194
H	2.74688	1.35005	2.43741
C	5.58257	0.39360	0.86381
H	5.66717	-0.72233	-0.96973
H	5.18520	1.47053	2.68060

H	6.65788	0.44481	0.97624
C	0.66399	1.63581	-0.74598
C	0.95927	2.93768	-0.33692
C	0.05450	1.43074	-1.98354
C	0.62415	4.01796	-1.14065
H	1.45995	3.11026	0.60753
C	-0.27331	2.51308	-2.79187
H	-0.15836	0.41961	-2.30651
C	0.00303	3.80694	-2.36851
H	0.85288	5.02427	-0.81431
H	-0.74223	2.34299	-3.75238
H	-0.25521	4.65025	-2.99576
C	-3.25923	1.26471	0.76317
C	-3.44409	2.47936	-0.14285
H	-4.20419	2.30734	-0.90502
H	-3.76570	3.32851	0.46150
H	-2.50736	2.74409	-0.63438
C	-4.59703	0.80981	1.34589
H	-5.02708	1.61537	1.94215
H	-5.31103	0.56182	0.56040
H	-4.47644	-0.06463	1.98348
H	-1.61061	2.40626	1.56644

dmpp_Cl2 (*NImag* = 0)

C	-2.18300	0.68765	0.68143
P	-3.78788	0.78394	-0.16722
C	-3.58158	2.07594	-1.43276
C	-4.85544	1.58848	1.09422
C	-6.36070	1.49900	0.83005
C	-6.92965	0.10747	1.11921
P	-6.53653	-1.17746	-0.13481
C	-6.95230	-2.71711	0.73799
Pd	-4.49840	-1.14247	-1.08227
Cl	-5.23535	-3.12469	-2.15213
C	-7.84606	-0.96709	-1.38133
H	-4.54102	2.63288	1.16164
H	-6.86944	2.22235	1.47063
H	-6.58665	1.79809	-0.19782
H	-6.55133	-0.24816	2.08099
H	-8.01826	0.15024	1.20447
H	-1.44365	0.33138	-0.03208
H	-1.89851	1.66482	1.07626
H	-2.25144	-0.02962	1.49884
H	-7.70293	-1.72840	-2.14560
H	-8.83317	-1.06385	-0.92582
H	-7.75465	0.01430	-1.84616
H	-7.96227	-2.66450	1.14899
H	-6.86742	-3.53993	0.03200
H	-6.23825	-2.87627	1.54551
H	-4.54715	2.30409	-1.88351
H	-3.16068	2.98280	-0.99493
H	-2.92174	1.68365	-2.20404
H	-4.62676	1.12703	2.05828
Cl	-2.40062	-1.10295	-2.18444

ethylenediamine_Cl2 (*NImag* = 0)

C	1.45652	0.45434	0.47225
C	0.43744	1.22984	-0.33134
H	1.27347	0.58362	1.53766
H	0.63873	1.12524	-1.39615
N	1.37215	-0.99724	0.18274
N	-0.93324	0.71989	-0.08889
H	0.49549	2.29764	-0.09114
H	2.46681	0.82634	0.26686
C	2.01848	-1.75812	1.27274
H	1.92136	-2.81867	1.06343
H	3.07478	-1.47650	1.34992
H	1.51124	-1.54323	2.21044
C	2.02485	-1.33927	-1.09789
H	3.09764	-1.11955	-1.04639
H	1.86781	-2.39765	-1.28705
H	1.57914	-0.77278	-1.91163
C	-1.47976	1.20532	1.19506
H	-1.58086	2.29674	1.17493
H	-2.45057	0.74186	1.34780
H	-0.82685	0.91854	2.01555
C	-1.82386	1.15095	-1.18697
H	-2.81506	0.74467	-1.01244
H	-1.85912	2.24537	-1.23186
H	-1.44976	0.75617	-2.12876
Pd	-0.71960	-1.40579	0.01675
Cl	-2.99575	-1.68884	-0.06542
Cl	-0.33093	-3.66764	0.03789

propylenediamine_Cl2 (*NImag* = 0)

C	-2.54976	0.72715	0.54672
N	-3.89343	0.87286	-0.06332
C	-3.79650	1.80510	-1.21162
C	-4.77914	1.45536	0.96892
C	-6.26661	1.36072	0.68372
C	-6.83159	-0.01340	0.99260
N	-6.58162	-1.05086	-0.03228
C	-6.86895	-2.36287	0.59618
Pd	-4.55591	-1.05578	-0.92155
Cl	-5.11708	-3.01651	-1.99575
C	-7.51208	-0.85367	-1.16922
H	-4.48656	2.50277	1.10770
H	-6.77269	2.07844	1.33337
H	-6.49675	1.66588	-0.33764
H	-6.39736	-0.36791	1.92849
H	-7.91515	0.04921	1.14715
H	-1.85265	0.37040	-0.20247
H	-2.21980	1.69349	0.94525
H	-2.60179	0.00043	1.35469
H	-7.33569	-1.63206	-1.90469
H	-8.54779	-0.89718	-0.81237
H	-7.33415	0.11387	-1.63258
H	-7.88428	-2.35857	1.00918
H	-6.76706	-3.14568	-0.14630
H	-6.15287	-2.53970	1.39591
H	-4.77661	1.94514	-1.66137
H	-3.41478	2.77466	-0.87075
H	-3.12847	1.37865	-1.95311
H	-4.56942	0.94019	1.90739
Cl	-2.53629	-1.16853	-2.02665

BiPy_pdIV_oxo_bent_t (*NImag* = 0)

N	-0.45039	1.23272	-0.00003
C	-1.67453	0.68278	-0.00009
C	-2.80908	1.48778	-0.00013
C	-2.66505	2.86552	-0.00013
C	-1.39074	3.41309	-0.00008

C	-0.30368	2.55504	-0.00003
C	-1.71578	-0.79185	-0.00009
C	-2.88813	-1.54300	-0.00015
C	-2.79705	-2.92655	-0.00015
C	-1.54680	-3.52853	-0.00009
C	-0.42286	-2.71444	-0.00004
N	-0.52328	-1.39274	-0.00004
Pd	1.29231	0.00264	0.00005
Cl	1.27334	0.12511	-2.34368
O	2.99182	-0.64498	0.00014
Cl	1.27310	0.12511	2.34379
H	-3.85487	-1.06251	-0.00019
H	-3.69600	-3.52828	-0.00019
H	-1.43765	-4.60361	-0.00009
H	0.58011	-3.11980	0.00001
H	-3.79324	1.04475	-0.00017
H	-3.53880	3.50312	-0.00016
H	0.71258	2.92188	0.00001
H	-1.23293	4.48176	-0.00007

BiPy_pdIV_oxo_s (*NImag* = 0)

C	-0.22915	1.48775	-0.00000
C	-0.58723	2.82993	-0.00005
C	-1.93208	3.16621	-0.00003
C	-2.88649	2.15890	0.00004
C	-2.46134	0.83963	0.00008
H	0.17119	3.59843	-0.00010
H	-2.23149	4.20547	-0.00007
H	-3.94338	2.38194	0.00006
H	-3.15321	0.00965	0.00014
C	1.14214	0.97437	-0.00001
C	2.29360	1.75142	-0.00009
C	3.52865	1.12186	-0.00009
H	2.22631	2.82904	-0.00016
C	2.39990	-0.98030	0.00007
C	3.58689	-0.26458	-0.00000
H	4.43694	1.70897	-0.00015
H	2.37661	-2.06058	0.00014
H	4.53041	-0.79044	0.00001
N	-1.16984	0.53054	0.00006
N	1.22293	-0.36526	0.00007
Pd	-0.56558	-1.49898	0.00014
O	-1.18375	-3.14952	0.00021
Cl	-0.54769	-1.45122	2.36184
Cl	-0.54769	-1.45142	-2.36157

bisbenzimi_pdIV_oxo_s (*NImag* = 0)

N	2.52448	-0.84078	0.38127
C	1.29547	-0.62191	-0.11620
C	2.45614	1.31194	-0.23296
C	0.09484	1.25034	-1.15281
H	0.17339	2.33281	-1.11829
H	0.06403	0.89060	-2.18263
C	-1.35343	-0.43004	-0.10393
N	-1.11734	0.84806	-0.47414
Pd	-0.14153	-2.06493	-0.33951
C	3.02679	-2.10837	0.89493
H	3.99549	-2.31179	0.43989
H	3.12024	-2.05824	1.97834
H	2.32613	-2.89657	0.64163
C	-3.27159	-1.65214	0.92403
H	-3.34781	-1.58943	2.00813
H	-4.26328	-1.71393	0.47730
H	-2.69375	-2.53285	0.66559
N	-2.59646	-0.46982	0.40514
C	-3.17375	0.79765	0.36338
C	-2.22428	1.65099	-0.21110
C	-2.48561	2.99777	-0.41302
C	-4.41575	1.26467	0.76772
C	-3.73043	3.46539	-0.00925
H	-1.76325	3.66162	-0.86721
C	-4.67745	2.61484	0.57115
H	-5.14773	0.60931	1.21767
H	-3.97228	4.51008	-0.14899
H	-5.63483	3.01716	0.87273
C	3.27802	0.33028	0.33325
C	4.57808	0.61315	0.72566
C	5.02981	1.91162	0.52568
H	5.21212	-0.14114	1.16926
C	2.90697	2.60711	-0.43828
C	4.20991	2.89016	-0.04648
H	6.03795	2.17155	0.81803
H	2.28362	3.36844	-0.88618
H	4.59850	3.88917	-0.18923
N	1.24260	0.67714	-0.48504
O	-0.27023	-3.82380	-0.61930
Cl	-0.12515	-1.68250	-2.70399
Cl	-0.15681	-2.42996	2.03088

bisbenzimi_pdIV_oxo_t (*NImag* = 0)

N	2.56948	-0.83061	0.42535
C	1.35813	-0.72954	-0.14760
C	2.37183	1.29214	-0.25474
C	0.08847	1.01973	-1.32388
H	0.15762	2.09596	-1.44074
H	0.10682	0.52671	-2.29596
C	-1.47722	-0.56820	-0.31347
N	-1.15350	0.70064	-0.65165
Pd	-0.07651	-2.14228	-0.42816
C	3.14858	-2.04553	0.97707
H	3.97134	-2.37886	0.34424
H	3.51269	-1.84712	1.98412
H	2.37723	-2.80534	1.03285
C	-3.38244	-1.68123	0.82453
H	-3.29355	-1.69015	1.90970
H	-4.42994	-1.62112	0.53175
H	-2.93065	-2.57797	0.40862
N	-2.67555	-0.52692	0.28166
C	-3.13653	0.78762	0.34580
C	-2.15536	1.58549	-0.25693
C	-2.30441	2.95994	-0.36691
C	-4.29848	1.33944	0.86545
C	-3.46785	3.51219	0.15468
H	-1.55517	3.58292	-0.83549
C	-4.44681	2.71646	0.76016
H	-5.05320	0.72617	1.33668
H	-3.61944	4.58105	0.09085
H	-5.33809	3.18438	1.15531
C	3.23633	0.39152	0.37781
C	4.49112	0.78138	0.82174
C	4.85313	2.10607	0.61030
H	5.16099	0.08624	1.30750

C	2.73269	2.61397	-0.46870
C	3.98946	3.00571	-0.02330
H	5.82432	2.44846	0.94038
H	2.07563	3.31620	-0.96269
H	4.30704	4.02858	-0.17289
N	1.22650	0.55920	-0.55187
O	-1.31750	-3.55869	-0.71757
Cl	0.56267	-2.04250	-2.74761
Cl	-0.11802	-2.58842	1.94669

bisCAAC_pdIV_diastereo_oxo_s (*NImag* = 0)

C	-3.42354	1.08326	-0.23550
N	-2.51232	-0.11454	-0.31783
C	-1.37198	0.02311	0.28312
C	-1.36333	1.28999	1.10421
C	-2.50516	2.10828	0.45771
C	-2.94557	-1.29184	-1.07307
Pd	0.06232	-1.39708	0.06234
O	-0.09875	-3.18641	0.00954
C	0.00210	2.00536	1.12479
C	0.94027	1.72350	-0.05900
C	1.27219	0.25648	-0.08014
N	2.54622	0.07691	-0.18334
C	3.37676	1.32746	-0.20389
C	2.31939	2.39343	0.14913
C	-1.72615	0.91579	2.55932
C	3.17460	-1.24418	-0.27177
C	3.97695	1.51338	-1.59723
C	4.46997	1.24575	0.85796
C	0.32351	2.12732	-1.40843
C	-4.68398	0.75399	0.56443
C	-3.79781	1.52366	-1.64986
H	-0.16529	3.08351	1.19869
H	0.52906	1.70246	2.02991
H	-0.98594	0.23475	2.97199
H	-1.75211	1.83020	3.15599
H	-2.70176	0.43836	2.62533
H	-3.99832	-1.46859	-0.86239
H	-2.79613	-1.12752	-2.13709
H	-2.35655	-2.15280	-0.77179
H	2.43471	2.68644	1.19284
H	2.43578	3.28839	-0.46002
H	4.65869	0.69988	-1.84715
H	3.20118	1.55043	-2.36048
H	4.54370	2.44468	-1.63057
H	5.22866	0.50375	0.60946
H	4.96662	2.21406	0.93492
H	4.04549	0.99682	1.83090
H	2.65040	-1.83869	-1.01653
H	4.21418	-1.12841	-0.56246
H	3.10667	-1.73945	0.69553
H	-3.04437	2.69786	1.19799
H	-2.10398	2.79551	-0.28474
H	-5.28886	1.65596	0.66293
H	-5.29289	0.00441	0.05972
H	-4.44907	0.39006	1.56201
H	-4.33151	2.47361	-1.59664
H	-2.90844	1.66353	-2.26441
H	-4.44907	0.80111	-2.14136
H	0.04914	3.18392	-1.38090
H	1.03099	1.97391	-2.22076
H	-0.55025	1.53018	-1.65277
Cl	0.05026	-1.28656	-2.36116
Cl	0.74597	-1.53088	2.35501

bisCAAC_pdIV_diastereo_oxo_t (*NImag* = 0)

C	-3.44914	1.15321	-0.23923
N	-2.57569	-0.06787	-0.39221
C	-1.46713	-0.03229	0.26767
C	-1.40398	1.19021	1.14553
C	-2.51845	2.08841	0.55613
C	-2.99660	-1.16065	-1.27197
Pd	0.07257	-1.36934	0.08709
O	-0.99868	-2.95604	0.28200
C	-0.01236	1.86247	1.18435
C	0.91925	1.64949	-0.02208
C	1.31709	0.19931	-0.10948
N	2.59113	0.08611	-0.27430
C	3.36779	1.36824	-0.23078
C	2.27204	2.36871	0.19262
C	-1.75386	0.73632	2.58001
C	3.27282	-1.19797	-0.42989
C	3.95140	1.66466	-1.61178
C	4.47033	1.26027	0.81967
C	0.27714	2.06007	-1.35888
C	-4.72807	0.79100	0.51602
C	-3.78566	1.71482	-1.61826
H	-0.15667	2.93680	1.32881
H	0.51913	1.49153	2.06069
H	-1.03068	0.00282	2.93213
H	-1.73131	1.60600	3.24006
H	-2.74729	0.29370	2.63147
H	-4.08024	-1.24346	-1.23578
H	-2.66969	-0.95844	-2.28971
H	-2.53982	-2.07988	-0.91781
H	2.39479	2.61509	1.24737
H	2.33659	3.29659	-0.37381
H	4.66687	0.89939	-1.91409
H	3.17129	1.71660	-2.36975
H	4.47738	2.61979	-1.58845
H	5.24789	0.55611	0.52289
H	4.93831	2.23680	0.95146
H	4.05747	0.93983	1.77668
H	2.67778	-1.83083	-1.08387
H	4.24947	-1.03614	-0.87726
H	3.37838	-1.66917	0.54617
H	-3.05774	2.62383	1.33638
H	-2.08766	2.83035	-0.11400
H	-5.32407	1.69212	0.66477
H	-5.33661	0.08191	-0.04476
H	-4.51054	0.35896	1.49074
H	-4.30566	2.66623	-1.49875
H	-2.88167	1.89032	-2.20140
H	-4.43729	1.04646	-2.18100
H	-0.04584	3.10152	-1.30786
H	0.98764	1.95975	-2.17710
H	-0.57090	1.43030	-1.61422
Cl	0.24156	-1.53595	-2.37065
Cl	1.08539	-1.51478	2.32627

bisCAAC_pdIV_oxo_s (*NImag* = 0)

C	1.37242	-0.01568	-0.40450

N	1.23479	1.24234	-0.68400
C	2.49715	1.99628	-1.00606
C	3.48831	0.83184	-1.14840
C	0.02658	1.99760	-0.42058
N	-1.17855	1.24914	-0.71542
C	-2.42790	2.01060	-1.06917
C	-3.42110	0.85188	-1.24010
C	-1.33038	-0.00838	-0.44087
C	-3.04961	-1.65163	-1.40065
C	3.10815	-1.67036	-1.31384
H	-2.64029	-2.53575	-0.92061
H	-4.12967	-1.77259	-1.51243
H	-2.59962	-1.57820	-2.38995
H	2.67801	-2.55060	-0.84496
H	2.68859	-1.59535	-2.31631
H	4.19018	-1.79931	-1.39279
H	0.03694	2.89582	-1.02763
H	0.01333	2.24809	0.64382
H	-3.56815	0.65294	-2.30171
H	3.66294	0.63002	-2.20531
H	-4.39180	1.09390	-0.81062
H	4.44838	1.06958	-0.69339
C	2.83254	-0.40986	-0.49384
C	-2.78988	-0.39438	-0.57041
C	-3.32141	-0.61887	0.86245
H	-2.83322	-1.47692	1.32139
H	-3.14721	0.23960	1.50900
H	-4.39436	-0.81004	0.80569
C	3.32344	-0.63386	0.95361
H	3.13097	0.22468	1.59488
H	2.82221	-1.49163	1.39878
H	4.39754	-0.82497	0.92690
C	2.83148	2.93293	0.15604
H	3.79346	3.41033	-0.03213
H	2.08709	3.72354	0.26484
H	2.89127	2.38953	1.09754
C	2.33738	2.77363	-2.30916
H	3.30700	3.17717	-2.60276
H	1.98376	2.12229	-3.10896
H	1.65303	3.61780	-2.21161
C	-2.78743	2.94710	0.08558
H	-2.87435	2.40245	1.02426
H	-2.04183	3.73343	0.21491
H	-3.74160	3.43001	-0.12670
C	-2.23048	2.78959	-2.36616
H	-1.85966	2.13801	-3.15795
H	-3.19036	3.19820	-2.68383
H	-1.54513	3.63054	-2.24947
O	-0.00726	-2.60370	1.62657
Pd	0.00654	-1.24269	0.46877
Cl	-0.01594	0.33160	2.33535
Cl	0.02532	-2.81659	-1.31762

bisCAAC_N_pdIV_oxo_s (*NImag* = 0)

C	-1.30903	0.00616	-0.14458
N	-1.19794	1.28490	0.03205
C	-2.45646	2.09171	-0.13594
C	-3.48911	0.96395	-0.31279
C	0.00016	1.93327	0.52412
N	1.19825	1.28471	0.03228
C	2.45682	2.09139	-0.13565
C	3.48942	0.96350	-0.31195
C	1.30919	0.00594	-0.14433
C	3.28140	-1.55945	0.03292
C	-3.28145	-1.55902	0.03228
H	2.71543	-2.45197	-0.22828
H	4.31582	-1.69238	-0.29055
H	3.26601	-1.45324	1.11790
H	-2.71551	-2.45161	-0.22871
H	-3.26634	-1.45275	1.11726
H	-4.31580	-1.69184	-0.29147
H	0.00005	1.87395	1.61562
H	0.00027	2.96657	0.19094
H	4.02776	0.81891	0.62463
H	-4.02798	0.81940	0.62349
H	4.21894	1.20657	-1.08273
H	-4.21820	1.20715	-1.08394
C	-2.69548	-0.32309	-0.65411
C	2.69574	-0.32342	-0.65358
C	2.62195	-0.55705	-2.17520
H	2.00444	-1.42390	-2.39657
H	2.20337	0.30322	-2.69877
H	3.63316	-0.72660	-2.54972
C	-2.62129	-0.55677	-2.17571
H	-2.20264	0.30354	-2.69918
H	-2.00369	-1.42359	-2.39692
H	-3.63240	-0.72638	-2.55048
C	-2.33113	3.00177	-1.35648
H	-3.25621	3.56458	-1.48470
H	-1.52267	3.72593	-1.23913
H	-2.15185	2.42818	-2.26411
C	-2.71321	2.90402	1.13159
H	-3.70747	3.34822	1.07395
H	-2.67098	2.26421	2.01333
H	-1.99655	3.71807	1.25359
C	2.33184	3.00109	-1.35649
H	2.15327	2.42723	-2.26408
H	1.52306	3.72500	-1.23984
H	3.25681	3.56419	-1.48435
C	2.71339	2.90408	1.13168
H	2.67083	2.26458	2.01363
H	3.70773	3.34808	1.07414
H	1.99684	3.71830	1.25323
O	-0.00077	-2.96124	1.38020
Pd	-0.00016	-1.41020	0.50529
Cl	0.00017	-2.69895	-1.50821
Cl	-0.00078	-0.32801	2.68420

bisCAAC_N_pdIV_oxo_t (*NImag* = 0)

C	-1.43541	0.04385	0.07133
N	-1.20036	1.29556	0.31094
C	-2.31164	2.25348	-0.02512
C	-3.44592	1.26012	-0.33689
C	0.07358	1.76365	0.83899
N	1.18900	1.20427	0.09015
C	2.42352	2.02266	-0.15960
C	3.40077	0.92563	-0.62569
C	1.26646	-0.05969	-0.19866
C	3.22600	-1.61719	-0.44186
C	-3.60031	-1.26473	0.02000
H	2.62202	-2.48629	-0.69661
H	4.20355	-1.71755	-0.91817

H	3.36163	-1.59362	0.63949
H	-3.12010	-2.22343	-0.16010
H	-3.70987	-1.14970	1.09805
H	-4.59273	-1.26797	-0.43609
H	0.17216	1.44090	1.87822
H	0.10570	2.84435	0.76987
H	4.09992	0.70062	0.17906
H	-4.11243	1.19262	0.52304
H	3.97934	1.24716	-1.49047
H	-4.03768	1.58393	-1.19163
C	-2.78330	-0.12165	-0.58196
C	2.55289	-0.33422	-0.93481
C	2.22671	-0.45025	-2.43718
H	1.55306	-1.28631	-2.61424
H	1.76196	0.45971	-2.82093
H	3.15608	-0.61503	-2.98555
C	-2.54322	-0.39318	-2.08116
H	-1.96827	0.40337	-2.55495
H	-2.00167	-1.32766	-2.21673
H	-3.51092	-0.46010	-2.58133
C	-1.90541	3.10943	-1.22397
H	-2.73190	3.76672	-1.49520
H	-1.04682	3.74156	-0.99359
H	-1.65869	2.49457	-2.08752
C	-2.62774	3.12691	1.18536
H	-3.52148	3.71647	0.97803
H	-2.81863	2.51441	2.06662
H	-1.82079	3.82557	1.41290
C	2.14067	3.07954	-1.22454
H	1.80470	2.62484	-2.15529
H	1.38244	3.79114	-0.89301
H	3.05064	3.64493	-1.42722
C	2.88002	2.66481	1.14914
H	2.94921	1.91544	1.93792
H	3.86479	3.10988	1.00294
H	2.20721	3.45950	1.47734
O	-1.16408	-2.68985	1.36807
Pd	-0.08198	-1.38566	0.50876
Cl	-0.06621	-2.90689	-1.44749
Cl	1.02591	-0.77810	2.66158

bisCAAC_pdIV_oxo_s (*NImag* = 0)

N	1.87893	-0.38399	-0.77072
C	1.16617	0.37088	0.04965
C	1.32425	-0.15812	1.46037
C	-0.00008	-0.07122	2.25301
H	-0.00016	-0.82797	3.04261
H	0.00005	0.89375	2.75811
C	2.35670	0.71970	2.19642
H	2.01724	1.75421	2.21733
H	2.47786	0.36406	3.22243
H	3.33109	0.69817	1.71215
C	-1.16609	0.37090	0.04951
Pd	0.00012	1.87417	-0.40173
C	2.00613	-0.11639	-2.19191
H	3.04022	-0.25083	-2.51037
H	1.37082	-0.78497	-2.77674
H	1.69698	0.91046	-2.37253
N	-1.87886	-0.38400	-0.77080
C	-1.87133	-1.58686	1.23953
H	-2.57598	-1.88518	2.01560
H	-1.05505	-2.30875	1.24646
C	-2.52628	-1.57988	-0.15756
C	-2.17483	-2.83969	-0.94896
H	-2.62871	-2.83700	-1.94058
H	-1.09520	-2.93922	-1.06258
H	-2.54296	-3.71834	-0.41725
C	-4.04816	-1.41189	-0.11610
H	-4.46386	-1.34585	-1.12238
H	-4.50181	-2.27576	0.37184
H	-4.34047	-0.51749	0.42956
C	-1.32437	-0.15785	1.46033
C	-2.35669	0.72034	2.19617
H	-2.47831	0.36466	3.22210
H	-2.01681	1.75470	2.21725
H	-3.33092	0.69926	1.71156
C	-2.00610	-0.11645	-2.19200
H	-1.69493	0.90972	-2.37291
H	-1.37241	-0.78650	-2.77690
H	-3.04060	-0.24882	-2.51004
C	1.87086	-1.58722	1.23937
C	2.52612	-1.58005	-0.15757
H	2.57522	-1.88594	2.01555
H	1.05436	-2.30885	1.24583
C	4.04801	-1.41225	-0.11584
H	4.50146	-2.27617	0.37220
H	4.46389	-1.34628	-1.12204
H	4.34035	-0.51786	0.42985
C	2.17463	-2.83966	-0.94927
H	2.54259	-3.71848	-0.41772
H	1.09499	-2.93903	-1.06301
H	2.62860	-2.83682	-1.94085
O	0.00034	3.62817	-0.80566

bisCAAC_pdIV_oxo_t (*NImag* = 0)

N	2.08654	-0.42086	-0.54481
C	1.44515	0.35781	0.31863
C	1.12374	-0.48423	1.54266
C	-0.19874	-0.09111	2.22997
H	-0.39949	-0.79858	3.04062
H	-0.01097	0.87244	2.70550
C	2.24323	-0.22576	2.57204
H	2.25800	0.82639	2.85512
H	2.07647	-0.82791	3.46883
H	3.22468	-0.47266	2.17205
C	-1.14874	0.52994	-0.01355
Pd	0.26544	1.89772	-0.32387
C	2.63755	0.08334	-1.79005
H	3.63162	-0.33197	-1.96309
H	1.99900	-0.17500	-2.63868
H	2.70202	1.16582	-1.72066
N	-1.80673	-0.21853	-0.87252
C	-2.29502	-1.23865	1.18141
H	-3.15092	-1.29822	1.85342
H	-1.67616	-2.11186	1.37834
C	-2.73951	-1.23540	-0.29286
C	-2.55165	-2.59792	-0.95574
H	-2.84389	-2.58238	-2.00653
H	-1.51635	-2.93064	-0.88844
H	-3.17711	-3.33490	-0.44993
C	-4.18723	-0.76644	-0.47243
H	-4.45969	-0.71046	-1.52706

H	-4.86648	-1.47280	0.00663
H	-4.34252	0.21562	-0.02885
C	-1.48807	0.06752	1.38646
C	-2.36035	1.14270	2.06216
H	-2.58197	0.84532	3.09001
H	-1.83979	2.09911	2.07519
H	-3.30564	1.28179	1.53764
C	-1.71599	-0.03775	-2.31154
H	-1.19039	0.89531	-2.49939
H	-1.16355	-0.85862	-2.77208
H	-2.71084	0.00548	-2.75633
C	1.19505	-1.93430	1.00559
C	2.09738	-1.88153	-0.24475
H	1.56978	-2.63786	1.74981
H	0.20566	-2.27279	0.70789
C	3.52170	-2.38108	0.01366
H	3.50388	-3.44557	0.25293
H	4.14876	-2.25219	-0.86942
H	3.98882	-1.85245	0.84197
C	1.48717	-2.66249	-1.41030
H	1.34818	-3.70681	-1.12515
H	0.51612	-2.24807	-1.68108
H	2.12900	-2.64092	-2.29178
O	1.17133	3.50855	-0.82830

bisDAC_pdIV_oxo_s (*NImag* = 0)

C	-1.29463	0.17599	-0.00585
N	-1.15881	1.51193	0.18340
C	-2.33661	2.21009	-0.11908
C	-3.30615	1.08724	-0.55002
N	-2.54226	-0.08984	-0.42677
C	0.01932	2.12818	0.75550
N	1.20592	1.54054	0.16982
C	2.35676	2.27087	-0.16333
C	3.34620	1.17413	-0.61517
N	2.61966	-0.02428	-0.47028
C	1.37503	0.20965	-0.02303
O	2.50848	3.45032	-0.10195
Pd	0.06936	-1.28714	0.37475
Cl	0.02343	-0.59739	2.65872
C	3.19609	-1.33346	-0.78856
O	4.46593	1.30014	-0.99829
O	-2.52057	3.38479	-0.04989
O	-4.43804	1.18481	-0.90527
C	-3.08374	-1.41982	-0.71959
O	0.17033	-2.99005	0.87850
Cl	0.07500	-1.92737	-1.92761
H	2.80323	-2.07122	-0.09463
H	2.92648	-1.61730	-1.80224
H	4.27428	-1.24429	-0.68771
H	-2.86194	-2.08618	0.11047
H	-4.15724	-1.30693	-0.84131
H	-2.62654	-1.80817	-1.62553
H	0.02738	1.95624	1.83162
H	0.00601	3.19114	0.52714

bisDAC_pdIV_oxo_t (*NImag* = 0)

C	-2.37048	1.74028	0.12599
N	-1.31530	1.01334	-0.44536
C	-1.46674	-0.32894	-0.27603
N	-2.61105	-0.56477	0.37806
C	-3.28298	0.63112	0.69606
C	-0.18180	1.60146	-1.12365
N	1.05299	1.27714	-0.42975
C	2.07305	2.19649	-0.15630
C	3.19512	1.30855	0.43248
N	2.65260	0.01110	0.40063
C	1.40900	0.00666	-0.10541
C	3.38509	-1.17629	0.83990
C	-3.12559	-1.87784	0.76928
H	-2.57947	-2.63233	0.20975
H	-2.97008	-2.02013	1.83629
H	-4.18845	-1.90843	0.54055
H	3.50423	-1.85536	-0.00203
H	4.35773	-0.84384	1.19164
H	2.83146	-1.66505	1.63729
H	-0.12453	1.21104	-2.13839
H	-0.30004	2.68220	-1.12306
Pd	0.22563	-1.60160	-0.38494
O	2.06359	3.37254	-0.34495
O	4.27059	1.63809	0.82247
O	-2.51568	2.92279	0.13849
O	-4.32613	0.74632	1.25920
O	-0.67748	-3.24651	-0.71176
Cl	0.14445	-1.91564	1.97734
Cl	0.96063	-1.31423	-2.63501

Bisimine_pdIV_oxo_s (*NImag* = 0)

C	0.01256	1.46728	-2.45079
N	0.00733	1.36775	-1.01309
Pd	0.00983	-0.55530	-0.14114
O	0.01608	-2.27444	-0.52231
N	0.00029	0.80952	1.46800
C	-0.00326	0.28370	2.81011
C	-0.00241	2.04935	1.18969
C	0.00177	2.36897	-0.23070
Cl	-2.35135	-0.50893	-0.13807
Cl	2.37061	-0.49518	-0.12274
H	-0.00742	2.82182	1.95487
H	0.00018	3.39480	-0.59121
H	-0.00772	1.08171	3.55511
H	-0.88677	-0.34607	2.92298
H	0.88213	-0.34221	2.92945
H	0.90044	0.95296	-2.82106
H	-0.86844	0.94637	-2.82821
H	0.01000	2.50740	-2.78260

bisimine_pdIV_oxo_t (*NImag* = 0)

C	1.97667	-1.18375	-0.97435
C	2.51716	-0.91452	0.36490
N	1.82577	-0.21795	1.16598
Pd	-0.06734	0.52933	0.55801
Cl	1.05293	2.46690	-0.14107
N	0.84538	-0.69829	-1.25188
Cl	-0.99851	-1.42637	1.45547
O	-1.68507	1.33977	0.45998
C	0.19076	-0.87910	-2.51790
C	2.27347	0.09923	2.50296
H	3.49059	-1.31146	0.64726
H	2.55821	-1.78420	-1.67605
H	3.25582	-0.32766	2.71508
H	2.30465	1.18506	2.59945

H	1.53450	-0.28609	3.20637
H	-0.77429	-1.35536	-2.33650
H	-0.00521	0.10622	-2.94415
H	0.78100	-1.48000	-3.21607

bisMIC_pdIV_diazo_oxo_s (*NImag* = 0)

N	-3.32399	-1.13846	0.22998
C	-2.69702	0.10014	0.35801
C	-1.44827	-0.00965	-0.20443
N	-1.36980	-1.32226	-0.64274
C	-2.49819	-1.99155	-0.37884
Pd	-0.03939	1.46041	-0.48974
O	0.06177	3.20776	-0.84269
C	-3.36560	1.26715	0.98303
C	-0.21538	-1.82951	-1.35627
N	0.97476	-1.45353	-0.62055
C	1.19095	-0.15742	-0.17942
C	2.43333	-0.18711	0.40658
N	2.92047	-1.48806	0.28901
C	2.01627	-2.24429	-0.33616
C	3.21608	0.89864	1.04537
C	4.20845	-1.95264	0.77600
C	-4.66471	-1.45587	0.69215
Cl	-0.03362	1.96033	1.85733
Cl	-0.04204	1.00868	-2.88080
H	-0.27521	-2.91220	-1.44074
H	-0.17841	-1.33387	-2.33044
H	-3.68503	1.05197	2.00551
H	-2.65510	2.08885	1.02707
H	-4.24049	1.58451	0.40878
H	4.13161	1.11577	0.48808
H	2.60103	1.79455	1.07690
H	3.49016	0.65019	2.07346
H	4.32092	-3.00858	0.54292
H	5.00934	-1.39079	0.29814
H	4.27006	-1.81217	1.85400
H	-5.38876	-0.80868	0.19987
H	-4.88992	-2.49292	0.45592
H	-4.73055	-1.30824	1.76895
H	-2.71293	-3.01680	-0.61976
H	2.11966	-3.28736	-0.57409

bisMIC_pdIV_diazo_oxo_t (*NImag* = 0)

N	-3.33434	-1.17490	0.24883
C	-2.77933	0.09936	0.36974
C	-1.52886	0.06850	-0.19943
N	-1.37677	-1.24618	-0.63192
C	-2.46313	-1.97859	-0.36058
Pd	-0.06905	1.46181	-0.48159
O	1.23994	2.82261	-0.75410
C	-3.51960	1.22232	0.99533
C	-0.21922	-1.69901	-1.37970
N	0.99086	-1.37037	-0.64843
C	1.27517	-0.07802	-0.24268
C	2.50333	-0.14901	0.35967
N	2.92050	-1.47943	0.29313
C	1.98454	-2.20650	-0.32157
C	3.30329	0.92242	0.99979
C	4.17912	-1.99443	0.80648
C	-4.64935	-1.57140	0.72225
Cl	-0.27911	2.17576	1.85149
Cl	-0.52948	1.20828	-2.90489
H	-0.27676	-2.77428	-1.53181
H	-0.20469	-1.15113	-2.32542
H	-3.89906	0.95718	1.98501
H	-2.83311	2.05596	1.12203
H	-4.36497	1.54167	0.37893
H	4.32206	0.95837	0.60585
H	2.81545	1.87109	0.78315
H	3.35537	0.79480	2.08407
H	4.22433	-3.06775	0.63900
H	5.01217	-1.51444	0.29523
H	4.25298	-1.79183	1.87364
H	-5.41293	-0.95220	0.25445
H	-4.82455	-2.61320	0.46523
H	-4.70811	-1.45179	1.80309
H	-2.61871	-3.01534	-0.59756
H	2.03890	-3.25972	-0.52998

bisNHC_pdIV_oxo_s (*NImag* = 0)

C	-2.34438	2.76380	-0.23502
N	-1.19088	2.00958	-0.37719
C	-1.33285	0.79762	0.19621
N	-2.57513	0.78612	0.70629
C	-3.21367	1.98883	0.45185
C	-0.00655	2.37689	-1.12602
N	1.17373	1.99711	-0.37705
C	2.33469	2.73962	-0.23401
C	3.19558	1.95592	0.45350
N	2.54485	0.75955	0.70705
C	1.30304	0.78369	0.19634
C	3.12636	-0.35903	1.44516
C	-3.16804	-0.32664	1.44424
H	-4.21839	2.18299	0.77965
H	-2.44265	3.75737	-0.63245
H	2.44328	3.73215	-0.63136
H	4.20202	2.13983	0.78195
H	-2.74475	-1.25785	1.07884
H	-2.93734	-0.23514	2.50287
H	-4.24315	-0.31575	1.27839
H	2.68843	-1.28538	1.08478
H	4.20067	-0.36347	1.27372
H	2.90261	-0.26129	2.50475
H	-0.00937	1.83992	-2.07633
H	-0.00084	3.45285	-1.27865
Pd	-0.02337	-0.78055	0.22500
Cl	-0.02390	-0.78618	-2.17289
O	-0.03373	-2.56570	0.23620
Cl	-0.02296	-0.75165	2.62489

bisNHCS_pdIV_oxo_s (*NImag* = 0)

C	-2.71163	2.03010	-0.14877
N	-1.35510	1.47059	-0.10546
C	-1.37960	0.13838	0.02357
N	-2.61095	-0.26029	0.29283
C	-3.52774	0.87738	0.44528
C	-0.23699	2.17689	-0.68156
N	0.99409	1.72437	-0.08110
C	2.20045	2.56073	-0.09779
C	3.23150	1.60758	0.51552
N	2.58209	0.30071	0.34686
C	1.29984	0.42784	0.05137

C	3.27776	-0.92039	0.70805
C	-3.03716	-1.60260	0.64219
H	-2.27507	-2.31674	0.34525
H	-3.19165	-1.68457	1.71974
H	-3.96841	-1.82431	0.11936
H	2.69198	-1.78013	0.39716
H	4.24545	-0.93706	0.20502
H	3.42395	-0.96977	1.78875
H	-0.20945	2.02629	-1.76774
H	-0.35392	3.23778	-0.45793
Pd	0.12455	-1.20701	-0.29664
H	-4.46080	0.69517	-0.08588
H	-2.77885	2.94834	0.43315
H	2.05808	3.47087	0.48332
H	4.19266	1.62994	0.00406
H	-2.99823	2.24167	-1.18369
H	-3.75085	1.03053	1.50537
H	2.45647	2.83088	-1.12708
H	3.39492	1.80254	1.57961
O	0.31528	-2.93156	-0.73660
Cl	0.16104	-1.76717	2.02803
Cl	0.08006	-0.57253	-2.61153

CAAC_benzimi_pdIV_oxo_s (*NImag* = 0)

N	2.37032	-0.57411	-0.73550
C	1.49285	-0.12003	0.10323
C	1.28221	-2.47014	0.13621
C	2.41544	-2.07921	-0.83475
H	1.53135	-3.35444	0.72152
C	1.05221	-1.23344	1.02450
C	-0.38002	-1.09746	1.54577
H	-0.78096	-2.08749	1.75662
H	-0.37233	-0.51796	2.46799
C	1.98426	-1.22441	2.25503
H	1.88922	-0.27978	2.78646
H	1.70245	-2.04285	2.92138
H	3.02467	-1.35809	1.97192
C	-0.98553	0.76983	0.06449
N	-1.27637	-0.43175	0.61599
Pd	0.85296	1.78195	0.06424
C	3.25020	0.22853	-1.58436
H	4.26264	-0.15909	-1.47651
H	2.93200	0.17293	-2.62101
H	3.21249	1.26382	-1.26226
C	-2.29898	2.50331	-1.16940
H	-2.33713	2.35918	-2.24737
H	-3.22145	2.96055	-0.81286
H	-1.45827	3.14697	-0.93742
N	-2.12475	1.22630	-0.49376
C	-3.15744	0.31254	-0.30864
C	2.07974	-2.47335	-2.27259
H	2.88433	-2.21723	-2.96130
H	1.92997	-3.55314	-2.31895
H	1.16695	-1.97860	-2.60484
C	3.78273	-2.63558	-0.44055
H	3.74362	-3.72488	-0.47119
H	4.55656	-2.31687	-1.13792
H	4.07882	-2.33602	0.56224
H	0.37619	-2.68382	-0.43363
C	-2.61713	-0.75101	0.42279
C	-3.39323	-1.83621	0.80352
C	-4.48826	0.32025	-0.70148
C	-4.72698	-1.82825	0.41337
H	-2.98728	-2.65449	1.38153
C	-5.26413	-0.77027	-0.32814
H	-4.90540	1.13581	-1.27480
H	-5.36371	-2.65725	0.69098
H	-6.30636	-0.80109	-0.61532
Cl	0.65240	2.04308	2.42270
Cl	0.64925	1.77906	-2.35127
O	1.93272	3.21539	-0.00367

CAAC_benzimi_pdIV_oxo_t (*NImag* = 0)

N	2.39232	-0.58642	-0.71866
C	1.62106	-0.08255	0.18560
C	1.23166	-2.42500	0.18062
C	2.34043	-2.09069	-0.83827
H	1.45941	-3.32391	0.75262
C	1.09685	-1.17858	1.07671
C	-0.32232	-0.94297	1.60034
H	-0.75028	-1.89577	1.90621
H	-0.27974	-0.28335	2.46588
C	2.01682	-1.22056	2.31475
H	1.98670	-0.26105	2.83013
H	1.67295	-2.00058	2.99776
H	3.04774	-1.43445	2.04343
C	-1.00209	0.89124	0.09849
N	-1.22944	-0.33189	0.63862
Pd	0.79917	1.83996	0.13468
C	3.24607	0.19076	-1.61609
H	4.23959	-0.25578	-1.61934
H	2.83163	0.19278	-2.62060
H	3.29795	1.20828	-1.24242
C	-2.38722	2.54823	-1.15674
H	-2.44008	2.40586	-2.23439
H	-3.31996	2.96975	-0.78250
H	-1.56575	3.21988	-0.93876
N	-2.15069	1.27774	-0.48999
C	-3.12654	0.29773	-0.34136
C	1.92961	-2.45762	-2.26264
H	2.71632	-2.23230	-2.98249
H	1.72804	-3.52874	-2.31168
H	1.02738	-1.91968	-2.55453
C	3.69077	-2.71722	-0.49418
H	3.60368	-3.80337	-0.53915
H	4.45904	-2.41994	-1.20735
H	4.02560	-2.44448	0.50474
H	0.29235	-2.59126	-0.35011
C	-2.53949	-0.73510	0.39832
C	-3.25416	-1.87410	0.74073
C	-4.44191	0.22354	-0.77657
C	-4.57225	-1.94934	0.30711
H	-2.81322	-2.67296	1.32019
C	-5.15549	-0.92000	-0.43992
H	-4.89311	1.01761	-1.35436
H	-5.16033	-2.82281	0.55403
H	-6.18391	-1.01587	-0.76052
Cl	0.40975	2.26169	2.48256
Cl	0.63540	2.02287	-2.28801
O	2.43251	2.82161	0.12014

CAAC_MIC_diazo_pdIV_oxo_s (*NImag* = 0)

N	1.56846	1.20965	1.34737
C	0.97290	0.67753	0.20534
C	0.12971	1.63900	-0.30060
N	0.24641	2.71692	0.56418
C	1.10875	2.44674	1.54564
Pd	-1.17520	1.44574	-1.91456
Cl	0.69602	0.64494	-3.21536
C	1.25082	-0.71235	-0.23314
C	-0.61930	3.88723	0.51013
C	-0.74913	4.44692	-0.90863
C	-0.58852	3.40129	-1.99635
N	0.09894	3.97858	-2.95982
C	0.63089	5.24149	-2.55562
C	0.22489	5.52201	-1.32424
C	0.31190	3.51266	-4.32827
C	-2.17816	5.05710	-1.06012
H	1.37657	3.09676	2.35868
O	-2.20857	0.27960	-2.78492
Cl	-3.01924	1.80034	-0.40370
H	0.41715	6.42777	-0.77155
H	1.24452	5.79611	-3.24682
H	-0.22345	4.64412	1.18591
H	-1.60174	3.56381	0.84979
H	-2.92099	4.28327	-0.88181
H	-2.29724	5.86242	-0.33329
H	-2.30913	5.47156	-2.05819
H	0.08405	4.34348	-4.99768
H	1.34276	3.19258	-4.46091
H	-0.33805	2.67052	-4.52781
H	1.08836	-1.42160	0.58277
H	0.59175	-0.96492	-1.05775
H	2.27605	-0.82715	-0.59267
C	2.53985	0.53246	2.18932
H	2.84840	1.20259	2.98789
H	2.09729	-0.36298	2.62291
H	3.41043	0.24891	1.59999

CAAC_MIC_pdIV_oxo_t (*NImag* = 0)

N	1.59585	1.29539	1.43061
C	1.18088	0.80794	0.28796
C	0.21447	2.92278	0.71281
C	0.99766	2.56098	1.72284
H	-0.33489	3.84677	0.62427
H	1.23938	3.05965	2.64766
C	0.36135	1.88878	-0.37971
C	-0.92952	1.39497	-1.04375
H	-1.64141	2.21324	-1.10851
H	-0.69687	1.02868	-2.04340
C	1.25897	2.47513	-1.51038
H	1.48286	1.69355	-2.23405
H	0.73096	3.29284	-2.00388
H	2.18698	2.86344	-1.09499
C	-0.97376	-0.88991	-0.03679
C	-2.06374	-1.71985	0.32841
N	-1.55548	0.27776	-0.34157
N	-2.90253	0.16136	-0.20744
N	-3.20678	-1.04191	0.22061
C	-3.86209	1.23364	-0.34116
H	-3.63963	2.04490	0.35353
H	-3.88655	1.61174	-1.36311
H	-4.82637	0.80094	-0.09465
Pd	1.03910	-1.20439	-0.17044
C	2.56549	0.67843	2.33304
H	3.34339	1.41078	2.55135
H	2.06978	0.36800	3.25038
H	2.98926	-0.18951	1.84009
C	-2.04126	-3.14800	0.73577
H	-3.05889	-3.51880	0.84467
H	-1.52026	-3.74448	-0.01434
H	-1.49545	-3.26675	1.67034
O	2.88839	-1.65285	-0.27478
Cl	0.79717	-1.01148	-2.58231
Cl	0.96208	-2.15537	2.04525

CAAC_NHC_pdIV_oxo_s (*NImag* = 0)

N	-1.83093	-0.45135	-0.61153
C	-0.81717	-0.25804	0.17354
C	-2.03822	1.76378	0.15314
C	-2.76314	0.72822	-0.73073
H	-2.73627	2.36018	0.73928
C	-1.08004	0.95124	1.04277
C	0.17794	1.71436	1.46718
H	-0.06747	2.76374	1.62895
H	0.55360	1.29138	2.39789
C	-1.76656	0.46260	2.33663
H	-1.10616	-0.21531	2.87371
H	-1.99007	1.32524	2.96855
H	-2.69778	-0.05684	2.12773
C	1.69120	0.47750	-0.02138
N	1.25657	1.64647	0.49446
Pd	0.81947	-1.42651	0.12251
C	-2.10387	-1.66097	-1.38643
H	-3.13978	-1.94660	-1.20663
H	-1.94042	-1.47904	-2.44432
H	-1.43804	-2.45336	-1.06199
C	3.71615	-0.17408	-1.33209
H	3.49519	-0.18081	-2.39647
H	4.75124	0.11277	-1.15421
H	3.53870	-1.16965	-0.93888
N	2.85063	0.77429	-0.64010
C	3.13953	2.12222	-0.51467
C	2.13963	2.67337	0.20856
H	4.02121	2.56032	-0.94556
H	1.98401	3.68078	0.54888
H	-1.46280	2.44040	-0.48147
C	-2.79452	1.16787	-2.19427
H	-3.32935	0.45438	-2.82052
H	-3.30855	2.12759	-2.26571
H	-1.78252	1.28197	-2.58334
C	-4.17385	0.39091	-0.25063
H	-4.78940	1.28961	-0.30152
H	-4.64082	-0.36051	-0.88646
H	-4.18659	0.02934	0.77509
O	0.84376	-3.22072	0.15253
Cl	1.22014	-1.38854	2.47016
Cl	0.87907	-1.43993	-2.30180

CAAC_NHC_pdIV_oxo_t (*NImag* = 0)

N	-1.88976	-0.47301	-0.60569
C	-0.91200	-0.35405	0.22930
C	-2.02388	1.74448	0.16773
C	-2.76559	0.74788	-0.74609

H	-2.71319	2.35688	0.74817
C	-1.10561	0.88806	1.06205
C	0.18967	1.58975	1.48016
H	-0.02874	2.62886	1.72462
H	0.59073	1.09594	2.36430
C	-1.79087	0.43567	2.36965
H	-1.15818	-0.28383	2.88851
H	-1.94796	1.30117	3.01726
H	-2.75570	-0.02797	2.17968
C	1.75011	0.44298	-0.04777
N	1.24748	1.58281	0.47772
Pd	0.89028	-1.39859	0.12057
C	-2.15463	-1.65957	-1.41827
H	-3.21526	-1.89683	-1.34691
H	-1.87909	-1.47409	-2.45281
H	-1.56327	-2.48088	-1.02663
C	3.82876	-0.10801	-1.30249
H	3.28185	-0.80255	-1.93402
H	4.52698	0.47235	-1.90085
H	4.37205	-0.65493	-0.53274
N	2.88445	0.81039	-0.67372
C	3.09770	2.17085	-0.53720
C	2.06970	2.65896	0.19210
H	3.94830	2.66224	-0.97247
H	1.85345	3.65532	0.53132
H	-1.41396	2.41073	-0.44527
C	-2.75026	1.19741	-2.20589
H	-3.28258	0.49739	-2.84958
H	-3.24244	2.16771	-2.28765
H	-1.72689	1.29352	-2.56955
C	-4.19573	0.45627	-0.29408
H	-4.78548	1.37137	-0.35812
H	-4.67081	-0.28547	-0.93537
H	-4.23456	0.09753	0.73252
O	0.18812	-3.16560	0.24868
Cl	1.60637	-1.37535	2.43296
Cl	0.97415	-1.58751	-2.31323

CAAC_PPh2_pdIV_oxo_t (*NImag* = 0)

C	3.53320	0.49947	-0.80542
N	2.73962	-0.45092	0.05699
C	1.59596	-0.81688	-0.42819
C	1.42768	-0.26946	-1.84099
C	2.50989	0.83002	-1.90863
C	3.25022	-0.82372	1.37397
Pd	0.01664	-1.51792	0.62521
Cl	-0.97026	-2.93830	-1.23428
C	-0.01487	0.25991	-2.04593
P	-0.86212	0.34827	-0.41738
C	-0.22377	1.84596	0.39581
C	0.00898	3.02962	-0.30806
C	0.54383	4.13677	0.33670
C	0.84478	4.07306	1.69338
C	0.59926	2.90393	2.40397
C	0.06749	1.79296	1.76064
C	1.71222	-1.40476	-2.84270
C	3.89293	1.74289	0.00530
C	4.79314	-0.19125	-1.32452
O	0.95487	-2.84345	1.64820
Cl	-1.78797	-1.45221	2.16926
C	-2.62376	0.61780	-0.69510
C	-3.24903	1.79686	-0.28605
C	-4.61038	1.96963	-0.49821
C	-5.35268	0.96870	-1.11193
C	-4.73350	-0.21286	-1.50564
C	-3.37548	-0.39707	-1.29486
H	2.97867	0.87782	-2.89034
H	-0.02610	1.20831	-2.58176
H	-0.59713	-0.46062	-2.61799
H	1.00711	-2.22023	-2.69857
H	1.60905	-1.01443	-3.85705
H	2.72378	-1.79313	-2.72840
H	2.61439	-1.60011	1.79191
H	4.27082	-1.18934	1.27358
H	3.24653	0.04854	2.02683
H	-2.68275	2.57407	0.20650
H	-2.89981	-1.33184	-1.56003
H	-5.09057	2.88430	-0.17568
H	-5.31249	-1.00351	-1.96444
H	-6.41478	1.10329	-1.27143
H	-0.23122	3.09337	-1.36116
H	-0.13766	0.88391	2.31198
H	0.72425	5.04832	-0.21817
H	0.81920	2.85444	3.46238
H	1.26394	4.93548	2.19529
H	4.63118	1.52938	0.77811
H	4.32164	2.48745	-0.66669
H	3.00651	2.17507	0.47048
H	5.33403	0.49085	-1.98117
H	5.46301	-0.46368	-0.50859
H	4.55502	-1.09154	-1.88810
H	2.06718	1.80273	-1.70133

CAAC_PPh2_pdIV_oxo_s (*NImag* = 0)

N	-2.71904	-0.33824	-0.03908
C	-1.53018	-0.67309	0.34844
C	-2.30839	0.94774	1.89295
H	-2.69044	1.06102	2.90635
C	-1.29221	-0.20919	1.78328
C	0.18564	0.19751	1.97715
H	0.28367	1.13792	2.51708
H	0.71354	-0.57893	2.52245
C	-1.64068	-1.35367	2.75616
H	-0.97115	-2.19501	2.59914
H	-1.52956	-0.98671	3.77843
H	-2.66360	-1.70044	2.62830
Pd	-0.13636	-1.56722	-0.82504
C	-3.36649	-0.76987	-1.27643
H	-2.81385	-1.60177	-1.69822
H	-4.38532	-1.07476	-1.04210
H	-3.37660	0.04019	-2.00078
P	0.92913	0.31485	0.29778
C	2.72643	0.17787	0.39820
C	3.49546	0.81256	-0.58265
C	3.35352	-0.62111	1.35592
C	4.87416	0.66650	-0.58902
H	3.01515	1.41761	-1.34009
C	4.73499	-0.75589	1.34795
H	2.76742	-1.16233	2.08245
C	5.49677	-0.11397	0.37919
H	5.46167	1.15787	-1.35335
H	5.21445	-1.37635	2.09353

H	6.57286	-0.22889	0.37216
C	0.64099	2.07183	-0.11116
C	1.30507	3.04670	0.64420
C	-0.27896	2.46838	-1.07783
C	1.04460	4.39199	0.43808
H	2.03435	2.75293	1.38829
C	-0.53619	3.82127	-1.28179
H	-0.75779	1.71868	-1.69210
C	0.11848	4.78286	-0.52594
H	1.56630	5.13656	1.02539
H	-1.24451	4.11896	-2.04439
H	-0.08077	5.83406	-0.68996
C	-3.43384	0.61833	0.88803
C	-3.85379	1.85705	0.09942
H	-4.65072	1.63718	-0.61074
H	-4.22711	2.60695	0.79831
H	-3.00700	2.28361	-0.43635
C	-4.66084	-0.04115	1.51623
H	-5.11820	0.65545	2.21938
H	-5.40906	-0.28194	0.76113
H	-4.41094	-0.95214	2.05403
H	-1.83457	1.88541	1.60225
O	-0.20107	-3.05402	-1.81900
Cl	-0.55769	-0.34244	-2.83759
Cl	0.79723	-2.89709	0.93099

dmpp_pdIV_oxo_s (*NImag* = 0)

C	-4.86525	1.56259	1.29153
C	-6.39218	1.52079	1.17056
C	-6.98755	0.11515	1.30119
H	-4.54730	1.07321	2.21269
H	-4.51933	2.59928	1.32008
H	-6.81248	2.14223	1.96376
H	-6.71139	1.98201	0.23205
H	-6.63469	-0.35875	2.21780
H	-8.07856	0.17034	1.34198
P	-3.97172	0.72256	-0.06189
P	-6.54777	-1.02527	-0.05607
Pd	-4.30099	-1.57133	-0.36058
Cl	-4.14265	-1.78207	2.03562
Cl	-4.43363	-1.37973	-2.71821
O	-3.31480	-3.05263	-0.56442
C	-7.46823	-0.44029	-1.50838
H	-7.25361	-1.10248	-2.34448
H	-8.53826	-0.42820	-1.29275
H	-7.14304	0.55942	-1.78899
C	-7.32165	-2.60439	0.39570
H	-8.39051	-2.47636	0.57779
H	-7.16761	-3.31399	-0.41636
H	-6.82767	-2.98336	1.28895
C	-2.21740	0.85795	0.38625
H	-1.94208	1.89837	0.56992
H	-2.04469	0.25651	1.27713
H	-1.61709	0.45572	-0.42895
C	-4.17286	1.78803	-1.51876
H	-3.77769	2.78426	-1.31219
H	-3.64690	1.33183	-2.35467
H	-5.22264	1.86424	-1.79398

dmpp_pdIV_oxo_t (*NImag* = 0)

C	-4.82649	1.52150	1.27497
C	-6.34518	1.48534	1.06109
C	-6.98562	0.10546	1.24625
H	-4.56047	0.98454	2.18624
H	-4.49512	2.55737	1.38482
H	-6.80006	2.15979	1.78963
H	-6.60301	1.89580	0.08099
H	-6.64368	-0.34098	2.18198
H	-8.07371	0.20435	1.30363
P	-3.83907	0.77815	-0.07505
P	-6.61549	-1.12732	-0.05273
Pd	-4.32795	-1.44653	-0.47934
Cl	-3.87951	-1.78680	1.88997
Cl	-4.09307	-1.08942	-2.82876
O	-4.61176	-3.32240	-0.78522
C	-7.55756	-0.63607	-1.52554
H	-7.34751	-1.35454	-2.31678
H	-8.62911	-0.60550	-1.31848
H	-7.22126	0.34002	-1.87156
C	-7.35927	-2.66503	0.54688
H	-8.41371	-2.52268	0.79123
H	-7.23998	-3.42674	-0.22071
H	-6.80529	-2.98539	1.42832
C	-2.11200	0.98749	0.44677
H	-1.89648	2.03165	0.67984
H	-1.93851	0.36206	1.32072
H	-1.46061	0.65350	-0.36053
C	-4.04874	1.93432	-1.46221
H	-3.80400	2.95009	-1.14637
H	-3.40435	1.62430	-2.28123
H	-5.07369	1.89932	-1.82650

ethylenediamine_pdIV_oxo_s (*NImag* = 0)

C	1.44940	0.47241	0.48770
C	0.44889	1.22837	-0.35388
H	1.21735	0.57584	1.54550
H	0.63849	1.06754	-1.41297
N	1.39574	-0.96840	0.15757
N	-0.91807	0.74210	-0.06610
Pd	-0.79402	-1.51456	0.01693
H	0.51273	2.30449	-0.15939
H	2.46221	0.85687	0.32527
C	2.02150	-1.77204	1.22821
H	1.94104	-2.82565	0.97100
H	3.07944	-1.50274	1.31529
H	1.50409	-1.60148	2.16530
C	2.11185	-1.24728	-1.10466
H	3.17313	-1.01499	-0.96771
H	1.99420	-2.29635	-1.35824
H	1.70049	-0.66812	-1.92165
C	-1.42347	1.30919	1.20152
H	-1.51365	2.39496	1.09199
H	-2.39554	0.87945	1.42312
H	-0.76568	1.06576	2.02627
C	-1.84796	1.13091	-1.14671
H	-2.83560	0.73542	-0.92122
H	-1.90257	2.22311	-1.20541
H	-1.51174	0.71272	-2.08852
Cl	-0.99403	-1.55235	2.38231
Cl	-0.72464	-1.65434	-2.35193
O	-1.84136	-2.93556	-0.01206

ethylenediamine_pdIV_oxo_t (*NImag* = 0)

C	1.42236	0.40171	0.59223
C	0.46120	1.23899	-0.23085
H	1.13246	0.41384	1.64110
H	0.71397	1.17936	-1.28713
N	1.41798	-1.01472	0.15376
N	-0.92739	0.77873	-0.07101
Pd	-0.72963	-1.57858	0.15127
H	0.54531	2.29101	0.06573
H	2.43545	0.81210	0.51141
C	2.16665	-1.84170	1.12686
H	2.12419	-2.88124	0.80918
H	3.21279	-1.51928	1.16021
H	1.71240	-1.75452	2.10742
C	2.08575	-1.15571	-1.15809
H	3.12164	-0.81034	-1.07310
H	2.06883	-2.19937	-1.45540
H	1.56544	-0.59526	-1.92490
C	-1.55779	1.32265	1.13912
H	-1.69061	2.40570	1.02698
H	-2.52451	0.84820	1.28365
H	-0.95060	1.11089	2.01223
C	-1.75207	1.06527	-1.25203
H	-2.74592	0.65304	-1.09615
H	-1.82763	2.14885	-1.40521
H	-1.31825	0.58972	-2.12604
Cl	-0.76523	-1.53517	2.51559
Cl	-0.74876	-2.02513	-2.15902
O	-2.46585	-2.20378	0.20970

propylenediamine_pdIV_oxo_s (*NImag* = 0)

C	-4.79953	1.44935	1.08801
C	-6.31246	1.40133	0.94908
C	-6.91452	0.01278	1.09044
H	-4.50218	0.92920	1.99548
H	-4.47523	2.49396	1.16573
H	-6.72029	2.00303	1.76455
H	-6.64475	1.88885	0.03180
H	-6.54018	-0.45380	1.99858
H	-8.00505	0.09637	1.16877
N	-4.04827	0.83166	-0.02864
N	-6.61837	-0.91512	-0.02520
Pd	-4.43163	-1.37116	-0.25985
Cl	-4.22775	-1.66479	2.11000
Cl	-4.50320	-1.26829	-2.61760
O	-3.44537	-2.82631	-0.44715
C	-7.29003	-0.48235	-1.26525
H	-7.09764	-1.20393	-2.05082
H	-8.36636	-0.40253	-1.07871
H	-6.91279	0.47735	-1.59808
C	-7.11808	-2.26544	0.32751
H	-8.20232	-2.22679	0.47776
H	-6.89228	-2.95131	-0.48540
H	-6.62939	-2.60460	1.23611
C	-2.60808	0.79866	0.32025
H	-2.24357	1.82092	0.46780
H	-2.47056	0.22077	1.22937
H	-2.05613	0.33442	-0.49345
C	-4.20622	1.61403	-1.26913
H	-3.88199	2.64396	-1.08518
H	-3.60947	1.16744	-2.05604
H	-5.23855	1.61801	-1.59844

propylenediamine_pdIV_oxo_t (*NImag* = 0)

C	-4.77679	1.43910	1.07614
C	-6.28743	1.37028	0.89722
C	-6.91213	-0.00630	1.07931
H	-4.49325	0.90731	1.98143
H	-4.47929	2.48841	1.19065
H	-6.71598	2.00409	1.67746
H	-6.59666	1.82513	-0.04441
H	-6.53081	-0.45615	1.99395
H	-7.99974	0.10636	1.18527
N	-3.97416	0.86850	-0.03518
N	-6.66981	-0.97476	-0.00544
Pd	-4.36683	-1.28762	-0.32437
Cl	-4.03925	-1.68171	1.99041
Cl	-4.32903	-1.11332	-2.66698
O	-4.36890	-3.13414	-0.57558
C	-7.31147	-0.58203	-1.26557
H	-7.09185	-1.32418	-2.02629
H	-8.39580	-0.50611	-1.11787
H	-6.93013	0.37236	-1.61237
C	-7.14835	-2.30836	0.40684
H	-8.22473	-2.26232	0.61174
H	-6.95062	-3.02164	-0.38657
H	-6.61425	-2.62140	1.29928
C	-2.54269	0.91287	0.34738
H	-2.23172	1.95016	0.51336
H	-2.39344	0.33234	1.25294
H	-1.94785	0.48682	-0.45784
C	-4.14278	1.69227	-1.25112
H	-3.91852	2.73728	-1.01171
H	-3.47392	1.33553	-2.02549
H	-5.15407	1.61687	-1.63428

Pincer_Py_bisbenzimi_oxo_s (*NImag* = 0)

C	4.27533	-1.05572	-4.12584
C	3.43327	-1.26210	-3.04354
C	2.72228	-0.18712	-2.47655
C	2.83443	1.10215	-2.96905
C	3.68105	1.30249	-4.05429
C	4.38721	0.24144	-4.62085
N	1.97179	-0.70201	-1.42417
C	2.16154	-2.01677	-1.29249
N	3.06957	-2.38752	-2.29112
C	3.44125	-3.73506	-2.37038
N	2.82390	-4.46705	-1.42508
C	3.02131	-5.79230	-1.30026
C	3.88240	-6.45760	-2.15691
C	4.52708	-5.70633	-3.13904
C	4.31668	-4.33327	-3.26149
N	2.26772	-6.32006	-0.24490
C	2.14215	-7.61816	0.26907
C	1.23441	-7.51022	1.34001
N	0.85735	-6.17358	1.42439
C	1.46203	-5.44049	0.48708
C	2.69703	-8.84523	-0.06187
C	2.32453	-9.95053	0.69962
C	1.42543	-9.83713	1.75990
C	0.86504	-8.60946	2.09667
Pd	1.56794	-3.55162	-0.15757

O	0.40166	-2.70242	1.02234
C	-0.06465	-5.56367	2.37483
C	1.06980	0.01929	-0.53456
H	4.05657	-7.51669	-2.07767
H	5.20549	-6.19964	-3.82094
H	4.82326	-3.76921	-4.02534
H	0.65789	-0.72281	0.16423
H	1.62247	0.79001	0.00440
H	0.27288	0.48372	-1.11691
H	-1.05183	-6.01635	2.27234
H	0.30107	-5.71568	3.39121
H	-0.10211	-4.49201	2.13121
H	3.39392	-8.96293	-0.87579
H	2.74488	-10.91770	0.45950
H	1.15966	-10.71743	2.32938
H	0.16813	-8.51261	2.91737
H	2.28304	1.91872	-2.52431
H	3.79310	2.29673	-4.46514
H	5.03822	0.42571	-5.46465
H	4.83288	-1.85617	-4.58436

Pincer_Py_bisbenzimi_oxo_t (*NImag* = 0)

C	0.81483	-8.63610	2.04556
C	1.19733	-7.52642	1.30670
C	2.12043	-7.62376	0.24857
C	2.67251	-8.84903	-0.09299
C	2.28744	-9.96338	0.64881
C	1.37650	-9.86017	1.70053
N	0.81757	-6.19731	1.40066
C	1.44628	-5.44901	0.46695
N	2.25885	-6.32266	-0.25082
C	3.03169	-5.79446	-1.28680
N	2.79391	-4.45874	-1.42622
C	3.45528	-3.72383	-2.36569
C	4.37291	-4.31079	-3.20417
C	4.61492	-5.68697	-3.06853
C	3.93707	-6.43883	-2.09577
N	3.06313	-2.38524	-2.30301
C	3.41497	-1.26665	-3.06878
C	2.69029	-0.19080	-2.52220
N	1.93888	-0.69222	-1.47172
C	2.14709	-2.01926	-1.32011
C	2.79237	1.09339	-3.03616
C	3.63968	1.28350	-4.12190
C	4.35536	0.21943	-4.67160
C	4.25405	-1.07019	-4.15504
C	1.03884	0.07680	-0.63447
Pd	1.52982	-3.55021	-0.17774
O	0.31657	-2.69049	1.00364
C	-0.12455	-5.65387	2.35956
H	4.13761	-7.49081	-1.98772
H	5.33430	-6.16997	-3.71294
H	4.90629	-3.73707	-3.94233
H	0.58901	-0.60086	0.08841
H	1.58909	0.85958	-0.10987
H	0.25742	0.53320	-1.24414
H	-1.10422	-6.11615	2.22820
H	0.22660	-5.83775	3.37617
H	-0.20044	-4.58215	2.18688
H	3.37260	-8.95750	-0.90493
H	2.70606	-10.92899	0.39978
H	1.10075	-10.74665	2.25564
H	0.10636	-8.55104	2.85765
H	2.23275	1.91366	-2.60886
H	3.74350	2.27228	-4.54801
H	5.00431	0.39529	-5.51870
H	4.81262	-1.87554	-4.60278

Pincer_py_bisimine_oxo_s (*NImag* = 0)

C	-2.01498	1.02957	1.63972
O	0.75616	-1.89675	0.53036
Pd	0.21477	-0.16599	0.32675
C	-1.44675	2.08982	0.81225
N	-0.35865	1.67090	0.11125
C	-1.86643	3.40547	0.66654
C	0.35023	2.46962	-0.73166
C	-1.16302	4.25319	-0.18976
H	-2.72600	3.77624	1.20591
C	-0.05097	3.78917	-0.89313
H	-1.48253	5.27894	-0.30933
H	0.48580	4.45510	-1.55317
C	1.46838	1.76578	-1.35299
C	2.66929	-0.32598	-1.53810
H	2.59910	-0.40862	-2.62667
H	3.65338	0.08088	-1.28776
H	2.54990	-1.30916	-1.08515
N	1.60922	0.51325	-1.01071
C	2.39300	2.43762	-2.31958
H	3.42160	2.40911	-1.95250
H	2.38282	1.92373	-3.28356
H	2.12115	3.47626	-2.48612
N	-1.40541	-0.12393	1.57918
C	-3.21884	1.25168	2.50111
H	-4.02624	0.57245	2.21773
H	-2.98522	1.05144	3.54934
H	-3.59002	2.26993	2.42497
C	-1.84429	-1.27953	2.33955
H	-2.86658	-1.56099	2.07046
H	-1.16226	-2.09423	2.10056
H	-1.81621	-1.07592	3.41401

Pincer_py_bisimine_oxo_t (*NImag* = 0)

C	-2.01296	1.03601	1.63589
O	0.77723	-1.97278	0.53735
Pd	0.21865	-0.18025	0.32802
C	-1.45099	2.08244	0.81809
N	-0.35756	1.66744	0.11175
C	-1.86645	3.39778	0.66863
C	0.35812	2.46469	-0.73615
C	-1.16123	4.24583	-0.18959
H	-2.72599	3.76955	1.20744
C	-0.04710	3.78218	-0.89444
H	-1.48091	5.27143	-0.30946
H	0.48885	4.44881	-1.55452
C	1.46293	1.77045	-1.35020
C	2.68263	-0.30272	-1.55633
H	2.61041	-0.37308	-2.64615
H	3.66283	0.11549	-1.30708
H	2.61275	-1.30207	-1.13694
N	1.60815	0.50276	-1.00603
C	2.39080	2.43623	-2.31675
H	3.42082	2.41014	-1.95217

H	2.38094	1.92656	-3.28351
H	2.11816	3.47499	-2.48106
N	-1.39851	-0.13254	1.57680
C	-3.21560	1.25160	2.49938
H	-4.02616	0.57456	2.21807
H	-2.98438	1.05452	3.54924
H	-3.58557	2.27015	2.42168
C	-1.86897	-1.26447	2.35368
H	-2.89490	-1.53436	2.08443
H	-1.22100	-2.11256	2.15281
H	-1.84458	-1.04976	3.42660

Pincer_Py_bisMIC_oxo_t (*NImag* = 0)

O	0.00211	-2.81521	0.27914
Pd	0.00360	-0.93929	0.18239
C	-1.13368	1.75194	0.04822
C	-1.19802	3.12778	-0.02170
C	1.21245	1.72202	0.05015
C	0.05202	3.81468	-0.05800
H	-2.13563	3.66014	-0.04868
C	1.24148	3.12591	-0.02319
H	0.06104	4.89427	-0.11450
H	2.18439	3.65400	-0.05380
C	2.00714	-0.56173	0.16474
C	3.60482	1.06960	0.08914
H	4.10442	2.01856	0.08704
N	2.26598	0.84756	0.09298
C	-2.00880	-0.53328	0.16095
C	-3.54611	1.13540	0.07959
H	-4.00439	2.10560	0.03186
N	-2.23772	0.86198	0.09239
N	-4.19566	-0.02966	0.13730
N	4.21417	-0.14621	0.18411
C	-3.26494	-1.07472	0.18818
C	3.23761	-1.14100	0.21141
C	5.63960	-0.34547	0.07598
H	6.16241	0.50010	0.52289
H	5.92243	-1.24812	0.61291
H	5.95552	-0.44373	-0.96723
C	-5.63904	-0.18076	0.14684
H	-5.95806	-0.77499	-0.70876
H	-5.95647	-0.67994	1.06178
H	-6.10531	0.80011	0.09455
C	-3.65414	-2.50522	0.25935
H	-4.24115	-2.81951	-0.60838
H	-2.73897	-3.09534	0.28503
H	-4.23490	-2.73329	1.15768
C	3.55383	-2.59038	0.28904
H	2.61265	-3.13865	0.29818
H	4.14374	-2.93157	-0.56653
H	4.10657	-2.84347	1.19866
N	0.00321	1.07096	0.08360

Pincer_Py_bisMIC_oxo_s (*NImag* = 0)

O	-0.03674	-2.76192	0.14622
Pd	-0.01188	-0.90798	0.13437
C	-1.14867	1.76880	0.12036
C	-1.17113	3.14653	0.11123
C	1.19535	1.73786	0.11362
C	0.05058	3.82895	0.10312
H	-2.10543	3.69036	0.11030
C	1.25390	3.11451	0.10431
H	0.06483	4.90896	0.09594
H	2.20208	3.63374	0.09795
C	1.99325	-0.56331	0.12634
C	3.59855	1.05650	0.11036
H	4.10444	2.00345	0.10263
N	2.26955	0.83389	0.11631
C	-2.00719	-0.51046	0.13764
C	-3.56898	1.15138	0.13214
H	-4.04945	2.11147	0.12740
N	-2.24635	0.89354	0.12934
N	-4.19712	-0.03169	0.14196
N	4.19516	-0.14278	0.11625
C	-3.26002	-1.06180	0.14551
C	3.23102	-1.14766	0.12603
C	5.63096	-0.35356	0.11288
H	6.13919	0.60771	0.10439
H	5.92742	-0.90921	1.00190
H	5.92142	-0.92172	-0.77018
C	-5.63800	-0.20454	0.14792
H	-5.94322	-0.75225	1.03892
H	-6.12097	0.76970	0.14261
H	-5.94903	-0.76482	-0.73318
C	-3.58730	-2.50933	0.15630
H	-4.16057	-2.81384	-0.72446
H	-2.62998	-3.03875	0.15621
H	-4.15378	-2.80214	1.04540
C	3.51980	-2.60338	0.13467
H	2.54887	-3.10738	0.14023
H	4.07958	-2.92264	-0.74956
H	4.08368	-2.91131	1.02032
N	0.01438	1.07416	0.12160

Pincer_py_bisNHC_oxo_s (*NImag* = 0)

O	0.52458	-2.36232	0.23208
Pd	0.10058	-0.55083	0.17450
C	-1.43858	1.84373	0.77656
N	-0.35595	1.40016	0.11264
C	-1.78758	3.18102	0.75931
C	0.42734	2.23694	-0.59158
C	-0.97650	4.05330	0.02883
H	-2.65577	3.54258	1.29050
C	0.14732	3.58877	-0.65932
H	-1.22254	5.10519	-0.00441
H	0.77280	4.26510	-1.22323
C	1.57490	0.17186	-0.98619
C	2.53132	1.97971	-1.99783
C	3.26402	0.88062	-2.29208
H	2.66406	3.00508	-2.28837
H	4.15349	0.78238	-2.88858
N	1.48559	1.54341	-1.18986
N	2.66258	-0.20425	-1.66597
C	3.07828	-1.60349	-1.68264
H	2.34885	-2.15186	-1.07147
H	3.07705	-1.97798	-2.70662
H	4.07875	-1.69762	-1.25961
C	-1.55738	-0.48822	1.31053
C	-3.22962	0.76570	2.22584
C	-3.40319	-0.52435	2.59606
H	-3.80715	1.64141	2.45606
H	-4.16500	-0.97056	3.21020

C	-2.12723	-2.70043	2.13414
H	-1.97417	-2.97312	3.17866
H	-2.97633	-3.25367	1.73198
H	-1.22197	-2.90426	1.54659
N	-2.37479	-1.26577	2.02739
N	-2.08804	0.79032	1.43027

Pincer_py_bisNHC_oxo_t (*NImag* = 0)

O	0.53693	-2.41382	0.23428
Pd	0.10463	-0.56735	0.17532
C	-1.44543	1.84438	0.78137
N	-0.35041	1.37734	0.11362
C	-1.78857	3.16999	0.76221
C	0.43346	2.24032	-0.59623
C	-0.97848	4.06189	0.02857
H	-2.65856	3.52592	1.29567
C	0.15322	3.57918	-0.66150
H	-1.22424	5.11230	-0.00471
H	0.78275	4.25111	-1.22749
C	1.57931	0.17943	-0.99034
C	2.53082	1.98236	-1.99828
C	3.26988	0.88809	-2.29792
H	2.65588	3.00979	-2.28388
H	4.15895	0.79444	-2.89526
N	1.48994	1.54340	-1.19268
N	2.67753	-0.19910	-1.67725
C	3.14189	-1.57466	-1.73228
H	2.45793	-2.18090	-1.14107
H	3.14643	-1.92985	-2.76325
H	4.14766	-1.64967	-1.31766
C	-1.56447	-0.48306	1.31467
C	-3.23054	0.76827	2.22586
C	-3.41207	-0.52001	2.60114
H	-3.80205	1.64891	2.45096
H	-4.17576	-0.96194	3.21555
C	-2.19778	-2.69990	2.18273
H	-2.05897	-2.95761	3.23317
H	-3.06011	-3.23774	1.78750
H	-1.30768	-2.97444	1.61953
N	-2.39056	-1.26711	2.03859
N	-2.09179	0.78862	1.43343

Pincer_Py_bis_NHCS_oxo_s (*NImag* = 0)

C	3.59873	1.28488	0.26961
N	2.17931	0.94681	0.16240
C	1.96863	-0.36703	-0.21663
N	3.14013	-0.94213	-0.37387
C	4.27718	-0.05244	-0.11198
C	1.09060	1.76613	0.39231
N	-0.06272	1.10975	0.19643
C	-1.26272	1.68745	0.35636
C	-1.35623	3.01969	0.74004
C	-0.16121	3.71265	0.94657
C	1.08316	3.10126	0.77729
N	-2.28677	0.79745	0.09419
C	-1.97734	-0.49879	-0.27736
N	-3.10244	-1.15041	-0.47095
C	-4.30386	-0.33878	-0.24536
C	-3.72821	1.04003	0.15705
Pd	0.01028	-0.81821	-0.35841
O	0.07821	-2.61244	-0.87451
C	-3.11396	-2.54483	-0.87412
C	3.25713	-2.33224	-0.77519
H	2.00118	3.64679	0.93979
H	-0.20050	4.75125	1.24583
H	-2.31317	3.50248	0.87399
H	-4.02100	1.82938	-0.53734
H	-4.91851	-0.77834	0.54304
H	-2.06211	-2.86040	-0.95774
H	-3.63694	-3.14708	-0.12654
H	-3.62388	-2.65327	-1.83508
H	3.85974	2.09203	-0.41695
H	4.90284	0.03861	-1.00245
H	3.79533	-2.89923	-0.01100
H	2.23178	-2.71715	-0.89128
H	3.80308	-2.40529	-1.71953
H	-4.02958	1.33665	1.16315
H	-4.90565	-0.28779	-1.15528
H	3.84819	1.60079	1.28400
H	4.89430	-0.45034	0.69639

Pincer_Py_CAAC_oxo_s (*NImag* = 0)

C	3.23314	-1.40875	0.01080
C	1.97080	-0.62201	-0.07229
N	2.25480	0.69592	-0.07617
C	3.71789	1.03622	-0.01617
C	4.35250	-0.36255	-0.20569
Pd	0.00009	-1.01024	-0.08771
C	-1.97092	-0.62216	-0.07226
N	-2.25481	0.69579	-0.07619
C	-3.71783	1.03625	-0.01626
C	-4.35260	-0.36250	-0.20554
C	-3.23330	-1.40878	0.01094
C	4.05864	1.63465	1.34997
C	4.12875	1.96753	-1.15630
C	1.18625	1.60475	-0.06080
N	0.00001	0.96179	-0.10049
C	-1.18626	1.60465	-0.06085
C	-1.21676	2.98749	0.01725
C	-0.00012	3.66758	0.05436
C	1.21660	2.98760	0.01731
C	-4.05860	1.63497	1.34977
C	-4.12855	1.96738	-1.15659
C	-3.22002	-2.51746	-1.05002
C	-3.27516	-2.05942	1.40909
C	3.21981	-2.51733	-1.05026
C	3.27509	-2.05951	1.40891
O	0.00004	-2.85714	-0.01233
H	-2.14236	3.53459	0.06278
H	-0.00018	4.74662	0.12078
H	2.14214	3.53479	0.06286
H	4.74148	-0.44865	-1.22066
H	2.33755	-3.14045	-0.89785
H	3.17298	-2.09310	-2.05510
H	4.12709	-3.12195	-0.97274
H	-5.19000	-0.50150	0.47783
H	-4.12724	-3.12217	-0.97240
H	-3.17332	-2.09332	-2.05491
H	-2.33768	-3.14048	-0.89763
H	-4.74167	-0.44871	-1.22047
H	2.41725	-2.72113	1.52063
H	4.19820	-2.63430	1.51466

H	3.24134	-1.31351	2.20483
H	-4.19832	-2.63409	1.51506
H	-2.41740	-2.72116	1.52078
H	-3.24121	-1.31339	2.20498
H	-3.54049	2.57686	1.52665
H	-5.13115	1.82554	1.40563
H	-3.79231	0.94548	2.15007
H	-5.21775	2.00769	-1.20206
H	-3.76789	2.98627	-1.03087
H	-3.76268	1.58615	-2.11033
H	5.18996	-0.50166	0.47758
H	5.13119	1.82527	1.40589
H	3.54049	2.57648	1.52703
H	3.79239	0.94498	2.15013
H	3.76860	2.98654	-1.03012
H	5.21796	2.00740	-1.20201
H	3.76252	1.58678	-2.11010

Pincer_Py_CAAC_oxo_t (*NImag* = 0)

C	3.23135	-1.40794	0.02006
C	1.96552	-0.61669	-0.07840
N	2.25168	0.70967	-0.07781
C	3.71142	1.04156	-0.01320
C	4.34165	-0.35653	-0.21574
Pd	0.00000	-1.02068	-0.09617
C	-1.96552	-0.61670	-0.07841
N	-2.25168	0.70967	-0.07781
C	-3.71143	1.04155	-0.01319
C	-4.34165	-0.35653	-0.21577
C	-3.23135	-1.40795	0.02003
C	4.05710	1.62978	1.35676
C	4.13358	1.97924	-1.14472
C	1.19209	1.60861	-0.06503
N	-0.00000	0.95953	-0.10606
C	-1.19209	1.60861	-0.06503
C	-1.21818	2.98936	0.01044
C	-0.00001	3.67165	0.04667
C	1.21816	2.98936	0.01045
C	-4.05711	1.62971	1.35681
C	-4.13358	1.97928	-1.14466
C	-3.25126	-2.52619	-1.02823
C	-3.29814	-2.03719	1.42554
C	3.25128	-2.52620	-1.02818
C	3.29813	-2.03718	1.42558
O	0.00003	-2.90255	-0.04778
H	-2.14385	3.53621	0.05725
H	-0.00001	4.75059	0.11270
H	2.14384	3.53621	0.05726
H	4.70762	-0.43974	-1.23948
H	2.39904	-3.18984	-0.88168
H	3.18809	-2.11435	-2.03674
H	4.17525	-3.10430	-0.94711
H	-5.19475	-0.49638	0.44782
H	-4.17523	-3.10429	-0.94717
H	-3.18806	-2.11433	-2.03678
H	-2.39902	-3.18984	-0.88172
H	-4.70759	-0.43972	-1.23952
H	2.44740	-2.70183	1.57206
H	4.22188	-2.61131	1.53146
H	3.27286	-1.27823	2.20854
H	-4.22190	-2.61133	1.53140
H	-2.44741	-2.70184	1.57203
H	-3.27289	-1.27826	2.20851
H	-3.54700	2.57550	1.53484
H	-5.13161	1.80919	1.41591
H	-3.78130	0.94097	2.15430
H	-5.22337	2.00474	-1.19218
H	-3.78704	3.00112	-1.00614
H	-3.75932	1.61389	-2.10129
H	5.19474	-0.49637	0.44788
H	5.13160	1.80926	1.41586
H	3.54699	2.57558	1.53475
H	3.78129	0.94107	2.15429
H	3.78704	3.00108	-1.00624
H	5.22337	2.00469	-1.19223
H	3.75932	1.61381	-2.10134

Pincer_Py_DAC_oxo_s (*NImag* = 0)

C	-1.94175	-0.51781	0.11647
N	-2.28259	0.84654	0.06543
C	-3.66668	1.03645	0.03004
C	-4.22337	-0.40609	0.06545
N	-3.08779	-1.21789	0.11545
C	-1.25544	1.80187	0.05607
N	-0.05941	1.21415	0.10149
C	1.07970	1.90745	0.10177
C	1.07044	3.29178	0.05496
C	-0.17920	3.91058	0.00791
C	-1.36881	3.18148	0.00721
N	2.18774	1.04874	0.15295
C	1.96926	-0.34066	0.19269
N	3.17244	-0.93542	0.23660
C	4.23169	-0.02503	0.22954
C	3.54958	1.36204	0.17076
Pd	0.03119	-0.80845	0.16654
O	0.11410	-2.65810	0.22240
C	3.31670	-2.39018	0.28632
C	-3.10382	-2.67978	0.16363
H	1.99166	3.85199	0.05514
H	-0.22730	4.99011	-0.02949
H	-2.33585	3.65676	-0.02982
O	-4.27083	2.06858	-0.01659
O	-5.36946	-0.74955	0.05288
H	-2.06006	-3.00684	0.18900
H	-3.63959	-3.00270	1.05491
H	-3.61597	-3.06115	-0.71831
O	4.06018	2.44421	0.14623
O	5.40358	-0.26435	0.26140
H	3.84279	-2.66702	1.19863
H	2.30580	-2.80865	0.27007
H	3.89550	-2.72199	-0.57419

Pincer_Py_DAC_oxo_t (*NImag* = 0)

C	-1.96184	-0.53653	0.11631
N	-2.28884	0.82888	0.06466
C	-3.68003	1.04394	0.02894
C	-4.24942	-0.36207	0.06386
N	-3.12200	-1.20413	0.11469
C	-1.25913	1.76862	0.05519
N	-0.05796	1.18418	0.09973
C	1.08639	1.87485	0.10050
C	1.07233	3.26037	0.05429

C	-0.17770	3.87509	0.00792
C	-1.36795	3.14988	0.00707
N	2.19570	1.03197	0.15141
C	1.99096	-0.35710	0.19266
N	3.20506	-0.91868	0.23709
C	4.25364	0.02107	0.22854
C	3.56233	1.37082	0.16928
Pd	0.03159	-0.81436	0.16633
O	0.12104	-2.74703	0.23001
C	3.42653	-2.35913	0.28983
C	-3.21670	-2.65847	0.16180
H	1.99550	3.81683	0.05506
H	-0.22591	4.95504	-0.02880
H	-2.33665	3.62118	-0.02912
O	-4.25089	2.10387	-0.01794
O	-5.40163	-0.71441	0.05203
H	-2.20316	-3.05465	0.19156
H	-3.77574	-2.95416	1.04831
H	-3.74753	-3.01305	-0.72038
O	4.03750	2.47757	0.14288
O	5.43232	-0.22648	0.26185
H	3.97278	-2.60628	1.19888
H	2.45147	-2.84353	0.27859
H	4.02234	-2.66302	-0.56947

Pincer_Py_NHCS_oxo_t (*NImag* = 0)

C	3.59177	1.32211	0.12018
N	2.18136	0.94346	0.14621
C	1.97351	-0.35185	-0.26100
N	3.17069	-0.90644	-0.49250
C	4.27407	-0.04527	-0.06501
C	1.09706	1.76566	0.37309
N	-0.06179	1.08087	0.18684
C	-1.26905	1.67908	0.36228
C	-1.35872	3.00103	0.73887
C	-0.16165	3.71537	0.93382
C	1.08596	3.09115	0.74805
N	-2.28883	0.78749	0.09962
C	-1.98230	-0.50815	-0.23789
N	-3.13158	-1.18561	-0.35352
C	-4.30293	-0.31219	-0.27137
C	-3.72066	0.99208	0.30293
Pd	0.01044	-0.83212	-0.36556
O	0.07781	-2.65709	-0.89181
C	-3.21654	-2.52623	-0.88226
C	3.36017	-2.31560	-0.74236
H	2.00833	3.63451	0.89541
H	-0.20094	4.75390	1.22860
H	-2.31971	3.47515	0.87785
H	-4.07472	1.87788	-0.22308
H	-5.07357	-0.74472	0.36781
H	-2.22109	-2.96723	-0.86418
H	-3.89631	-3.12908	-0.27585
H	-3.58300	-2.51750	-1.91504
H	3.78756	1.99731	-0.71674
H	5.06774	-0.02089	-0.81256
H	3.72285	-2.82799	0.15612
H	2.40310	-2.74634	-1.03205
H	4.08662	-2.46185	-1.54484
H	-3.93902	1.10752	1.36762
H	-4.73124	-0.16132	-1.26893
H	3.88756	1.81770	1.04405
H	4.70007	-0.41688	0.87406

Pincer_Py_terpy_oxo_s (*NImag* = 0)

Pd	0.03203	-0.44472	0.17899
C	-1.47101	1.91274	0.80689
C	-1.77995	3.26487	0.77884
C	0.42558	2.28691	-0.59173
C	-0.96558	4.13159	0.05058
H	-2.63891	3.64668	1.31239
C	0.14392	3.64441	-0.63970
H	-1.19568	5.18735	0.02101
H	0.77258	4.31967	-1.20279
C	1.52960	1.57169	-1.23660
C	2.51013	2.16336	-2.03153
C	3.50429	1.37829	-2.58679
H	2.48895	3.22908	-2.20968
C	2.51839	-0.53579	-1.54863
C	3.51177	0.00473	-2.34336
H	4.26892	1.82970	-3.20489
H	2.43776	-1.58870	-1.30149
H	4.27484	-0.63546	-2.76272
C	-2.18494	0.83890	1.50220
C	-3.33181	1.01093	2.27586
C	-3.91578	-0.08544	2.88410
H	-3.75785	1.99679	2.39636
C	-2.21126	-1.46884	1.93845
C	-3.34858	-1.34861	2.71474
H	-4.80596	0.03959	3.48612
H	-1.69733	-2.40450	1.74707
H	-3.78109	-2.22462	3.17680
N	-1.64947	-0.40905	1.35239
N	1.55983	0.22410	-1.01386
O	0.42209	-2.23319	0.22913
N	-0.38524	1.46911	0.12527

Pincer_Py_terpy_oxo_t (*NImag* = 0)

N	1.55393	0.22228	-1.00810
C	1.52596	1.58205	-1.23547
C	2.51359	2.16359	-2.03441
C	3.50498	1.37907	-2.58635
C	3.51097	-0.00013	-2.33918
C	2.51706	-0.53439	-1.54580
C	0.43379	2.29607	-0.59838
N	-0.38737	1.48441	0.12642
C	-1.48181	1.91852	0.81399
C	-1.78561	3.26895	0.78199
C	-0.96606	4.13522	0.05013
C	0.14787	3.65001	-0.64388
C	-2.18590	0.85038	1.50076
C	-3.33527	1.01030	2.27880
C	-3.91615	-0.08443	2.88475
C	-3.34462	-1.35219	2.71277
C	-2.21040	-1.46658	1.93624
N	-1.64340	-0.40799	1.34694
Pd	0.02809	-0.42994	0.17856
O	0.43189	-2.30629	0.22504
H	-2.64409	3.65395	1.31385
H	-1.19652	5.19081	0.01986
H	0.77419	4.32743	-1.20690
H	2.49300	3.22910	-2.21492

H	4.27057	1.82816	-3.20472
H	2.44889	-1.58842	-1.30706
H	4.27229	-0.64362	-2.75555
H	-3.76276	1.99559	2.40055
H	-4.80583	0.03820	3.48773
H	-1.70767	-2.40799	1.75279
H	-3.77334	-2.23055	3.17293

Pincer_Ph_bisbenzimi_CO (*NImag* = 0)

C	0.85646	-8.63892	2.19238
C	1.21970	-7.53705	1.43163
C	2.12005	-7.62955	0.36278
C	2.69168	-8.85113	0.02237
C	2.32994	-9.95413	0.78229
C	1.42878	-9.85412	1.84855
N	0.83130	-6.19818	1.51943
C	1.43573	-5.46881	0.56895
N	2.22662	-6.33492	-0.14209
C	2.97789	-5.77861	-1.20799
C	2.76837	-4.41817	-1.33571
C	3.41021	-3.69593	-2.32430
C	4.28200	-4.32669	-3.20437
C	4.48281	-5.69741	-3.05841
C	3.84229	-6.44232	-2.07084
N	3.05887	-2.32269	-2.29267
C	3.44466	-1.23128	-3.06880
C	2.75487	-0.13524	-2.53534
N	1.99245	-0.61674	-1.46862
C	2.17072	-1.93848	-1.32057
C	2.88333	1.14519	-3.05395
C	3.73486	1.30015	-4.13716
C	4.42795	0.20962	-4.67518
C	4.29810	-1.07007	-4.15524
C	1.13935	0.24486	-0.66970
Pd	1.51373	-3.49574	-0.10009
C	0.22126	-2.58727	1.09289
O	-0.53611	-2.07410	1.75570
C	-0.10410	-5.72318	2.52323
H	4.02828	-7.50095	-1.99953
H	5.15847	-6.20080	-3.73580
H	4.79920	-3.79416	-3.98494
H	0.72116	-0.32426	0.14981
H	1.72672	1.06869	-0.26612
H	0.33196	0.64427	-1.28313
H	0.26478	-5.97494	3.51687
H	-0.20020	-4.64853	2.44434
H	-1.07944	-6.18547	2.37290
H	3.38747	-8.95863	-0.79287
H	2.75735	-10.91776	0.54195
H	1.17477	-10.74004	2.41353
H	0.15995	-8.55949	3.01478
H	2.34574	1.98538	-2.63812
H	3.86566	2.28012	-4.57435
H	5.08362	0.36678	-5.52040
H	4.84395	-1.89031	-4.59043

Pincer_Ph_bisCAAC_CO (*NImag* = 0)

Pd	-0.00001	-0.92038	-0.07307
C	-1.20494	1.72315	-0.04070
C	0.00003	1.04886	-0.07675
C	-1.22189	3.11160	0.03719
C	1.20501	1.72311	-0.04065
C	0.00007	3.78196	0.07382
H	-2.13619	3.68028	0.08053
C	1.22201	3.11156	0.03721
H	0.00009	4.86118	0.13710
H	2.13633	3.68020	0.08055
C	2.03895	-0.46661	-0.07056
C	3.77818	1.17010	-0.02249
C	4.41572	-0.22215	-0.21785
H	4.81335	-0.30179	-1.22883
N	2.31433	0.81458	-0.06463
C	3.32591	-2.34744	-1.11019
H	2.55421	-3.10045	-0.95552
H	3.18461	-1.90829	-2.09847
H	4.29190	-2.85426	-1.09905
C	-2.03893	-0.46654	-0.07051
C	-3.77814	1.17019	-0.02264
C	-4.41570	-0.22209	-0.21768
H	-5.24506	-0.37135	0.47118
C	-3.32594	-2.34750	-1.10982
H	-4.29193	-2.85434	-1.09855
H	-3.18473	-1.90847	-2.09817
H	-2.55421	-3.10048	-0.95511
N	-2.31429	0.81465	-0.06471
H	-4.81343	-0.30191	-1.22860
C	3.37086	-1.94475	1.37225
H	2.53622	-2.62866	1.52534
H	4.29622	-2.51727	1.44674
H	3.35801	-1.21003	2.17731
C	-3.37075	-1.94452	1.37256
H	-4.29611	-2.51702	1.44718
H	-2.53610	-2.62841	1.52567
H	-3.35785	-1.20970	2.17754
C	-3.29845	-1.27644	-0.01420
C	3.29847	-1.27652	-0.01443
C	-4.11367	1.77861	1.33899
H	-3.58355	2.71319	1.51392
H	-5.18260	1.98745	1.38206
H	-3.86870	1.09117	2.14813
C	-4.14911	2.10575	-1.17114
H	-5.23605	2.16963	-1.22786
H	-3.76885	3.11569	-1.03967
H	-3.78312	1.71712	-2.12186
H	5.24514	-0.37153	0.47091
C	4.11376	1.77819	1.33928
H	5.18269	1.98699	1.38237
H	3.58367	2.71274	1.51445
H	3.86878	1.09057	2.14826
C	4.14910	2.10593	-1.17078
H	3.76878	3.11583	-1.03909
H	5.23604	2.16988	-1.22750
H	3.78313	1.71749	-2.12159
C	0.00005	-2.90408	0.01809
O	-0.00052	-4.02936	0.10787

Pincer_Ph_Bisimine_CO (*NImag* = 0)

C	-1.21395	3.05602	-0.00014
C	-1.22053	1.65755	-0.00030
C	0.00321	1.00185	-0.00033
C	1.22629	1.65880	-0.00023
C	1.21826	3.05726	-0.00032

C	0.00181	3.73655	-0.00024
C	2.37157	0.74204	-0.00017
C	3.78155	1.24249	-0.00032
Pd	0.00420	-0.93602	-0.00020
C	0.00517	-2.98066	0.00083
O	0.00582	-4.10505	0.00160
C	-2.36488	0.73963	-0.00025
C	-3.77537	1.23863	0.00045
N	2.05687	-0.51806	-0.00020
C	3.05431	-1.57650	-0.00060
N	-2.04889	-0.52016	-0.00044
C	-3.04527	-1.57959	0.00051
H	2.13904	3.62485	-0.00026
H	0.00126	4.81750	-0.00022
H	-2.13531	3.62266	-0.00011
H	-4.06592	-1.20584	-0.00429
H	-2.89724	-2.20790	-0.87907
H	-2.90319	-2.20071	0.88632
H	-4.31304	0.88550	-0.88101
H	-4.31265	0.88436	0.88167
H	-3.80560	2.32364	0.00113
H	4.31952	0.88891	-0.88140
H	3.81068	2.32753	-0.00086
H	4.31923	0.88976	0.88128
H	4.07461	-1.20173	-0.00086
H	2.90980	-2.20101	-0.88356
H	2.91006	-2.20162	0.88192

Pincer_Ph_bisMIC_CO (*NImag* = 0)

Pd	-0.01447	-0.94847	0.17270
C	-1.16949	1.74974	0.01036
C	-1.17681	3.13586	-0.07221
C	1.21522	1.71599	0.00183
C	0.05148	3.79520	-0.11694
H	-2.09545	3.70644	-0.10173
C	1.26094	3.10137	-0.08095
H	0.06653	4.87420	-0.18074
H	2.19496	3.64602	-0.11722
C	2.02835	-0.56352	0.13849
C	3.62606	1.03511	0.03078
H	4.12415	1.98573	-0.03112
N	2.31309	0.81966	0.05162
C	-2.04569	-0.50585	0.15259
C	-3.59842	1.13715	0.05634
H	-4.06978	2.10153	-0.00164
N	-2.29190	0.88477	0.06803
N	-4.23613	-0.03232	0.12995
N	4.23109	-0.15179	0.10051
C	-3.29426	-1.06621	0.19043
C	3.26092	-1.15878	0.16808
C	5.67307	-0.35716	0.10640
H	5.97455	-0.85477	1.02670
H	5.96563	-0.96854	-0.74561
H	6.17046	0.60688	0.04214
C	-5.68318	-0.19761	0.14452
H	-5.99810	-0.79745	-0.70777
H	-5.99229	-0.69020	1.06495
H	-6.15425	0.77999	0.08694
C	-3.71664	-2.48685	0.27837
H	-4.31645	-2.78763	-0.58373
H	-2.83743	-3.12394	0.31052
H	-4.30476	-2.68237	1.17802
C	3.64417	-2.59063	0.25372
H	2.74795	-3.20302	0.29588
H	4.22645	-2.90904	-0.61407
H	4.23593	-2.80136	1.14752
C	-0.04188	-2.90112	0.30292
O	-0.05795	-4.02947	0.38087
C	0.01315	1.03517	0.04781

Pincer_Ph_bisNHC_CO (*NImag* = 0)

C	3.65998	1.26768	0.00222
N	2.30138	1.04209	0.00141
C	2.02302	-0.29536	-0.00032
N	3.22409	-0.89617	-0.00305
C	4.24356	0.04487	0.00071
C	1.19266	1.92856	-0.00023
C	-0.00332	1.24097	0.00365
C	-1.19792	1.93096	0.00209
C	-1.22245	3.31864	-0.00347
C	-0.00057	3.99275	-0.00720
C	1.21996	3.31620	-0.00561
N	-2.30840	1.04672	0.00742
C	-2.03269	-0.29124	0.01977
N	-3.23495	-0.88962	0.02714
C	-4.25255	0.05338	0.01587
C	-3.66655	1.27498	0.00474
Pd	-0.00529	-0.75024	0.01154
C	-0.00711	-2.72835	0.01940
O	-0.00802	-3.85714	0.02388
C	-3.45092	-2.33201	0.02311
C	3.43723	-2.33892	0.01227
H	-2.14934	3.87537	-0.00462
H	0.00051	5.07371	-0.01146
H	2.14797	3.87107	-0.00883
H	4.09391	2.25022	0.00098
H	5.28098	-0.23573	-0.00069
H	2.85127	-2.80601	-0.77527
H	4.48977	-2.53964	-0.16640
H	3.15384	-2.75202	0.97883
H	-4.09854	2.25835	-0.00181
H	-5.29052	-0.22516	0.01937
H	-2.86542	-2.79412	0.81392
H	-4.50375	-2.52930	0.20392
H	-3.16888	-2.75316	-0.94036

Pincer_Ph_DAC_CO (*NImag* = 0)

C	3.68449	1.35730	0.00334
N	2.30987	1.09343	0.00289
C	2.03655	-0.25241	-0.00063
N	3.18164	-0.93660	-0.00246
C	4.30956	-0.05551	-0.00032
C	1.19687	1.97520	0.00492
C	-0.00001	1.28559	0.00317
C	-1.19689	1.97520	0.00455
C	-1.22442	3.36308	0.00775
C	-0.00001	4.03334	0.00951
C	1.22440	3.36308	0.00813
N	-2.30989	1.09343	0.00224
C	-2.03656	-0.25240	-0.00100
N	-3.18165	-0.93659	-0.00323
C	-4.30957	-0.05551	-0.00183

C	-3.68451	1.35730	0.00199
Pd	-0.00000	-0.69310	-0.00174
C	-0.00003	-2.70256	-0.00646
O	0.00010	-3.82817	-0.00882
C	-3.33388	-2.38889	-0.00696
O	-5.45034	-0.37496	-0.00334
O	-4.23818	2.41086	0.00411
C	3.33389	-2.38890	-0.00633
O	5.45033	-0.37497	-0.00122
O	4.23817	2.41085	0.00599
H	-2.15702	3.90695	0.00885
H	-0.00001	5.11433	0.01198
H	2.15700	3.90695	0.00952
H	2.88370	-2.81497	0.88750
H	2.88079	-2.81044	-0.90082
H	4.39942	-2.60335	-0.00856
H	-4.39941	-2.60336	-0.00656
H	-2.88304	-2.81037	-0.90265
H	-2.88143	-2.81500	0.88568

Pincer_Ph_NHCS_CO (*NImag* = 0)

C	-2.06384	-0.49578	-0.27232
N	-2.35160	0.77080	0.09754
C	-3.78471	1.05262	0.16880
C	-4.38610	-0.31191	-0.21276
N	-3.19528	-1.14986	-0.46644
C	-1.28330	1.64108	0.35005
C	-0.05917	1.02921	0.17504
C	1.11579	1.72283	0.37993
C	1.08797	3.05938	0.76777
C	-0.15863	3.66329	0.93898
C	-1.35657	2.97626	0.73664
N	2.24663	0.92733	0.15387
C	2.05418	-0.35501	-0.22375
N	3.23165	-0.92980	-0.39386
C	4.35660	-0.01357	-0.11132
C	3.65527	1.30715	0.25314
Pd	0.01209	-0.87237	-0.37612
C	3.51471	-2.29169	-0.78618
C	-3.37503	-2.52770	-0.86409
H	1.99529	3.62357	0.93440
H	-0.19767	4.70122	1.23986
H	-2.30412	3.47745	0.87880
H	-4.05558	1.84225	-0.53263
H	-4.97898	-0.75272	0.58941
H	-2.41038	-2.99610	-1.01315
H	-3.92103	-3.07459	-0.09274
H	-3.94237	-2.57884	-1.79544
H	3.88518	2.11202	-0.44571
H	4.99145	0.07210	-0.99346
H	4.07257	-2.80290	0.00105
H	2.58871	-2.82341	-0.96526
H	4.11208	-2.30235	-1.69985
H	-4.06481	1.36947	1.17353
H	-5.00276	-0.26785	-1.11084
H	3.89281	1.64488	1.26198
H	4.95713	-0.41368	0.70641
C	0.08247	-2.78022	-0.91947
O	0.12210	-3.86779	-1.22392

Pincer_Ph_terpy_CO (*NImag* = 0)

Pd	0.01044	-0.49200	0.16430
C	-1.48967	1.87283	0.83179
C	-1.76714	3.24188	0.80164
C	0.45446	2.24787	-0.60006
C	-0.93852	4.09449	0.07716
H	-2.61646	3.65117	1.33265
C	0.16579	3.61476	-0.62197
H	-1.15755	5.15298	0.05702
H	0.78700	4.30774	-1.17400
C	1.55612	1.55253	-1.26188
C	2.53458	2.14397	-2.04921
C	3.52715	1.36301	-2.62019
H	2.51399	3.21179	-2.21080
C	2.52959	-0.54592	-1.60226
C	3.52726	-0.00621	-2.39455
H	4.29203	1.81844	-3.23451
H	2.49564	-1.60688	-1.40342
H	4.28159	-0.65168	-2.81993
C	-2.23009	0.82212	1.52670
C	-3.36909	1.00505	2.29892
C	-3.97486	-0.08429	2.90512
H	-3.77510	1.99851	2.42125
C	-2.29398	-1.47646	1.95024
C	-3.43005	-1.34842	2.72952
H	-4.86247	0.05233	3.50791
H	-1.84039	-2.44335	1.78996
H	-3.86977	-2.22425	3.18356
N	-1.70504	-0.42940	1.36286
N	1.56940	0.20226	-1.04869
O	0.65117	-3.59273	0.22223
C	0.42367	-2.49111	0.20191
C	-0.38394	1.41429	0.12813

Pincer_Py_bisbenzimi_CO (*NImag* = 0)

No *NImag=0* structure could be found with the default grid settings of gaussian09 despite of extensive efforts with various starting geometries and convergence or symmetry settings. The reported coordinates and energies relate therefore to single-point calculations with the default g09 grid (int=finegrid) on the optimized structure (*NImag=0*) as could be obtained with an ultrafine grid (int=ultrafine).

C	-5.64385	0.49999	-0.31417
C	-4.26992	0.30861	-0.29108
C	-3.69206	-0.96721	-0.28026
C	-4.49061	-2.10606	-0.29035
C	-5.86486	-1.91672	-0.31281
C	-6.43512	-0.63853	-0.32495
N	-3.21950	1.22916	-0.27385
C	-2.03722	0.59482	-0.25833
N	-2.31578	-0.74812	-0.25961
C	-1.20324	-1.62682	-0.23521
C	-0.00231	-0.94244	-0.21254
C	1.19882	-1.62660	-0.19509
C	1.21786	-3.01655	-0.19444
C	0.00204	-3.68885	-0.21361
C	-1.22202	-3.01678	-0.23522
N	2.31138	-0.74768	-0.18231
C	3.68763	-0.96649	-0.15739
C	4.26528	0.30945	-0.14889

N	3.21468	1.22979	-0.16644
C	2.03266	0.59520	-0.19019
C	5.63918	0.50111	-0.12636
C	6.43060	-0.63726	-0.11100
C	5.86051	-1.91556	-0.11792
C	4.48631	-2.10518	-0.14108
C	3.43586	2.66486	-0.16191
Pd	0.00210	1.04529	-0.23692
C	0.00114	3.01410	-0.44218
O	0.00356	4.13403	-0.59048
C	-3.44105	2.66418	-0.27704
H	-2.13923	-3.58144	-0.25212
H	-0.00194	-4.76995	-0.21318
H	2.13522	-3.58106	-0.18062
H	2.49771	3.17211	0.02002
H	4.13793	2.92384	0.62963
H	3.84101	2.98426	-1.12189
H	-4.14251	2.92738	0.51365
H	-2.50287	3.17267	-0.09876
H	-3.84709	2.97814	-1.23844
H	-4.08036	-3.10195	-0.28169
H	-6.51208	-2.78276	-0.32153
H	-7.51124	-0.53768	-0.34339
H	-6.08175	1.48790	-0.32519
H	6.07700	1.48911	-0.12278
H	7.50672	-0.53619	-0.09372
H	6.50784	-2.78148	-0.10525
H	4.07619	-3.10115	-0.14617

Pincer_Py_bisCAAC_CO (*NImag* = 0)

C	3.24414	-1.29963	0.00364
C	1.95944	-0.52109	-0.10980
N	2.24674	0.82268	-0.07799
C	3.70006	1.15561	-0.01712
C	4.33204	-0.23195	-0.25626
Pd	0.00000	-0.90558	-0.09581
C	-1.95944	-0.52109	-0.10980
N	-2.24673	0.82268	-0.07797
C	-3.70004	1.15563	-0.01712
C	-4.33203	-0.23192	-0.25628
C	-3.24415	-1.29962	0.00364
C	4.05806	1.72157	1.35970
C	4.12120	2.11431	-1.13309
C	1.20096	1.70841	-0.04775
N	0.00001	1.04605	-0.05679
C	-1.20095	1.70841	-0.04774
C	-1.21665	3.08872	0.01347
C	0.00000	3.77278	0.05097
C	1.21666	3.08872	0.01346
C	-4.05806	1.72159	1.35970
C	-4.12115	2.11434	-1.13309
C	-3.33100	-2.42869	-1.02860
C	-3.36270	-1.90495	1.41594
C	3.33098	-2.42871	-1.02859
C	3.36268	-1.90495	1.41595
H	-2.14221	3.63668	0.04742
H	0.00000	4.85212	0.10761
H	2.14222	3.63668	0.04739
H	4.65770	-0.29940	-1.29482
H	2.56626	-3.18320	-0.84894
H	3.19122	-2.04194	-2.03851
H	4.30696	-2.91828	-0.97408
H	-5.21201	-0.37354	0.37121
H	-4.30699	-2.91825	-0.97411
H	-3.19121	-2.04193	-2.03851
H	-2.56629	-3.18320	-0.84894
H	-4.65768	-0.29937	-1.29484
H	2.55421	-2.61405	1.59123
H	4.31579	-2.42979	1.52472
H	3.30011	-1.13803	2.18760
H	-4.31583	-2.42976	1.52471
H	-2.55425	-2.61407	1.59121
H	-3.30011	-1.13803	2.18759
H	-3.54659	2.66352	1.55328
H	-5.13293	1.90183	1.41438
H	-3.78748	1.02267	2.14975
H	-5.21061	2.12163	-1.19773
H	-3.79596	3.13826	-0.96417
H	-3.72404	1.77900	-2.09145
H	5.21200	-0.37357	0.37124
H	5.13293	1.90179	1.41440
H	3.54660	2.66351	1.55327
H	3.78746	1.02265	2.14975
H	3.79601	3.13823	-0.96417
H	5.21065	2.12159	-1.19772
H	3.72410	1.77898	-2.09145
C	-0.00003	-2.77667	-0.01268
O	-0.00004	-3.92135	0.03722

Pincer_py_bisimine_CO (*NImag* = 0)

C	2.42745	-0.38434	-0.01608
Pd	-0.19117	1.23097	-0.13839
C	1.37871	-1.40581	-0.05467
N	0.12456	-0.92442	-0.21725
C	1.60510	-2.76510	0.13365
C	-0.93868	-1.74392	-0.04673
C	0.52023	-3.62873	0.22190
H	2.61195	-3.14262	0.24013
C	-0.76664	-3.11126	0.14125
H	0.67514	-4.68581	0.39081
H	-1.62353	-3.76001	0.25324
C	-2.23518	-1.06482	0.00042
C	-3.42922	0.92612	0.47153
H	-3.88147	1.07041	-0.51516
H	-4.16517	0.42185	1.10610
H	-3.21528	1.90449	0.89328
N	-2.19467	0.16692	0.39099
C	-3.49723	-1.78052	-0.39479
H	-4.21103	-1.82199	0.43194
H	-3.98724	-1.24208	-1.20994
H	-3.30542	-2.79415	-0.73620
N	2.03865	0.78548	0.37318
C	3.83922	-0.71073	-0.41762
H	4.14794	-0.06293	-1.24203
H	4.54033	-0.53617	0.40244
H	3.94520	-1.74018	-0.74891
C	3.00477	1.86644	0.44555
H	3.86010	1.59411	1.07225
H	3.38764	2.13422	-0.54471
H	2.52325	2.74248	0.87158
C	-0.45579	2.97959	-0.69245
O	-0.62722	4.09877	-0.88936

Pincer_Py_bisMIC_CO (*NImag* = 0)

Pd	-0.01519	-1.01522	0.04971
C	-1.14739	1.71866	-0.09261
C	-1.16420	3.06675	0.16283
C	1.19022	1.68675	-0.10131
C	0.05078	3.76126	0.25062
H	-2.10373	3.57183	0.34449
C	1.24565	3.03381	0.15404
H	0.06604	4.81320	0.49282
H	2.19986	3.51306	0.32902
C	2.01481	-0.57211	0.20855
C	3.58493	1.03707	-0.21672
H	4.05666	1.94895	-0.52944
N	2.26479	0.80757	-0.06595
C	-2.03112	-0.51692	0.22333
C	-3.55971	1.13451	-0.19097
H	-4.00861	2.05889	-0.50055
N	-2.24529	0.86907	-0.04946
N	-4.21518	-0.02716	-0.00921
N	4.20976	-0.14202	-0.03939
C	-3.30563	-1.04264	0.23560
C	3.27457	-1.13235	0.21202
C	5.64871	-0.31580	-0.05725
	6.02260	-0.53532	0.94376
H	5.91793	-1.13581	-0.72158
H	6.11521	0.59845	-0.41671
C	-5.65837	-0.16212	-0.01944
H	-5.95405	-0.96794	-0.68997
H	-6.03158	-0.38148	0.98181
H	-6.10225	0.76797	-0.36662
C	-3.73461	-2.44558	0.46975
H	-4.27335	-2.86344	-0.38616
H	-2.85038	-3.05528	0.63763
H	-4.38029	-2.54263	1.34764
C	3.66663	-2.54637	0.44405
H	2.76698	-3.13141	0.61763
H	4.18833	-2.97924	-0.41494
H	4.31486	-2.66071	1.31796
N	0.01111	1.00909	-0.32378
C	-0.04256	-2.90759	0.01689
O	-0.05813	-4.01504	0.33717

Pincer_py_bisNHC_CO (*NImag* = 0)

Pd	0.00020	-0.96873	-0.24754
C	-1.15623	1.90405	-0.14797
N	-0.00007	1.26758	-0.38729
C	-1.20947	3.25933	0.12905
C	1.15613	1.90402	-0.14788
C	-0.00004	3.94756	0.22715
H	-2.15150	3.75856	0.30483
C	1.20937	3.25932	0.12907
H	-0.00003	5.00447	0.45470
H	2.15139	3.75855	0.30486
C	1.99487	-0.32661	0.15796
C	3.59089	1.28210	-0.26277
C	4.21919	0.09943	-0.09796
H	3.98170	2.25472	-0.49754
H	5.26736	-0.13917	-0.13866
N	2.23339	1.02121	-0.11766
N	3.24627	-0.85540	0.15710
C	3.50280	-2.26712	0.35460
H	2.59933	-2.71671	0.75628
H	3.74739	-2.75186	-0.59249
H	4.32579	-2.40645	1.05602
C	-1.99521	-0.32656	0.15749
C	-3.59097	1.28224	-0.26359
C	-4.21939	0.09957	-0.09927
H	-3.98164	2.25495	-0.49826
H	-5.26756	-0.13898	-0.14042
C	-3.50335	-2.26700	0.35358
H	-4.32336	-2.40635	1.05850
H	-3.75243	-2.75080	-0.59281
H	-2.59843	-2.71744	0.75099
N	-3.24659	-0.85533	0.15596
N	-2.23355	1.02127	-0.11789
C	0.00118	-2.69518	-1.00946
O	0.00248	-3.84042	-1.14951

Pincer_Py_NHCS_CO_twist (*NImag* = 0)

C	-3.70467	1.01179	0.17943
N	-2.26139	0.78161	0.11242
C	-1.94166	-0.51483	-0.24984
N	-3.06178	-1.18066	-0.42378
C	-4.26906	-0.38435	-0.17563
C	-1.24525	1.68644	0.35233
N	-0.04044	1.11920	0.19331
C	1.10703	1.79036	0.37106
C	1.08808	3.13163	0.73391
C	-0.16161	3.73273	0.90112
C	-1.35062	3.02391	0.71432
N	2.20251	0.97856	0.14759
C	3.61883	1.33591	0.23163
C	4.30925	-0.00466	-0.11515
N	3.18042	-0.90526	-0.37529
C	2.00353	-0.34107	-0.21734
Pd	0.04872	-0.81381	-0.34177
O	0.13168	-2.61108	-0.84224
C	3.31196	-2.30109	-0.75189
C	-3.06435	-2.58280	-0.79978
H	2.00150	3.68914	0.88079
H	-0.20991	4.77584	1.18293
H	-2.31182	3.49882	0.84565
H	-4.01024	1.77577	-0.53773
H	-4.84843	-0.81800	0.64260
H	-2.01123	-2.88896	-0.89894
H	-3.56448	-3.17628	-0.02973
H	-3.59310	-2.71590	-1.74724
H	3.86379	2.12401	-0.48269
H	4.94796	0.07268	-0.99739
H	3.85438	-2.84880	0.02339
H	2.29144	-2.69940	-0.86324
H	3.86079	-2.38524	-1.69358
H	-4.00172	1.33745	1.17756
H	-4.90288	-0.36239	-1.06453
H	3.87412	1.68660	1.23285
H	4.91615	-0.38419	0.70996

Pincer_Py_bisDAC_CO (*NImag* = 0)

N	-2.23162	1.06005	0.02804
C	-1.96316	-0.31312	0.20723
N	-3.17162	-0.93420	0.05371

C	-4.23215	-0.05824	-0.15355
C	-3.59112	1.32757	-0.16809
Pd	0.00000	-0.73510	0.01937
C	0.00001	-2.56675	-0.47982
O	-0.00006	-3.69997	-0.60811
C	-3.35750	-2.36758	0.17454
C	-1.17009	1.95720	0.01831
N	0.00001	1.31255	-0.05128
C	1.17009	1.95721	0.01856
C	1.21903	3.33964	0.08547
C	-0.00001	4.01601	0.10366
C	-1.21904	3.33964	0.08521
N	2.23163	1.06005	0.02850
C	1.96313	-0.31312	0.20761
N	3.17162	-0.93419	0.05430
C	4.23220	-0.05823	-0.15267
C	3.59117	1.32759	-0.16732
C	3.35746	-2.36758	0.17515
H	-2.16679	3.85220	0.11883
H	-0.00002	5.09609	0.15573
H	2.16676	3.85221	0.11929
O	4.11732	2.40061	-0.29712
O	5.40091	-0.33432	-0.25256
H	2.80495	-2.73352	1.03819
H	3.00360	-2.87467	-0.72303
H	4.42030	-2.55864	0.29973
O	-4.11726	2.40060	-0.29803
O	-5.40084	-0.33435	-0.25370
H	-4.42027	-2.55860	0.29972
H	-3.00421	-2.87464	-0.72389
H	-2.80450	-2.73359	1.03722

Pincer_Py_terpy_CO (*NImag* = 0)

Pd	0.01822	-0.97367	0.04904
C	-1.37298	1.75083	0.85357
C	-1.60641	3.12017	0.95154
C	0.47838	2.10653	-0.50211
C	-0.76535	3.99692	0.28400
H	-2.41378	3.49109	1.56616
C	0.29851	3.48615	-0.44330
H	-0.91553	5.06566	0.35931
H	0.99923	4.14682	-0.93289
C	1.62283	1.49187	-1.20102
C	2.22526	2.08062	-2.31397
C	3.30596	1.44782	-2.90464
H	1.83710	3.00281	-2.72322
C	3.08479	-0.28152	-1.27466
C	3.75354	0.24469	-2.36900
H	3.78507	1.87840	-3.77404
H	3.38417	-1.22666	-0.83834
H	4.59839	-0.27978	-2.79396
C	-2.18420	0.76060	1.58667
C	-3.52777	0.97554	1.89790
C	-4.21669	0.00285	2.60254
H	-4.02854	1.87602	1.57085
C	-2.21679	-1.29973	2.60719
C	-3.54580	-1.15733	2.97487
H	-5.26138	0.14071	2.84836
H	-1.66111	-2.19560	2.85606
H	-4.04287	-1.93955	3.53207
N	-1.54486	-0.37000	1.92736
N	2.04227	0.31893	-0.69958
N	-0.36674	1.25975	0.11022
O	-0.07464	-3.82871	-0.82471
C	-0.07256	-2.71692	-0.53089

Pincer_Ph_bisbenzimi_oxo_s (*NImag* = 0)

C	4.25131	-0.99365	-4.21573
C	3.45645	-1.22167	-3.09930
C	2.67372	-0.19559	-2.54579
C	2.65685	1.08529	-3.07852
C	3.45013	1.30783	-4.19381
C	4.23162	0.28649	-4.74959
N	1.99932	-0.74101	-1.44757
C	2.34336	-2.02560	-1.31029
N	3.21995	-2.33679	-2.29867
C	3.62032	-3.69182	-2.30598
C	3.01314	-4.38204	-1.27030
C	3.19484	-5.74368	-1.14611
C	4.02819	-6.42763	-2.02695
C	4.65330	-5.70815	-3.04064
C	4.45251	-4.33857	-3.20703
N	2.42292	-6.28728	-0.09552
C	2.16160	-7.59537	0.31827
C	1.16876	-7.48068	1.29889
N	0.87825	-6.11957	1.43891
C	1.64086	-5.41561	0.60100
C	2.66583	-8.83921	-0.04452
C	2.14104	-9.94556	0.60720
C	1.14912	-9.82481	1.58814
C	0.64471	-8.58638	1.95312
Pd	1.90478	-3.40206	0.14211
O	1.25816	-3.11473	1.78988
C	-0.12121	-5.62081	2.37786
C	1.08612	0.00055	-0.58849
H	4.19389	-7.48914	-1.94639
H	5.30283	-6.23044	-3.72884
H	4.92730	-3.81669	-4.02299
H	0.69782	-0.66865	0.17387
H	1.61678	0.82293	-0.11052
H	0.26295	0.39331	-1.18357
H	-1.07355	-6.10461	2.16553
H	0.19164	-5.85606	3.39421
H	-0.19312	-4.54547	2.26481
H	3.43441	-8.96016	-0.78965
H	2.51256	-10.92814	0.35143
H	0.77146	-10.71482	2.07172
H	-0.11735	-8.48937	2.71281
H	2.05595	1.87365	-2.64823
H	3.46724	2.29067	-4.64355
H	4.83844	0.50165	-5.61812
H	4.86388	-1.76496	-4.65463

Pincer_Ph_bisbenzimi_oxo_t (*NImag* = 1; -15.4 cm^{-1})

No *NImag=0* structure could be found with the default grid settings or an ultrafine grid of gaussian09 despite of extensive efforts with various starting geometries and convergence criteria or symmetry settings on a very flat potential energy surface. The reported Gibbs free energies were

therefore corrected by 1 kcal mol^{-1} as was suggested by the electronic energies.

C	4.29491	-1.01074	-4.15452
C	3.44636	-1.21177	-3.07293
C	2.73537	-0.14131	-2.51091
C	2.84119	1.15268	-2.99764
C	3.68979	1.34838	-4.07740
C	4.40146	0.28448	-4.64279
N	1.98021	-0.66375	-1.45488
C	2.19101	-1.97573	-1.34496
N	3.08197	-2.33141	-2.32197
C	3.43645	-3.70063	-2.37193
C	2.78830	-4.43993	-1.38781
C	3.00687	-5.80901	-1.27357
C	3.87464	-6.45665	-2.14048
C	4.51316	-5.69311	-3.11888
C	4.31073	-4.31874	-3.25377
N	2.25910	-6.37619	-0.21439
C	2.13518	-7.67073	0.29466
C	1.23012	-7.56433	1.36079
N	0.85420	-6.21886	1.44327
C	1.47265	-5.51078	0.49786
C	2.69445	-8.89721	-0.04213
C	2.31744	-9.99495	0.71968
C	1.41413	-9.88214	1.78231
C	0.85288	-8.65995	2.12220
Pd	1.55063	-3.53789	-0.13914
O	0.38156	-2.68681	1.04591
C	-0.08007	-5.69237	2.42794
C	1.09809	0.13063	-0.61194
H	4.06763	-7.51524	-2.08120
H	5.19267	-6.18651	-3.80007
H	4.83013	-3.77727	-4.02733
H	0.63577	-0.52080	0.12320
H	1.67425	0.90359	-0.10485
H	0.32702	0.59541	-1.22516
H	-1.04908	-6.17558	2.30874
H	0.29986	-5.88251	3.43095
H	-0.18464	-4.62277	2.27459
H	3.39176	-9.01241	-0.85571
H	2.73488	-10.96389	0.48332
H	1.14898	-10.76390	2.34864
H	0.15508	-8.56964	2.94241
H	2.29011	1.97229	-2.55901
H	3.80362	2.34104	-4.48993
H	5.05363	0.47448	-5.48394
H	4.85460	-1.81184	-4.60874

Pincer_Ph_Bisimine_oxo_s (*NImag* = 0)

C	0.10310	3.79121	-0.66304
C	0.37246	2.42202	-0.75072
C	-0.49132	1.58954	-0.06702
C	-1.57328	1.96827	0.70269
C	-1.82335	3.34197	0.77595
C	-0.98288	4.22760	0.09848
C	-2.28736	0.84083	1.34350
C	-3.42102	1.11278	2.27193
Pd	-0.19615	-0.28985	-0.25861
N	-1.82273	-0.32929	1.04833
C	-2.26104	-1.58777	1.62732
C	1.46402	1.71563	-1.45864
C	2.51827	2.49761	-2.16447
O	0.84704	-1.29018	0.82574
N	1.41732	0.42626	-1.37177
C	2.42336	-0.49535	-1.87145
H	-2.65331	3.72860	1.35226
H	-1.17866	5.28859	0.16760
H	0.72478	4.51637	-1.17108
H	2.01701	-1.04544	-2.72221
H	3.34254	0.00500	-2.16483
H	2.63245	-1.20426	-1.07108
H	3.19683	2.93944	-1.42898
H	3.09885	1.89951	-2.85838
H	2.05954	3.31921	-2.71499
H	-4.02989	0.23697	2.46820
H	-3.03052	1.48888	3.22201
H	-4.05912	1.89177	1.85413
H	-2.79151	-2.16737	0.86958
H	-1.36469	-2.13602	1.91521
H	-2.89953	-1.45063	2.49613

Pincer_Ph_Bisimine_oxo_t (*NImag* = 0)

C	-0.98215	4.26287	0.10813
C	0.13360	3.82359	-0.60515
C	0.41991	2.45802	-0.65416
C	-0.43476	1.58891	0.01510
C	-1.54911	2.00659	0.73438
C	-1.82532	3.37448	0.77631
C	1.52534	1.76623	-1.33495
N	1.51642	0.47675	-1.20122
C	2.51154	-0.40462	-1.79276
Pd	-0.04683	-0.31257	-0.05315
O	0.43804	-2.13207	0.04265
C	-2.28491	0.89261	1.35215
N	-1.77924	-0.27884	1.12269
C	-2.34526	-1.51820	1.63313
C	-3.51376	1.11349	2.17476
C	2.56486	2.50723	-2.11333
H	-2.67939	3.76227	1.31483
H	-1.19926	5.32130	0.14434
H	0.75698	4.55006	-1.10861
H	2.00616	-1.12135	-2.44089
H	3.25968	0.13403	-2.36860
H	3.00305	-0.96546	-0.99702
H	3.55994	2.31870	-1.70725
H	2.56648	2.18852	-3.15706
H	2.38505	3.57737	-2.08634
H	-3.37527	0.73079	3.18721
H	-3.75514	2.16966	2.24186
H	-4.36959	0.59574	1.73846
H	-3.24585	-1.35760	2.22014
H	-2.57619	-2.17250	0.79187
H	-1.59760	-2.01979	2.24860

Pincer_Ph_bisMIC_oxo_t

C	3.23601	-1.14656	0.14043
C	2.02026	-0.52359	0.13687
N	2.31483	0.85188	0.11967
C	3.63289	1.04891	0.11323
N	4.21945	-0.15007	0.12559
Pd	-0.01344	-0.93900	0.14830
C	-2.03536	-0.46984	0.14895

N	-2.29300	0.91304	0.13323
C	-3.60531	1.14542	0.13426
N	-4.22375	-0.03737	0.15012
C	-3.26739	-1.05994	0.15954
C	1.21614	1.74889	0.11254
C	0.01333	1.06179	0.12416
C	-1.17090	1.78051	0.11957
C	-1.17901	3.16778	0.10374
C	0.04978	3.82856	0.09237
C	1.26072	3.13545	0.09653
C	-5.66846	-0.22881	0.15683
C	5.65855	-0.38000	0.12387
C	-3.64205	-2.49532	0.17791
C	3.57157	-2.59163	0.15613
O	-0.03878	-2.79166	0.17128
H	-2.09780	3.73868	0.10015
H	0.06408	4.90942	0.08009
H	2.19413	3.68206	0.08739
H	4.14513	1.99394	0.10057
H	-4.09190	2.10391	0.12405
H	6.17245	0.57737	0.11047
H	5.94677	-0.93028	1.01785
H	5.94114	-0.95220	-0.75805
H	-5.96602	-0.77142	1.05245
H	-6.15688	0.74185	0.14650
H	-5.97145	-0.79305	-0.72347
H	-4.23130	-2.77222	-0.69923
H	-2.73497	-3.09587	0.18082
H	-4.22335	-2.75170	1.06653
H	2.64839	-3.16708	0.16667
H	4.14569	-2.88459	-0.72585
H	4.15302	-2.86379	1.03994

Pincer_Ph_bisMIC_oxo_s (*NImag* = 0)

C	-3.54973	1.13480	0.00344
N	-2.23482	0.91787	0.01869
C	-1.96954	-0.45274	0.01524
C	-3.18947	-1.07045	-0.00299
N	-4.15180	-0.05659	-0.00989
C	-1.12548	1.79301	0.03572
C	0.06320	1.08887	0.04623
C	1.24301	1.81609	0.06240
C	1.26592	3.20193	0.06870
C	0.04493	3.87433	0.05803
C	-1.16291	3.18138	0.04133
N	2.34658	0.93279	0.07014
C	2.01340	-0.42019	0.05977
C	3.20047	-1.09950	0.06892
N	4.21315	-0.13512	0.08460
C	3.66991	1.08654	0.08497
Pd	0.04801	-0.97052	0.03840
O	-0.53587	-2.66126	0.02933
C	3.48330	-2.55577	0.06461
C	5.64370	-0.41674	0.09859
C	-3.56083	-2.50452	-0.01482
C	-5.59444	-0.26560	-0.02888
H	-2.09765	3.72579	0.03296
H	0.03521	4.95499	0.06260
H	2.19328	3.75879	0.08135
H	4.21259	2.01419	0.09547
H	-4.04450	2.08869	0.00191
H	6.18979	0.52262	0.10805
H	5.89916	-0.99079	0.98742
H	5.91790	-0.98367	-0.78924
H	-5.87626	-0.81758	-0.92360
H	-5.89814	-0.82798	0.85208
H	-6.09257	0.70015	-0.02934
H	-4.13761	-2.76006	-0.90662
H	-2.64817	-3.09624	-0.00770
H	-4.15726	-2.76959	0.86115
H	2.54196	-3.10118	0.05246
H	4.05901	-2.85325	-0.81450
H	4.04118	-2.86192	0.95222

Pincer_Ph_bisNHC_oxo_s (*NImag* = 0)

C	2.05538	-0.34142	-0.01765
N	2.27347	1.00665	0.05148
C	3.61309	1.27146	0.26763
C	4.22590	0.06498	0.33318
N	3.24705	-0.90683	0.16232
C	1.13072	1.84211	0.01168
C	-0.05275	1.12704	-0.04120
C	-1.29688	1.73265	-0.03553
C	-1.37131	3.11824	-0.02407
C	-0.17600	3.84069	0.00634
C	1.07973	3.22873	0.02364
N	-2.36059	0.79780	-0.03867
C	-3.72592	0.94083	0.12510
C	-4.22995	-0.31592	0.16904
N	-3.16187	-1.19558	0.03792
C	-2.01976	-0.52512	-0.09704
Pd	0.03714	-0.81361	-0.16839
O	0.07020	-2.09603	1.12246
C	-3.25680	-2.65362	0.06721
C	3.47108	-2.35058	0.19759
H	1.97764	3.83029	0.04617
H	-0.22490	4.92069	0.01785
H	-2.31989	3.63654	-0.03751
H	-4.21518	1.89416	0.20184
H	-5.24208	-0.65922	0.28490
H	-2.26711	-3.04746	0.28179
H	-3.94471	-2.94944	0.85602
H	-3.61650	-3.02363	-0.89179
H	4.01138	2.26476	0.36162
H	5.25954	-0.18620	0.48860
H	4.15565	-2.58441	1.00974
H	2.51387	-2.83164	0.37925
H	3.89475	-2.68586	-0.74797

Pincer_Ph_bisNHC_oxo_t

N	-2.30827	0.90210	-0.00641
C	-2.02562	-0.43208	-0.00807
N	-3.21188	-1.04920	-0.01066
C	-4.24155	-0.11623	-0.01063
C	-3.67162	1.11277	-0.00793
Pd	0.00429	-0.89297	-0.00544
C	2.03260	-0.42501	-0.00126
N	2.31065	0.91014	0.00133
C	3.67326	1.12551	0.00402
C	4.24743	-0.10151	0.00309
N	3.22097	-1.03804	-0.00015
C	1.19917	1.79052	0.00086

C	0.00087	1.09512	-0.00225
C	-1.19986	1.78633	-0.00316
C	-1.22665	3.17175	-0.00102
C	-0.00394	3.84734	0.00210
C	1.22112	3.17602	0.00309
C	3.42675	-2.48302	-0.00221
C	-3.41268	-2.49491	-0.01319
O	0.00704	-2.75724	-0.00835
H	-2.15356	3.72819	-0.00170
H	-0.00583	4.92845	0.00380
H	2.14608	3.73570	0.00553
H	4.11492	2.10476	0.00634
H	5.28284	-0.39056	0.00443
H	2.45734	-2.97194	-0.00381
H	3.98296	-2.77224	-0.89220
H	3.98142	-2.77496	0.88785
H	-4.11667	2.09049	-0.00709
H	-5.27596	-0.40882	-0.01259
H	-3.96830	-2.78874	0.87564
H	-3.96597	-2.78597	-0.90440
H	-2.44160	-2.98050	-0.01265

Pincer_Ph_CAAC_oxo_s (*NImag* = 0)

O	-0.44989	-2.80141	0.03051
Pd	0.02611	-1.07842	-0.04535
C	-1.14357	1.65583	-0.03639
C	0.04924	0.96398	-0.06677
C	-1.16104	3.04613	0.02073
C	1.24493	1.66126	-0.05282
C	0.05627	3.72265	0.03787
H	-2.07902	3.60913	0.05980
C	1.27529	3.04737	-0.00028
H	0.05494	4.80247	0.08396
H	2.19837	3.60504	0.02093
C	1.97241	-0.51253	-0.06758
C	3.81122	0.97674	-0.03584
C	4.33287	-0.46880	-0.22442
H	4.70863	-0.58935	-1.23936
N	2.32641	0.73703	-0.07541
C	3.07520	-2.53818	-1.07266
H	2.22219	-3.19638	-0.90904
H	2.99394	-2.10690	-2.07100
H	3.98415	-3.13956	-1.03322
C	-1.98699	-0.51207	-0.06807
C	-3.72374	1.09720	-0.00183
C	-4.34695	-0.30314	-0.19505
H	-5.15346	-0.46893	0.51627
C	-3.23888	-2.38831	-1.16664
H	-4.18703	-2.92745	-1.14000
H	-3.15025	-1.90193	-2.13923
H	-2.42340	-3.10030	-1.05439
N	-2.25095	0.75919	-0.05613
H	-4.76958	-0.37942	-1.19540
C	3.16645	-2.09588	1.40185
H	2.28571	-2.71899	1.55481
H	4.05178	-2.72721	1.48325
H	3.20313	-1.34957	2.19547
C	-3.27792	-2.07840	1.33276
H	-4.20463	-2.65095	1.38668
H	-2.43613	-2.76051	1.43722
H	-3.26392	-1.37148	2.16327
C	-3.22043	-1.36141	-0.02922
C	3.14411	-1.44915	0.00295
C	-4.04268	1.69510	1.36686
H	-3.52268	2.63628	1.53737
H	-5.11375	1.88899	1.42473
H	-3.77845	1.00576	2.16827
C	-4.10950	2.03178	-1.14435
H	-5.19766	2.08020	-1.19260
H	-3.74275	3.04634	-1.01040
H	-3.74657	1.65087	-2.09920
H	5.15252	-0.67966	0.45959
C	4.18394	1.56999	1.32160
H	5.26504	1.70020	1.36803
H	3.72269	2.54400	1.47923
H	3.88454	0.91250	2.13712
C	4.24102	1.87883	-1.18813
H	3.91562	2.90877	-1.05865
H	5.33004	1.88151	-1.23914
H	3.85814	1.50748	-2.13891

Pincer_Ph_CAAC_oxo_t (*NImag* = 0)

O	0.00017	-2.90589	-0.01378
Pd	-0.00000	-1.03777	-0.06863
C	-1.20481	1.62337	-0.04772
C	-0.00003	0.94279	-0.07423
C	-1.22343	3.01088	0.01229
C	1.20474	1.62341	-0.04771
C	-0.00010	3.68138	0.04098
H	-2.13862	3.57920	0.04644
C	1.22327	3.01093	0.01232
H	-0.00012	4.76136	0.09049
H	2.13843	3.57929	0.04648
C	2.03031	-0.55887	-0.06366
C	3.78615	1.04473	-0.01902
C	4.39855	-0.36409	-0.19968
H	4.80204	-0.45923	-1.20687
N	2.31413	0.71488	-0.06516
C	3.25389	-2.48224	-1.08203
H	2.43028	-3.17996	-0.93183
H	3.16167	-2.04516	-2.07712
H	4.18905	-3.04248	-1.04181
C	-2.03035	-0.55892	-0.06371
C	-3.78615	1.04470	-0.01896
C	-4.39856	-0.36412	-0.19987
H	-5.21856	-0.52364	0.49769
C	-3.25380	-2.48230	-1.08215
H	-4.18900	-3.04248	-1.04204
H	-3.16144	-2.04524	-2.07724
H	-2.43026	-3.18008	-0.93185
N	-2.31417	0.71482	-0.06515
H	-4.80195	-0.45917	-1.20710
C	3.30190	-2.06242	1.39617
H	2.44874	-2.72577	1.53539
H	4.21377	-2.65481	1.47990
H	3.29930	-1.32364	2.19789
C	-3.30197	-2.06247	1.39602
H	-4.21389	-2.65478	1.47972
H	-2.44889	-2.72591	1.53528
H	-3.29934	-1.32368	2.19773
C	-3.26145	-1.40087	0.00374
C	3.26144	-1.40081	0.00389

C	-4.12182	1.65838	1.33947
H	-3.60806	2.60478	1.50088
H	-5.19401	1.84799	1.38844
H	-3.85862	0.98286	2.15286
C	-4.17424	1.96525	-1.17270
H	-5.26212	2.01603	-1.22346
H	-3.80514	2.98076	-1.04982
H	-3.80965	1.57370	-2.12269
H	5.21849	-0.52353	0.49798
C	4.12185	1.65862	1.33931
H	5.19405	1.84824	1.38824
H	3.60810	2.60505	1.50055
H	3.85867	0.98325	2.15283
C	4.17437	1.96502	-1.17292
H	3.80538	2.98058	-1.05023
H	5.26225	2.01570	-1.22366
H	3.80978	1.57333	-2.12285

Pincer_Ph_DAC_oxo_s (*NImag* = 0)

C	-2.02279	-0.47420	0.11384
N	-2.36000	0.85977	0.06144
C	-3.74799	1.05158	0.02588
C	-4.29613	-0.39557	0.06362
N	-3.11943	-1.21729	0.11610
C	-1.29078	1.78662	0.05273
C	-0.05613	1.14450	0.10045
C	1.11642	1.89586	0.09966
C	1.08011	3.27824	0.05257
C	-0.17840	3.88907	0.00571
C	-1.37736	3.16679	0.00465
N	2.26385	1.06921	0.15152
C	2.04577	-0.28953	0.19242
N	3.20335	-0.93197	0.23798
C	4.30298	-0.00832	0.22999
C	3.62954	1.38428	0.16932
Pd	0.03137	-0.81216	0.16767
O	0.12073	-2.68988	0.22701
C	3.41102	-2.37784	0.29173
C	-3.19987	-2.67593	0.16554
H	1.98424	3.86842	0.05178
H	-0.22665	4.96866	-0.03130
H	-2.33019	3.67325	-0.03253
O	-4.35327	2.07426	-0.02187
O	-5.41429	-0.78183	0.05320
H	-2.19189	-3.07900	0.19293
H	-3.75007	-2.97339	1.05608
H	-3.72671	-3.03426	-0.71658
O	4.14255	2.45696	0.14431
O	5.45078	-0.29299	0.26342
H	3.94641	-2.62857	1.20537
H	2.44238	-2.86854	0.27564
H	4.00484	-2.68515	-0.56677

Pincer_Ph_DAC_oxo_t (*NImag* = 0)

C	-2.02279	-0.47420	0.11384
N	-2.36000	0.85977	0.06144
C	-3.74799	1.05158	0.02588
C	-4.29613	-0.39557	0.06362
N	-3.11943	-1.21729	0.11610
C	-1.29078	1.78662	0.05273
C	-0.05613	1.14450	0.10045
C	1.11642	1.89586	0.09966
C	1.08011	3.27824	0.05257
C	-0.17840	3.88907	0.00571
C	-1.37736	3.16679	0.00465
N	2.26385	1.06921	0.15152
C	2.04577	-0.28953	0.19242
N	3.20335	-0.93197	0.23798
C	4.30298	-0.00832	0.22999
C	3.62954	1.38428	0.16932
Pd	0.03137	-0.81216	0.16767
O	0.12073	-2.68988	0.22701
C	3.41102	-2.37784	0.29173
C	-3.19987	-2.67593	0.16554
H	1.98424	3.86842	0.05178
H	-0.22665	4.96866	-0.03130
H	-2.33019	3.67325	-0.03253
O	-4.35327	2.07426	-0.02187
O	-5.41429	-0.78183	0.05320
H	-2.19189	-3.07900	0.19293
H	-3.75007	-2.97339	1.05608
H	-3.72671	-3.03426	-0.71658
O	4.14255	2.45696	0.14431
O	5.45078	-0.29299	0.26342
H	3.94641	-2.62857	1.20537
H	2.44238	-2.86854	0.27564
H	4.00484	-2.68515	-0.56677

Pincer_Ph_terpy_oxo_s (*NImag* = 0)

Pd	-0.15368	-0.41779	-0.06535
C	-1.53014	1.89781	0.79944
C	-0.42724	1.47896	0.07088
C	-1.80000	3.26798	0.77340
C	0.43847	2.28599	-0.65152
C	-0.96063	4.11857	0.05337
H	-2.65185	3.67795	1.29945
C	0.14397	3.65130	-0.65938
H	-1.17607	5.17817	0.04454
H	0.75688	4.35010	-1.21297
C	1.53689	1.57172	-1.31727
C	2.58630	2.14649	-2.01275
C	3.57682	1.33034	-2.54857
H	2.63545	3.21982	-2.12523
C	2.43316	-0.56908	-1.66533
C	3.50639	-0.04245	-2.36968
H	4.40267	1.76930	-3.09181
H	2.33091	-1.62612	-1.46576
H	4.26736	-0.70313	-2.75832
C	-2.26246	0.82254	1.48316
C	-3.33075	0.97974	2.34881
C	-3.88807	-0.14159	2.95405
H	-3.71602	1.96742	2.55658
C	-2.28979	-1.50039	1.81606
C	-3.36065	-1.39651	2.69226
H	-4.72166	-0.02984	3.63409
H	-1.81337	-2.44333	1.58912
H	-3.75899	-2.28576	3.15825
N	-1.77726	-0.42508	1.22234
N	1.47668	0.21657	-1.17609
O	0.80695	-1.57812	0.92761

Pincer_Ph_terpy_oxo_t (*NImag* = 0)

Pd	0.03864	-0.44515	0.18831
C	-1.49858	1.90369	0.82507
C	-0.38000	1.44915	0.12711
C	-1.78968	3.26618	0.78174
C	0.45264	2.28837	-0.61262
C	-0.96462	4.11876	0.04800
H	-2.64258	3.67564	1.30661
C	0.14958	3.64852	-0.64731
H	-1.19545	5.17468	0.01732
H	0.76201	4.34684	-1.20219
C	1.56444	1.59286	-1.26330
C	2.54134	2.17118	-2.05975
C	3.53890	1.37759	-2.60944
H	2.51858	3.23494	-2.24639
C	2.54657	-0.51674	-1.55343
C	3.54463	0.01423	-2.35445
H	4.30416	1.82177	-3.23147
H	2.50348	-1.57215	-1.32483
H	4.30359	-0.63594	-2.76425
C	-2.22534	0.84549	1.52879
C	-3.36882	1.00535	2.29684
C	-3.94713	-0.09931	2.90716
H	-3.80089	1.98844	2.41449
C	-2.23033	-1.45901	1.96279
C	-3.37214	-1.35043	2.74000
H	-4.83906	0.01790	3.50761
H	-1.74342	-2.40987	1.79898
H	-3.79261	-2.23350	3.19833
N	-1.67548	-0.39513	1.37683
N	1.58809	0.24838	-1.02534
O	0.38975	-2.30113	0.17220

11. References

1. (a) Poverenov, E.; Efremenko, I.; Frenkel, A. I.; Ben-David, Y.; Shimon, L. J. W.; Leitus, G.; Konstantinovski, L.; Martin, J. M. L.; Milstein, D., *Nature* **2008,** *455*, 1093-1096. (b) Efremenko, I.; Poverenov, E.; Martin, J. M. L.; Milstein, D., *J. Am. Chem. Soc.* **2010,** *132*, 14886-14900.
2. Broclawik, E.; Yamauchi, R.; Endou, A.; Kubo, M.; Miyamoto, A., *J. Chem. Phys.* **1996,** *104*, 4098-4104.
3. Andrada, D. M.; Holzmann, N.; Hamadi, T.; Frenking, G., *Beilstein J. Org. Chem.* **2015,** *11*, 2727-2736.
4. (a) Gusev, D. G., *Organometallics* **2009,** *28*, 6458-6461. (b) Gusev, D. G., *Organometallics* **2009,** *28*, 763-770.

12. Full Gaussian Citation

M. J. Frisch, G. W. Trucks, H. B. Schlegel, G. E. Scuseria, M. A. Robb, J. R. Cheeseman, G. Scalmani, V. Barone, B. Mennucci, G. A. Petersson, H. Nakatsuji, M. Caricato, X. Li, H. P. Hratchian, A. F. Izmaylov, J. Bloino, G. Zheng, J. L. Sonnenberg, M. Hada, M. Ehara, K. Toyota, R. Fukuda, J. Hasegawa, M. Ishida, T. Nakajima, Y. Honda, O. Kitao, H. Nakai, T. Vreven, J. J. A. Montgomery, J. E. Peralta, F. Ogliaro, M. Bearpark, J. J. Heyd, E. Brothers, K. N. Kudin, V. N. Staroverov, R. Kobayashi, J. Normand, K. Raghavachari, A. Rendell, J. C. Burant, S. S. Iyengar, J. Tomasi, M. Cossi, N. Rega, J. M. Millam, M. Klene, J. E. Knox, J. B. Cross, V. Bakken, C. Adamo, J. Jaramillo, R. Gomperts, R. E. Stratmann, O. Yazyev, A. J. Austin, R. Cammi, C. Pomelli, J. W. Ochterski, R. L. Martin, K. Morokuma, V. G. Zakrzewski, G. A. Voth, P. Salvador, J. J. Dannenberg, S. Dapprich, A. D. Daniels, Ö. Farkas, J. B. Foresman, J. V. Ortiz, J. Cioslowski and D. J. Fox in *Gaussian 09, Rev. D01*, Gaussian Inc., Wallingford CT, **2009**.
